# Supplementary material for: Accounting for age of onset and family history improves power in genome-wide association studies
Source: Am J Hum Genet. 2022 Feb 8;109(3):417–32. doi: 10.1016/j.ajhg.2022.01.009 (PMC8948165; doi:10.1016/j.ajhg.2022.01.009)
Supplement: Document S1. Figures S1–S50 and Tables S1–S12, S14, and S15 [file mmc1.pdf]

The American Journal of Human Genetics, Volume *109*

Supplemental information

Accounting for age of onset and family history improves power in genome-wide association studies

Emil M. Pedersen, Esben Agerbo, Oleguer Plana-Ripoll, Jakob Grove, Julie W. Dreier, Katherine L. Musliner, Marie Bækvad-Hansen, Georgios Athanasiadis, Andrew Schork, Jonas Bybjerg-Grauholm, David M. Hougaard, Thomas Werge, Merete Nordentoft, Ole Mors, Søren Dalsgaard, Jakob Christensen, Anders D. Børglum, Preben B. Mortensen, John J. McGrath, Florian Privé, and Bjarni J. Vilhjálmsson

Supplemental Information

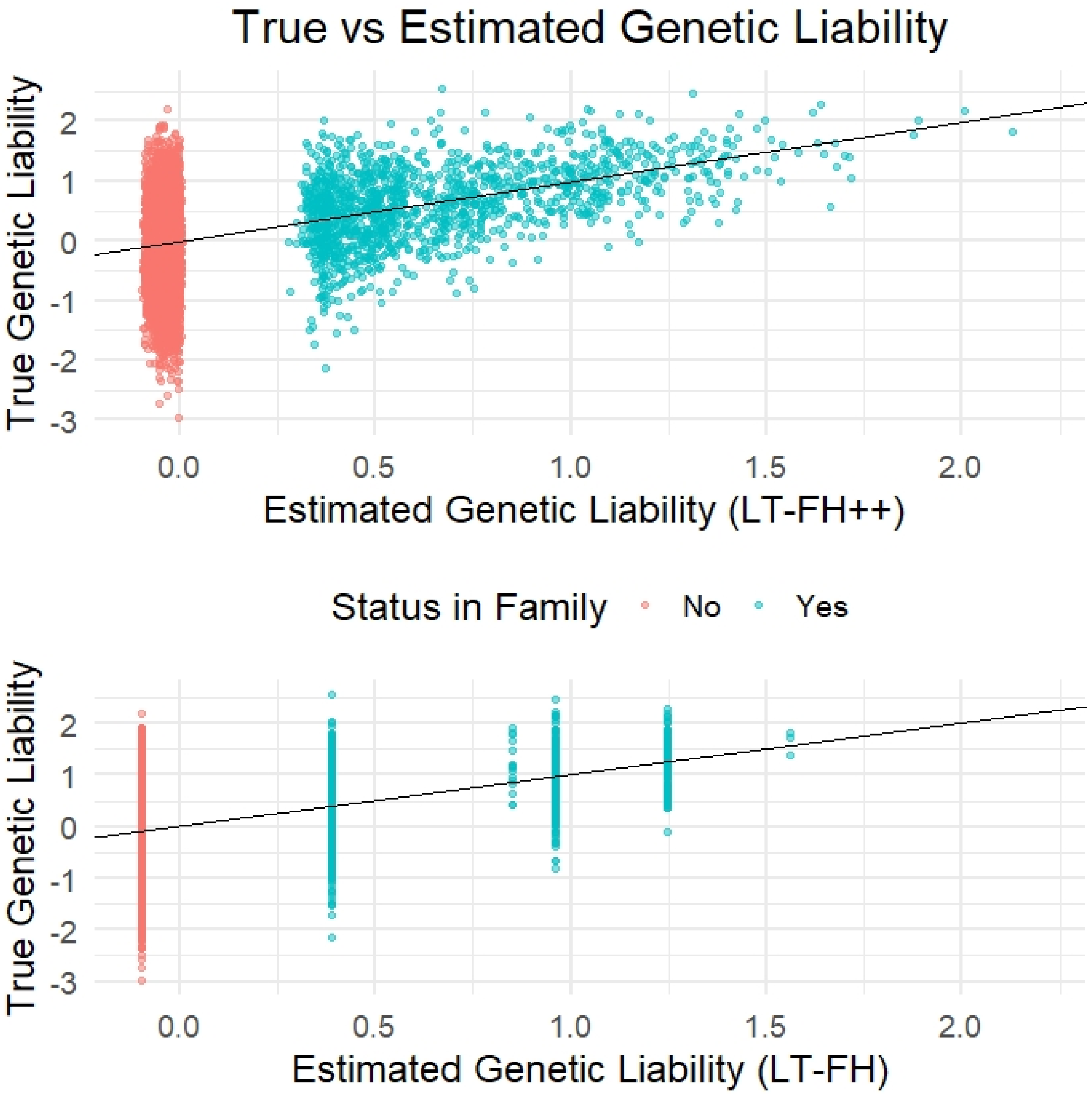

Figure S1: Simulated genetic liabilities assuming two parents and 0 siblings, a heritability of 50% and a prevalence of 10% (see Methods for details). We see that LT-FH estimates for the genetic liabilities fall into specific groups, depending on the case status of the individual and family

members. LT-FH++ takes age into account to obtain a more refined prediction of the genetic liability.

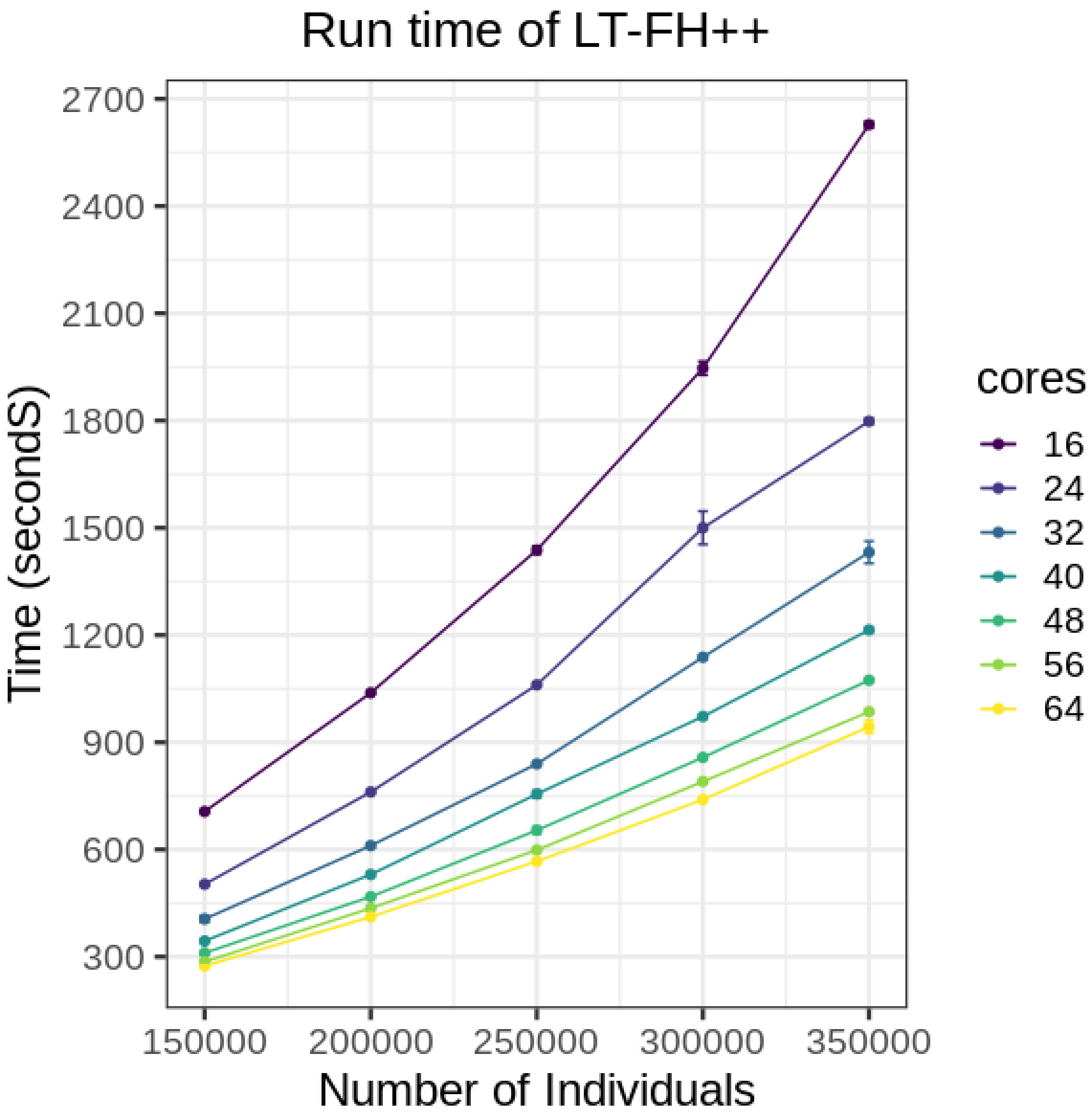

Figure S2: Plot of computation times of LT-FH++ with varying number of cores and individuals. This plot shows computation times for more than 100k individuals and 16 to 64 cores. Error bars correspond to the standard error of the times multiplied by 1.96.

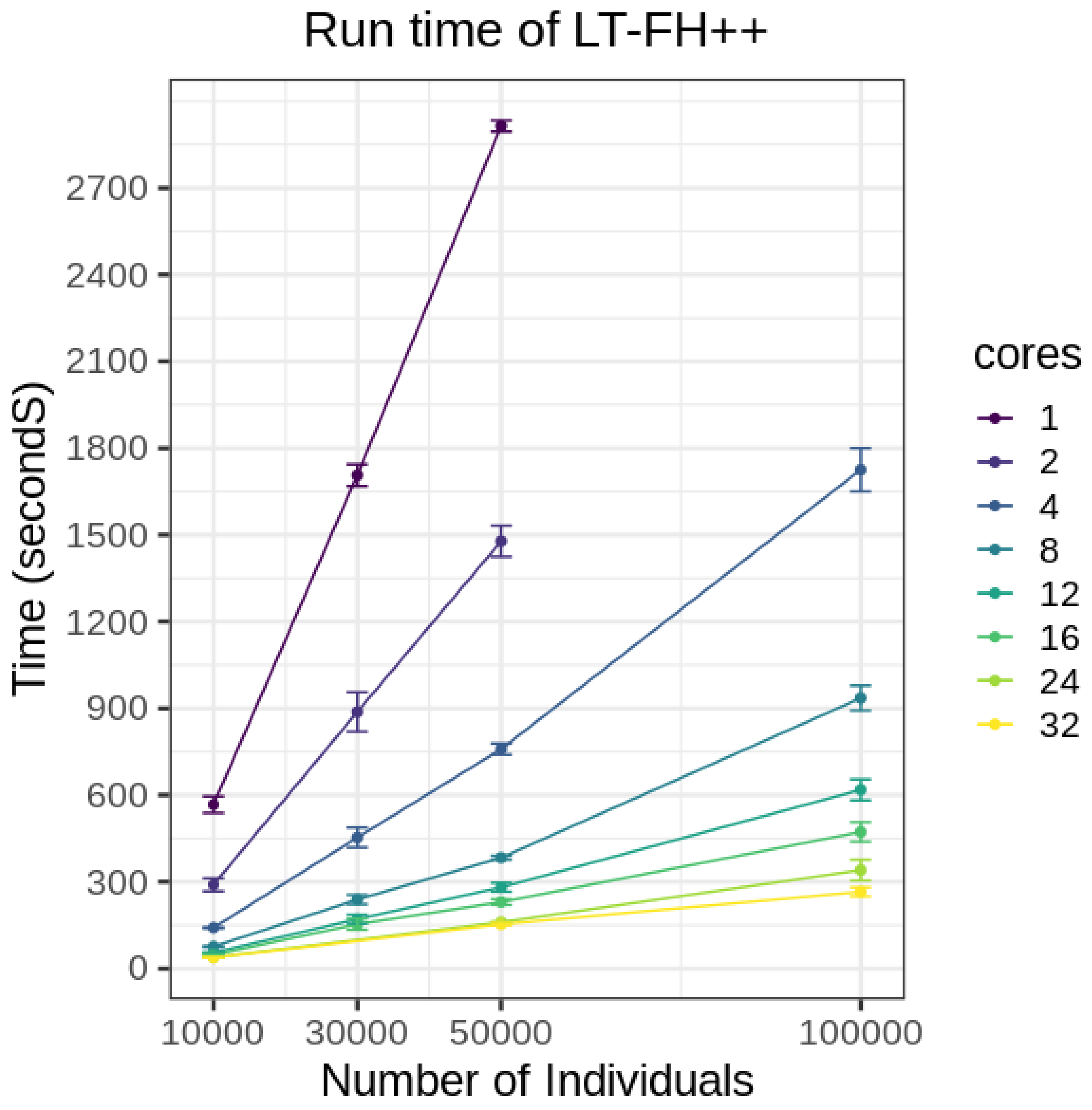

Figure S3: Plot of computation times of LT-FH++ with varying number of cores and individuals. This plot shows computation times for at most 100k individuals and 1 to 32 cores. Error bars correspond to the standard error of the times multiplied by 1.96.

Simulation Results

Simulation Results: 5% Prevalence

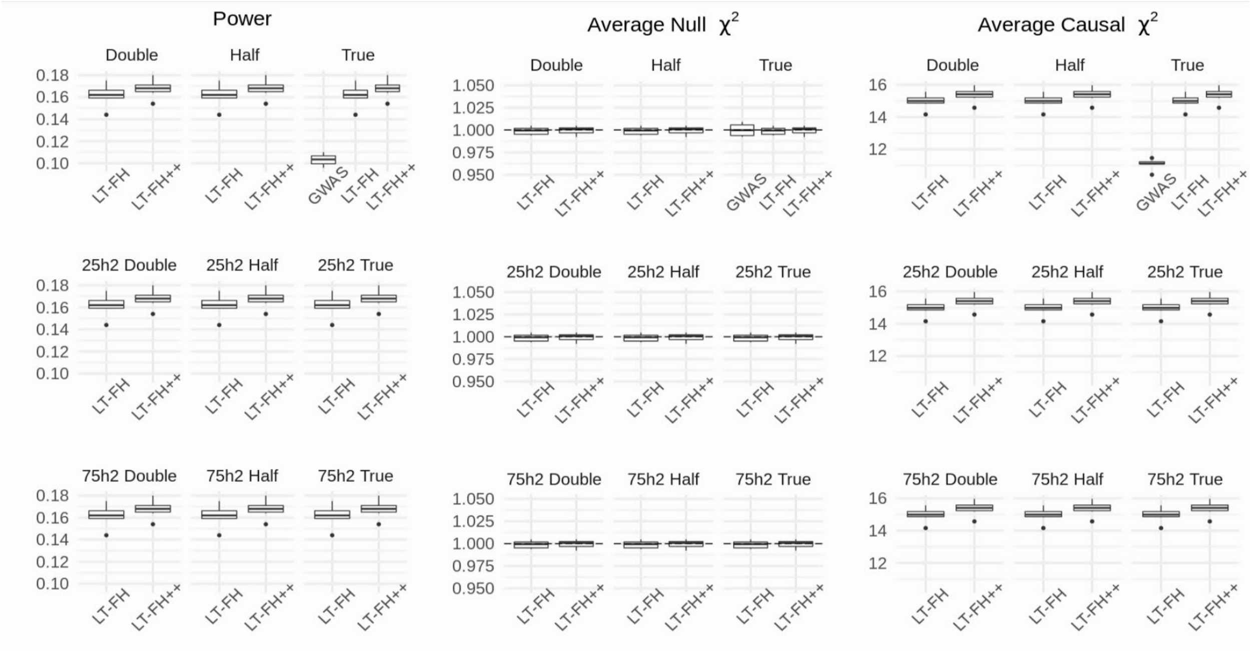

Figure S4: Simulation results with misspecified parameters and a prevalence of 5%. “Half” and “Double” refers to the misspecified prevalence, where “Half” means half of the true prevalence was used, and “Double” means double of the true prevalence was used. For reference, we added “True”, which is the true prevalence. If no heritability is specified in a subplot’s title, the default heritability of 50% was used. The true underlying heritability remains 50%.

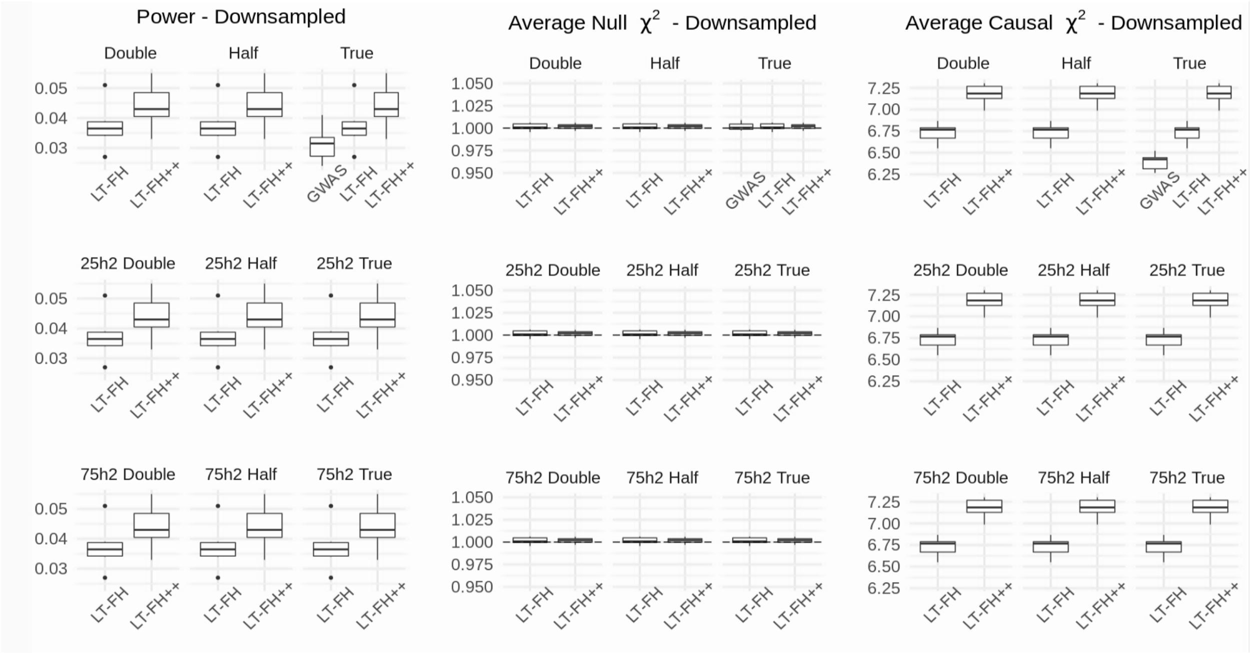

Figure S5: Simulation results with misspecified parameters, a prevalence of 5%, and downsampling of controls. “Half” and “Double” refers to the misspecified prevalence, and “Half” means half of the true prevalence was used, and “Double” means double of the true prevalence was used. For reference, we added “True”, which is the true prevalence. If no heritability is specified in a subplot’s title, the default heritability of 50% was used. The true underlying heritability remains 50%.

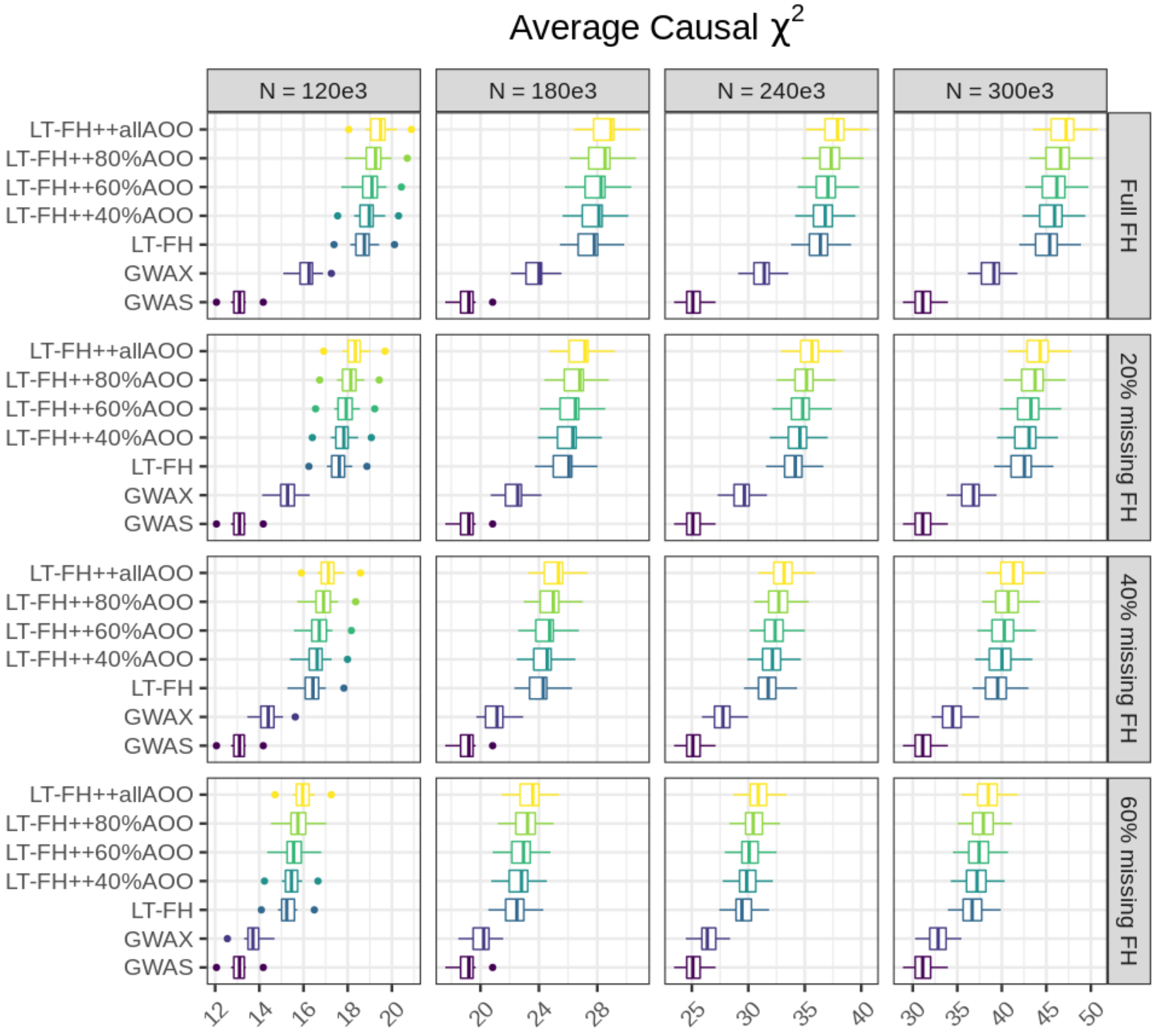

Figure S6: Simulation results of varying degrees of missingness in family history and age-of-onset. The simulation setup used is the default setting, with a prevalence of 5%, varying the number of individuals between 120k and 300k in steps of 60k, and family history and age-of-onset available for everyone to 40% in steps of 20% for each method (where applicable). Family history and age-of-onset information is removed at random, but for the same individuals across phenotypes.

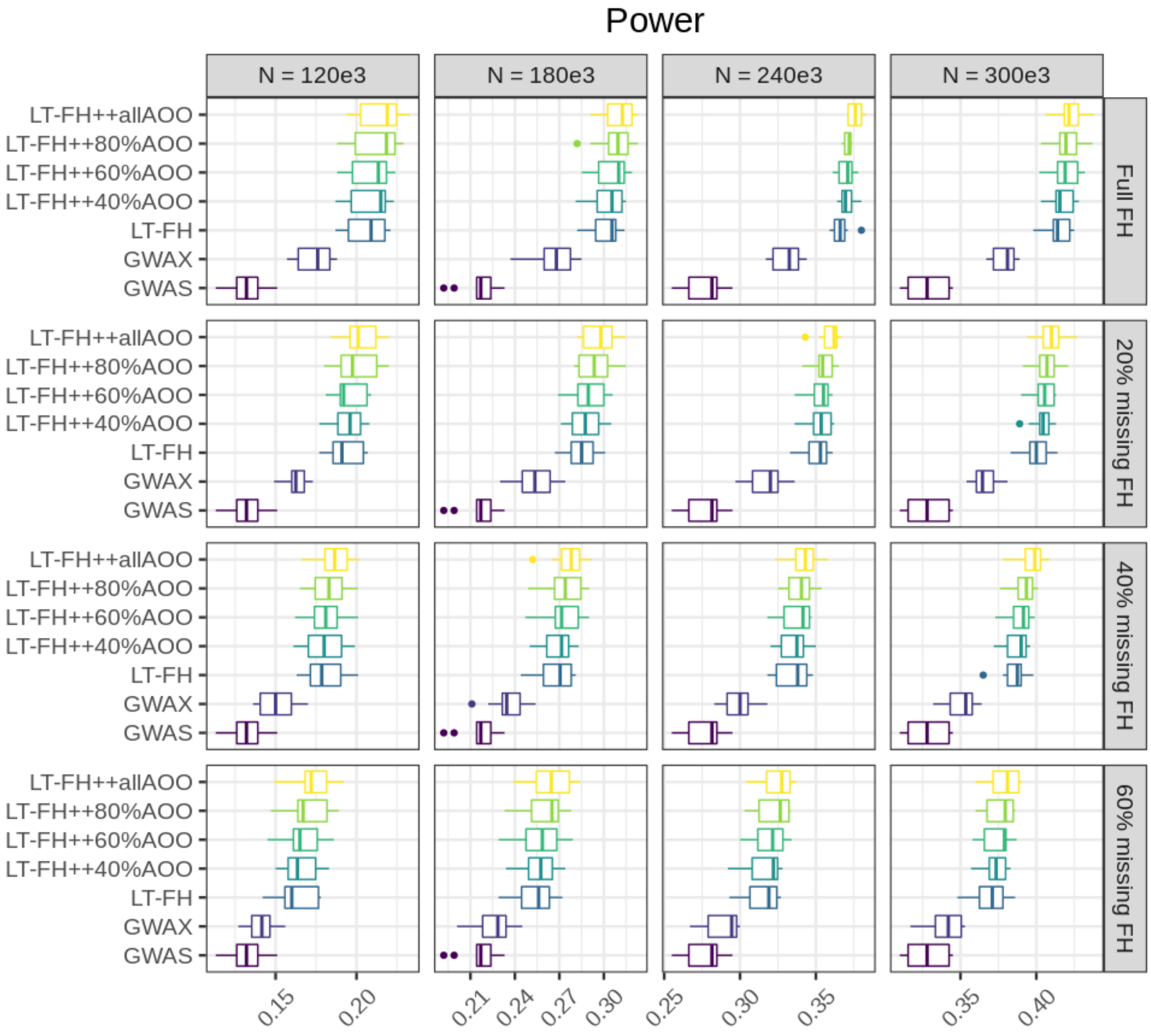

Figure S7: Simulation results of varying degrees of missingness in family history and age-of-onset. The simulation setup used is the default setting, with a prevalence of 5%, varying the number of individuals between 120k and 300k in steps of 60k, and family history and age-of-onset available for everyone to 40% in steps of 20% for each method (where applicable). Family history and age-of-onset information is removed at random, but for the same individuals across phenotypes.

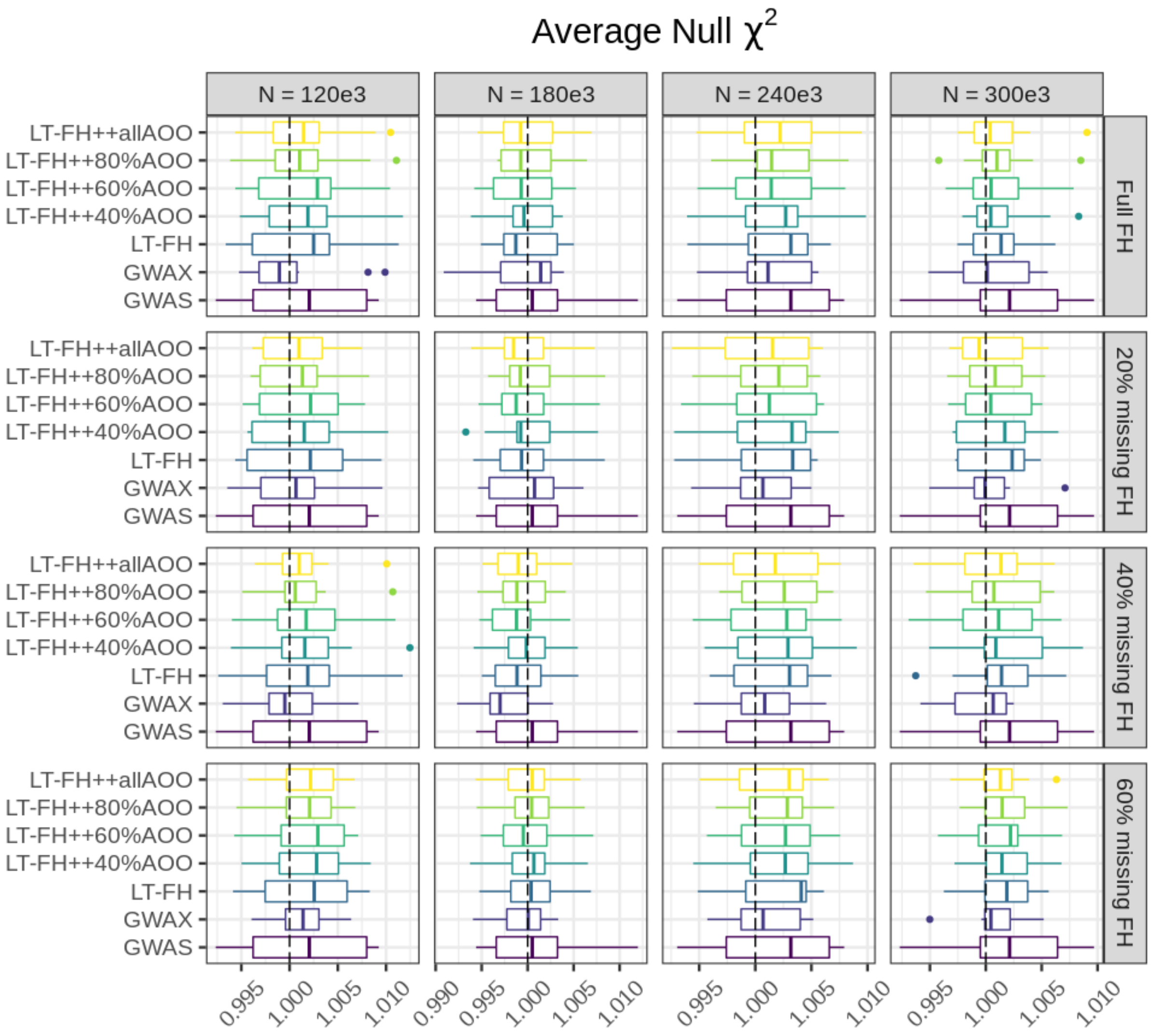

Figure S8: Simulation results of varying degrees of missingness in family history and age-of-onset. The simulation setup used is the default setting, with a prevalence of 5%, varying the number of individuals between 120k and 300k in steps of 60k, and family history and age-of-onset available for everyone to 40% in steps of 20% for each method (where applicable). Family history and age-of-onset information is removed at random, but for the same individuals across phenotypes.

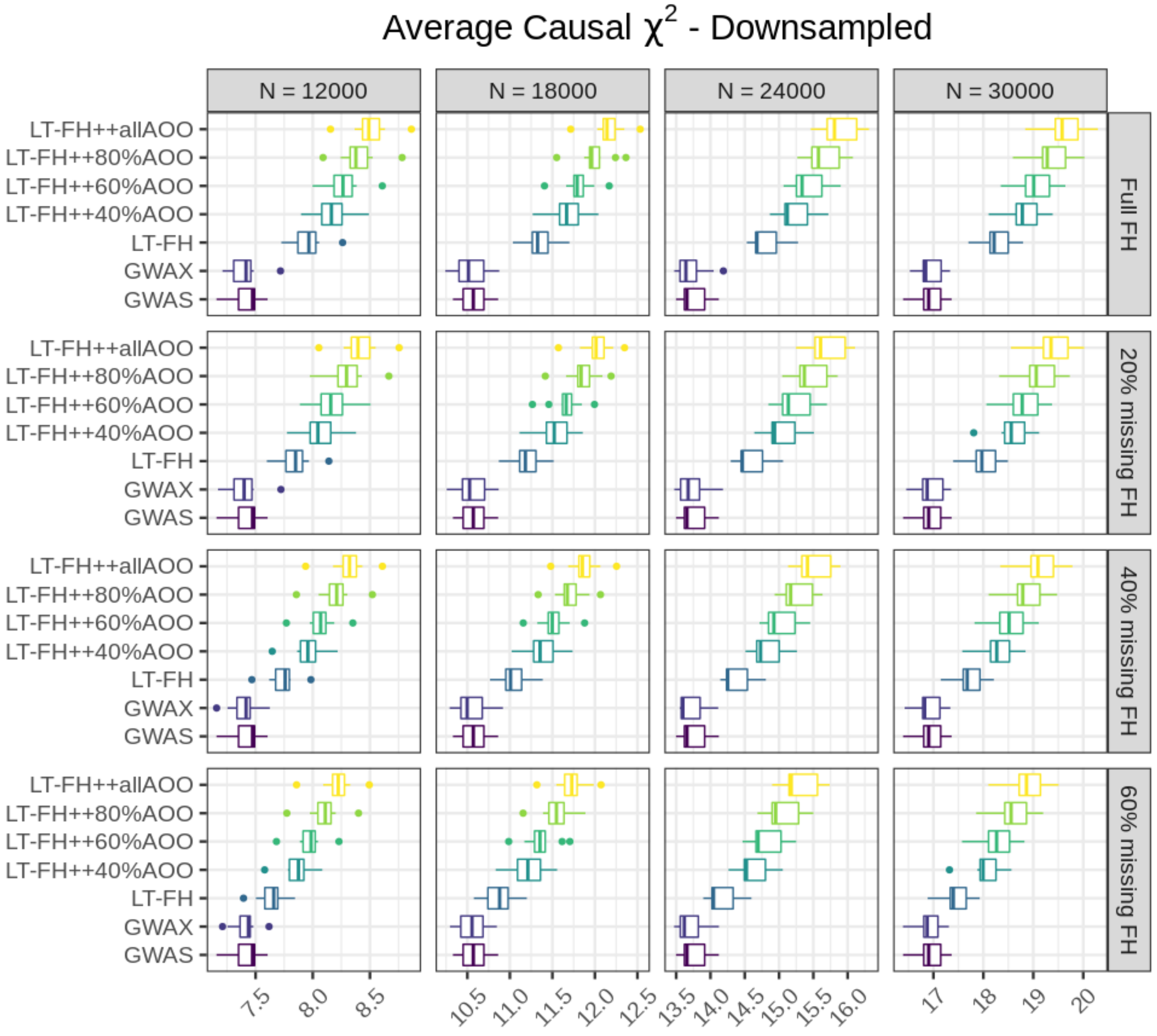

Figure S9: Simulation results of varying degrees of missingness in family history and age-of-onset when downsampling controls. The simulation setup used is the default setting, with a prevalence of 5%, varying the number of individuals between 12k and 30k in steps of 6k, and family history and age-of-onset available for everyone to 40% in steps of 20% for each method (where applicable). Family history and age-of-onset information is removed at random, but for the same individuals across phenotypes.

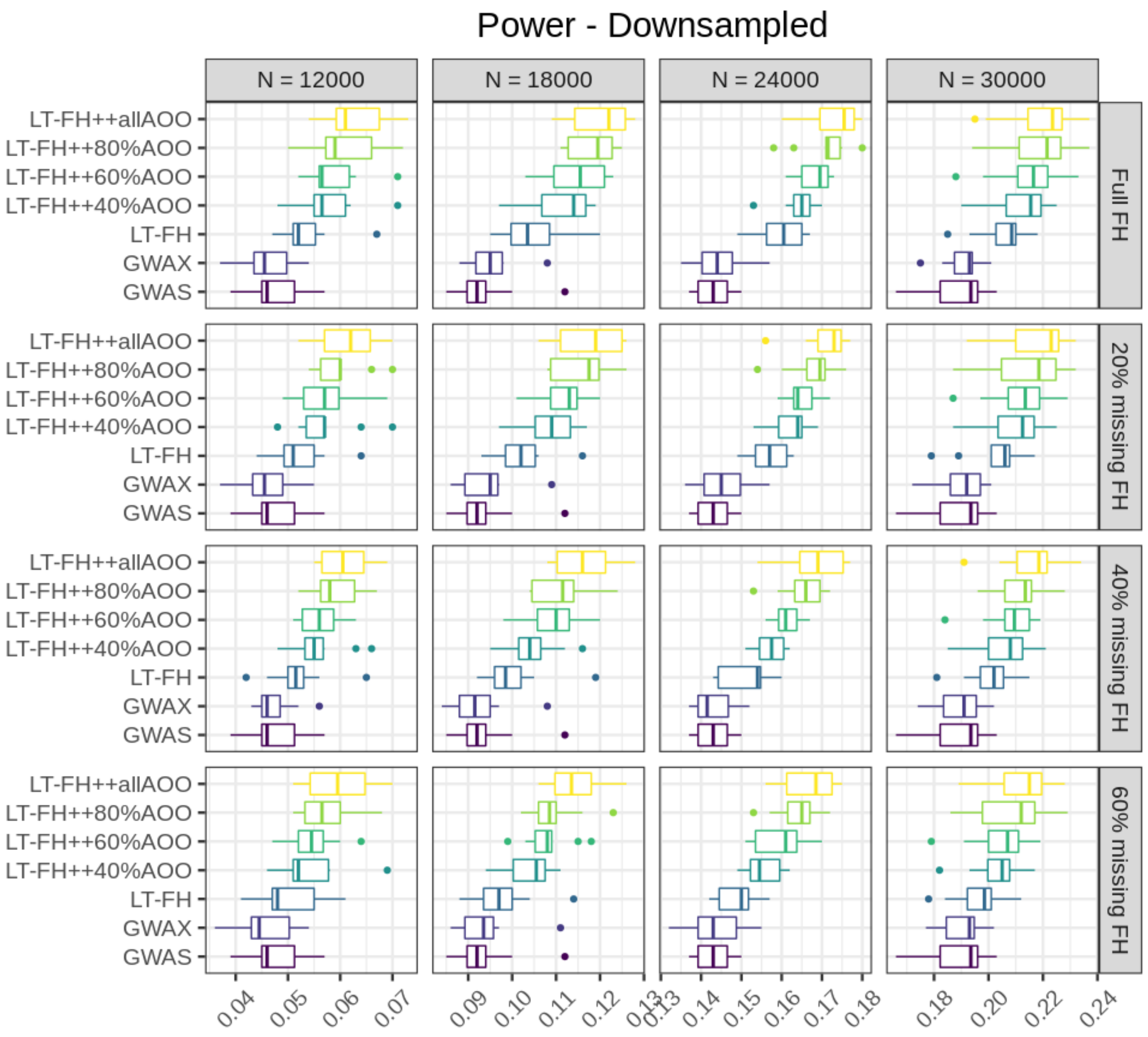

Figure S10: Simulation results of varying degrees of missingness in family history and age-of-onset when downsampling controls. The simulation setup used is the default setting, with a prevalence of 5%, varying the number of individuals between 12k and 30k in steps of 6k, and family history and age-of-onset available for everyone to 40% in steps of 20% for each method (where applicable). Family history and age-of-onset information is removed at random, but for the same individuals across phenotypes.

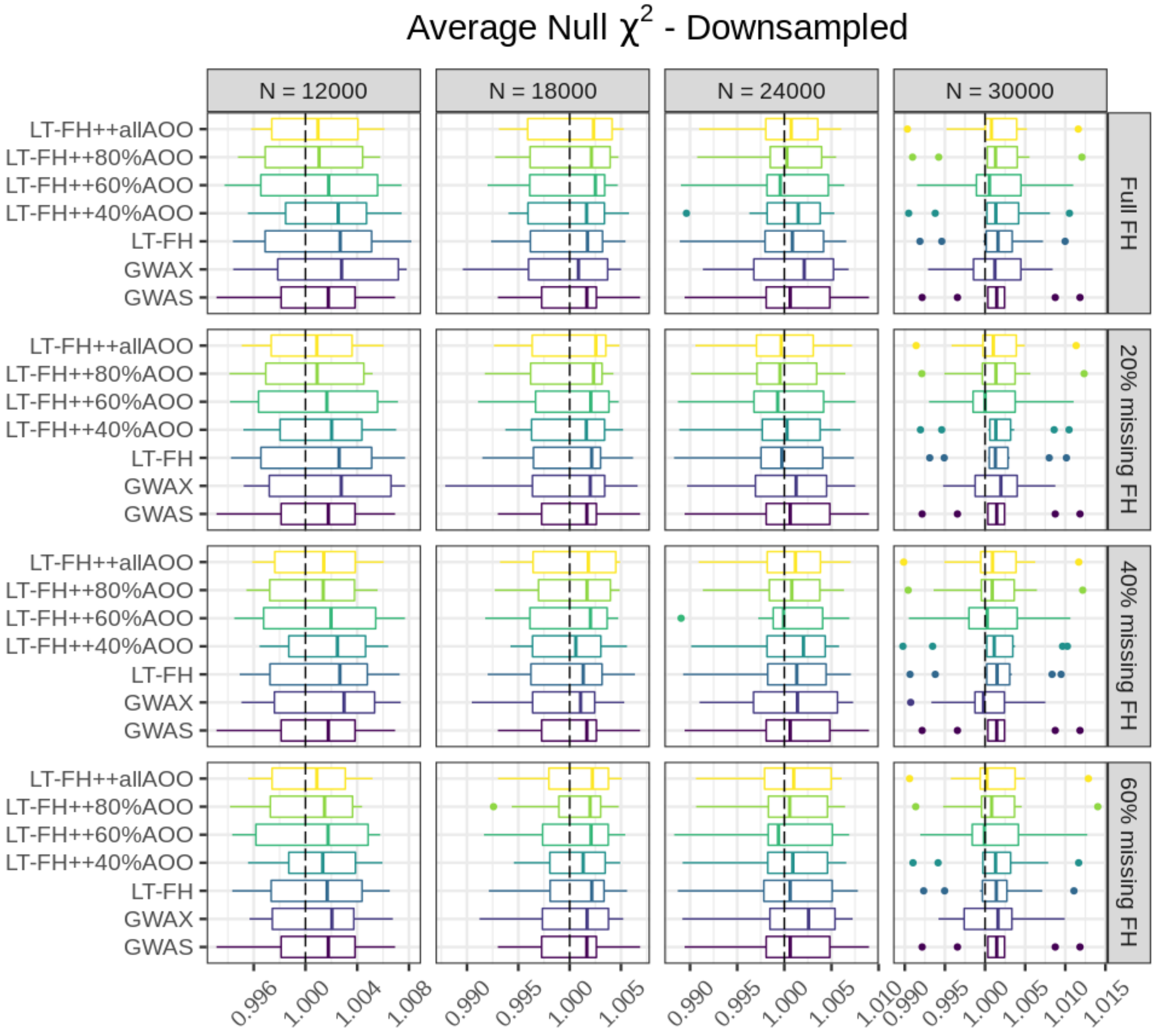

Figure S11: Simulation results of varying degrees of missingness in family history and age-of-onset when downsampling controls. The simulation setup used is the default setting, with a prevalence of 5%, varying the number of individuals between 12k and 30k in steps of 6k, and family history and age-of-onset available for everyone to 40% in steps of 20% for each method (where applicable). Family history and age-of-onset information is removed at random, but for the same individuals across phenotypes.

Simulation Results: 10% Prevalence

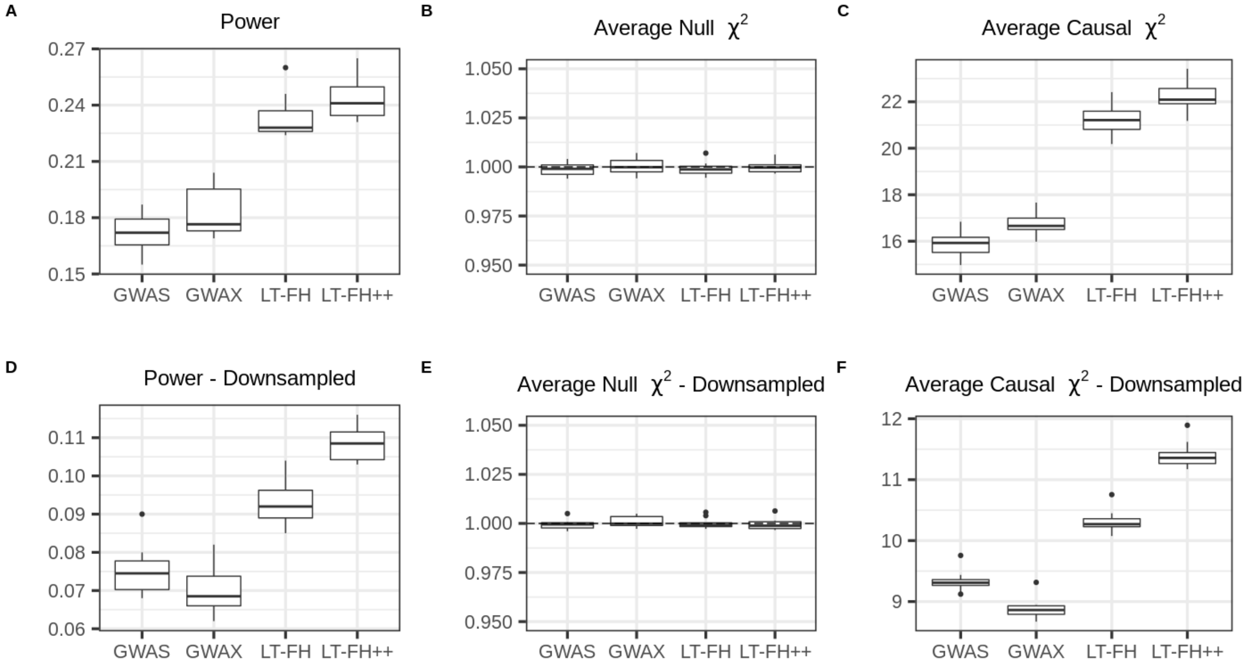

Figure S12: Simulation results under the default simulation parameters and a prevalence of 10%.

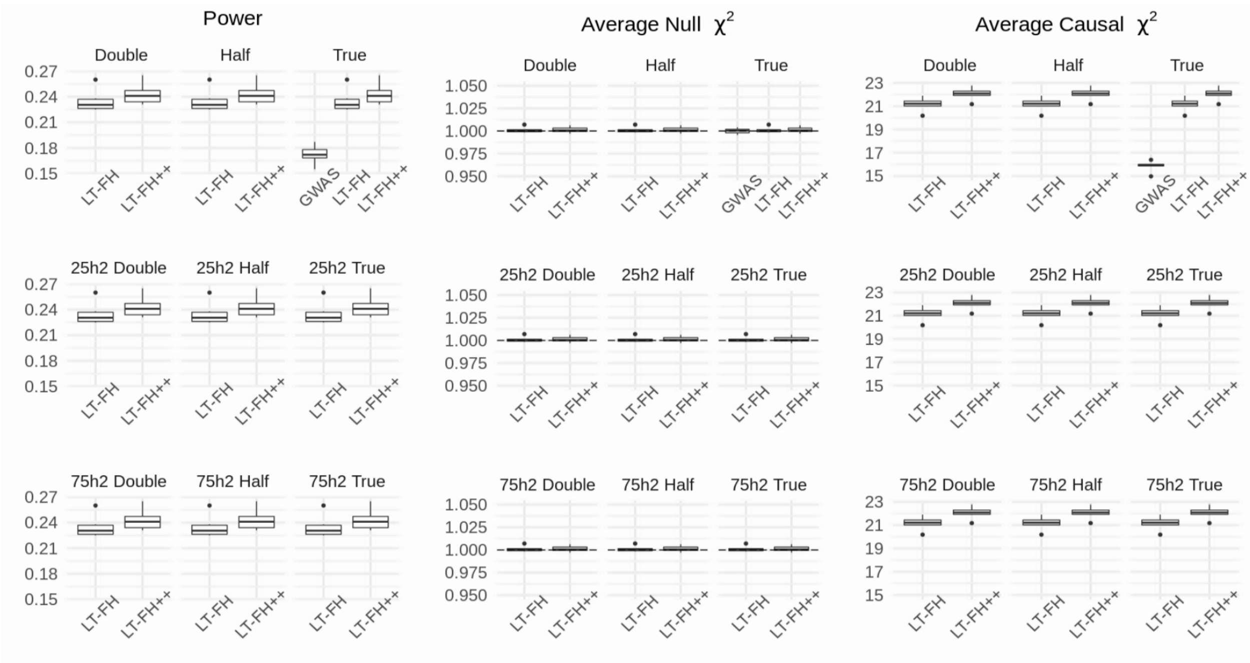

Figure S13: Simulation results with misspecified parameters and a prevalence of 10%. “Half” and “Double” refers to the misspecified prevalence, and “Half” means half of the true prevalence was used, and “Double” means double of the true prevalence was used. For reference, we added “True”, which is the true prevalence. If no heritability is specified in a subplot’s title, the default heritability of 50% was used. The true underlying heritability remains 50%.

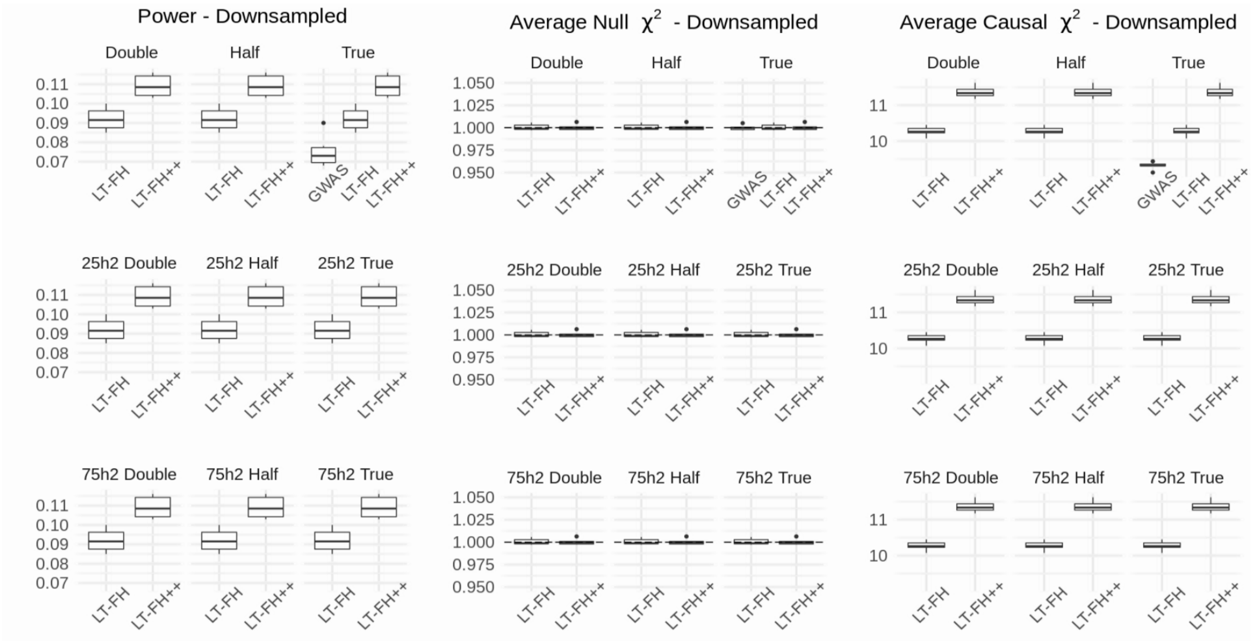

Figure S14: Simulation results with misspecified parameters, a prevalence of 10%, and downsampling of controls. “Half” and “Double” refers to the misspecified prevalence, and “Half” means half of the true prevalence was used, and “Double” means double of the true prevalence was used. For reference, we added “True”, which is the true prevalence. If no heritability is specified in a subplot’s title, the default heritability of 50% was used. The true underlying heritability remains 50%.

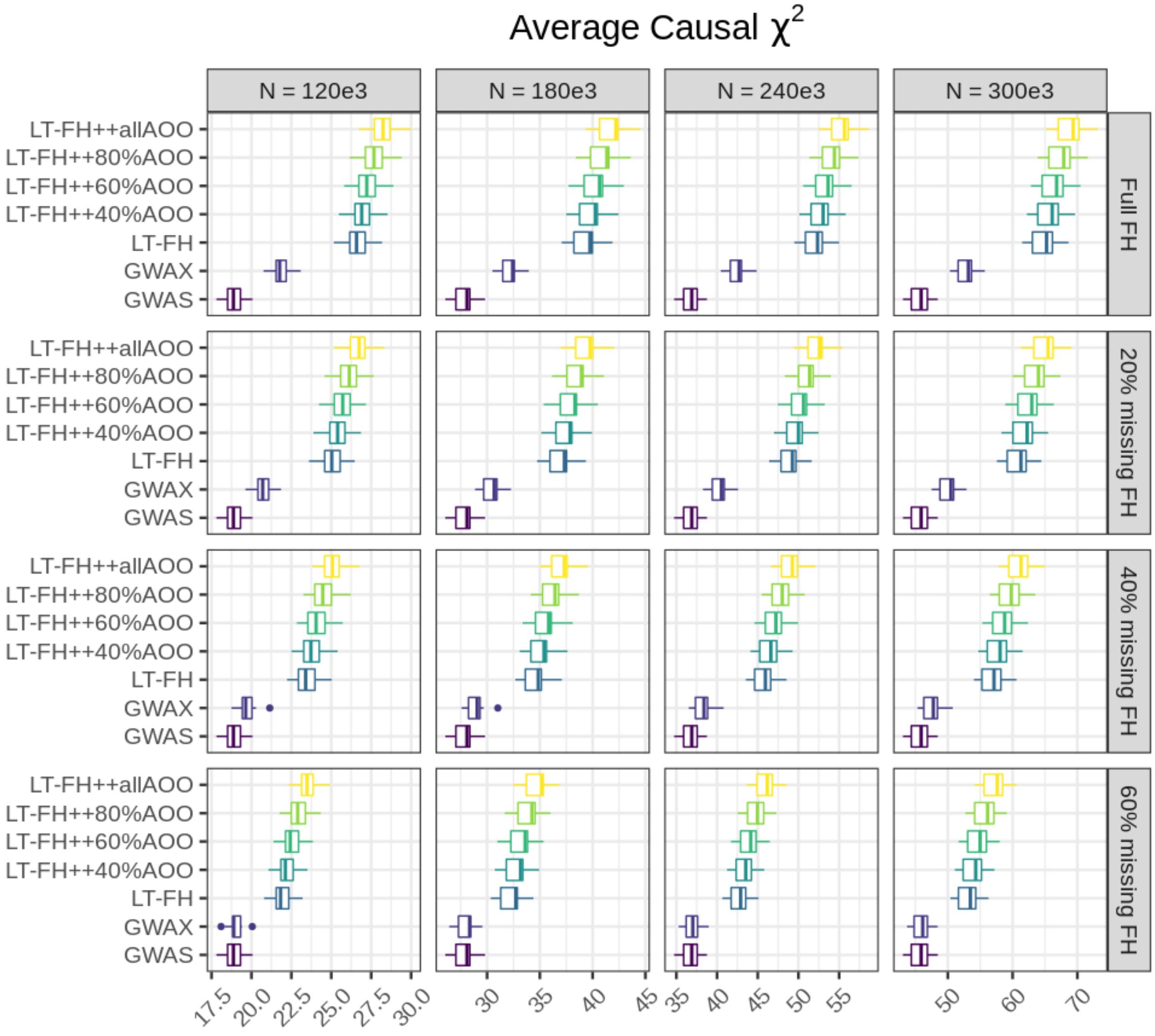

Figure S15: Simulation results of varying degrees of missingness in family history and age-of-onset. The simulation setup used is the default setting, with a prevalence of 10%, varying the number of individuals between 120k and 300k in steps of 60k, and family history and age-of-onset available for everyone to 40% in steps of 20% for each method (where applicable). Family history and age-of-onset information is removed at random, but for the same individuals across phenotypes.

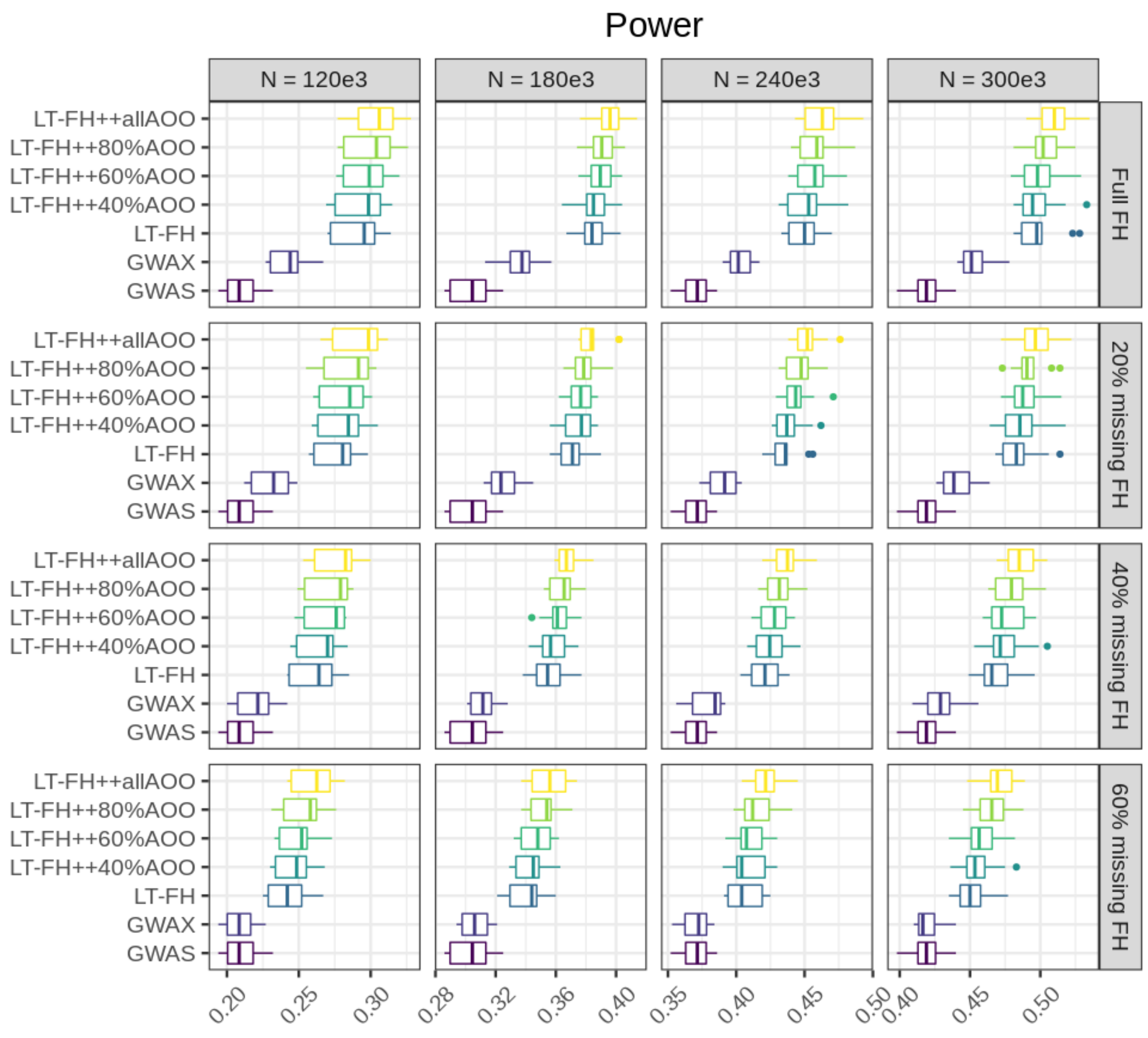

Figure S16: Simulation results of varying degrees of missingness in family history and age-of-onset. The simulation setup used is the default setting, with a prevalence of 10%, varying the number of individuals between 120k and 300k in steps of 60k, and family history and age-of-onset available for everyone to 40% in steps of 20% for each method (where applicable). Family history and age-of-onset information is removed at random, but for the same individuals across phenotypes.

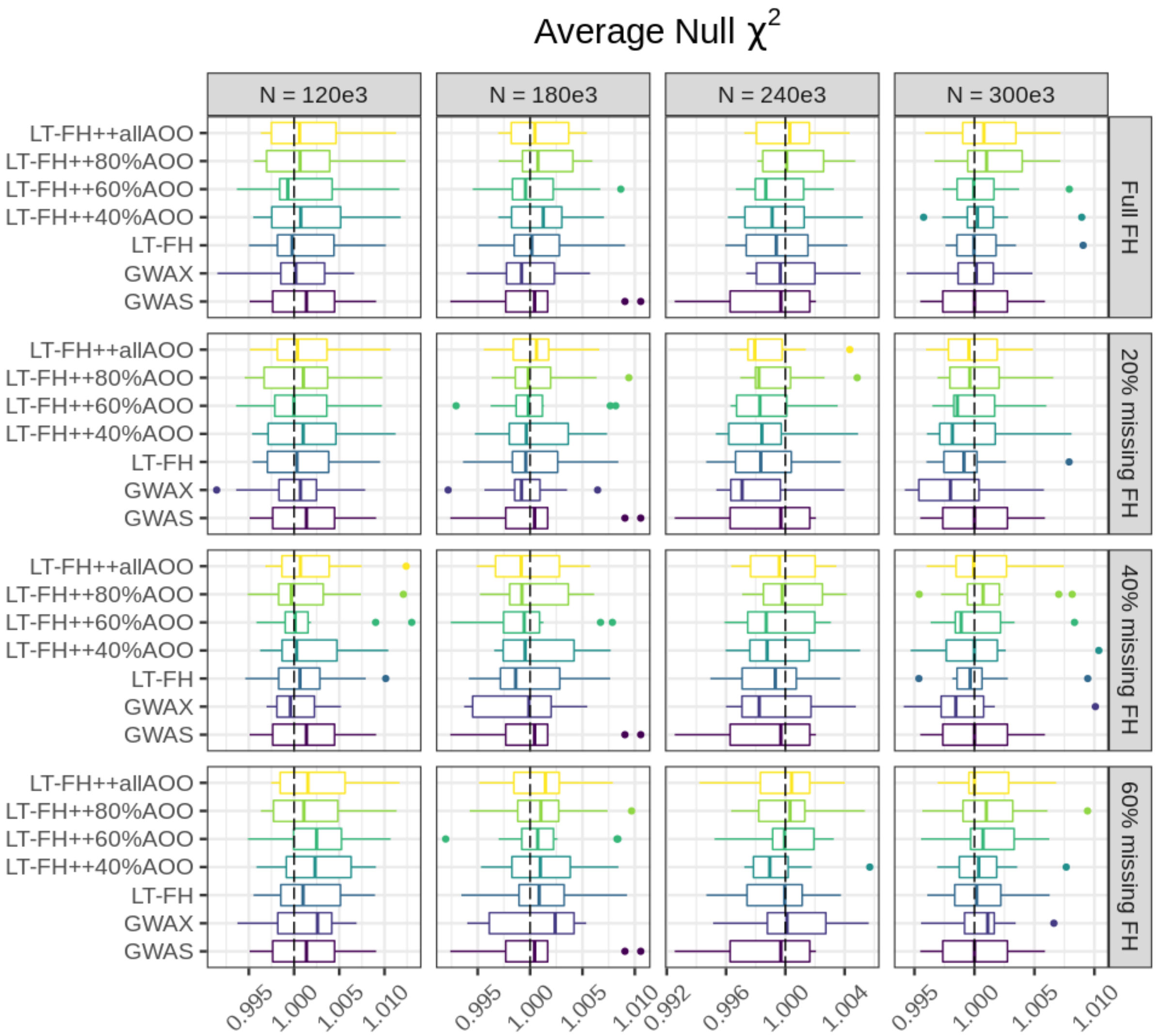

Figure S17: Simulation results of varying degrees of missingness in family history and age-of-onset. The simulation setup used is the default setting, with a prevalence of 10%, varying the number of individuals between 120k and 300k in steps of 60k, and family history and age-of-onset available for everyone to 40% in steps of 20% for each method (where applicable). Family history and age-of-onset information is removed at random, but for the same individuals across phenotypes.

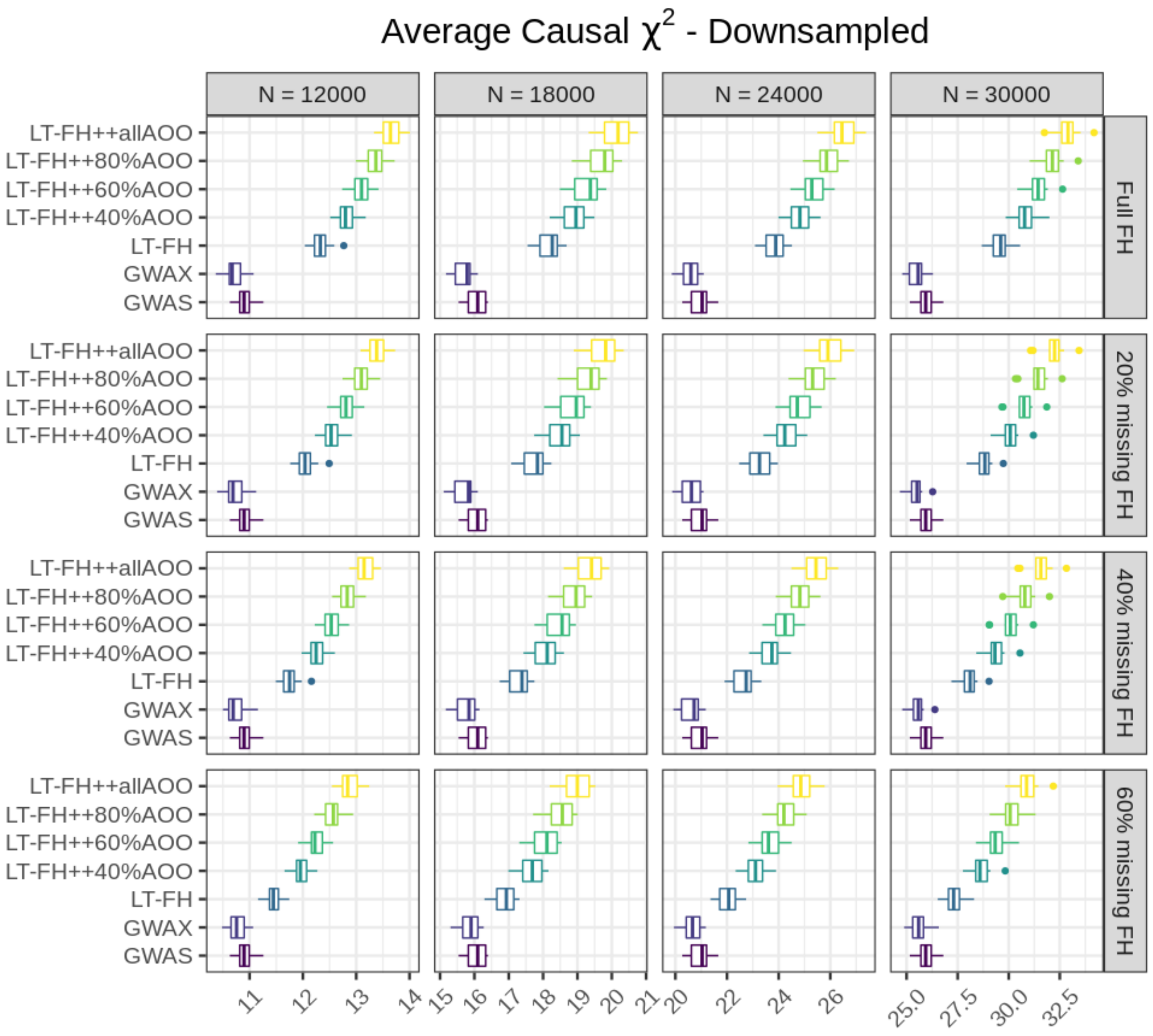

Figure S18: Simulation results of varying degrees of missingness in family history and age-of-onset when downsampling controls. The simulation setup used is the default setting, with a prevalence of 10%, varying the number of individuals between 12k and 30k in steps of 6k, and family history and age-of-onset available for everyone to 40% in steps of 20% for each method (where applicable). Family history and age-of-onset information is removed at random, but for the same individuals across phenotypes.

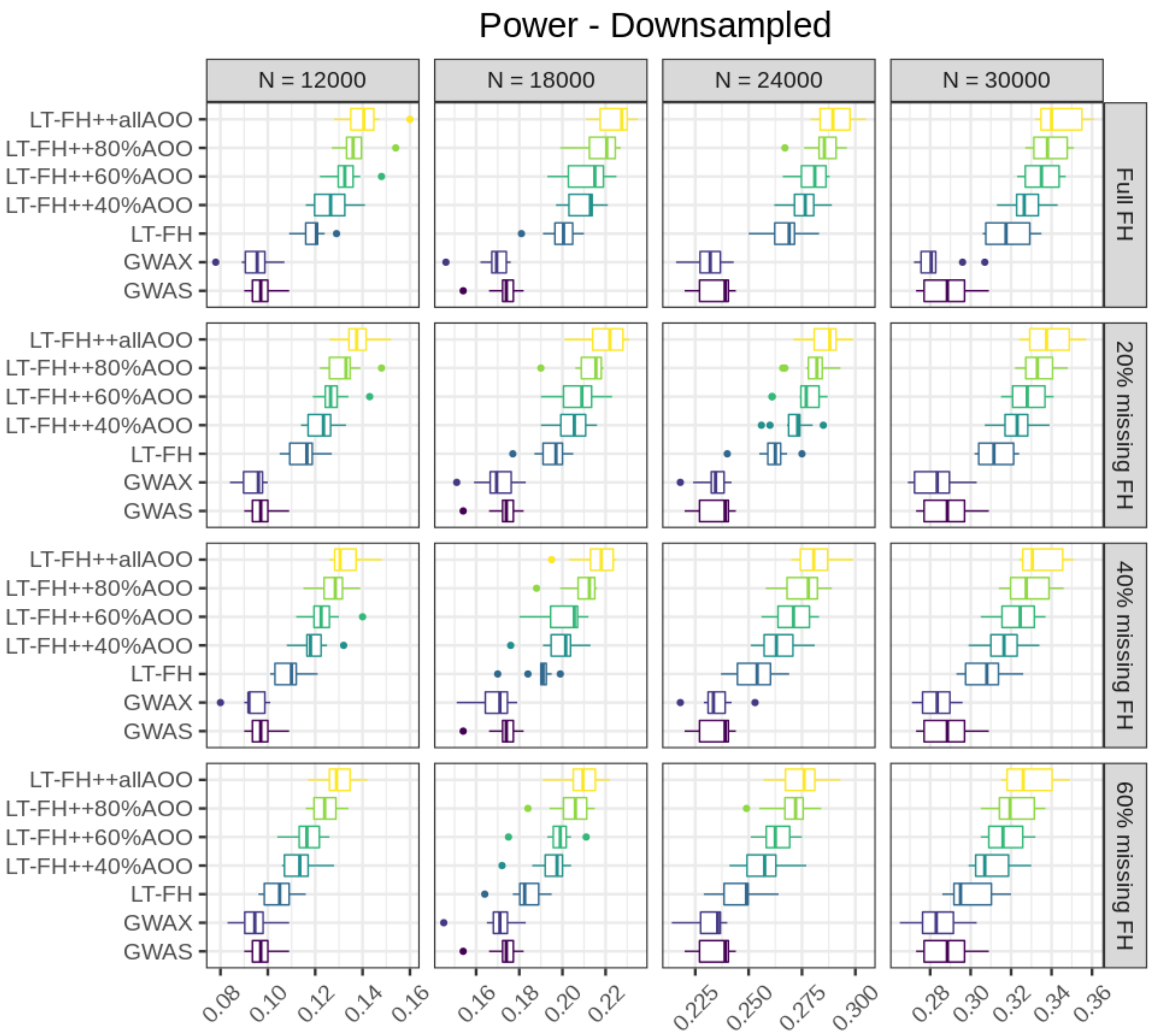

Figure S19: Simulation results of varying degrees of missingness in family history and age-of-onset when downsampling controls. The simulation setup used is the default setting, with a prevalence of 5%, varying the number of individuals between 12k and 30k in steps of 6k, and family history and age-of-onset available for everyone to 40% in steps of 20% for each method (where applicable). Family history and age-of-onset information is removed at random, but for the same individuals across phenotypes.

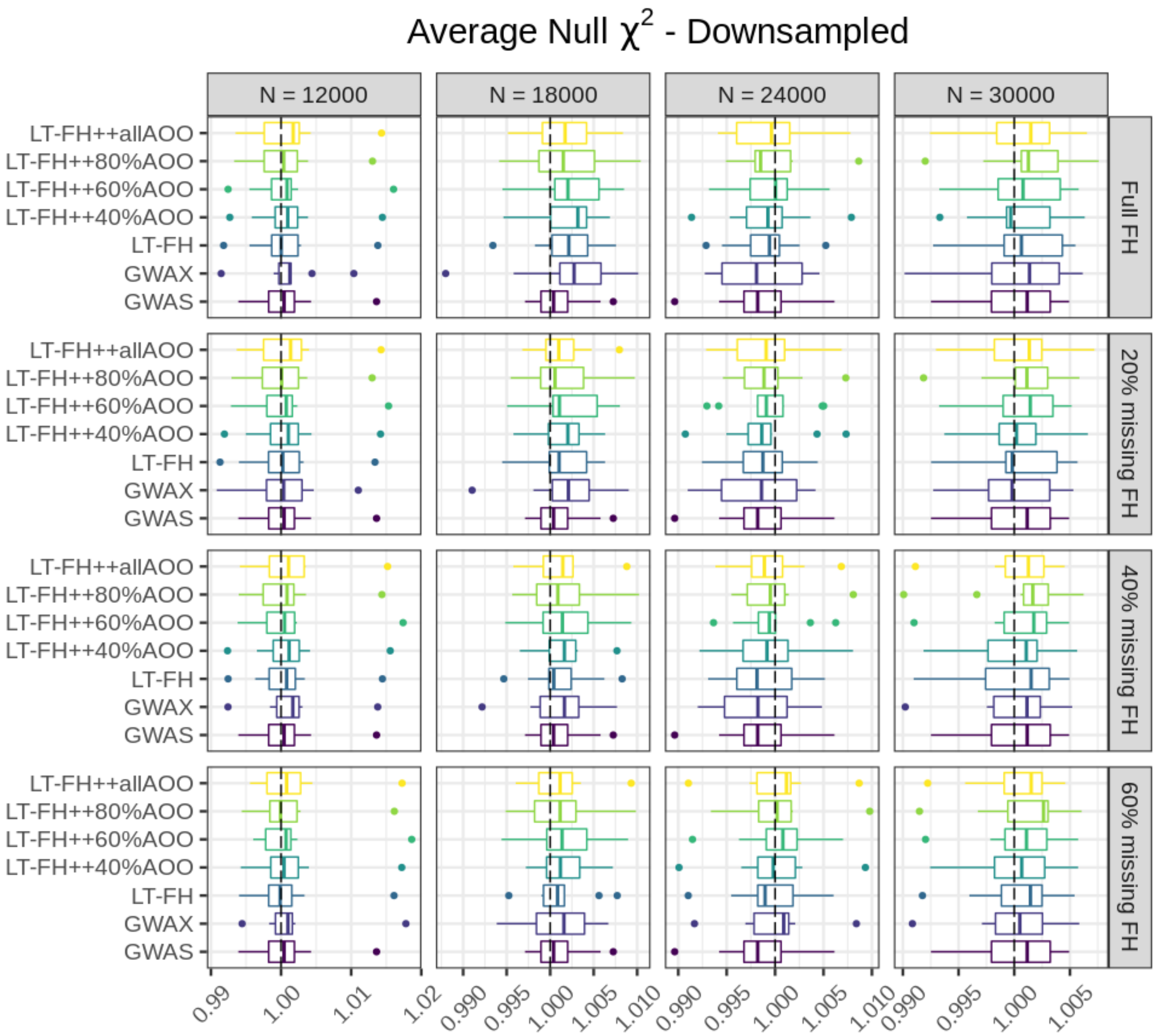

Figure S20: Simulation results of varying degrees of missingness in family history and age-of-onset when downsampling controls. The simulation setup used is the default setting, with a prevalence of 5%, varying the number of individuals between 12k and 30k in steps of 6k, and family history and age-of-onset available for everyone to 40% in steps of 20% for each method (where applicable). Family history and age-of-onset information is removed at random, but for the same individuals across phenotypes.

Mortality Results

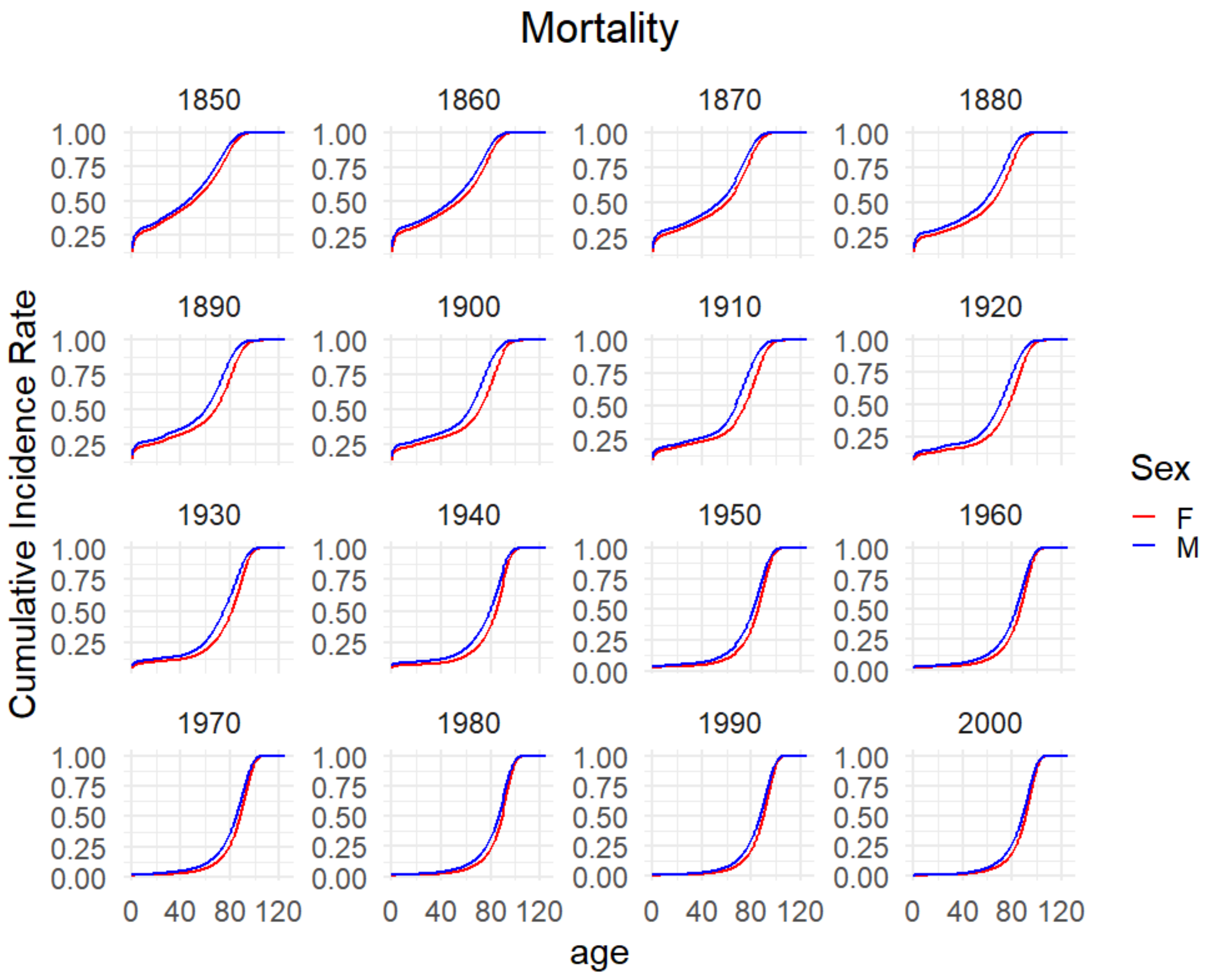

Figure S21: Plot of mortality from England and Wales, obtained from the Office for National Statistics (ONS). We plotted the cumulative mortality for each sex and from the begining of each decade from 2000 to the begining of the data. Historic mortality rates have been used upto the present, and projections for future predictions.

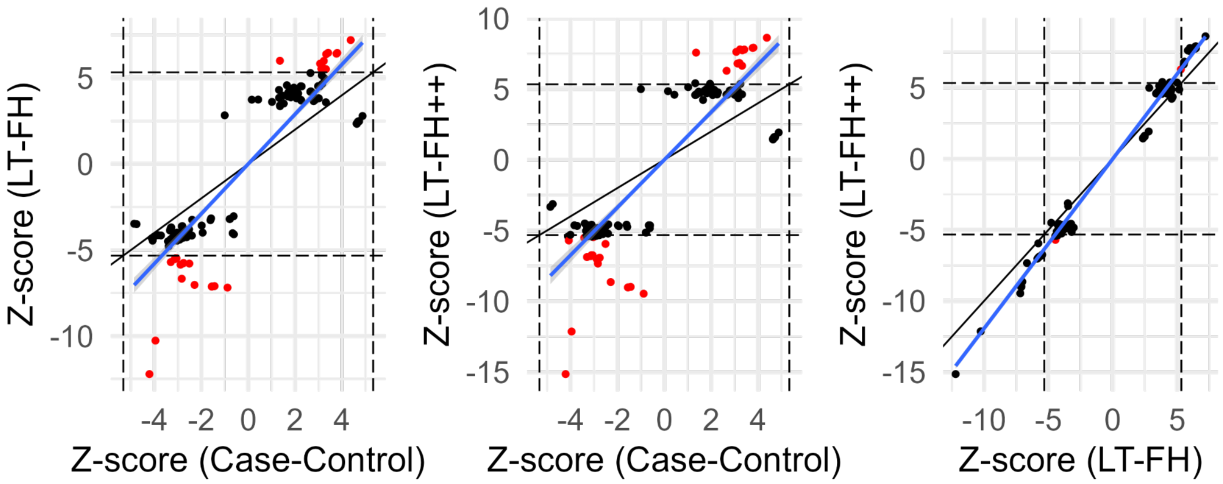

Figure S22: Z-scores for mortality in the UK biobank. We filtered on variants that had a p-value < 5×10^{-6} for at least one of the three compared outcomes. The common set of variants were LD clumped (prioritizing on minor allele frequencies) in an attempt to not bias one outcome over another. The dashed line correspond to a p-value of 5×10^{-8}, and the red dots are SNPs that are genome-wide significant for only one method. The black line is the identity line and the blue line is the best fitted line. We filtered on the p-values, keeping SNPs that are below 5×10^{-6} for at least one of the compared methods and performed . The squared slope of the fitted line indicates the power improvement of one method over another

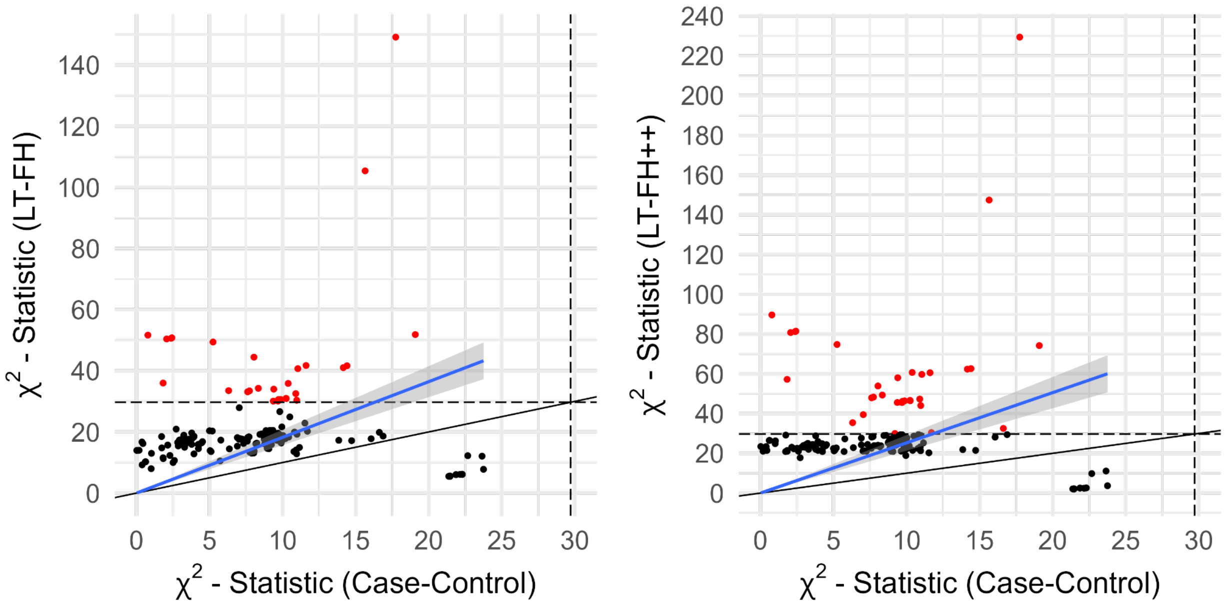

Figure S23: The χ^2 statistics for mortality between case-control status and LT-FH and LT-FH++ can be seen above. We filtered on variants that had a p-value $< 5 \times 10^{-6}$ for at least one of the three compared outcomes. The common set of variants were LD clumped (prioritizing on minor allele frequencies) in an attempt to not bias one outcome over another. The red dots are variants identified as genome-wide significant for only one of the outcomes. The black dots are suggestive associations identified by either method. The black line indicates the identity line and the blue line is the best fitted line using linear regression. The black dashed lines correspond to the threshold for genome-wide significance.

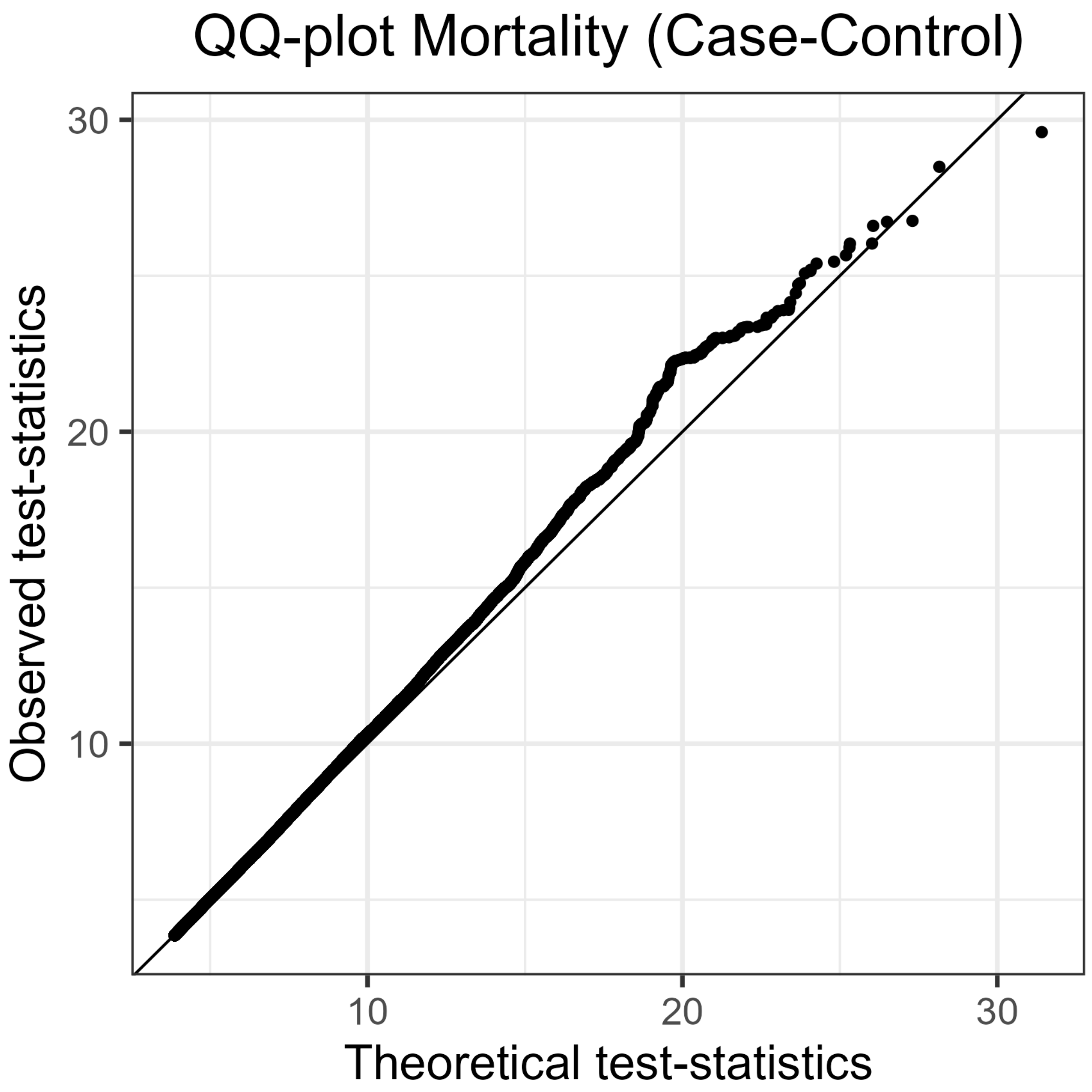

Figure S24: QQ plot of Mortality for Case-Control status. We excluded SNPs with p-values greater than 0.05.

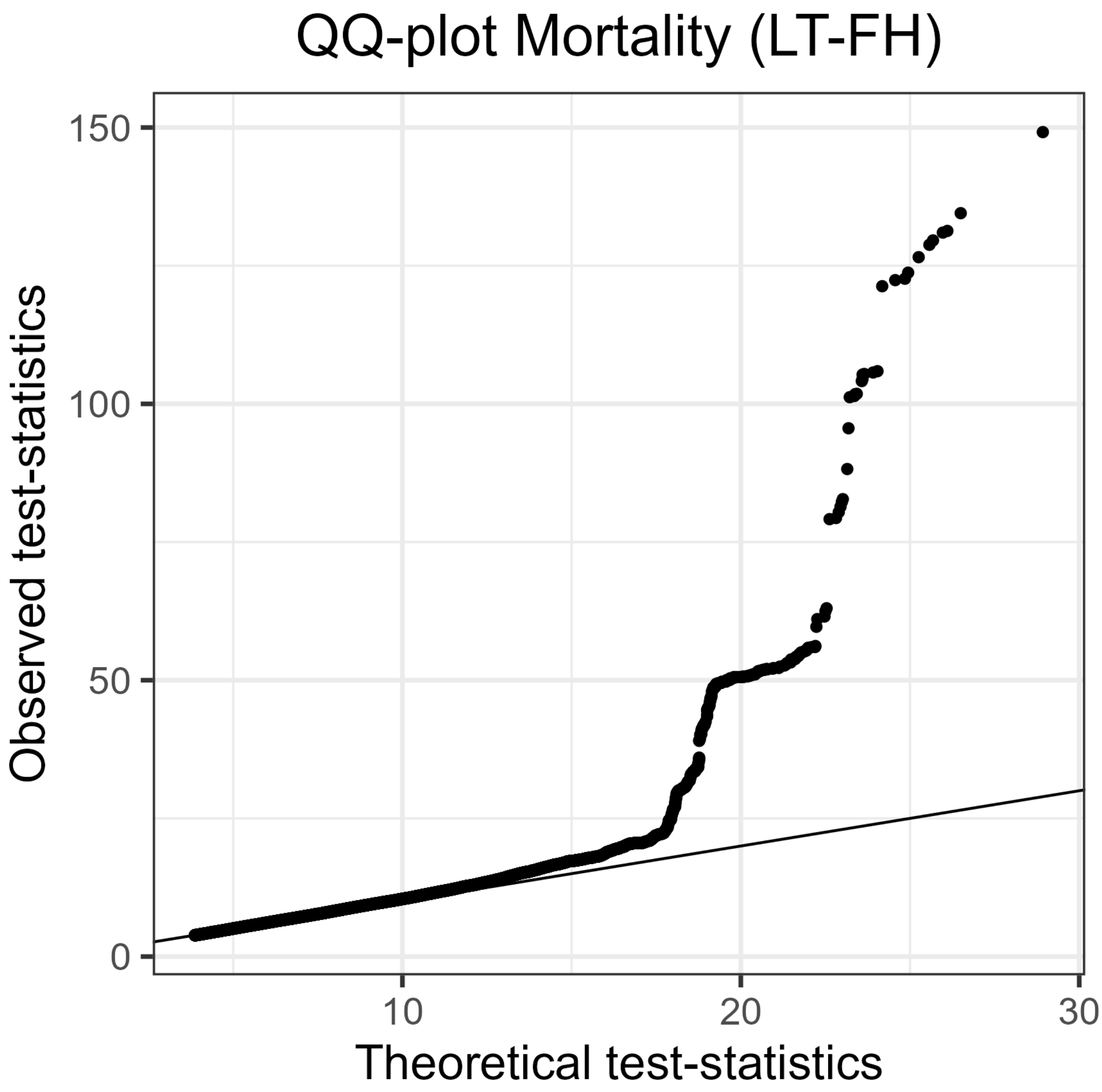

Figure S25: QQ plot of Mortality for LT-FH. We excluded SNPs with p-values greater than 0.05.

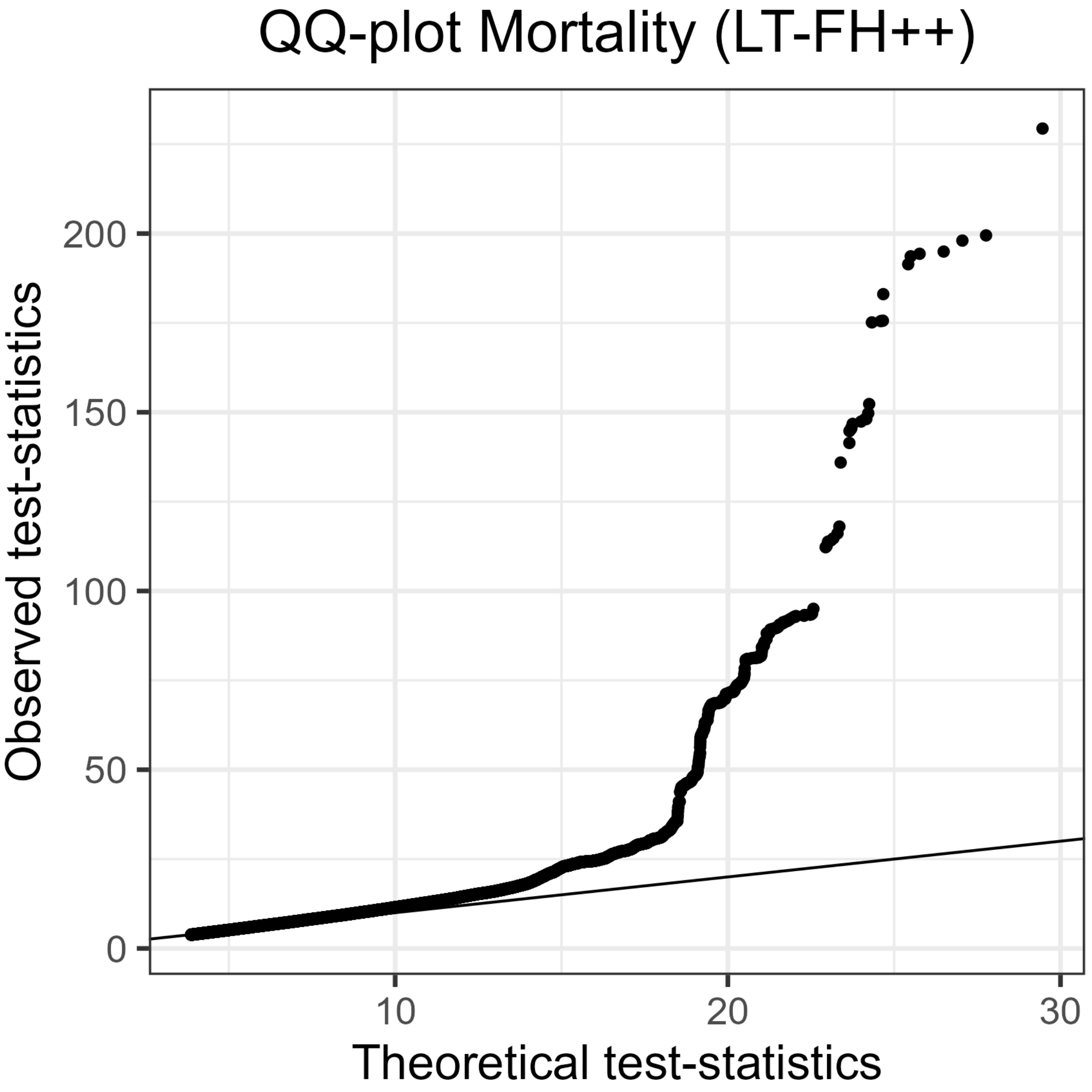

Figure S26: QQ plot of Mortality for LT-FH++. We excluded SNPs with p-values greater than 0.05.

iPSYCH Results

This part of the supplementary notes contains plots associated with the analysis of the iPSYCH, in particular about ADHD, ASD, DEP, and SCZ. The results appear in this order.

Attention Deficit Hyperactivity Disorder

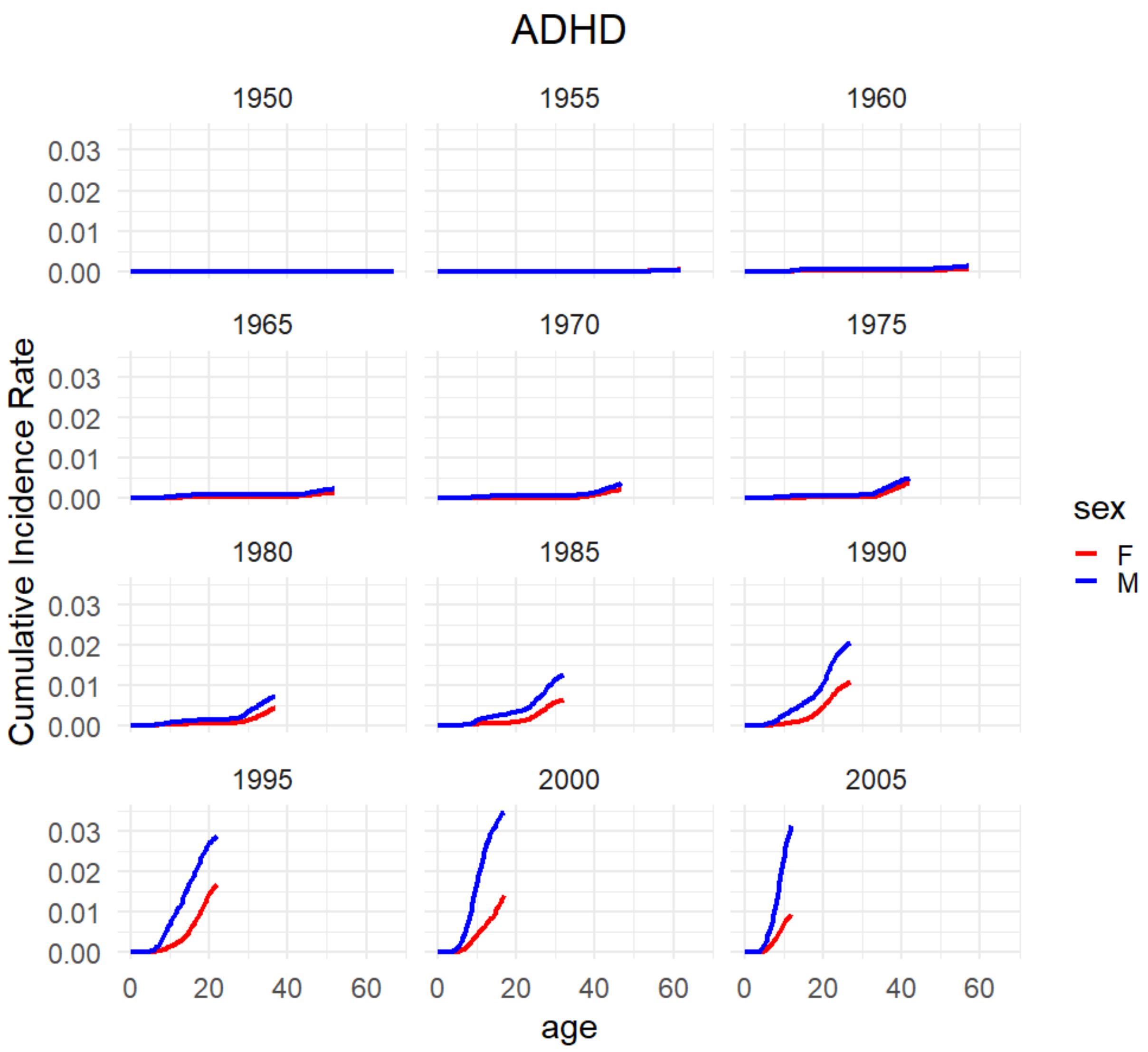

Figure S27: Plot of the cumulative incidence rate for Attention Deficit Hyperactivity Disorder grouped by birth year in the Danish registers. The red line corresponds to females and the blue corresponds to males.

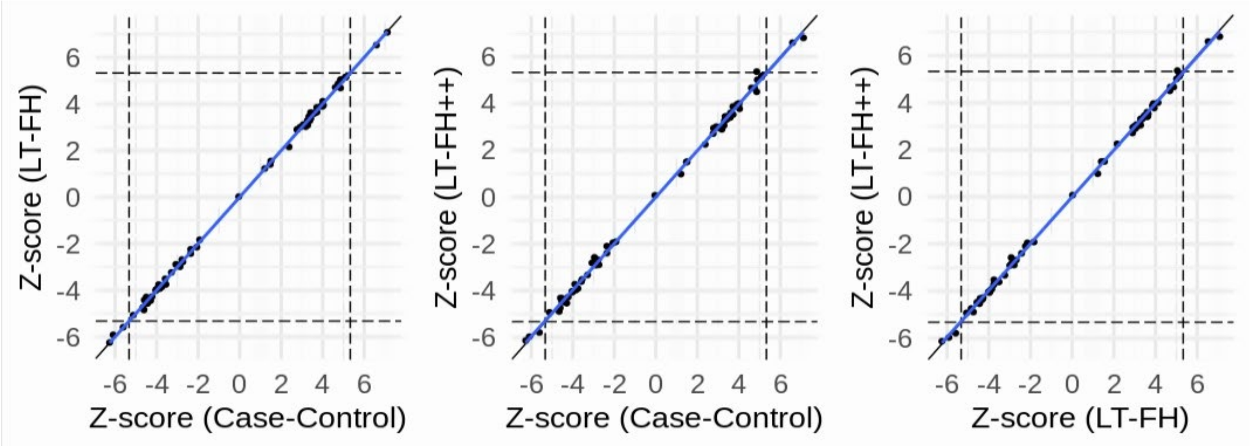

Figure S28: The Z-scores for ADHD for the three outcomes plotted against each other. The dots correspond to LD clumped SNPs that are genome-wide significant in the largest published meta-analysis and present in the iPSYCH cohort (see Methods for details). The blue line indicates the linear regression line between two outcomes and a black line indicates the identity line. The slopes of the regression lines are not significantly different from 1 for any pair of outcomes.

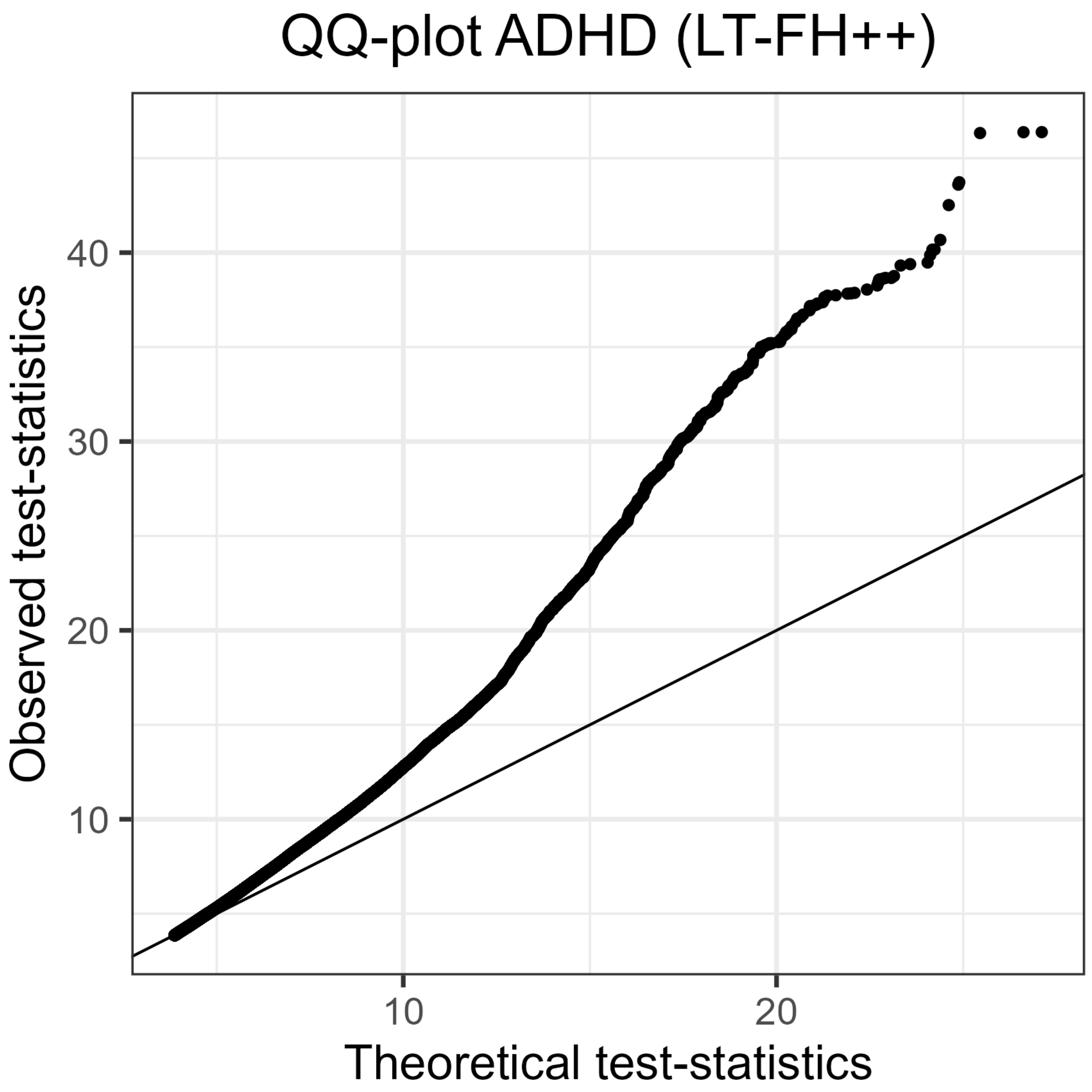

Figure S29: QQ plot of ADHD for LT-FH++. We excluded SNPs with p-values greater than 0.05.

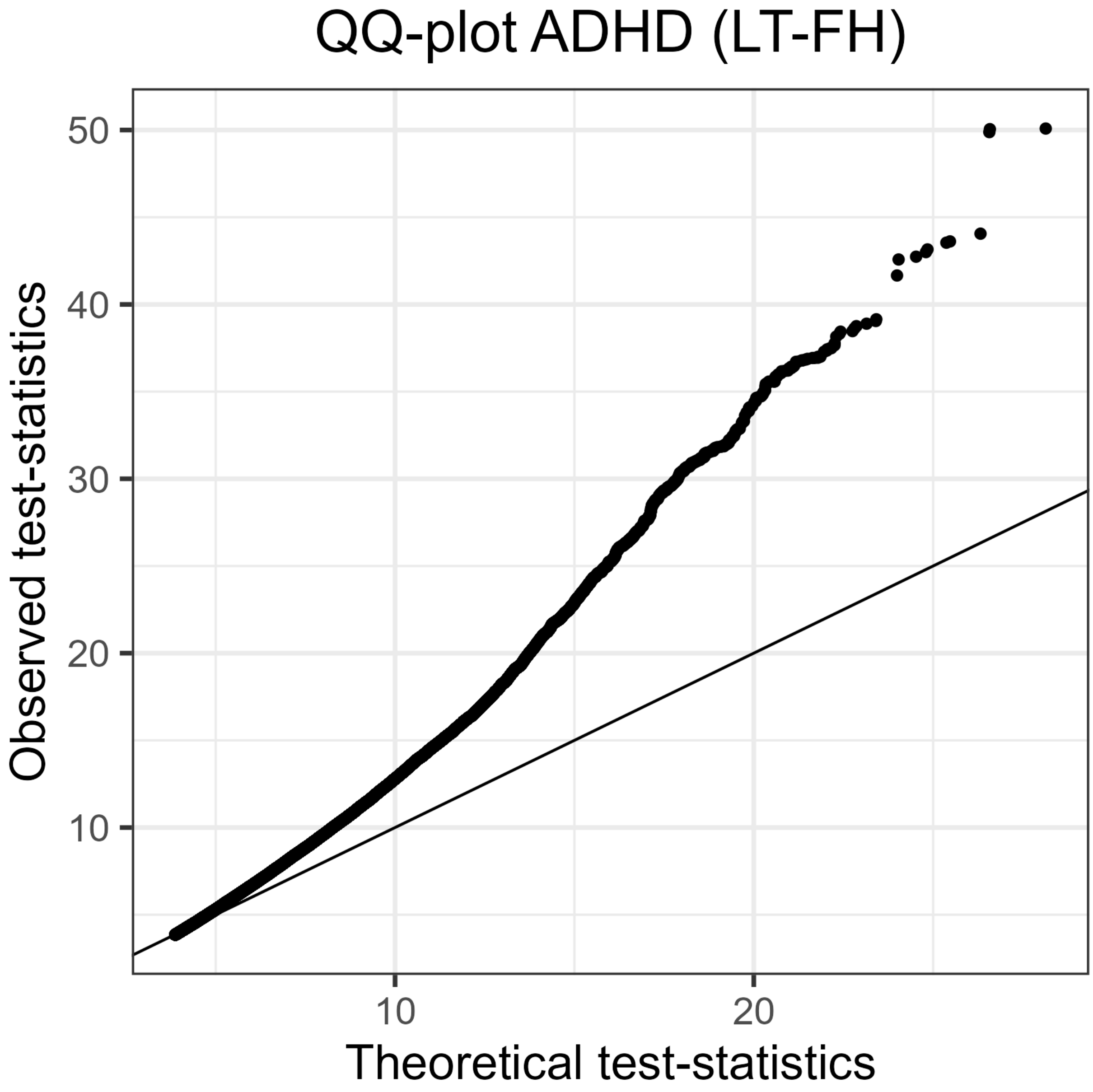

Figure S30: QQ plot of ADHD for LT-FH. We excluded SNPs with p-values greater than 0.05.

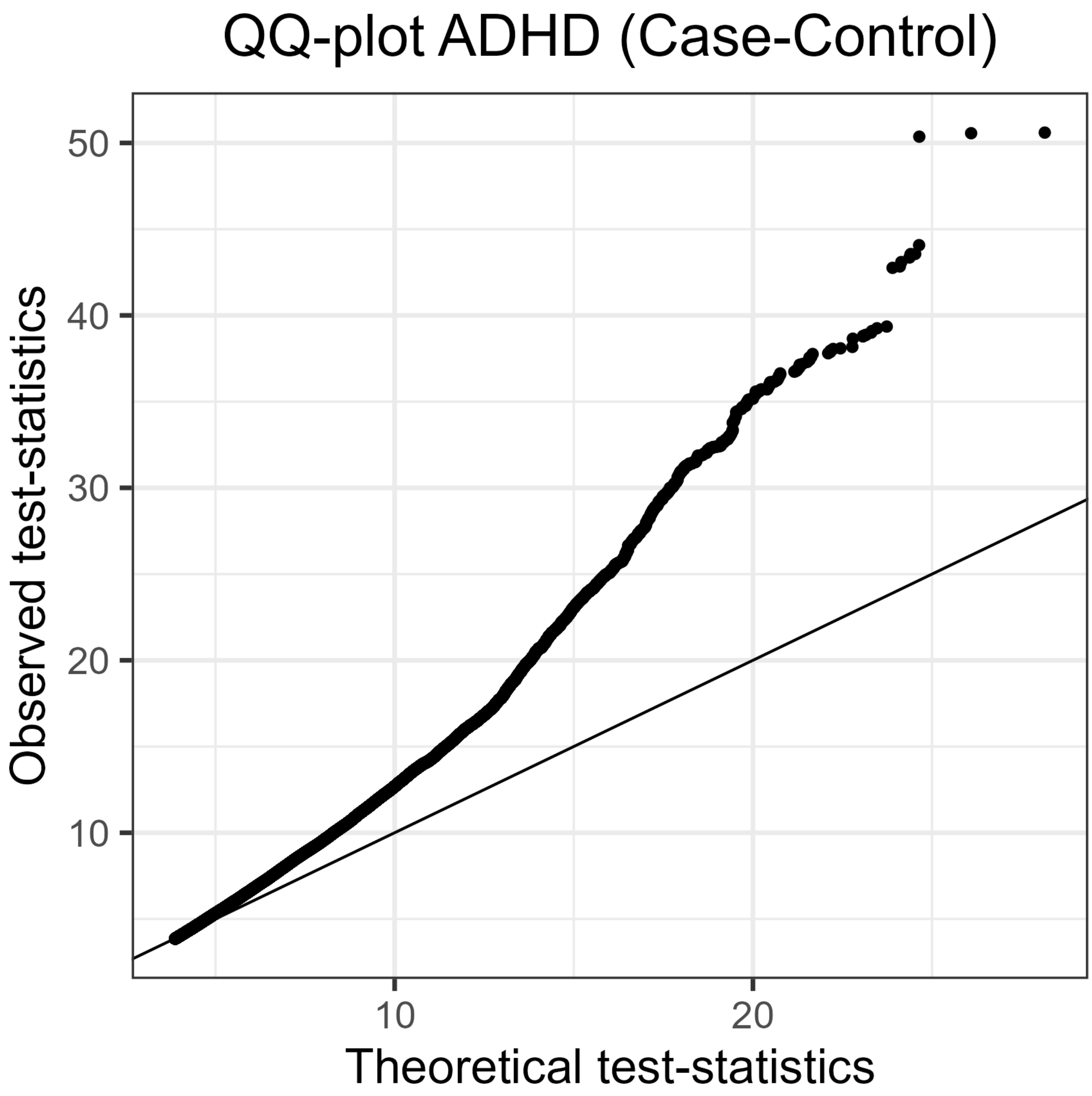

Figure S31: QQ plot of ADHD for case-control status. We excluded SNPs with p-values greater than 0.05.

Autism Spectrum Disorder

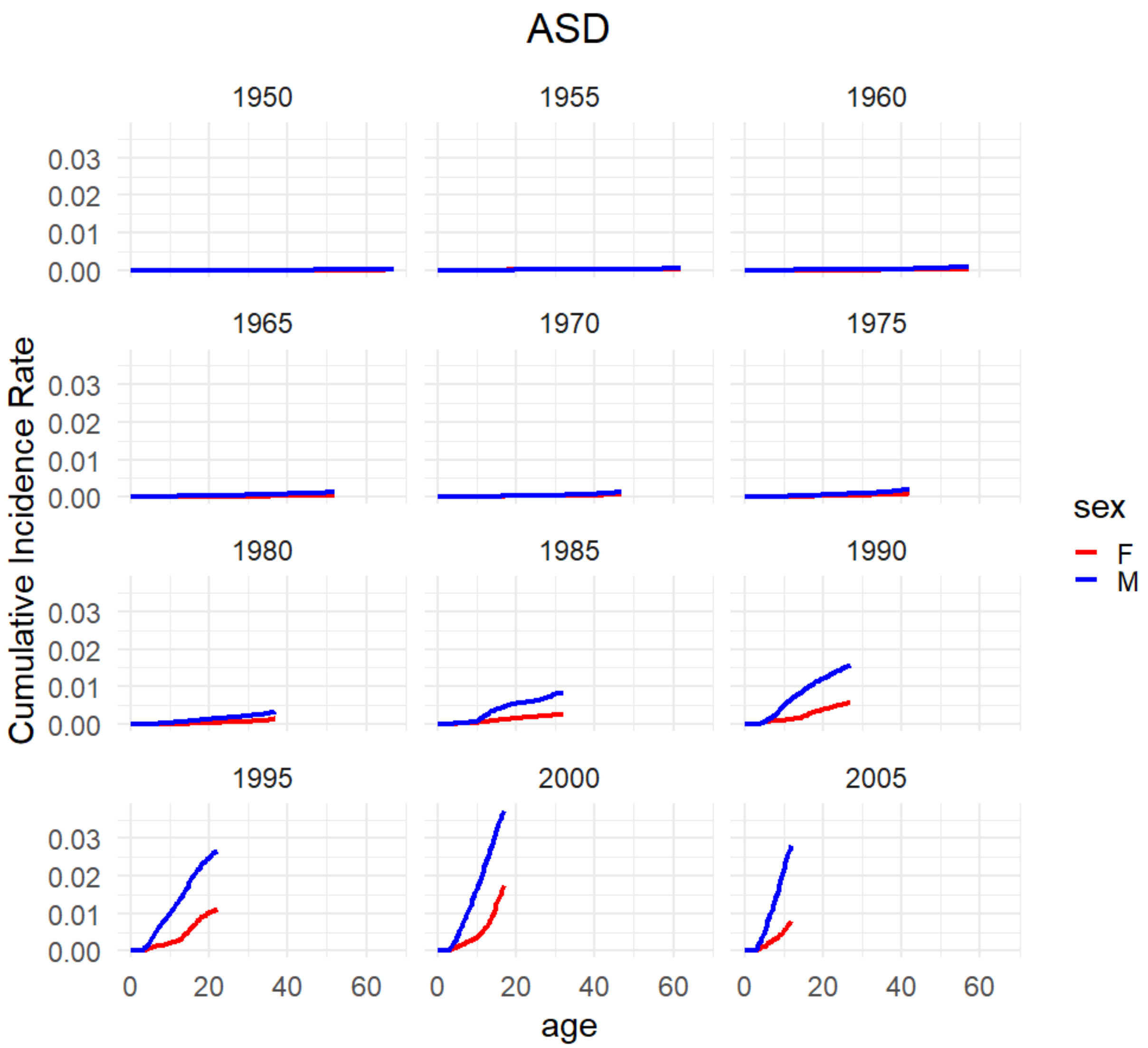

Figure S32: Plot of the cumulative incidence rate for autism spectrum disorder grouped by birth year in the Danish registers. The red line corresponds to females and the blue corresponds to males.

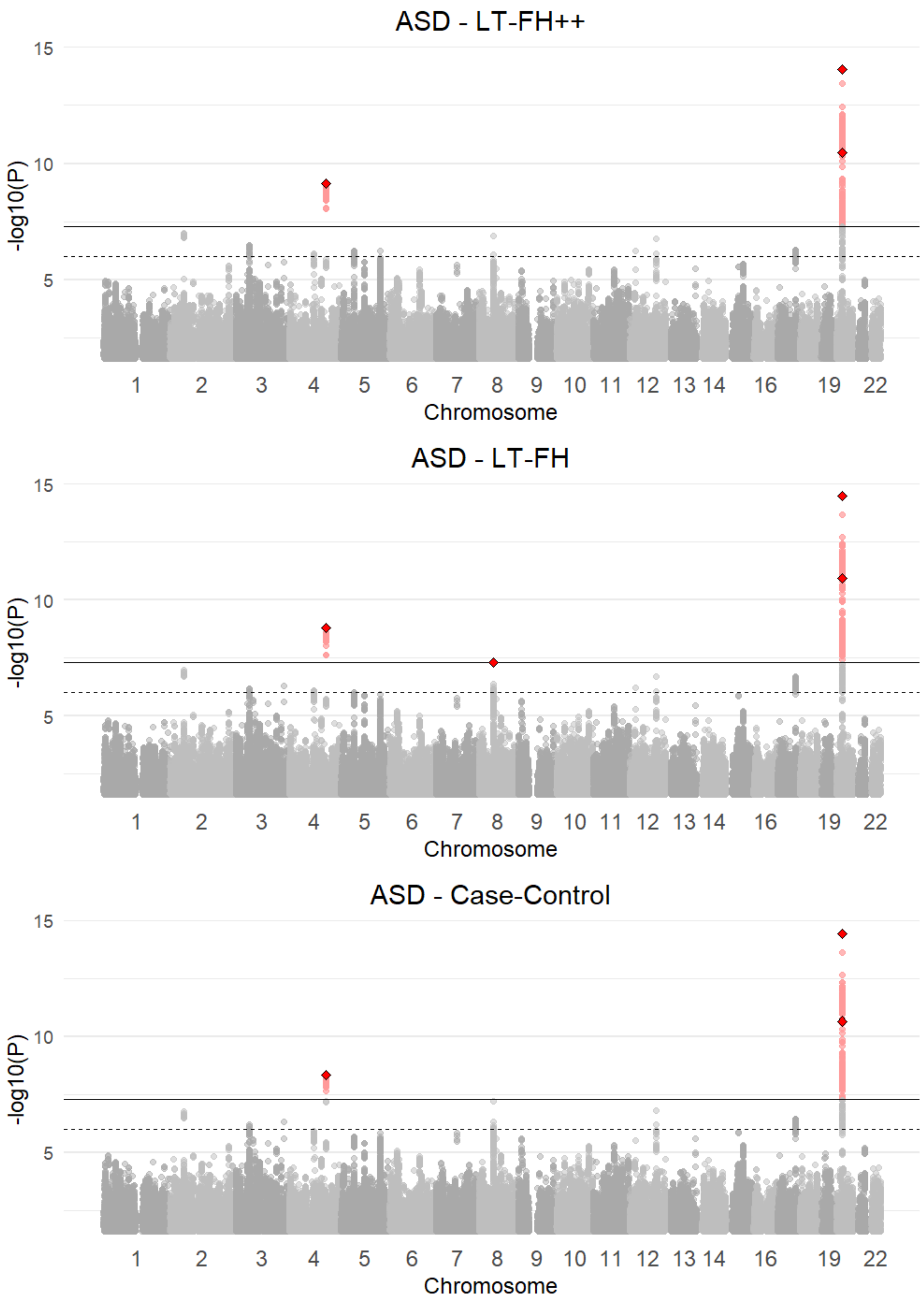
ASD - LT-FH++
15
10
5
-log10(P)
1 2 3 4 5 6 7 8 9 10 11 12 13 14 16 19 22
Chromosome
ASD - LT-FH
15
10
5
-log10(P)
1 2 3 4 5 6 7 8 9 10 11 12 13 14 16 19 22
Chromosome
ASD - Case-Control
15
10
5
-log10(P)
1 2 3 4 5 6 7 8 9 10 11 12 13 14 16 19 22
Chromosome

Figure S33: Manhattan plots for LT-FH++, LT-FH, and case-control GWAS of autism spectrum disorder (ASD) in the iPSYCH cohort. The Manhattan plots display a Bonferroni corrected significance level of 5×10^{-8}, and a suggestive threshold of 5×10^{-6}. The genome-wide significant SNPs are colored in red. The diamonds correspond to top SNPs in a window of size 300k base pairs.

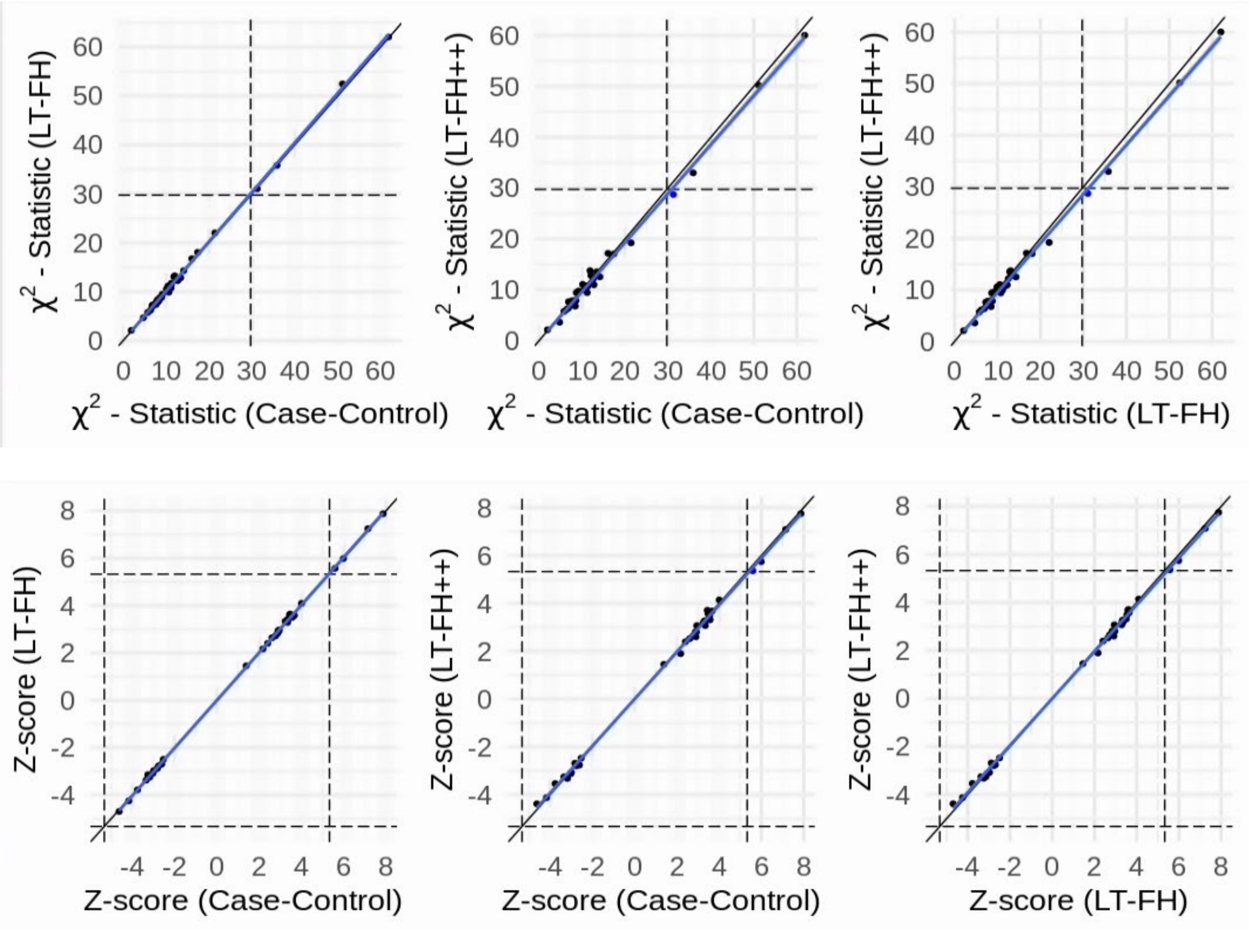

Figure S34: The Z-scores and χ^2 statistics for ASD for the three outcomes plotted against each other. The dots correspond to LD clumped SNPs that are genome-wide significant in the largest published meta-analysis and present in the iPSYCH cohort (see Methods for details). The blue line indicates the linear regression line between two outcomes and a black line indicates the identity line. The slopes of the regression lines are not significantly different from 1 for any pair of outcomes.

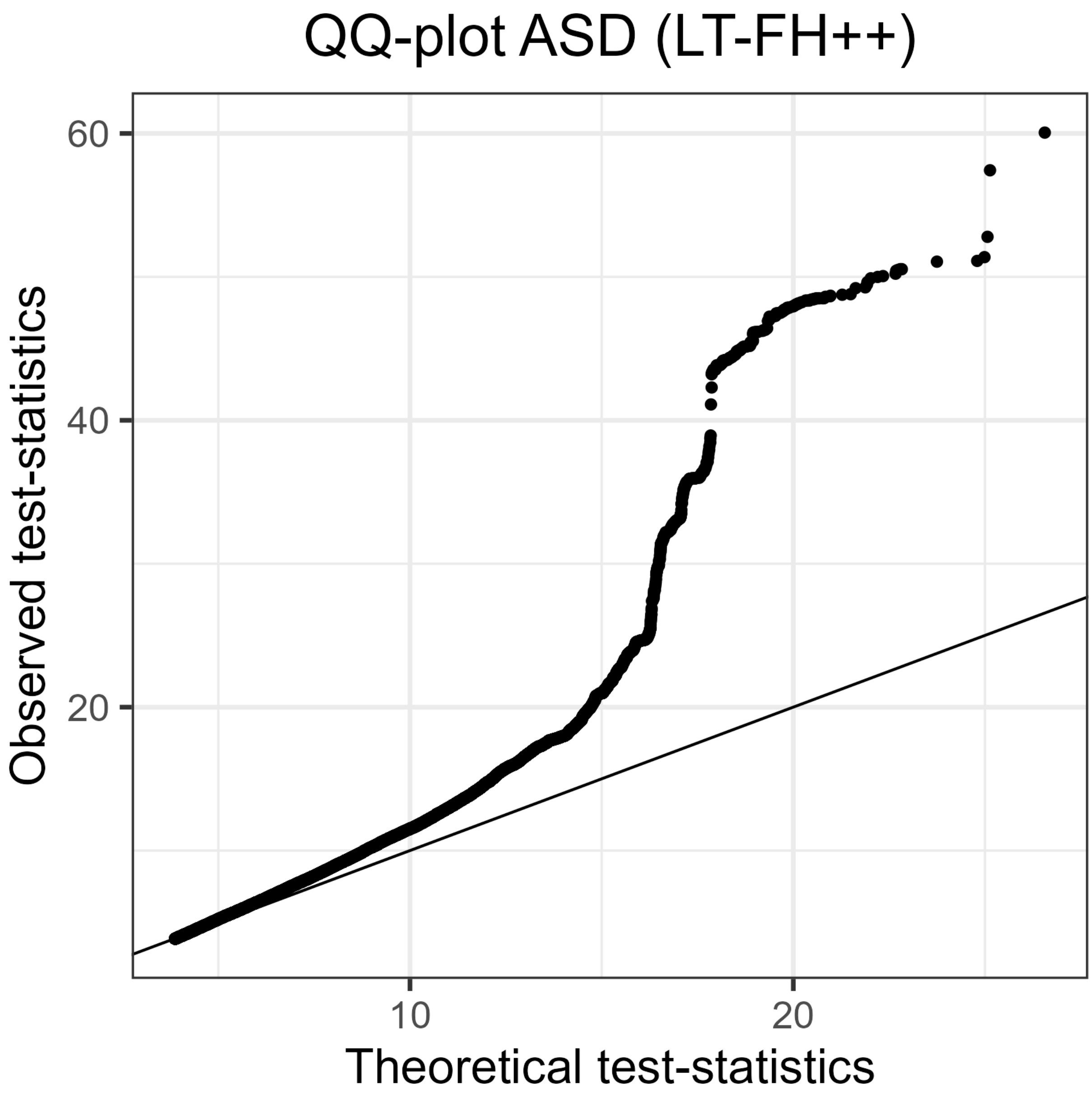

Figure S35: QQ plot of ASD for LT-FH++. We excluded SNPs with p-values greater than 0.05.

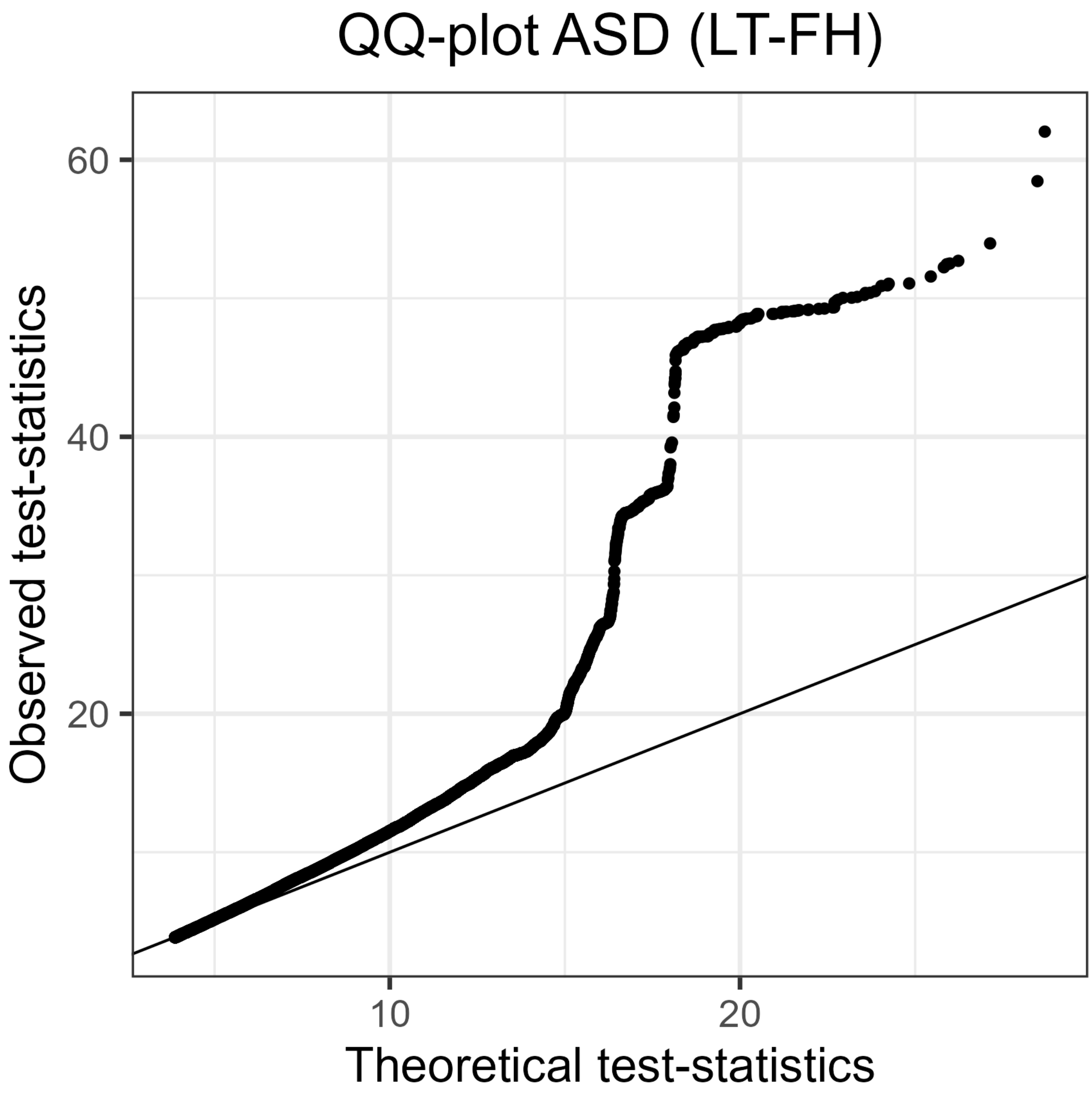

Figure S36: QQ plot of ASD for LT-FH. We excluded SNPs with p-values greater than 0.05.

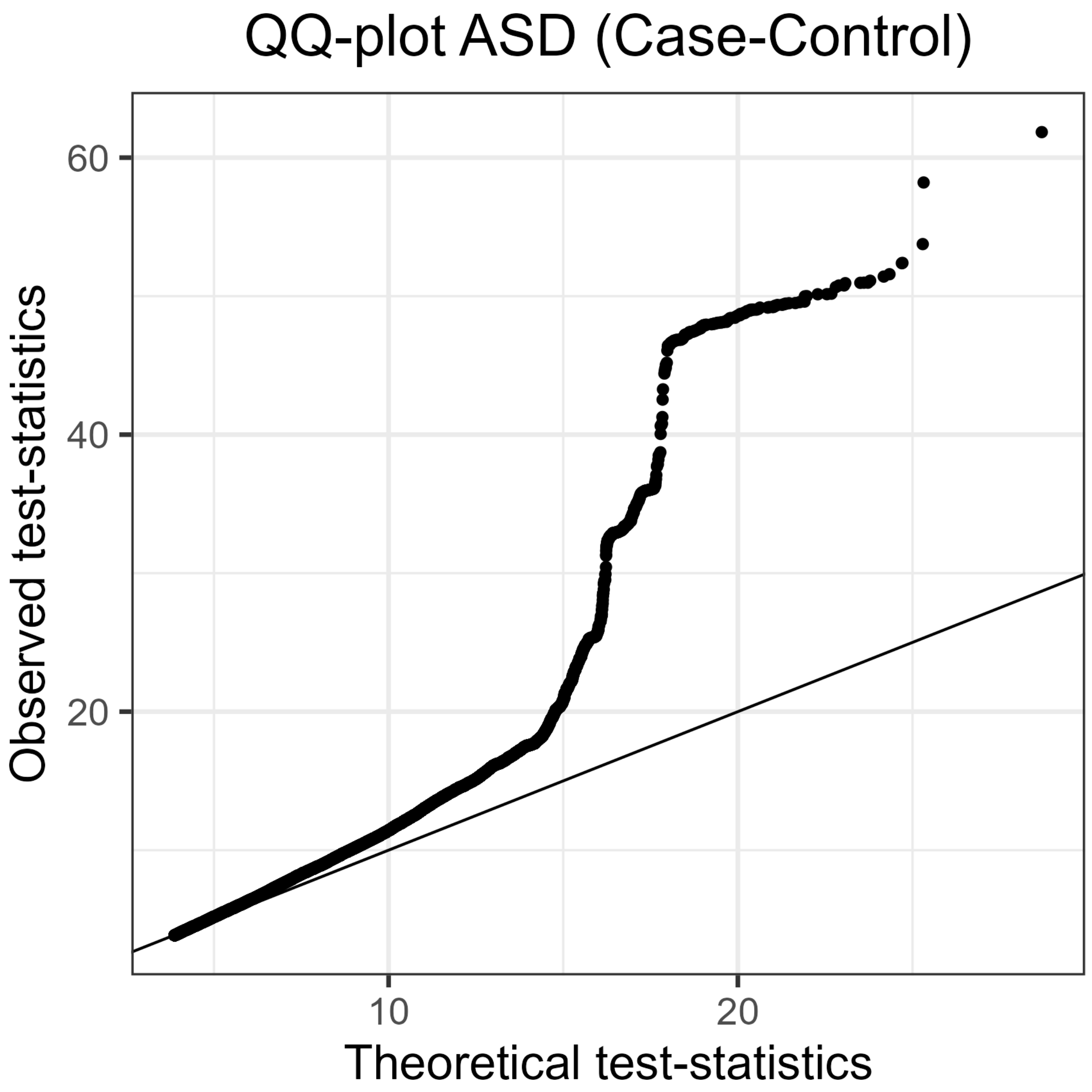

Figure S37: QQ plot of ASD for case-control status. We excluded SNPs with p-values greater than 0.05.

Depression

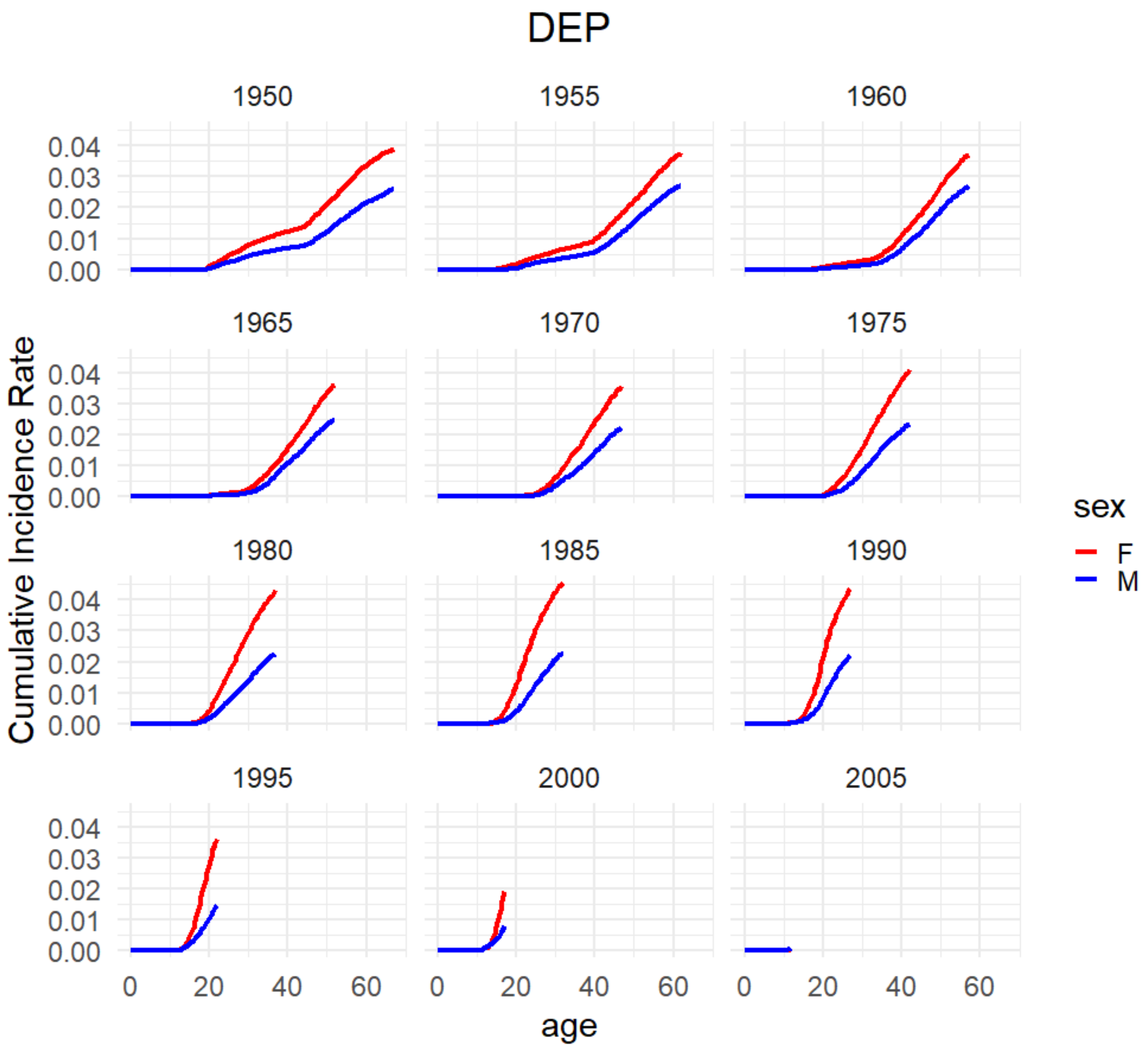

Figure S38: Plot of the cumulative incidence rate for depression grouped by birth year in the Danish registers. The red line corresponds to females and the blue corresponds to males.

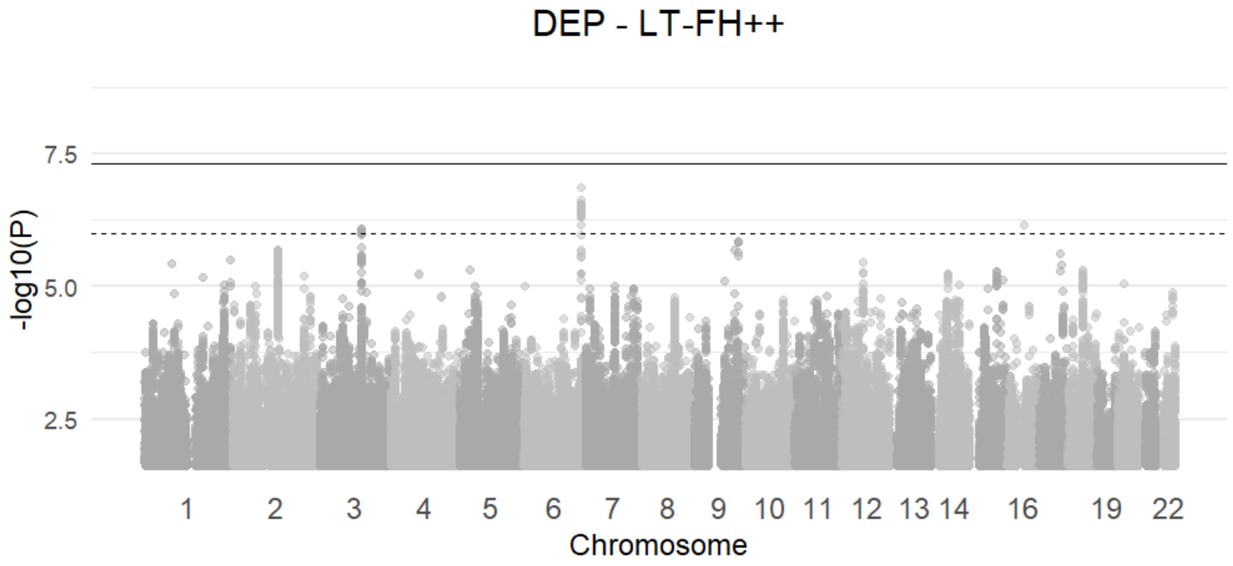
DEP - LT-FH++
7.5
5.0
2.5
-log10(P)
1 2 3 4 5 6 7 8 9 10 11 12 13 14 16 19 22
Chromosome

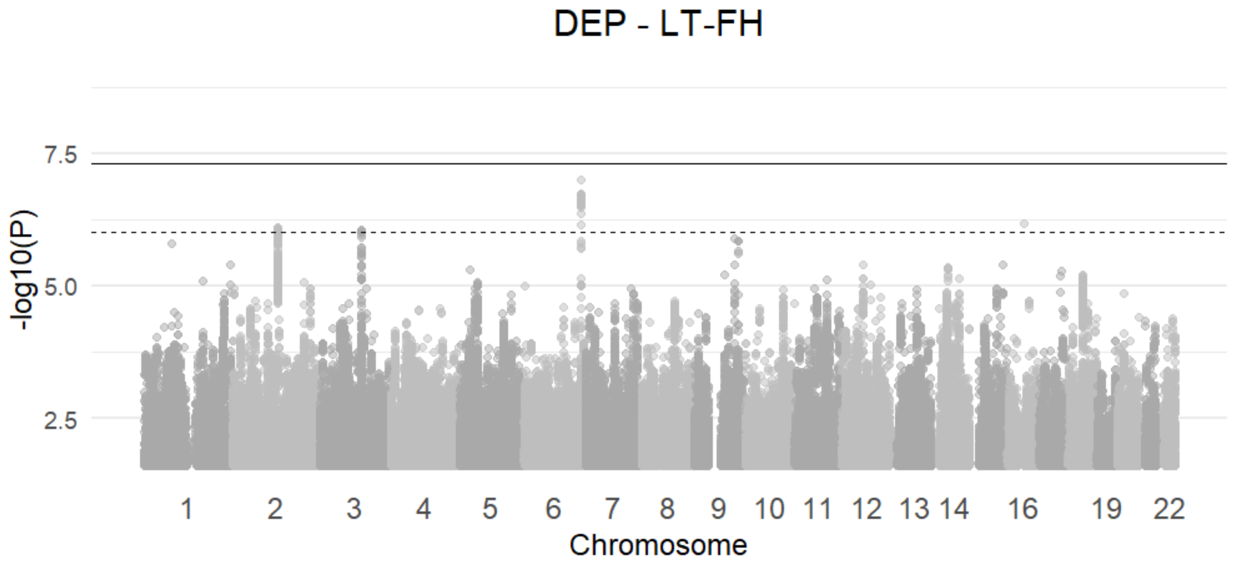
DEP - LT-FH
7.5
5.0
2.5
-log10(P)
1 2 3 4 5 6 7 8 9 10 11 12 13 14 16 19 22
Chromosome

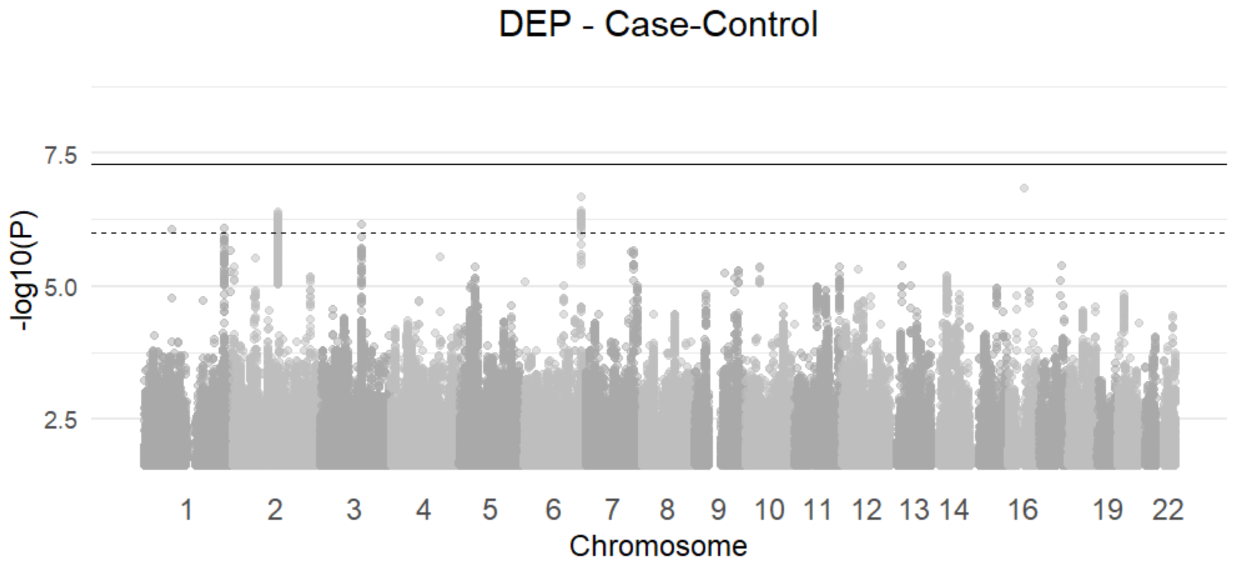
DEP - Case-Control
7.5
5.0
2.5
-log10(P)
1 2 3 4 5 6 7 8 9 10 11 12 13 14 16 19 22
Chromosome

Figure S39: Manhattan plots for LT-FH++, LT-FH, and case-control GWAS of depression in the iPSYCH cohort. The Manhattan plots display a Bonferroni corrected significance level of 5×10^{-8}, and a suggestive threshold of 5×10^{-6}. The genome-wide significant SNPs are colored in red. The diamonds correspond to top SNPs in a window of size 300k base pairs.

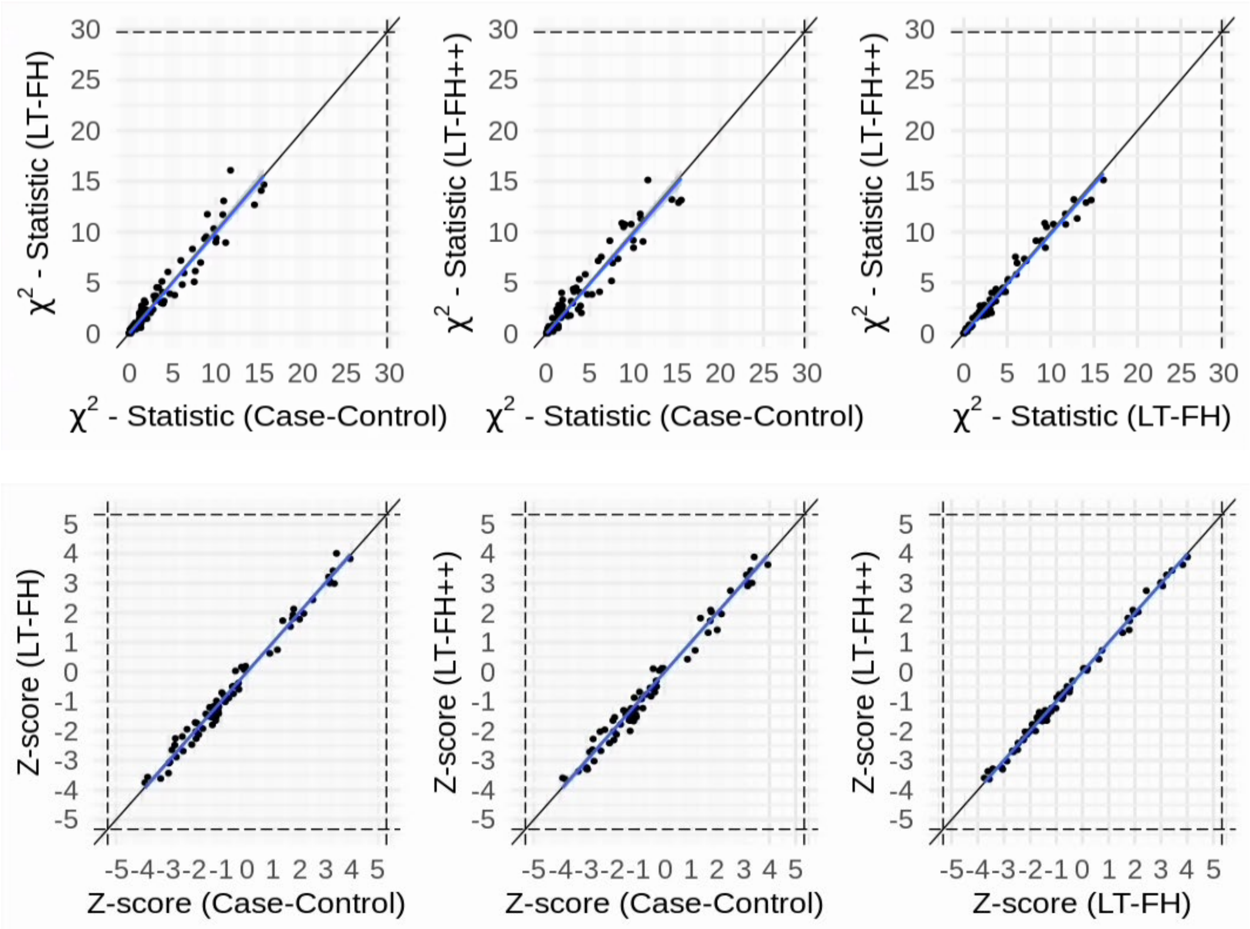

Figure S40: The Z-scores and χ^2 statistics for depression for the three outcomes plotted against each other. The dots correspond to LD clumped SNPs that are genome-wide significant in the largest published meta-analysis and present in the iPSYCH cohort (see Methods for details). The blue line indicates the linear regression line between two outcomes and a black line indicates the identity line. The slopes of the regression lines are not significantly different from 1 for any pair of outcomes.

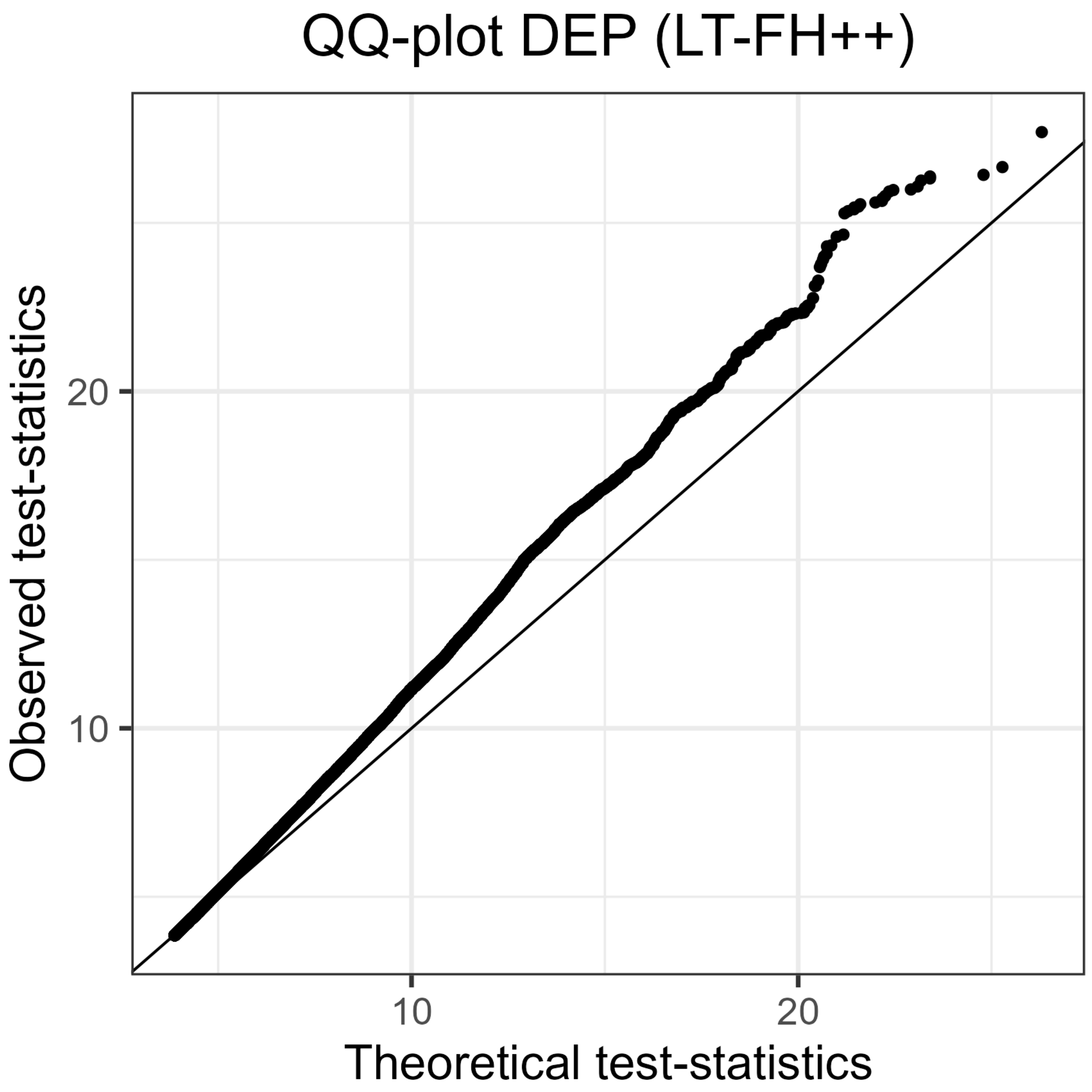

Figure S41: QQ plot of DEP for LT-FH++. We excluded SNPs with p-values greater than 0.05.

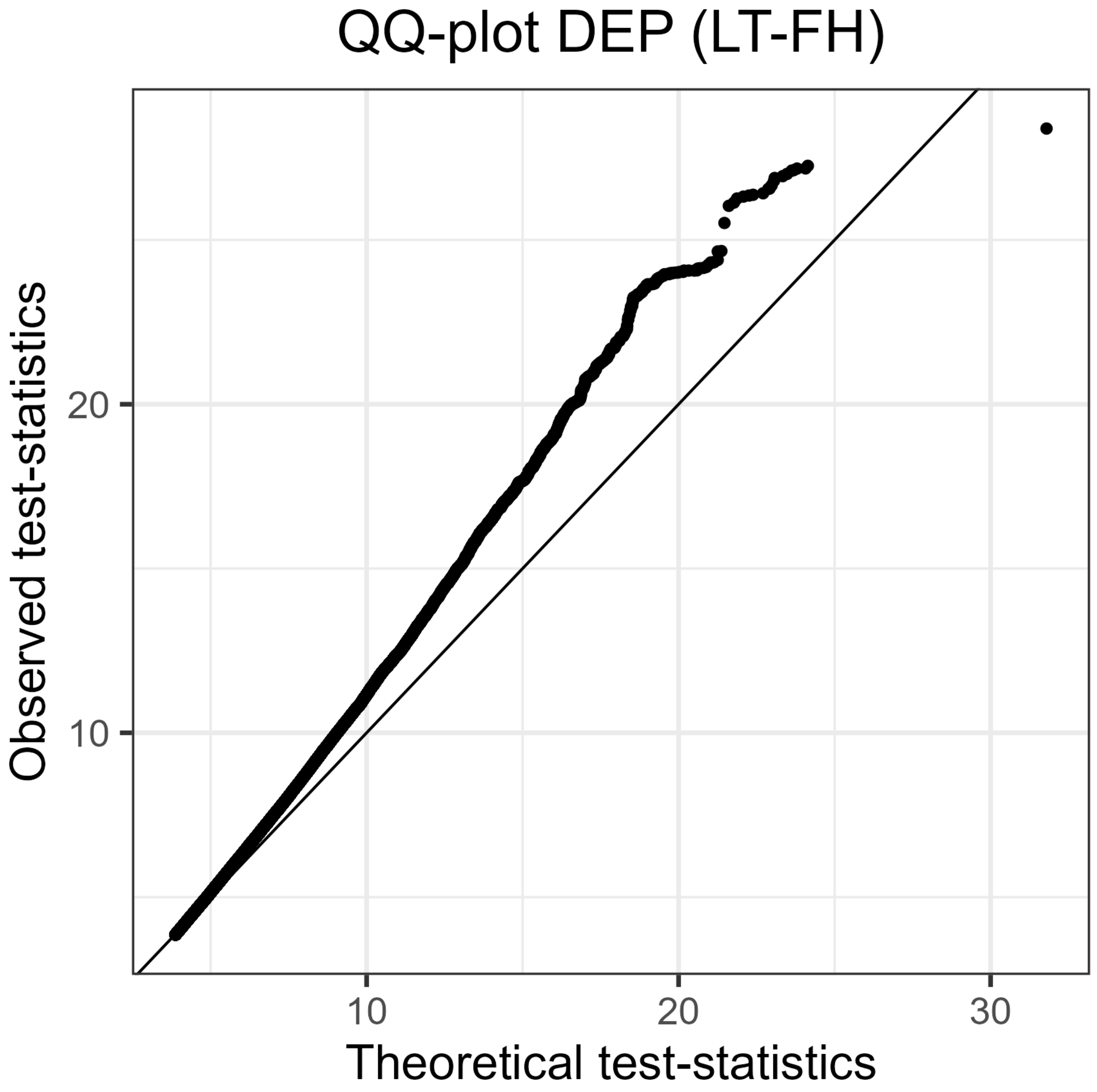

Figure S42: QQ plot of DEP for LT-FH. We excluded SNPs with p-values greater than 0.05.

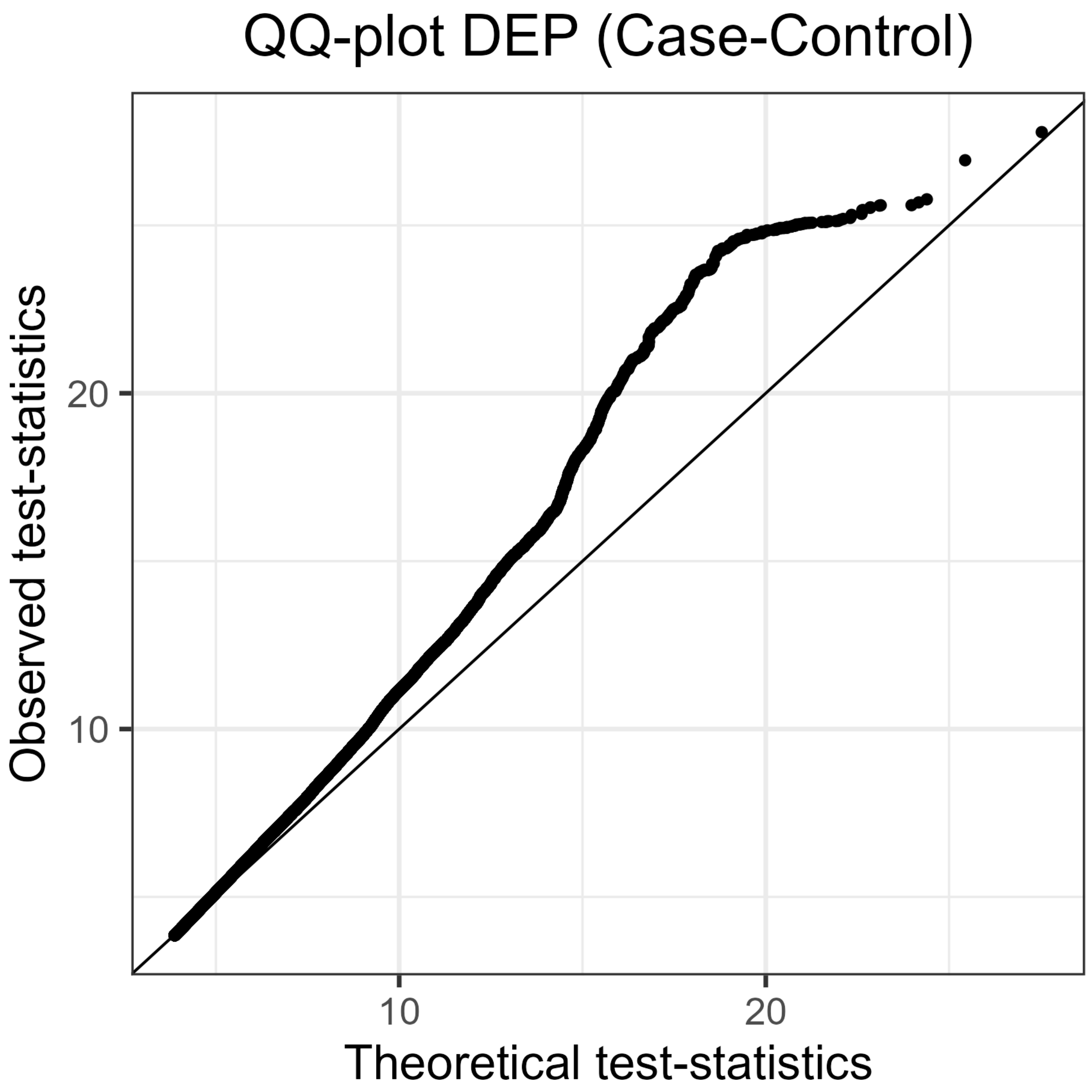

Figure S43: QQ plot of DEP for case-control status. We excluded SNPs with p-values greater than 0.05.

Schizophrenia

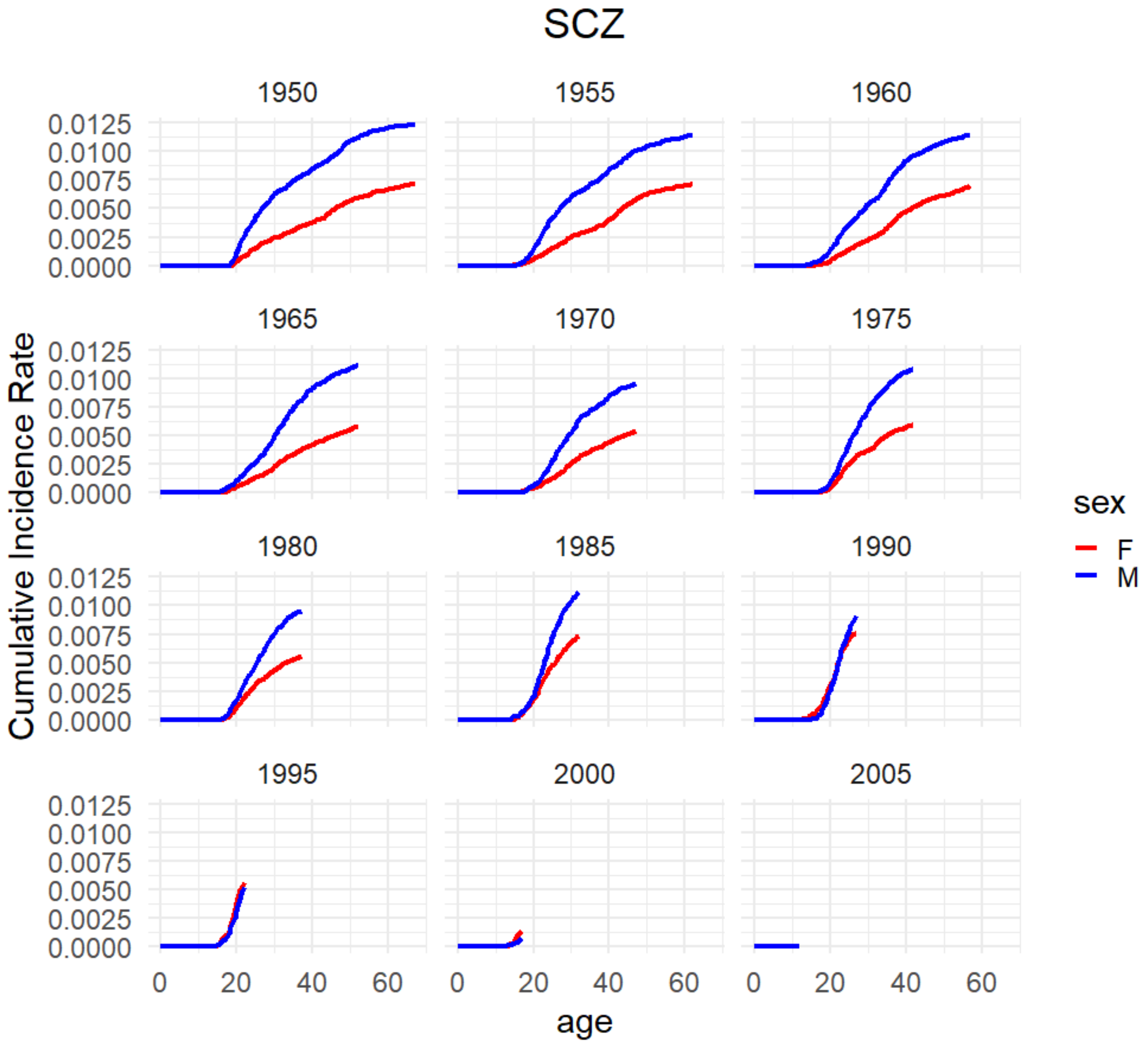

Figure S44: Plot of the cumulative incidence rate for schizophrenia grouped by birth year in the Danish registers. The red line corresponds to females and the blue corresponds to males.

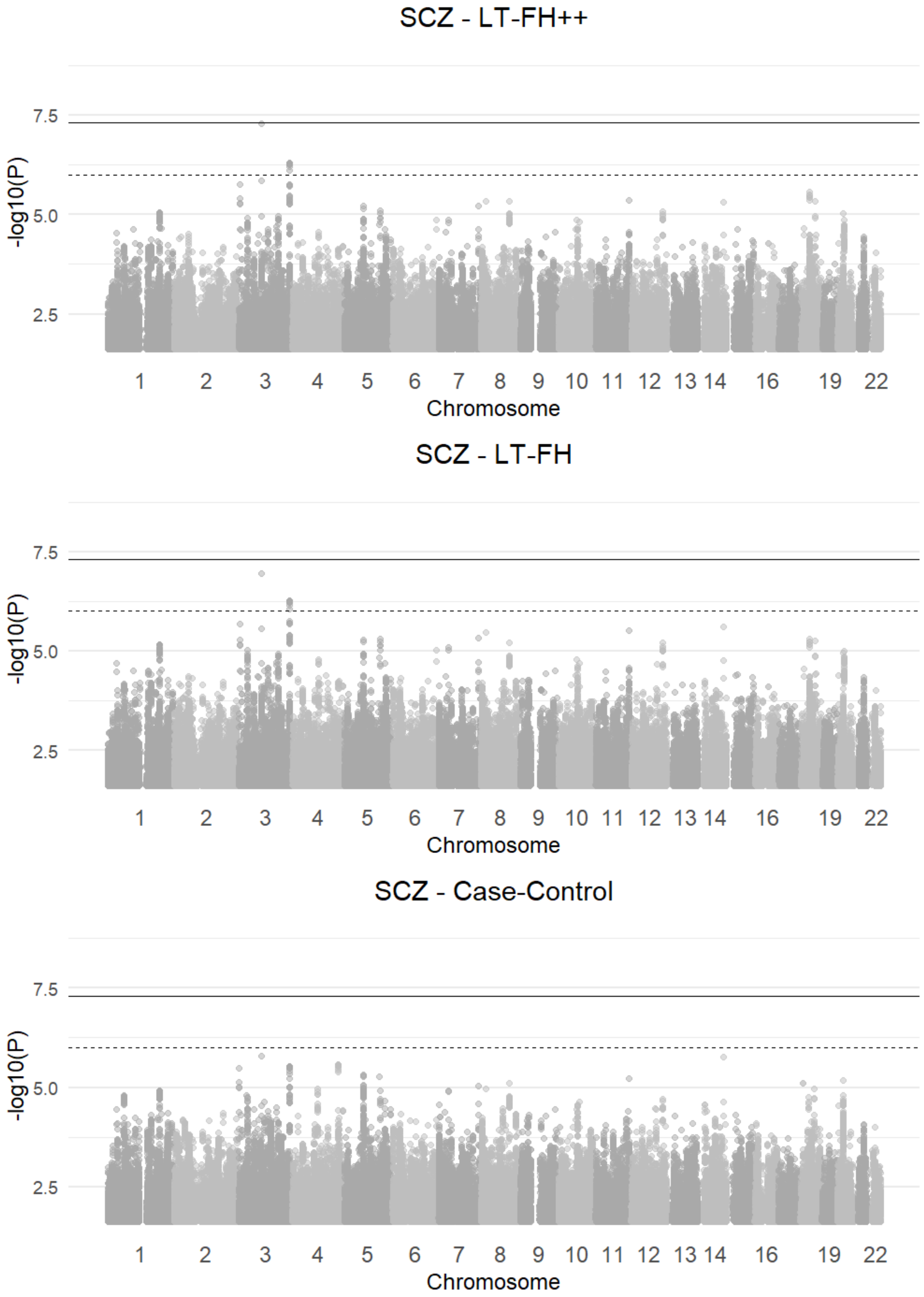
SCZ - LT-FH++
-log10(P)
7.5
5.0
2.5
1 2 3 4 5 6 7 8 9 10 11 12 13 14 16 19 22
Chromosome
SCZ - LT-FH
-log10(P)
7.5
5.0
2.5
1 2 3 4 5 6 7 8 9 10 11 12 13 14 16 19 22
Chromosome
SCZ - Case-Control
-log10(P)
7.5
5.0
2.5
1 2 3 4 5 6 7 8 9 10 11 12 13 14 16 19 22
Chromosome

Figure S45: Manhattan plots for LT-FH++, LT-FH, and case-control GWAS of schizophrenia in the iPSYCH cohort. The Manhattan plots display a Bonferroni corrected significance level of 5×10^{-8}, and a suggestive threshold of 5×10^{-6}. The genome-wide significant SNPs are colored in red. The diamonds correspond to top SNPs in a window of size 300k base pairs.

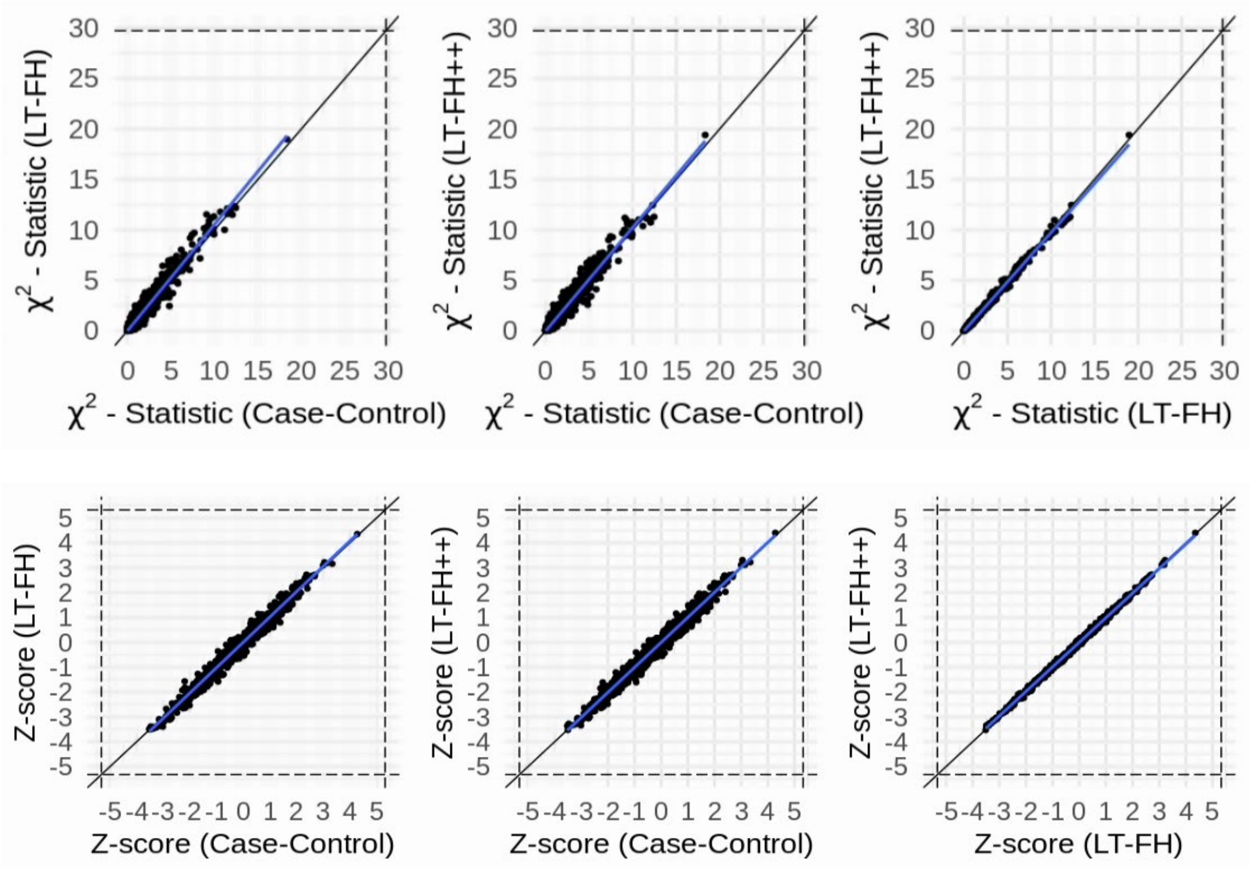

Figure S46: The Z-scores and χ^2 statistics for schizophrenia for the three outcomes plotted against each other. The dots correspond to LD clumped SNPs that are genome-wide significant in the largest published meta-analysis and present in the iPSYCH cohort (see Methods for details). The blue line indicates the linear regression line between two outcomes and a black line indicates the identity line. The slopes of the regression lines are not significantly different from 1 for any pair of outcomes.

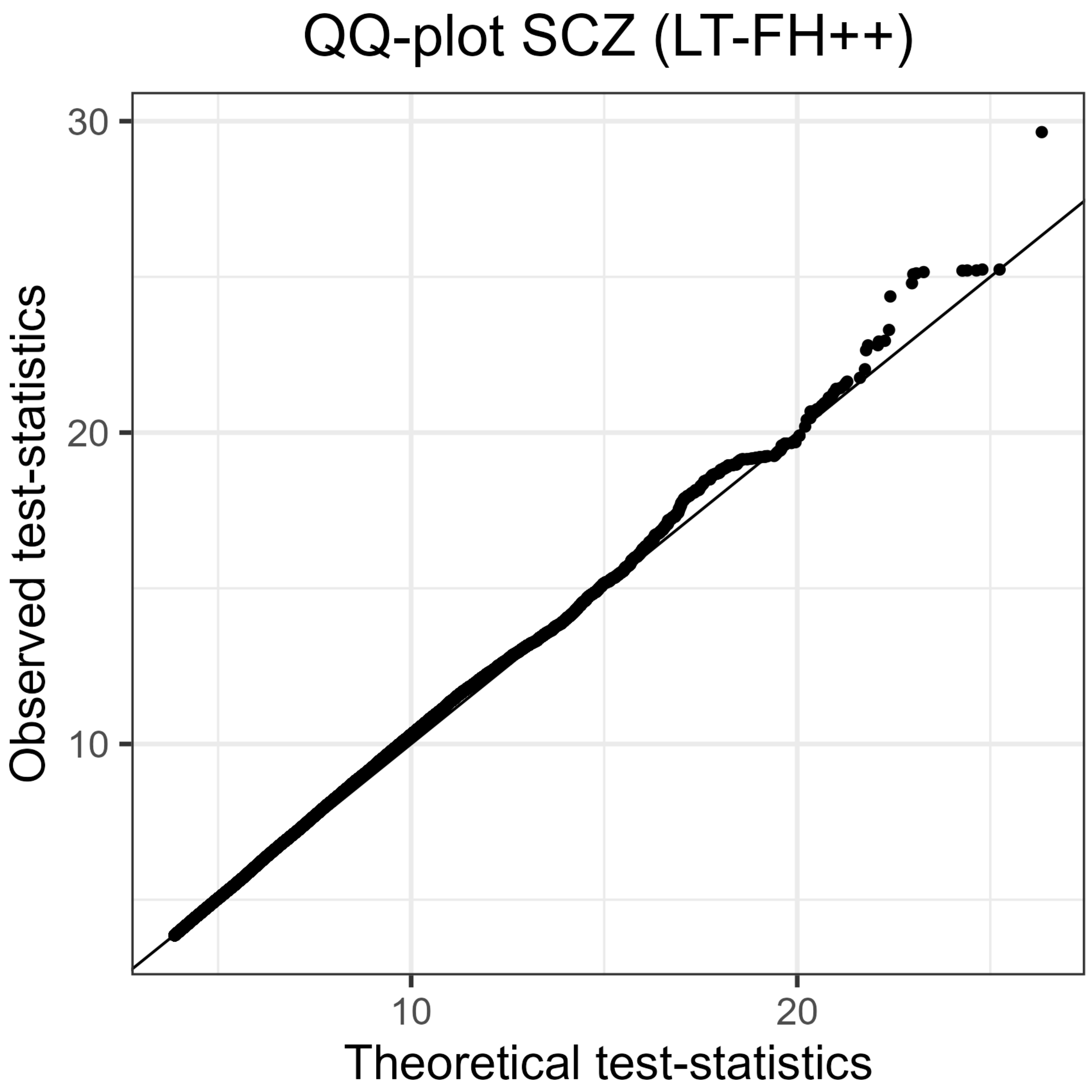

Figure S47: QQ plot of SCZ LT-FH++. We excluded SNPs with p-values greater than 0.05.

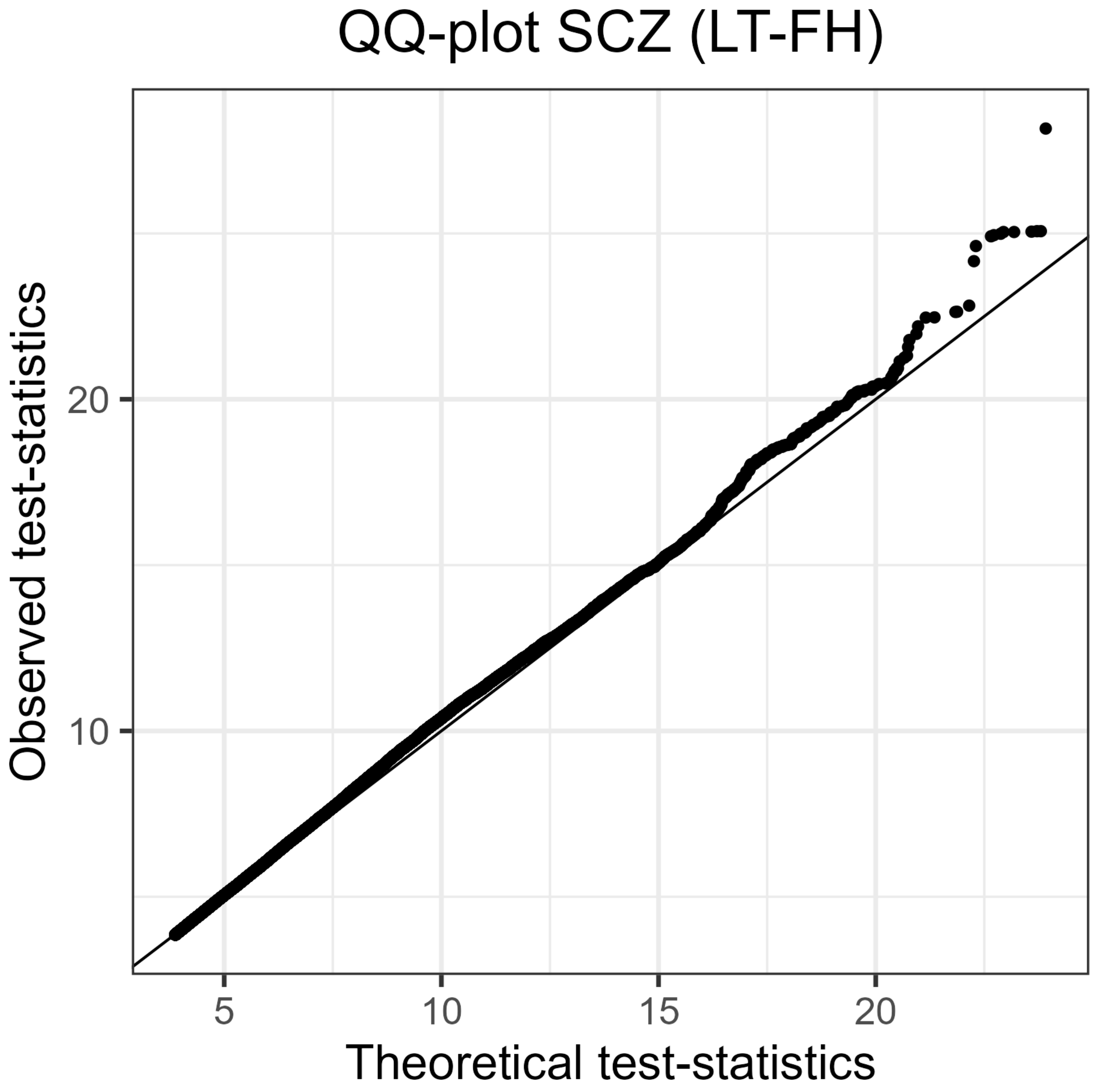

Figure S48: QQ plot of SCZ for LT-FH. We excluded SNPs with p-values greater than 0.05.

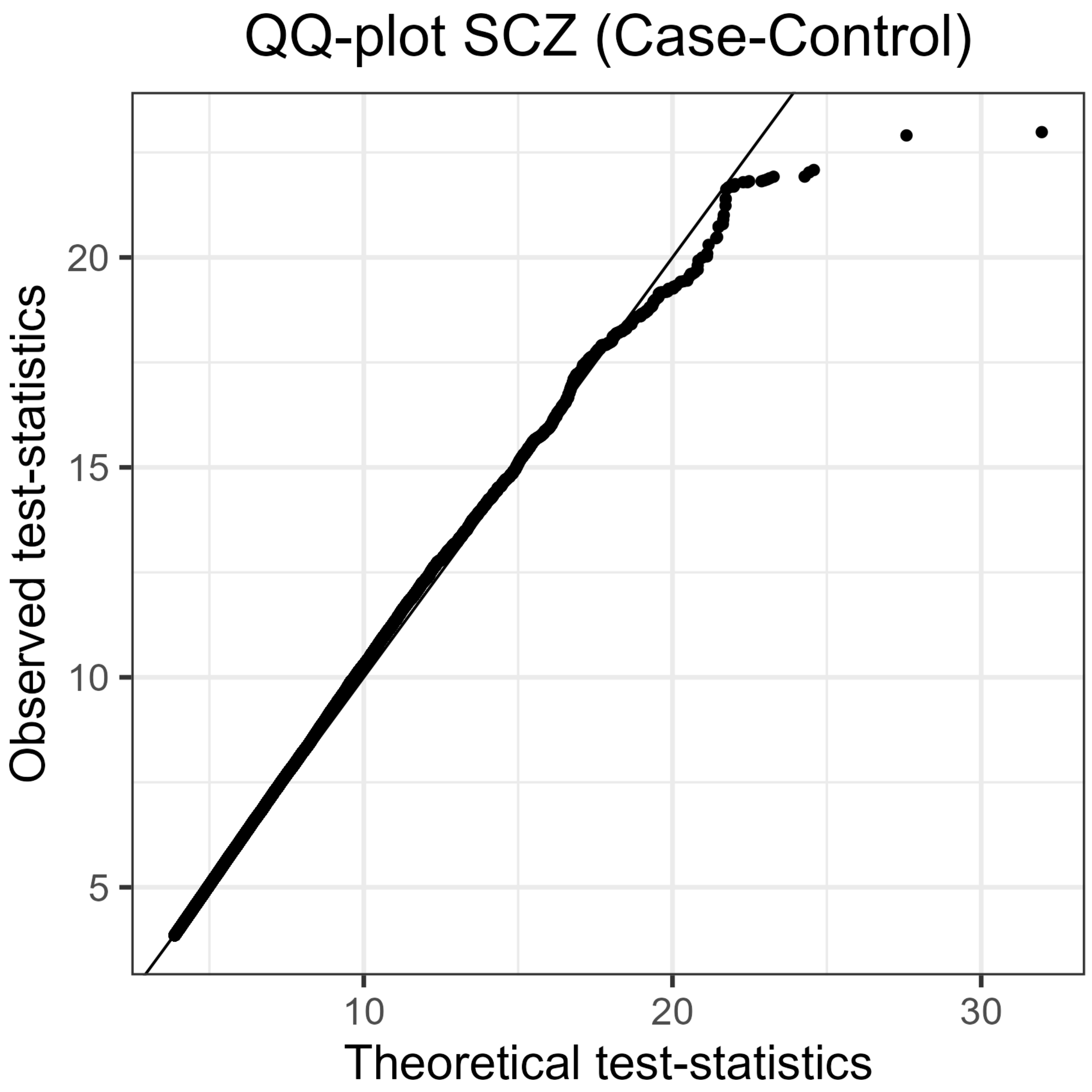

Figure S49: QQ plot of SCZ for case-control status. We excluded SNPs with p-values greater than 0.05.

Time-to-event model

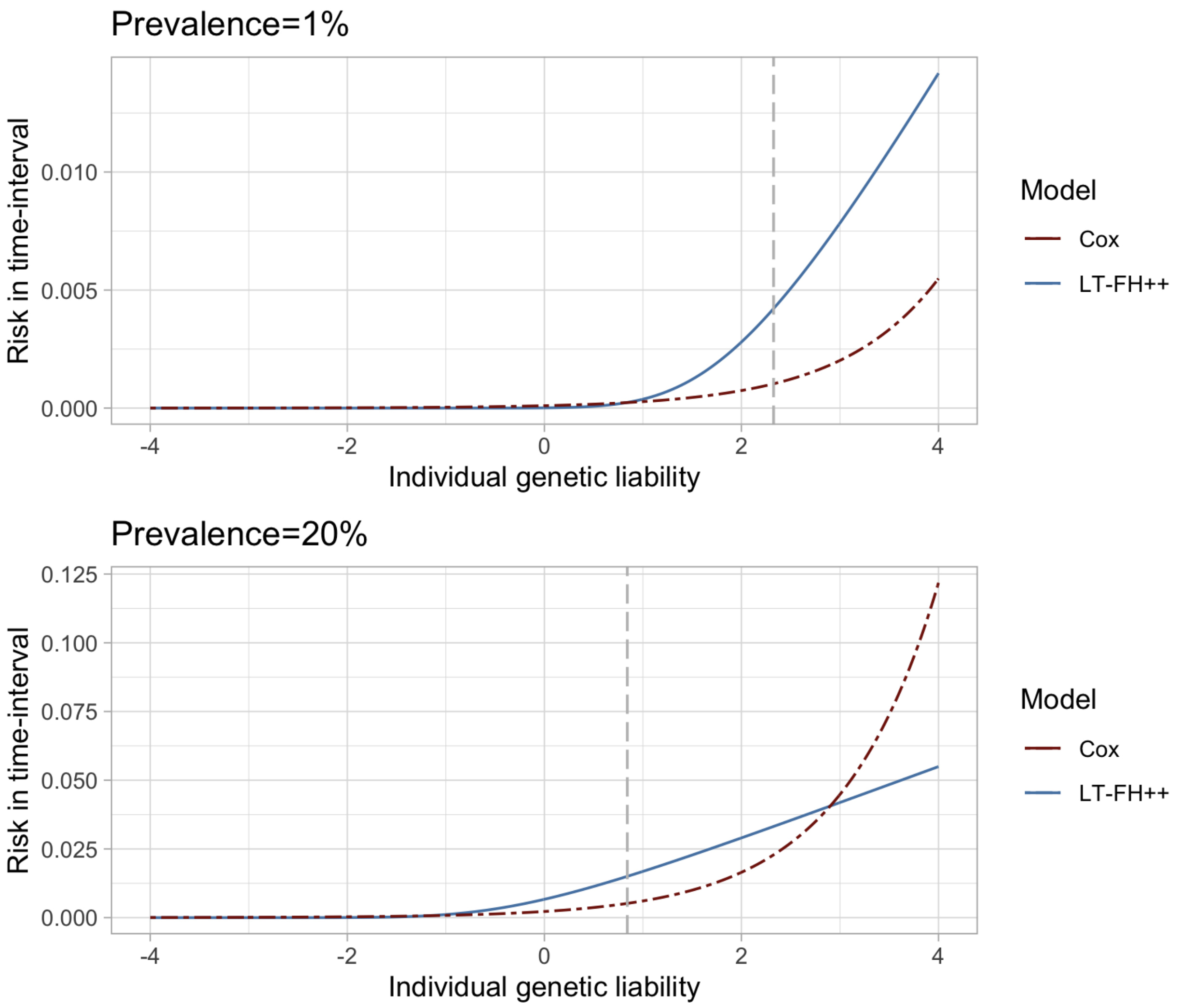

Figure S50: Risk (probability) for becoming a case within a time-interval corresponding to 1% relative increase in prevalence as a function of the genetic liability. The total prevalence changes from 1% and 20% to 1.01% and 20.2% respectively. For Cox regression we assume a constant base incidence rate, corresponding to the prevalence. The vertical dotted grey line denotes the liability threshold corresponding to the prevalence. We note that the risk for becoming a case within a small time-interval is proportional to the hazard rate.

Supplemental Tables

In this section we will include supplementary tables. We have split the tables into results from the simulations and results from the real-world analysis.

Simulation Results

Power & chi-square statistics

downsampling	Method	Power	Power sd	Mean causal chisq	Mean causal chisq sd	Mean null chisq	Mean null chisq sd
No	GWAS	0.1032	0.00711493	11.0968799	0.43115084	0.99897719	0.00578427
No	GWAX	0.1231	0.00546606	12.5786011	0.4423601	0.99990076	0.00449278
No	LT-FH	0.1594	0.0090701	15.0321414	0.55502615	0.99931755	0.00428873
No	LT-FH++	0.1659	0.00769488	15.4442868	0.55315674	0.9999296	0.00423999
Yes	GWAS	0.0315	0.00538	6.39052221	0.14904845	0.99977481	0.00449538

Yes	GWAX	0.0326	0.00474225	6.29082337	0.16595288	0.99942523	0.00382606
Yes	LT-FH	0.0376	0.00636309	6.74523086	0.16838193	0.99971582	0.00422892
Yes	LT-FH++	0.0436	0.00638053	7.18764144	0.18546156	0.99969847	0.00427345

Table S1: Table containing simulation results for the default simulation setup with a prevalence of 5%.

downsampling	Method	Power	Power sd	Mean causal chisq	Mean causal chisq sd	Mean null chisq	Mean null chisq sd
No	GWAS	0.1722	0.01050714	15.8921563	0.53905794	0.99877984	0.00315812
No	GWAX	0.1825	0.0134433	16.7631072	0.48298124	1.0003692	0.00452109
No	LT-FH	0.2335	0.01162612	21.2427511	0.65085209	0.9991315	0.00352166
No	LT-FH++	0.2444	0.01220382	22.1870996	0.64715594	1.00009105	0.00312604
Yes	GWAS	0.0752	0.00657943	9.33170612	0.17869466	0.99945213	0.00251433
Yes	GWAX	0.0702	0.00694102	8.88419642	0.17602146	1.00085604	0.00278447
Yes	LT-FH	0.0929	0.00597123	10.3183013	0.18243892	1.00009436	0.00268586
Yes	LT-FH++	0.1086	0.00471876	11.4003675	0.21725906	0.99952079	0.00294178

Table S2: Table containing simulation results for the default simulation setup with a prevalence of 10%.

Method	Mean causal chisq	Mean causal chisq sd	Power	Power sd	Mean null chisq	Mean null chisq sd
GWAS	31.2035947	3.257629924	0.3285	0.013938356	1.002499879	0.005953325
GWAX	38.88355922	3.743794854	0.3791	0.00807534	1.000536754	0.005106242
LT-FH	45.22722295	4.623442789	0.4145	0.009046178	1.001252174	0.003933444
LT-FH++	47.0349021	4.831365713	0.4227	0.008857514	1.00116158	0.004565413

Table S3: Table containing the mean chi-square test statistic for the causal and null snps, as well as the power. The table contains these values for N = 300,000, 5% prevalence, no downsampling, and full family history and age-of-onset information.The other parameter setups can be found in the supplementary data, and include 2 different prevalences, 4 different values of N, 4 different levels of completeness of family history and age-of-onset information.

Method	Mean causal chisq	Mean causal chisq sd	Power	Power sd	Mean null chisq	Mean null chisq sd
GWAS	45.69678239	4.422692924	0.4195	0.012385027	1.000089338	0.008232819
GWAX	52.84787089	4.169667408	0.4549	0.013461468	0.999932281	0.006525725
LT-FH	64.87177976	5.687542538	0.4989	0.015701734	1.000850998	0.008694631
LT-FH++	69.00093357	6.287391807	0.5095	0.013826303	1.001138582	0.007489548

Table S4: Table containing the mean chi-square test statistic for the causal and null snps, as well as the power. The table contains these values for N = 300,000, 10% prevalence, no downsampling, and full family history and age-of-onset information. The other parameter setups can be found in the supplementary data, and include 2 different prevalences, 4 different values of N, 4 different levels of completeness of family history and age-of-onset information.

False positive rates

Method	Alpha level	Proportion of False positives	Standard error
GWAS	0.000005	5.0505E-06	3.8671E-06
GWAS	0.00005	5.6566E-05	2.3664E-05
GWAS	0.0005	0.00053131	7.3058E-05
GWAS	0.005	0.0050303	0.0002248
GWAS	0.05	0.04988889	0.0006919
GWAX	0.000005	5.0505E-06	4.7546E-06
GWAX	0.00005	4.2424E-05	1.9987E-05
GWAX	0.0005	0.00048232	6.9577E-05
GWAX	0.005	0.00505202	0.00022527
GWAX	0.05	0.05031414	0.00069471

LT-FH	0.000005	6.0606E-06	5.4689E-06
LT-FH	0.00005	4.9495E-05	2.1687E-05
LT-FH	0.0005	0.00049697	7.0602E-05
LT-FH	0.005	0.00493434	0.00022266
LT-FH	0.05	0.04987929	0.00069187
LT-FH++	0.000005	5.0505E-06	4.4588E-06
LT-FH++	0.00005	5.2525E-05	2.1524E-05
LT-FH++	0.0005	0.00049545	7.0385E-05
LT-FH++	0.005	0.00495253	0.00022305
LT-FH++	0.05	0.04985859	0.00069173

Table S5: Table of the false positive rate at varying levels of significance thresholds in the default simulation setup with a prevalence of 5%.

Method	Alpha level	Proportion of False positives	Standard error
GWAS	0.000005	5.0505E-06	5.0505E-06
GWAS	0.00005	5.2525E-05	2.2498E-05
GWAS	0.0005	0.00046465	6.8385E-05
GWAS	0.005	0.00504646	0.00022517
GWAS	0.05	0.04989293	0.00069196
GWAX	0.000005	6.0606E-06	5.173E-06
GWAX	0.00005	5.7071E-05	2.3661E-05
GWAX	0.0005	0.00051111	7.1703E-05
GWAX	0.005	0.00503889	0.00022499
GWAX	0.05	0.0498697	0.0006918

LT-FH	0.000005	2.5253E-06	2.5252E-06
LT-FH	0.00005	6.1616E-05	2.4492E-05
LT-FH	0.0005	0.00049596	7.0626E-05
LT-FH	0.005	0.00507475	0.0002258
LT-FH	0.05	0.05006364	0.00069308
LT-FH++	0.000005	3.5354E-06	3.5353E-06
LT-FH++	0.00005	5.303E-05	2.2861E-05
LT-FH++	0.0005	0.00051263	7.179E-05
LT-FH++	0.005	0.00501919	0.00022455
LT-FH++	0.05	0.04988535	0.00069191

Table S6: Table of the false positive rate at varying levels of significance thresholds in the default simulation setup with a prevalence of 5% and downsampling of controls.

Method	Alpha level	Proportion of False positives	Standard error
GWAS	0.000005	4.0404E-06	3.4487E-06
GWAS	0.00005	6.2626E-05	2.4478E-05
GWAS	0.0005	0.00047071	6.8846E-05
GWAS	0.005	0.00484949	0.00022073
GWAS	0.05	0.04968586	0.0006906
GWAX	0.000005	7.0707E-06	5.8386E-06
GWAX	0.00005	4.3939E-05	1.9716E-05
GWAX	0.0005	0.00050303	7.0968E-05
GWAX	0.005	0.00499848	0.00022406
GWAX	0.05	0.05002525	0.00069283

LT-FH	0.000005	3.5354E-06	2.9436E-06
LT-FH	0.00005	4.899E-05	2.0835E-05
LT-FH	0.0005	0.00048939	7.0106E-05
LT-FH	0.005	0.00487525	0.00022135
LT-FH	0.05	0.04968333	0.00069058
LT-FH++	0.000005	8.0808E-06	6.8487E-06
LT-FH++	0.00005	4.596E-05	2.0976E-05
LT-FH++	0.0005	0.00048535	6.9909E-05
LT-FH++	0.005	0.00494747	0.00022295
LT-FH++	0.05	0.04999091	0.00069261

Table S7: Table of the false positive rate at varying levels of significance thresholds in the default simulation setup with a prevalence of 10%.

Method	Alpha level	Proportion of False positives	Standard error
GWAS	0.000005	1.1111E-05	9.9276E-06
GWAS	0.00005	4.3434E-05	2.0597E-05
GWAS	0.0005	0.0005101	7.1635E-05
GWAS	0.005	0.00507879	0.00022588
GWAS	0.05	0.04976263	0.0006911
GWAX	0.000005	7.0707E-06	6.1831E-06
GWAX	0.00005	6.2121E-05	2.456E-05
GWAX	0.0005	0.00052374	7.2577E-05
GWAX	0.005	0.00510808	0.00022654
GWAX	0.05	0.05011818	0.00069344

LT-FH	0.000005	6.5657E-06	5.9739E-06
LT-FH	0.00005	4.9495E-05	2.1948E-05
LT-FH	0.0005	0.00052222	7.2523E-05
LT-FH	0.005	0.00512071	0.00022682
LT-FH	0.05	0.04984899	0.00069167
LT-FH++	0.000005	9.596E-06	7.4763E-06
LT-FH++	0.00005	5.5556E-05	2.2965E-05
LT-FH++	0.0005	0.0005	7.091E-05
LT-FH++	0.005	0.00501616	0.00022451
LT-FH++	0.05	0.04996465	0.00069242

Table S8: Table of the false positive rate at varying levels of significance thresholds in the default simulation setup with a prevalence of 10% and downsampling of controls.

Method	Alpha level	Proportion of False positives	Standard error
GWAS	0.000005	5.0505E-06	3.7697E-06
GWAS	0.00005	4.3434E-05	2.0434E-05
GWAS	0.0005	0.00050505	7.1238E-05
GWAS	0.005	0.00494949	0.000223
GWAS	0.05	0.04972828	0.00069088
GWAX	0.000005	3.0303E-06	2.4386E-06
GWAX	0.00005	4.5455E-05	2.1107E-05
GWAX	0.0005	0.00050505	7.1313E-05
GWAX	0.005	0.00494545	0.00022291
GWAX	0.05	0.05018384	0.00069387

LT-FH	0.000005	2.0202E-06	2.0202E-06
LT-FH	0.00005	5.1515E-05	2.2289E-05
LT-FH	0.0005	0.00049293	7.0392E-05
LT-FH	0.005	0.0050101	0.00022436
LT-FH	0.05	0.05006162	0.00069307
LT-FH++	0.000005	5.0505E-06	3.0303E-06
LT-FH++	0.00005	4.8485E-05	2.1632E-05
LT-FH++	0.0005	0.00049192	7.031E-05
LT-FH++	0.005	0.00504848	0.00022521
LT-FH++	0.05	0.0501798	0.00069384

Table S9: Table containing the false positive rates with varying levels of alpha level for each of the considered methods with N = 300,000, 5% prevalence, no downsampling, and full family history and age-of-onset information. The other parameter setups can be

found in the supplementary data, and include 2 different prevalences, 4 different values of N, 4 different levels of completeness of family history and age-of-onset information.

Significant associations - Mortality

Variant ID	Chromosome :Position (hg38)	LT-FH++ P-value	Effect size (SE)	Nearest gene	Selected previously reported associations
rs429358	19:44908684	8.8e-52	-0.176493(0.0116573)	APOE	Alzheimer's[7], metabolic traits[80], mortality[60,70]
15:78828640	15:78828640	1.9e-22	0.088522 (0.00908256)	HYKK	Smoking and lung cancer[6], mortality[70]
rs10455872	6:160589086	7.5e-15	-0.120683 (0.0155212)	LPA	heart disease, mortality[70]
6:161075384	6: 161075384	5.1e-14	-0.243674(0.0323606)	MAP3K4	Endometriosis[81]
rs34386495	6:32658953	4.7e-10	0.0664307(0.0106654)	HLA-DQB1	Asthma[82], autoimmune diseases[83],

					mortality[70]
rs61905747	11:113769120	8.5e-9	-0.0620208(0.0107705)	ZW10	Glioma[84] mortality[70,85]
rs2507989	6:31356638	1.6e-8	-0.0592997(0.0104863)	HLA-B	White blood cell count[62], Psoriasis[63]
rs3838008	20:63357289-63357318 (indel)	1.9e-8	0.0608869 (0.0108248)	CHRNA4	Smoking and lung cancer[6], mortality[70]
rs17691989	13:77093116	4.4e-8	-0.1571(0.028695)	MYCBP2	Circadian rhythm (chronotype)[64]
rs79339645	3:166883110	4.7e-8	0.120294(0.0220177)	ZBBX	DNA methylation in older people[66]

Table S10: Independent LT-FH++ associations for mortality in UK biobank identified using COJO[61] and sorted by lowest p-value. The two strongest associations are shared with LT-FH, and seven out of three were previously identified in association studies of longevity[85] or parental age[70].

Significant associations - iPSYCH

Variant ID	Chromosome :Position (hg38)	LT-FH++ P-value	Effect size (SE)	Nearest gene	Selected previously reported associations
rs56022653	5:88588020	5.8e-12	0.132154(0.191985)	LINC00461	Educational attainment[68], ADHD[10,86]
rs11210887	1:43610348	1.1e-11	0.133962(0.0203968)	PTPRF	Smoking initiation[6], Educational attainment[68], ADHD[10,86]
rs9969232	7:114518899	2.1e-9	-0.120184(0.0200724)	FOXP2	Risk taking[87], ADHD[10]
rs6082363	20:21270205	5.0e-9	0.122019(0.0208684)	ZNF877P	ASD[9,88]
rs11030386	11:28609701	3.7e-8	-0.106526(0.0193581)	LINC02758	ADHD[10]

rs4261436	14:32830276	4.3e-8	-0.103069(0.0188137)	AKAP6	Cognitive traits[67,68]
rs7026534	9:134907263	4.7e-8	0.111291(0.0203778)		Education attainment, Smoking initiation [6,68]

Table S11: Independent LT-FH++ genome-wide significant associations for ADHD using COJO[61] and sorted by lowest p-value.

Variant ID	Chromosome :Position (hg38)	LT-FH++ P-value	Effect size (SE)	Nearest gene	Selected previously reported associations
rs910805	20:21248116	9.6e-15	0.194518 (0.0251149)	ZNF877P, AL117332.1	ASD[9]
rs4274907	4:135863730	7.7e-10	0.173381 (0.0281911)	LOC105377437	None reported

Table S12: Independent LT-FH++ genome-wide significant associations for ASD using COJO[61] and sorted by lowest p-value.

Table S13: Excel file containing all simulation results on power, mean causal and null chi-square test statistics, as well as their standard deviations. Furthermore, information on false positive rates in simulations are included for different significance levels (alpha levels), and the numbers from the run time simulations of LT-FH++.

Method	prev	downsampling	Symmetry test	Paired t-test	Wilcoxon Signed rank test	Paired Mcnemar
LT-FH++	10%	No	0	0.000160	0.00592	0
LT-FH++	10%	Yes	0	0.00000179	0.00586	0
LT-FH++	5%	No	0	0.0000208	0.00554	0
LT-FH++	5%	Yes	0	0.00000854	0.00563	0

Table S14: Table containing tests between LT-FH and LT-FH++ for significant differences. Symmetry test corresponds to a test for independence in a contingency table. The table contains the sum of all causal SNPs detected across all 10 simulations for each method in the first row and the sum of all undetected in the second. The paired t-test corresponds to a t-test on the average power across all 10 simulations with each group being a method. Wilcoxon signed rank test corresponds to a non-parametric test for difference in location between two data sets. Paired Mcnemar is a paired test for independence in a contingency table. All parameter setups showed that there was a significant difference between the number of SNPs found by LT-FH++ compared to LT-FH.

Method	prev	downsampling	diff_mean	diff_sd	ratio_mean	ratio_sd
GWAS	10%	No	-61.3	6.90	0.737	0.0267
GWAX	10%	No	-51	6.46	0.781	0.0294
LT-FH	10%	No	0	0	1	0
LT-FH++	10%	No	10.9	5.57	1.05	0.0241
GWAS	5%	No	-56.2	6.21	0.648	0.0306
GWAX	5%	No	-36.3	5.56	0.773	0.0249
LT-FH	5%	No	0	0	1	0

LT-FH++	5%	No	6.5	2.55	1.04	0.0181
GWAS	10%	Yes	-17.7	4.57	0.809	0.0458
GWAX	10%	Yes	-22.7	4.37	0.755	0.0480
LT-FH	10%	Yes	0	0	1	0
LT-FH++	10%	Yes	15.7	4.57	1.17	0.0546
GWAS	5%	Yes	-6.1	2.60	0.839	0.0606
GWAX	5%	Yes	-5	4.71	0.876	0.113
LT-FH	5%	Yes	0	0	1	0
LT-FH++	5%	Yes	6	2.11	1.16	0.0625

Table S15: Table containing the absolute and relative difference between LT-FH and all other considered phenotypes, case-control status (GWAS), GWAX, and LT-FH++. The differences are shown for each parameter configuration. The default simulation setup was used with a heritability of 50% and 1000 causal SNPs.
