# Supplementary material for: Accounting for age of onset and family history improves power in genome-wide association studies
Source: Am J Hum Genet. 2022 Feb 8;109(3):417–32. doi: 10.1016/j.ajhg.2022.01.009 (PMC8948165; doi:10.1016/j.ajhg.2022.01.009)
Supplement: Document S2. Article plus supplemental information [file mmc3.pdf]

Accounting for age of onset and family history improves power in genome-wide association studies

Authors

Emil M. Pedersen, Esben Agerbo,
Oleguer Plana-Ripoll, ..., John J. McGrath,
Florian Privé, Bjarni J. Vilhjálmsson

Correspondence

bjv@econ.au.dk (B.J.V.),
emp@ph.au.dk (E.M.P.)

Pedersen et al., 2022, The American Journal of Human Genetics *109*, 417–432
March 3, 2022 © 2022 The Authors.
https://doi.org/10.1016/j.ajhg.2022.01.009

CellPress

Accounting for age of onset and family history improves power in genome-wide association studies

Emil M. Pedersen,[1,2,*] Esben Agerbo,[1,2,3] Oleguer Plana-Ripoll,[1] Jakob Grove,[2,4,5,6] Julie W. Dreier,[1,3] Katherine L. Musliner,[1,2,3] Marie Bækvad-Hansen,[2,10] Georgios Athanasiadis,[11] Andrew Schork,[2,11] Jonas Bybjerg-Grauholm,[2,10] David M. Hougaard,[2,10] Thomas Werge,[2,11,12] Merete Nordentoft,[2,13] Ole Mors,[2,14] Søren Dalsgaard,[1] Jakob Christensen,[1,8,9] Anders D. Børglum,[2,6,7] Preben B. Mortensen,[1,2,3] John J. McGrath,[1,15,16] Florian Privé,[1,17] and Bjarni J. Vilhjálmsson[1,2,4,17,*]

Summary

Genome-wide association studies (GWASs) have revolutionized human genetics, allowing researchers to identify thousands of disease-related genes and possible drug targets. However, case-control status does not account for the fact that not all controls may have lived through their period of risk for the disorder of interest. This can be quantified by examining the age-of-onset distribution and the age of the controls or the age of onset for cases. The age-of-onset distribution may also depend on information such as sex and birth year. In addition, family history is not routinely included in the assessment of control status. Here, we present LT-FH++, an extension of the liability threshold model conditioned on family history (LT-FH), which jointly accounts for age of onset and sex as well as family history. Using simulations, we show that, when family history and the age-of-onset distribution are available, the proposed approach yields statistically significant power gains over LT-FH and large power gains over genome-wide association study by proxy (GWAX). We applied our method to four psychiatric disorders available in the iPSYCH data and to mortality in the UK Biobank and found 20 genome-wide significant associations with LT-FH++, compared to ten for LT-FH and eight for a standard case-control GWAS. As more genetic data with linked electronic health records become available to researchers, we expect methods that account for additional health information, such as LT-FH++, to become even more beneficial.

Introduction

Identifying the genetic variants underlying diseases and traits is a hallmark of human genetics. In recent years, large meta-analyses of genome-wide association studies (GWASs) have identified thousands of genetic variants for common diseases,[1–7] including psychiatric disorders,[8–12] revealing a remarkably complex and polygenic genetic architecture for most traits. International research collaboration where GWAS summary statistics have been shared in large consortia has been vital to this success, allowing researchers to obtain large sample sizes needed to study polygenic diseases. Novel advances in computational methods have also contributed to this success by enabling researchers to do more with less data.[13–17] Yet, for most of these traits and diseases, only a small fraction of the estimated heritable variation has been identified in GWASs,[18,19] highlighting the need for even larger samples and more powerful analysis methods.

Currently, most case-control GWASs are conducted with a regression model where the outcome is the case-control status or occasionally the age of onset of disease.[20] In this paper, we have opted for using the phrase age of onset over age at first diagnosis because they commonly refer to the same underlying thing, i.e., when a diagnosis is given. Recently, researchers have proposed several methods that leverage additional information to improve the power to detect genetic associations without having to increase the number of genotyped individuals. These include multivariate methods that leverage shared environmental or genetic correlations between traits and diseases[21–25] as well as methods that account for age of onset.[26–29] Perhaps the most fruitful development has come from methods that leverage family information to increase statistical power to identify associations, such as genome-wide association study by proxy (GWAX)[30,31] and liability-threshold-model-based approach.[32] The liability threshold model conditioned on family history (LT-FH)[32] estimates the

[1]National Centre for Register-Based Research, Aarhus University, 8210 Aarhus, Denmark; [2]Lundbeck Foundation Initiative for Integrative Psychiatric Research, 8210 Aarhus, Denmark; [3]Centre for Integrated Register-Based Research at Aarhus University, 8210 Aarhus, Denmark; [4]Bioinformatics Research Centre, Aarhus University, 8000 Aarhus, Denmark; [5]Department of Biomedicine and Center for Integrative Sequencing, Aarhus University, 8000 Aarhus, Denmark; [6]Center for Genomics and Personalized Medicine, Aarhus University, 8000 Aarhus, Denmark; [7]Department of Biomedicine - Human Genetics, Aarhus University, 8000 Aarhus, Denmark; [8]Department of Neurology, Aarhus University Hospital, 8200 Aarhus, Denmark; [9]Department of Clinical Medicine, Aarhus University, 8200 Aarhus, Denmark; [10]Center for Neonatal Screening, Department for Congenital Disorders, Statens Serum Institut, 2300 Copenhagen, Denmark; [11]Institute of Biological Psychiatry, MHC Sct. Hans, Mental Health Services Copenhagen, 4000 Roskilde, Denmark; [12]Department of Clinical Medicine, University of Copenhagen, 2200 Copenhagen, Denmark; [13]Mental Health Services in the Capital Region of Denmark, Mental Health Center Copenhagen, University of Copenhagen, 2100 Copenhagen, Denmark; [14]Psychosis Research Unit, Aarhus University Hospital, 8245 Risskov, Denmark; [15]Queensland Brain Institute, University of Queensland, St Lucia, QLD 4072, Australia; [16]Queensland Centre for Mental Health Research, The Park Centre for Mental Health, Wacol, QLD 4076, Australia
[17]These authors contributed equally
*Correspondence: bjv@econ.au.dk (B.J.V.), emp@ph.au.dk (E.M.P.)
https://doi.org/10.1016/j.ajhg.2022.01.009.
© 2022 The Authors. This is an open access article under the CC BY license (http://creativecommons.org/licenses/by/4.0/).

posterior mean genetic liability under the liability threshold model conditional on the case-control status of the individual, parents, and siblings. Here, "family history" refers to the case-control status of all family members, i.e., parents and siblings. As for GWAX, it considers any individual with a family member who has the disorder being studied as a case, increasing the number of cases. The GWAX phenotype remains a case-control phenotype. Although both GWAX and LT-FH can lead to power increases over case-control GWAS on real data, they achieve it in two different ways. It has been shown that GWAX can lead to a reduction in power when compared to a case-control GWAS; if the in-sample disease prevalence is high, however, LT-FH consistently provides an increase in power compared to case-control GWAS and GWAX.[32] This power improvement in LT-FH stems from two main sources. First, it distils family information and the individual's case-control status into a genetic liability estimate, resulting in a more informative outcome than the case-control status alone, to be used in GWASs. Second, it also allows researchers to include more individuals in their analysis. For instance, when studying breast cancer, we can derive the posterior genetic liability for genotyped males conditional on the family history for their mothers and sisters and thus include them in the GWAS.

However, family members often span a large age range, which can affect the expected disease prevalence because of changes in diagnostic methods and criteria over time. We refer to such differences in prevalence by birth year as "birth cohort effects." For instance, in the iPSYCH (Lundbeck Foundation Initiative for Integrative Psychiatric Research) data,[33] where genotyped individuals are born after 1980, we expect severe right censoring for many diagnoses. Survival models are routinely used in epidemiology to model time-to-event data in order to account for right censoring, time at risk, and age of onset as well as cohort effects.[34] They can be used to improve genomic prediction and predict disease progression[35,36] and have also been shown to provide up to 10% increase in power to detect genetic variants in GWASs when compared to standard logistic regression.[26] Recently, computationally efficient survival models for GWASs have been proposed: both Cox regression[29] and frailty models that can control for population and family structure in large samples.[27,28] However, to the best of our knowledge, these advanced time-to-event GWAS methods cannot account for family history (without genotype information for family members) to boost statistical power, as observed for LT-FH. Furthermore, LT-FH posterior liability estimates cannot be used directly as an outcome in survival analysis, as these are not binary and, more fundamentally, survival models are based on a different generative model than the liability threshold model. Hujoel et al.[32] proposed an approach to address this problem by accounting for age of onset in the genotyped individuals by linearly shifting the threshold for the genetic liabilities based on observed in-sample prevalence in different age groups but did not observe any improvements in power. We believe that this approach was unsuccessful in part because the in-sample estimate of the prevalence is subject to both a survival and selection bias and does not properly reflect prevalence in the population.

In this paper, we propose LT-FH++, a method that extends the model underlying LT-FH to account for information such as right censoring, age of onset, sex, and cohort effects. We achieve this by using a personalized threshold for each person (including family members), conditional on available information as well as general population incidence rates by age, sex, and birth year. LT-FH++ has been implemented into an R package (see data and code availability), which utilizes a Gibbs sampler implemented in C++ through the Rcpp R package.[37] The personalized thresholds are made possible by replacing the Monte Carlo sampling used by Hujoel et al. with a much more efficient Gibbs sampler. The Gibbs sampler allows us to estimate the posterior mean genetic liability for each individual independent of one another, thereby making it highly scalable.

First, we perform a GWAS with the standard case-control phenotype as well as GWAX, LT-FH, and LT-FH++ outcomes for simulated data with the liability threshold model as the generative model. For real-world application, we analyzed mortality in the UK Biobank and four psychiatric disorders in the iPSYCH cohort.

Material and methods

Model

The underlying model is identical to the one used in LT-FH;[32] as a result the model will only briefly be presented here, and the main differences will be elaborated on. Under the liability threshold model, each individual has a liability, ℓ, which follows the standard normal distribution. An individual will be considered a case, $z = 1$, when their liability is above a given threshold, i.e., $\ell \geq T$, and a control, $z = 0$, if the liability is below the threshold, $\ell < T$. The threshold, T, is determined from the prevalence of the dichotomous disorder, such that $P(\ell \geq T) = K$, where K denotes the prevalence in the population.

LT-FH builds on this idea, and for a single individual, the liability is assumed to be further decomposed into a genetic and environmental component, $\ell = \ell_g + \ell_e$. Both ℓ_e and ℓ_g are normally distributed and independent. We have

$$\ell_g \sim N(0, h^2), \ell_e \sim N(0, 1 - h^2)$$

Here, h^2 is the heritability on the liability scale. The LT-FH setup extends this idea to include parents and siblings. It considers a multivariate normal distribution given by

$$\ell = (\ell_g, \ell_o, \ell_{p_1}, \ell_{p_2}, \ell_s) \sim N(0, \Sigma),\ \Sigma = \begin{bmatrix} h^2 & h^2 & 0.5h^2 & 0.5h^2 & 0.5h^2 \\ h^2 & 1 & 0.5h^2 & 0.5h^2 & 0.5h^2 \\ 0.5h^2 & 0.5h^2 & 1 & 0 & 0.5h^2 \\ 0.5h^2 & 0.5h^2 & 0 & 1 & 0.5h^2 \\ 0.5h^2 & 0.5h^2 & 0.5h^2 & 0.5h^2 & 1 \end{bmatrix}.$$

Here, ℓ_o denotes the full liability for the individual (denoted ℓ for a single individual above), and ℓ_g denotes the genetic component

of this liability. ℓ_{p_1} and ℓ_{p_2} denotes the *full* liability of each parent, while ℓ_s denotes those of the sibling. The example above includes one sibling only, but in theory any number of siblings could be included in the model. We are interested in estimating the posterior mean genetic liability for each individual conditional on family information:

$$\boldsymbol{E}\left[\ell_g|\boldsymbol{Z}\right], \boldsymbol{Z} = \left(z_o, z_{p_1}, z_{p_2}, z_s\right)^T.$$

Here, **Z** denotes the vector of status for the family, consequently a restriction is placed on each individual's full liability. In the case of everyone's having the disorder, we would consider the space $\{\ell \in R | \ell_i \geq T_i \text{for all } i\}$, where i denotes a family member, T_i denotes the family member's threshold, and ℓ_i denotes their full liability. In LT-FH, the thresholds are the same for all children (the offspring and any siblings), and another threshold is used for all parents.

The choice of thresholds is where LT-FH++ starts to differentiate itself from LT-FH. In short, the liability thresholds are personalized, such that every individual, sibling, or parent has a potentially unique threshold that is determined by their age, birth year, and sex. Furthermore, we adopt an age-dependent liability threshold model, where the threshold is dynamic in the sense that it decreases as a population grows older. This idea is illustrated in Figure 1A, where the threshold decreases as time progresses for a population, with marks for ages 15, 25, 35, 50, and 80. This model assumes that the threshold decreases continuously as time progresses, and these marks can be seen as snapshots in time, where an individual who was diagnosed at one of the marks had an assumed (fixed) liability equal to said mark. This age-dependent liability threshold model allows us to be very precise with the liability for cases when an accurate age of onset is available. If an accurate estimate of age of onset is not available, then the threshold can still be personalized on the basis of other available information, with the modification that we do not fix the full liability but integrate over all liabilities above the personalized threshold. Interestingly, the age-dependent liability threshold model can be thought of as a survival model (see below).

Another point where LT-FH++ differs from LT-FH is in how siblings are included. LT-FH includes the siblings by specifying the number of siblings and assigns a single case-control status to the siblings with the condition that at least one sibling has the disorder. However, a more fine-grained inclusion of the siblings, where each sibling is added individually, is not available. LT-FH++ expects each individual and their family members to be added separately, such that information on each individual can be accounted for.

Relationship with survival analysis

In survival analysis GWASs, the risk for becoming a case in a time interval depends on the covariates in the model. This is reflected by a hazard rate $\lambda(t|x)$, which describes the event rate. In our context, it would refer to the rate for becoming a case. This rate depends on both time t, and covariates of the model x, e.g., genotypes. The hazard rate (also referred to as the intensity) can be approximated by $\lambda(t|x) \approx \frac{P(T(t+dt)<\ell|T(t)>\ell,x)}{dt}$, where dt is a small change in time,[39] $T(t)$ is the threshold for being a case at time t, and ℓ is the full liability of an individual. This means that the hazard rate is proportional to the probability an event occurs within a time interval $(t, t+dt)$, given that no event had occurred earlier. For different types of survival analyses, we can estimate this probability by using the hazard rate, e.g., for a Cox proportional hazards model where we aim to estimate the effect of a genotype x on the hazard rate, it becomes $P(T(t+dt) < \ell | T(t) > \ell, x) = dt\lambda(t|x) = dt\lambda_0(t)exp(\beta x)$. To keep notation simpler, we will denote the genetic liability of individual i as g_i instead of ℓ_{g_i}, and if we further assume that the genetic component for an individual of a case-control outcome contributes to the hazard rate such that $\lambda(t|g_i) = \lambda_0(t)exp(g_i) = \lambda_0(t)exp(\beta x_i)$, where x_i denotes the genotype of the ith individual and β their true effects (in the Cox-regression model). Conceptually, this means that individuals with higher than average genetic risk, i.e., $g_i > 0$, will be at higher risk to become cases throughout their lives, irrespective of age. These high-risk individuals will on average also have earlier age of onset.

To understand how this model relates to the proposed age-dependent liability threshold model, we can derive the same probability to approximate the corresponding hazard rate. Under the LT-FH++ model, the probability for an individual i to be diagnosed (become a case) within a time interval dt can be written as $P(T(t+dt) \leq \ell_i | T(t) > \ell_i, g_i)$, where t again denotes the age of the individual and $T(t)$ now denotes the age-dependent liability threshold. We note that $T(t)$ is a monotonic decreasing function as the prevalence of a case-status (i.e., cumulative lifetime incidence proportion) always increases with age (conditional on birth year and sex). Furthermore, ℓ_i denotes the full liability of the individual and g_i the genetic component of that liability (which is generally on a different scale than a genetic component in Cox regression). The liability threshold model assumes that the liability of an individual consists of genetic and environmental components, i.e., $\ell_i = g_i + e_i$. It also assumes that these are independent, follow a Gaussian distribution, and have variance h^2 and $1 - h^2$, respectively. Hence using these, we can expand the probability of being diagnosed within a time interval dt further as follows:

$$\begin{aligned}
&P(T(t+dt) \leq \ell_i | T(t) > \ell_i, g_i) \\
&= P(T(t+dt) \leq \ell_i < T(t) | g_i) \times (P(T(t) > \ell_i | g_i))^{-1} \\
&= \left(\Phi\left(\frac{T(t) - g_i}{\sqrt{1-h^2}}\right) - \Phi\left(\frac{T(t+dt) - g_i}{\sqrt{1-h^2}}\right)\right) \\
&\times \left(\Phi\left(\frac{T(t) - g_i}{\sqrt{1-h^2}}\right)\right)^{-1} = 1 - \Phi\left(\frac{T(t+dt) - g_i}{\sqrt{1-h^2}}\right) \\
&\times \left(\Phi\left(\frac{T(t) - g_i}{\sqrt{1-h^2}}\right)\right)^{-1}.
\end{aligned}$$

Plotting this function for different thresholds and genetic liability values shows that the probability for being diagnosed within the time interval, and thus the hazard rate, increases linearly as a function of the genetic liability when g_i is near $T(t)$ or larger. We compare this probability with the corresponding Cox regression probability assuming a base incidence rate of $\lambda_0(t) = \alpha$, where α is determined by the prevalence. These two probabilities, which are proportional to the hazard rate, are plotted as a function of g_i in Figure S50, illustrating how the hazard rates of the two models depend on g_i. We note that the two models share the properties that individuals with higher than average genetic risk will, on average, be more likely to become cases within any time interval and have earlier age of onset.

It may seem counterintuitive that a deterministic model such as the age-dependent liability threshold model, where the liability is constant throughout life, can be recast as a survival analysis model. The reason for this is that although the outcome of the age-dependent liability threshold model is always known

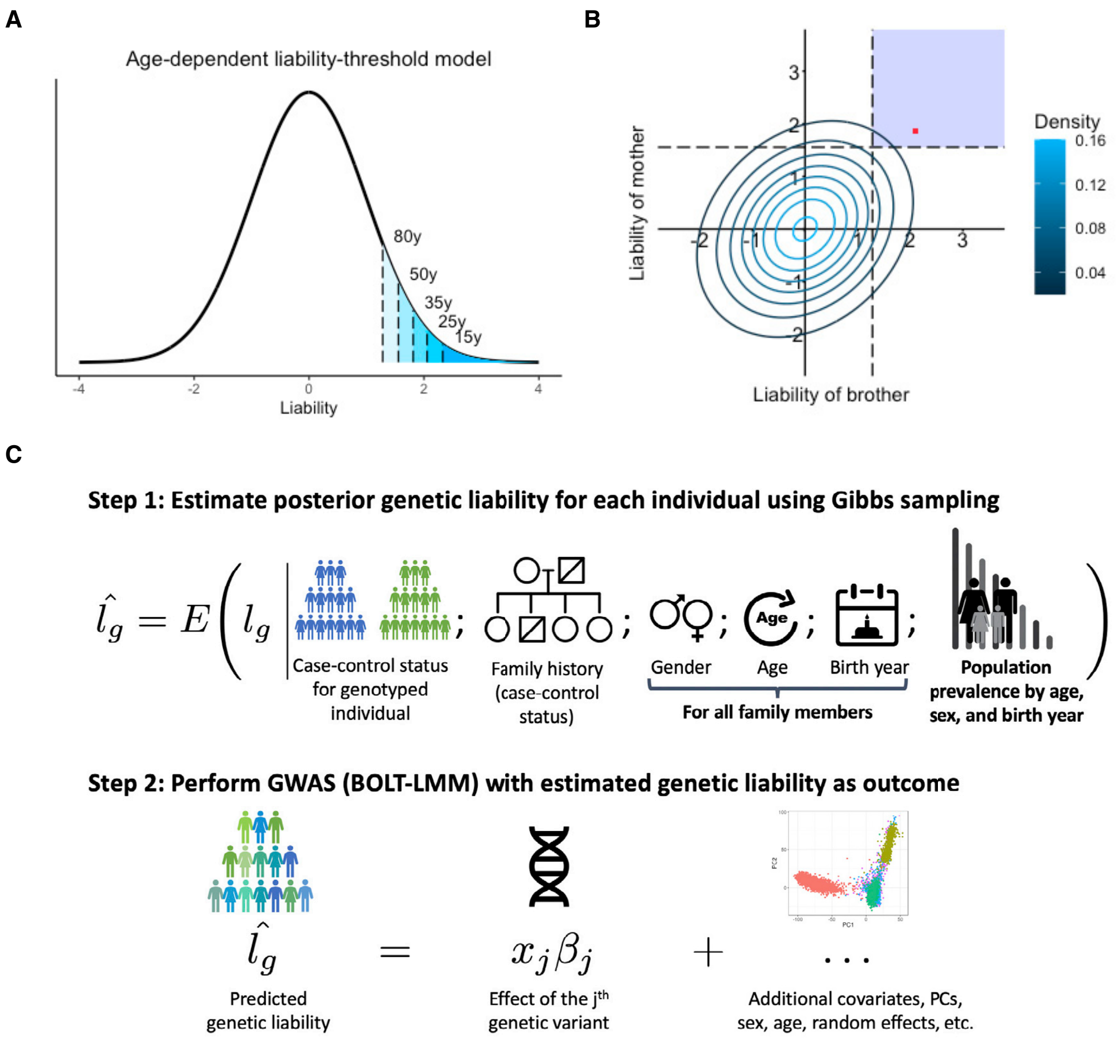

Figure 1. Overview of LT-FH++ and illustration of the differences between LT-FH and LT-FH++

(A and B) An age-dependent liability threshold model with different thresholds marked (A). The marks correspond to the prevalence at the age of 80 years (10%), 50 years (6%), 35 years (3.5%), 25 years (2%), and 15 years (1%). The posterior mean estimate of the liability is obtained by integrating over the liability space spanned by the genotyped individual and their family members (B). Here, we consider a brother and a mother, where the contour lines indicate the joint multivariate liability density of the mother and the brother (assuming a heritability of 0.5). Using fixed population prevalence for males and females (dashed lines), and assuming mother and brother are cases, LT-FH integrates over the blue shaded area to estimate the genetic liability. In contrast LT-FH++ considers the age of onset, sex, and birth year for family members to obtain a more precise genetic liability estimate highlighted by the red dot. In short, the additional information collapses the area to integrate to a single value.

(C) An overview of how LT-FH++ GWAS works and what information it accounts for. In contrast to LT-FH, which accounts for the case-control status of the genotyped individual and family history, LT-FH++ also uses population prevalence information to account for gender, age, and birth year of family members. As with LT-FH, the predicted liabilities are then used as a continuous outcome in a GWAS via BOLT-LMM.[38]

given the liability, one never observes this liability. Hence, the environmental term, which can be thought of as capturing various environmental effects as well as chance events and other non-genetic effects, leads to a non-deterministic survival analysis model.

Sampling strategy

If we consider an individual with disease status available for both parents, but no siblings, then we have a total of six unique ways to configure the status vector, $\boldsymbol{Z}$, when disregarding other information because the scenario where a single parent is a case can happen in two ways. LT-FH estimates the posterior mean genetic liability for each of these configurations by sampling a large number of observations from the multivariate normal distribution described above. The observations are then grouped into these six unique configurations, and the genetic liabilities are estimated by averaging genetic liabilities within each configuration. This strategy works well when there are a limited number of configurations but becomes infeasible when the number of configurations becomes too large.

LT-FH++ cannot efficiently use the same sampling strategy because the personalized thresholds increase the number of potential configurations such that the strategy becomes intractable. Instead LT-FH++ considers each family as a unique configuration because it uses individualized thresholds. To derive the posterior means efficiently, we use a Gibbs sampler to sample from a

truncated multivariate normal distribution.[40] The truncation points in the truncated multivariate normal distribution are the personalized thresholds. Sampling for all individuals is fast, requires far fewer observations, and can be easily parallelized across individuals, as each family is independent from each other.

Practical considerations for LT-FH++ GWAS

When deriving the posterior mean genetic liabilities, it is important to ensure that the genotyped individuals do not have shared family members, as that can otherwise lead to individuals' being more correlated than expected given their genetic similarity.[32] This can cause problems in subsequent GWAS analysis and lead to inflation of false positive rates. We therefore recommend only applying LT-FH++ to unrelated individuals, where the relatedness threshold is stringent enough to ensure that no genotyped pair of individuals have common family members.

As LT-FH++ reports effects on a genetic liability scale, these can be hard to interpret. However, the general strategy proposed by Hujoel et al.[32] can be used to transform these to per-allele observed-scale effect sizes for non-standardized phenotypes.

LT-FH++ has several ways to deal with missing information. If age-of-onset information is missing for an individual, the threshold used for that individual will correspond to the average prevalence (the LT-FH threshold). If age-of-onset information is available for the family members, their threshold can still be personalized. The estimated genetic liability under LT-FH++ with no age-of-onset information available for an individual and their family members but complete family history information would be identical to the LT-FH estimate. If case-control status is missing for the genotyped individual, we integrate over the entire range of liabilities for this individual. If case-control status is missing for family members, we exclude these from the analysis. For example, if the case-control status is known for one parent but not the other parent, we exclude the second parent from the analysis. Finally, age-of-onset information acts as an additional level of fine-tuning in the age-dependent liability threshold model. In our analysis, the threshold depends on sex, birth year, and age or age of onset, but if less information is available, e.g., no sex, then an estimate of the threshold could still be based on the birth year and age or age of onset. Similarly, if prevalence estimates are known for a given (categorical) risk factor (e.g., smoking status), then LT-FH++ can account for this additional risk factor (also in family members).

Prevalence information

The age-dependent prevalence of attention deficit-hyperactivity disorder (ADHD [MIM: 143465]), autism spectrum disorder (ASD [MIM: 209850]), depression (DEP [MIM: 608516]), and schizophrenia (SCZ [MIM: 181500]) was obtained through Danish national population-based registers. For these estimates, we included all 9,251,071 persons living in Denmark at some point between January 1, 1969 and December 31, 2016. Each individual in the study was followed from birth, immigration to Denmark, or January 1, 1969 (whichever happened last) until death, emigration from Denmark, or December 31, 2016 (whichever happened first). All dates were obtained from the Danish Civil Registration System,[41] which has maintained information on all residents since 1968, including sex, date of birth, continuously updated information on vital status, and a unique personal identification number that can be used to link information from various national registers. Information on mental disorders was obtained from the Danish Psychiatric Central Research Register,[42] which contains data on all admissions to psychiatric inpatient facilities since 1969 and visits to outpatient psychiatric departments and emergency departments since 1995. The diagnostic system used was the Danish modification of the *International Classification of Diseases, Eighth Revision (ICD-8)* from 1969 to 1993, and *Tenth Revision (ICD-10)* from 1994 onward. The specific disorders were identified with the following ICD-8 and ICD-10 codes: ADHD (308.01 and F90.0), autism (299.00, 299.01, 299.02, 299.03 and F84.0, F81.4, F84.5, F84.8, F84.9), depression (296.09, 296.29, 298.09, 300.49 and F32, F33), and schizophrenia (295.x9 excluding 295.79 and F20). For each individual in the study, the date of onset for each disorder was defined as the date of first contact with the psychiatric care system (inpatient, outpatient, or emergency visit). All analyses were done separately for each sex and for each birth year. The cumulative incidence function for each disorder was estimated with the Aalen-Johansen approach considering death and emigration as competing events.[43] The cumulative incidence over age is interpreted as the proportion of persons diagnosed with the specific disorder before a certain age.

Personalized thresholds

With the cumulative incidence rate tables, we are able to assign personalized thresholds to everyone with sufficient information available. Examples of cumulative incidence rate curves can be seen in Figures S21, S27, S32, S38, and S44. Under the liability threshold model, sex, birth year, and age for controls or age of onset for cases can uniquely determine the threshold for an individual. On the basis of this information, a proportion is assigned to them, which is transformed to an individual's threshold through the inverse normal cumulative distribution function.

For controls, it has allowed us to tailor the threshold in the liability threshold model to each individual, similar to what is seen in Figure 1A, where the threshold is decreasing as an individual is getting older. In short, the older a control is, the larger a proportion of the possible liabilities in the liability threshold model can be excluded as no longer attainable. For cases, the tailored threshold means we are able to very accurately estimate what a person's *full* liability is for a given disorder under the liability threshold model. Because the full liability can be accurately estimated for a case by the assigned threshold, we will fix the full liability of a case to be the threshold in the model.

Simulation details

For the simulations, we simulated 100,000 unrelated individuals each with 100,000 independent single-nucleotide polymorphisms (SNPs). We simulated two parents and between zero and two siblings. The parents' genotypes were drawn from a binomial distribution with probability parameters equal to the allele frequency (AF) of the corresponding variant. The variant AF was drawn from a uniform distribution on the interval $(0.01, 0.49)$. The parents' genotypes were either 0, 1, or 2; we defined the child's genotypes as the average between the genotypes of both parents, rounding values of 0.5 or 1.5 up or down with equal probability. Allele effect sizes were drawn from $N\left(0, h^2/C\right)$, where C was the number of causal SNPs and h^2 denoted the heritability. Case-control status was assigned with a liability threshold model.

The default simulation setup consisted of causal SNPs assigned to positions at random, two different prevalenes, 5% and 10%, C set to 1,000, and a sex-specific prevalence of 8% for men and 2% for women. When the prevalence was 10%, these sex-specific

Table 1. Breakdown of the number of cases and controls for mortality for the UK Biobank participants (here children) and their parents

Mortality					
**Participants**		**Father**		**Mother**	
Case	control	case	control	case	control
13,819	323,656	258,932	75,545	199,856	130,757

The case-control GWAS only used the children column as input, while LT-FH and LT-FH++ used all columns.

prevalences were doubled. To generate the age of onset, we assumed that the cumulative incidence curve followed a logistic function because it resembles real-world cumulative incidence rates for some traits, see Figures S21, S27, S32, S38, and S44. The logistic function is given by

$$T(x) = \frac{L}{1 + e^{-k(x - x_0)}}$$

where L denotes the maximal attainable prevalence value, k is the growth rate, and x_0 denotes the age (in years) at which K is $L/\ 2$, which is the midpoint of the curve, i.e., median age of onset. Due to the properties of the function, the lifetime prevalence will only be approximately L (only slightly smaller). These parameters resulted in an age of onset that was largely normally distributed around the median age, x_0. The cumulative incidence rate curve allows us to obtain the expected prevalence at each age, which we can then translate into a threshold in the liability threshold model, i.e., an earlier diagnosis indicates higher liability for the trait. We fix the lifetime prevalence L in the combined population and the corresponding sex-specific lifetime prevalences. We then assigned each individual a male or female sex with equal probability. In our simulation, we assumed males were four times as likely to be cases than females. For the two lifetime prevalences (5% and 10%), this corresponded to 8% and 16% prevalence among males (liability thresholds $T_{male} = 1.41$ and $T_{male} = 0.99$) and 2% and 4% prevalence among females (liability thresholds $T_{female} = 2.05$ and $T_{female} = 1.75$). E.g., with an overall prevalence of 5%, we used $L = 0.08$ for males and $L = 0.02$ for females. We also set k to 1/8 and x_0 to 60 such that 90% of cases have an age of onset between 36.5 and 83.5.

A family consisted of one offspring, two parents, and zero to two siblings. The age of the cases was set to the age of onset. The age of onset was assigned by taking the inverse of the logistic function on the full liability's quantile under the standard normal distribution. Individuals with an age lower than their age of onset would normally be considered controls because they had not yet had the time to develop the disorder. However, setting high liability individuals to controls because age of onset was later than age was decided against to properly fix the number of cases to the prevalence in the simulated data. For controls, the offspring's age was uniformly distributed between 10 and 60. The parents' age was set to the age of the child plus a uniform draw between 20 and 35, allowing for up to 95 year olds. The threshold was assigned with the logistic function with the age and sex as inputs. For simplicity, birth year was not modeled. Finally, we simulated sample ascertainment by downsampling controls such that cases and controls had equal proportions (50% each). For 5% prevalence, this resulted in a sample size of 10,000 and 20,000 individuals when using a prevalence of 10%.

GWAS in UK Biobank

We restricted individuals to the White British group (field 22006) and to the individuals used for computing the principal components (PCs) in the UK Biobank (field 22020). These individuals are unrelated and have passed some quality control (see section S3 of Bycroft et al.[44]). This resulted in 337,475 individuals. Table 1 shows a breakdown of how many people are cases and controls for the genotyped individuals and parents. We used the genotyped SNPs for the UK Biobank participants as model SNPs in BOLT-LMM,[38] after removing SNPs with minor allele frequency (MAF) < 0.01, missing call rate > 0.01, and Hardy-Weinberg equilibrium p value $< 1 \times 10^{-50}$, which left us with a total of 504,138 SNPs. When performing the GWAS, we used the imputed SNPs in bgen files and removed SNPs with an MAF < 0.005 or info score $<$ 0.6, which resulted in 11,335,564 SNPs. We used BOLT-LMM v2.3.2 with age, sex, and the first 16 PCs as covariates. The three mortality outcomes used in the UK Biobank were case-control status, LT-FH, and LT-FH++. We considered the binary death outcome as the case-control phenotype, and LT-FH and LT-FH++ further utilize the mortality status of both parents but no siblings. The UK Biobank data was downloaded on the March 17, 2020.

LT-FH++ and LT-FH require prevalence information, which was acquired from the Office for National Statistics (ONS). Mortality rates for England and Wales were available from 1841 to the present day. The same information was available for all of the United Kingdom (UK), but only from the 1950's onward. Because England is the most populous country in the UK, we believe these mortality rate estimates are a good proxy for all of the UK. From the mortality rates provided by ONS, we calculated the cumulative incidence curves for death for each birth year from 1841 onward and for both sexes. We used this information to calculate the personalized thresholds in LT-FH++, accounting for birth year, sex, and current age or age of death.

To determine the birth year of the parents in the UK Biobank, we assumed they were 25 years older than their child (for which year of birth is available in data field 34). The resulting estimated birth year was then used in the prevalence curves to get the liability thresholds for each parent. Age of death for the parents are available in data fields 1807 and 3526.

Note that, in LT-FH, it is not possible to adjust for sex, age, or cohort effects at the individual level, but two different thresholds can be specified, one for all parents and one for all children. Therefore, we assumed the same age for all children and the same age for all parents when running LT-FH. We used the last recorded death as the endpoint, which happened in 2018, and assumed all children were 55 years old and parents were 85 years old. This translated into an assumed birth year of 1963 and 1933, respectively. On the basis of these birth years, we found the prevalence of death for these birth years and ages in the survival curve and averaged the sex-specific prevalences. For LT-FH, we also considered thresholds on the basis of prevalence estimated in the UK Biobank participants and their parents, however we did not see any significantly different results when comparing to the population-based prevalence estimates (results not shown). A heritability of 20% was used for LT-FH and LT-FH++.

GWAS in iPSYCH

The iPSYCH cohort has recently received a second wave of genotyped individuals, increasing the number of genotyped individuals from ~80,000 to ~143,000.[45] The two iPSYCH waves have been imputed separately with the Ricopili pipeline.[46] After

Table 2. **Breakdown of how many cases and controls each GWAS was performed with**

	Children		Father		Mother		Sibling status			
	**Case**	**Control**	**Case**	**Control**	**Case**	**Control**	**0**	**1**	**2**	**3**
ADHD	21,255	36,584	498	57,001	751	57,088	43,558	1,777	78	<5
ASD	18,076	36,781	84	54,438	76	54,781	42,585	1,359	62	6
Depression	27,266	38,882	2,632	63,164	4,336	52,821	50,449	2,281	86	5
Schizophrenia	5,749	36,961	429	42,051	576	34,871	34,358	494	16	<5

The sibling status refers to the number of affected siblings that each genotyped individual has. For case-control outcome, only the children column was used. For LT-FH and LT-FH++, all columns were used. LT-FH only included a binary variable for sibling status; for ASD, this meant 1,427 satisfied the "at least one sibling is a case" condition of LT-FH, while 42,585 had siblings, but none of them had been diagnosed with ASD. Differences between the number of cases and controls for a trait and sibling status are due to some individuals having no siblings and thus no sibling status.

combining the two waves and removing any SNP with missingness > 0.1 or MAF < 0.01, we have a total of 4,706,774 SNPs. When performing a GWAS, we restrict the analysis to individuals classified as controls in the iPSYCH design and individuals diagnosed with the analyzed phenotype, even when using LT-FH or LT-FH++. We filtered for relatedness with a 0.0884 KING-relatedness cutoff and restricted the analysis to a genetically homogeneous group of individuals by calculating a Mahalanobis distance based on the first 16 PCs and keeping individuals within a log-distance of 4.5.[47] For a breakdown of the number of individuals included in each GWAS and the number of cases and controls, see Table 2. We used BOLT-LMM[38] v2.3.2 to perform the GWAS with sex, age, wave, and the first 20 PCs as covariates. LT-FH and LT-FH++ require an estimate for the heritability; we used 75% for ADHD,[48] 83% for autism,[49] 37% for depression,[50] and 75% for schizophrenia.[50,51] See prevalence information for details on how the cumulative incidence curves were derived.

When assessing power between outcomes, we considered SNPs that are in the iPSYCH cohort and have been found to be significantly associated with the psychiatric disorder being analyzed in the largest publicly available meta-analyzed GWAS.[8–10,52] We used PLINK to perform linkage disequilibrium (LD) clumping on the external summary statistics. We used PLINK's default parameters, except for the significance thresholds. The PLINK p value threshold we used was 5×10^{-6} for both the index SNPs and the clumped SNPs. We used the default window size of 250 kb and the LD threshold of 0.5.

Results

Overview of methods

The LT-FH++ method proposed here extends the LT-FH method to account for additional information for family members, such as age, sex, and cohort effects for case-control outcomes. LT-FH assumes a liability threshold model, where every individual has an underlying liability for the outcome but only becomes a case if the liability exceeds a given threshold, which is determined by the sample or population prevalence.[53] It further assumes that the covariance structure depends on the heritability and relatedness coefficient between each individual, which is a reasonable assumption for polygenic case-control diseases.[54,55] Under these assumptions, LT-FH estimates the posterior mean genetic liability conditional on the case-control status of the genotyped individual and their family members via a Monte Carlo sampling. The posterior mean genetic liability is then used as the continuous outcome in a GWAS, e.g., with BOLT-LMM.[38]

In LT-FH++, we introduce an "age-dependent liability threshold model" to capture the effect of age and replace the Monte Carlo sampling with a much more computationally efficient Gibbs sampler. Illustrated in Figure 1A, the age-dependent liability threshold model extends the liability threshold model by assuming that the threshold for becoming a case at a given age corresponds to the prevalence of the disease at that age. Interestingly, this model can be viewed as a type of survival analysis (see material and methods). We can then account for additional information, such as birth year and sex, by further conditioning the disease prevalence on this information. This leads to an individualized disease liability threshold for each person, including family members, which in practice requires us to be able to estimate separate genetic liabilities for each individual. This is made possible by replacing the Monte Carlo strategy of LT-FH with the computationally efficient Gibbs sampler that can sample from multivariate truncated Gaussian distributions to obtain personalized genetic liability estimates. As illustrated in Figure 1B, this results in more precise genetic liability estimates for LT-FH++ under the model compared to LT-FH, which for a population translates also into more variable genetic liability estimates (see Figure S1). Thus, in order to reap the full benefit of LT-FH++, it requires prevalence information to be available by age, sex, and birth year. Fortunately, such information is often partially or fully available on a population level, e.g., in the Danish registers.[56] The use of population prevalence information also allows LT-FH++ to estimate the genetic liability on a population scale, which may also reduce the risk of ascertainment and selection bias.[57–59] We summarize the information that LT-FH++ can account for and the two-step procedure of estimating individual genetic liabilities and performing GWASs on these in Figure 1C.

Simulation results

We examined the performance of LT-FH++ by using both simulated and real data. We simulated 100,000 unrelated

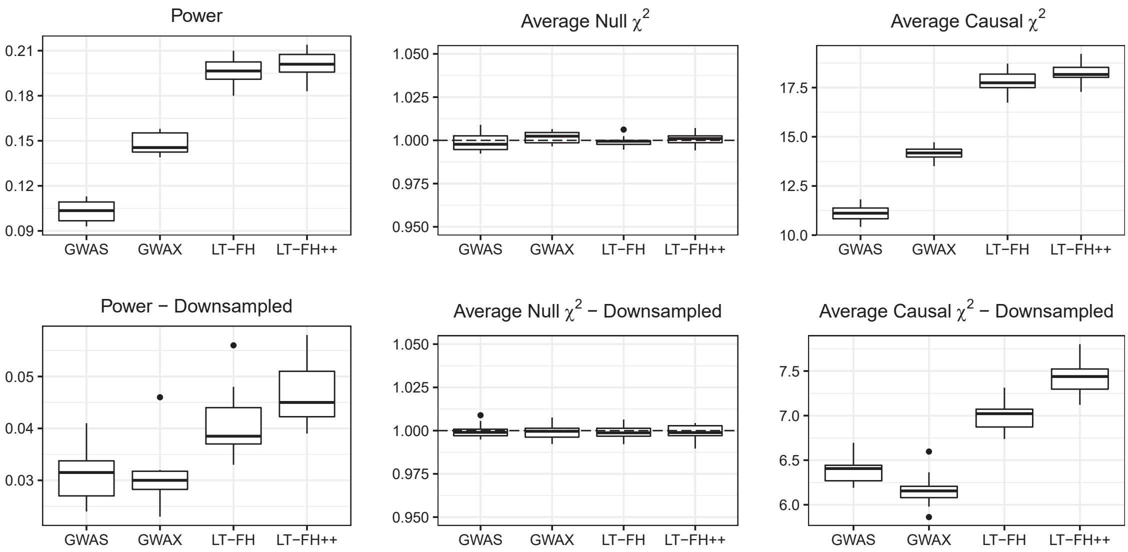

Figure 2. Simulation results for a 5% prevalence, with and without downsampling of controls
Linear regression was used to perform the GWAS for LT-FH and LT-FH++, while a 1-df chi-squared test was used for case-control status. We assessed the power of each method by considering the fraction of causal SNPs with a p value below 5×10^{-8}. Here, GWAS refers to case-control status and LT-FH and LT-FH++ are both without siblings. Downsampling refers to downsampling the controls such that we have equal proportions of cases and controls, i.e., we have 10,000 individuals total for a 5% prevalence and 20,000 individuals for a 10% prevalence.

individuals each with 100,000 independent SNPs and their family (two parents and 0–2 siblings). We generated case-control outcomes under the liability threshold model and assigned age of onset by assuming the prevalence followed a logistic curve as a function of age (see material and methods for simulation details).

We first considered the simulations for families with no siblings. We benchmarked LT-FH++ against case-control status and LT-FH. The results for 5% prevalence are shown in Figure 2, and the results for 10% prevalence can be found in Figure S12. We simulated sample ascertainment by downsampling controls such that cases and controls had equal proportions (50% each), which translated into a total of 10,000 individuals for a 5% prevalence and 20,000 individuals for a 10% prevalence. The simulation results confirmed the increase in power (number of causal SNPs detected) of LT-FH over standard GWASs when accounting for family history.[32] When also accounting for sex differences and age in LT-FH++, we observed a further increase in power, especially when the cases were ascertained (downsampling controls). Averaging over ten simulations, LT-FH had a power improvement over standard GWASs between 14% and 54%, where less power improvement was observed when downsampling controls. In contrast, the average power increase for LT-FH++ and standard GWASs was between 34% and 61%. Without downsampling controls, the relative improvements of LT-FH++ over LT-FH for a 5% and 10% prevalence were 4% and 5%, respectively. However, when downsampling controls, we observed an improvement of 18% for a 5% prevalence and 15% for a 10% prevalence. In Table S14, p values for various tests of difference between LT-FH and LT-FH++ can be seen. All tests showed a significant difference between them, in favor of LT-FH++. In Table S15, the absolute and relative difference in the number of causal SNPs detected within each simulated dataset and for each phenotype compared to LT-FH is shown. When simulating families with two siblings, we observed an increase in mean power and causal test statistics (across the ten simulations) compared to families with no siblings, but the *relative* improvement of LT-FH++ over LT-FH remained the same (results not shown).

We also assessed the robustness of LT-FH++ by misspecifying model hyper-parameters, i.e., the heritability and prevalence parameters. Simulated heritability was 50%, and when misspecifying it, we used 25% and 75%. For the prevalence, we used simulated values of either 5% or 10% and used either half or double of the true value to assess the impact of misspecifying this parameter. This resulted in, e.g., a prevalence of 5% or 20% when the true prevalence was 10%. In Figures S4, S5, S13, and S14, when misspecifying the heritability and prevalence, we see similar results as in Figure 2 with nearly identical mean null χ^2 statistics, mean causal χ^2 statistics, and power. LT-FH++ is therefore robust to misspecification of heritability and prevalence.

To better understand when one could expect gain from accounting for age of onset and family history, we performed additional simulations where we varied the number of individuals N as well as the completeness/missingness of the family history and age-of-onset information (see Figures S6–S11 and S15–S20 and Table S13). We found that the relative gain in statistical power of using LT-FH++ instead of LT-FH was largely constant when varying sample

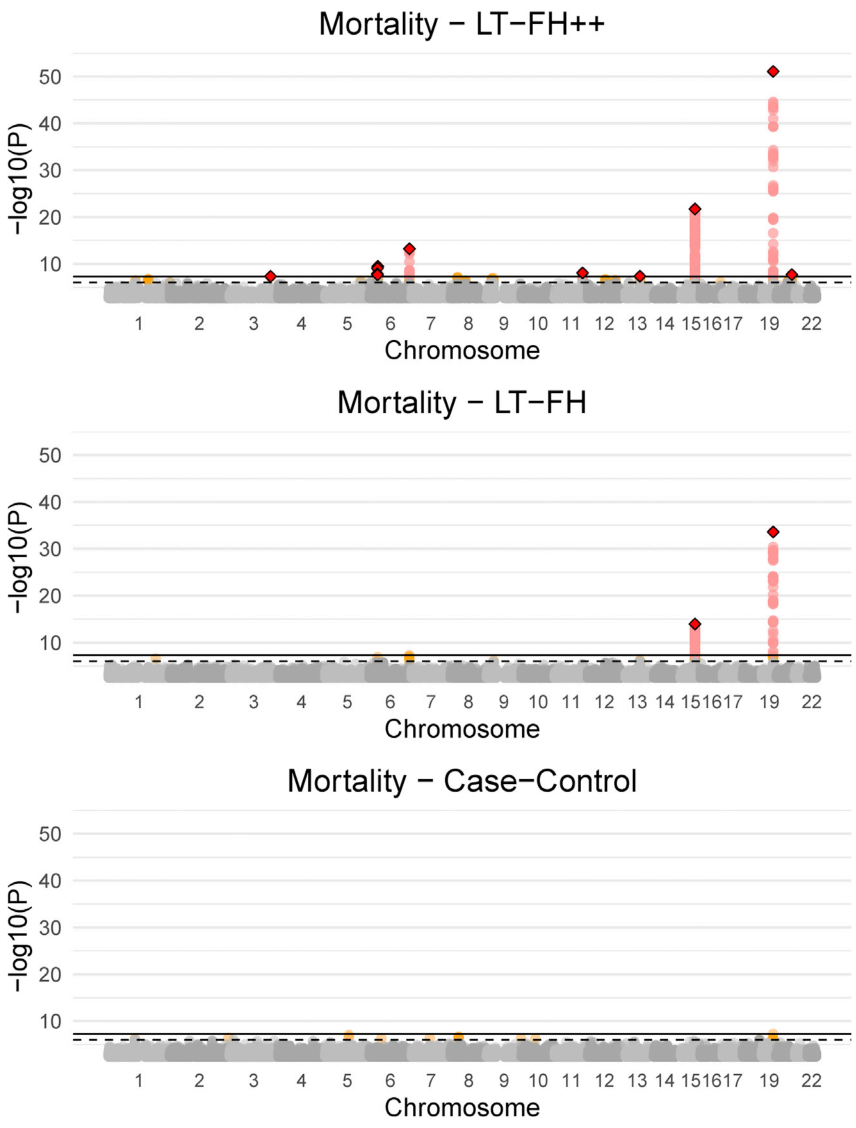

Figure 3. Manhattan plots for LT-FH++, LT-FH, and case-control GWAS of mortality in the UK Biobank
The Manhattan plots display a Bonferroni-corrected significance level of 5×10^{-8} and a suggestive threshold of $5\times\ 10^{-6}$. The genome-wide significant SNPs are colored in red. The diamonds correspond to top SNPs in a window of size 300,000 base pairs.

size, completeness of family history, and age-of-onset information. As expected, the power decreased for both LT-FH++ and LT-FH when family information was missing. However, the relative power gain of LT-FH++ over LT-FH increased when family information was missing or when the cases were ascertained. In short, one can expect to gain the most when the in-sample prevalence is high either among participants or in the family history.

Lastly, we have performed simulations for computation time. The results are shown in Figures S2 and S3 and the numbers are available in Table S13. In short, LT-FH++ scales linearly with sample size, and using 32 cores, it can estimate posterior genetic liabilities for 350,000 individuals in less than 25 min. All computation time simulations were performed on genomeDK.

Analysis of mortality in the UK Biobank

To evaluate the performance of LT-FH++ on real data, we chose mortality in the UK Biobank, as this is the only outcome available where we have age information for family members, i.e., we have age or age of death for mothers and fathers. We then obtained population prevalence information from the Office for National Statistics (ONS), which provides mortality rates for England and Wales by sex and birth year (since 1841), and for the UK since 1950. This allowed us to obtain individualized prevalence thresholds for LT-FH++ for each genotyped individual and their parents (see material and methods for details). The mortality rates by age and sex are shown for each decade in Figure S21.

The Manhattan plots for standard case-control, LT-FH, and LT-FH++ GWASs can be found in Figure 3 (see material and methods for analysis details). When using the case-control phenotype as the outcome in GWASs, we did not observe any genome-wide significant SNPs. For LT-FH, we found two genome-wide significant associations, including a well-known association with mortality in *APOE* (MIM: 107741)[60] and in *HYKK* (MIM: 614681), which is strongly associated with smoking behavior.[6] These were also the two strongest associations found with LT-FH++, which additionally found eight other independent associated variants, where independence was assessed with GCTA-COJO.[61] The ten identified variants are shown in Table S10, of which three variants have not previously been identified as associated with mortality or aging. One of these is near *HLA-B* (MIM: 142830), which is involved in immune response and has been found to be associated with white blood cell count[62] and Psoriasis.[63] The second association is near *MYCBP2* (MIM: 610392), which has previously been identified as being associated with chronotype,[64] and the expression of this gene was recently found to increase with age and interact with the SARS-CoV-2 proteome.[65] The third association was near *ZBBX* (MIM: 609118), which has been found to be associated with changes in DNA methylation with age.[66]

Because we do not know the true causal variants for mortality, we cannot accurately estimate power. Power has a formal statistical definition that requires us to know

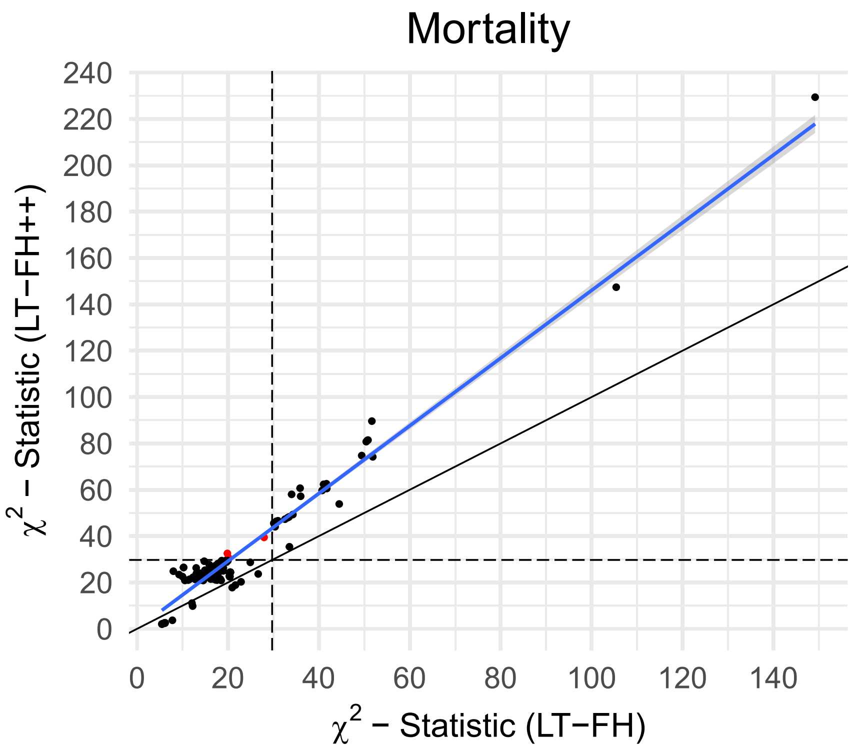

Figure 4. The X^2statistics for LT-FH++ versus the ones for LT-FH for the GWAS of mortality in the UK Biobank
We restricted to variants with a p value below 5×10^{-6} for at least one of the three compared outcomes. The common set of variants were LD clumped (prioritizing on minor allele frequencies) in an attempt to not bias one outcome over another. The red dots are variants identified as genome-wide significant for only one of the outcomes. The black dots are suggestive associations identified by either method, or genome-wide significant associations for both methods. The black line indicates the identity line and the blue line is the best fitted line via linear regression. The black dashed lines correspond to the threshold for genome-wide significance.

whether a SNP is causal or not. However, to approximate relative power gain (between methods) we considered a set of LD-pruned variants with a p value below 5×10^{-6} for at least one of the three compared outcomes. Assuming that these are enriched to be causal variants (or in strong linkage with causal variants), and that their null test statistics have similar inflation, then one can approximate relative power gain. We measure the increase in effective sample size by comparing *Z* scores from both methods. We then refer to this increase in effective sample size as an increase in power because power increases with sample size. We also note that the GWAS Q-Q plots for mortality for all methods (Figures S24–S26) showed no sign of test statistics' being inflated, suggesting that false-positive rates are similar across all methods. For LT-FH++, it leads to an estimated power increase of 42% over LT-FH. Because the *Z* scores squared are the χ^2 statistics, we opted to illustrate the power improvement of LT-FH++ over LT-FH through the χ^2 statistics. We plotted the χ^2 statistic for variants with a p value below 5×10^{-6} in Figure 4. LT-FH and LT-FH++ both had a large increase in power over case-control status, resulting in an estimated relative power increase of 110% and 187%, respectively. The χ^2 statistics and *Z* scores plots compared to case-control status can be found in Figures S22 and S23.

Application to four psychiatric disorders in iPSYCH

The iPSYCH data[33] with linked Danish registers has age and age-of-onset information for all close family members of genotyped individuals. We considered four psychiatric disorders in the iPSYCH data: ADHD, autism, depression, and schizophrenia. For each of these, we obtained prevalences by birth year, age, and sex by using the same diagnostic criteria (see material and methods for details). As shown in Figures S27, S32, S38, and S44 the prevalence of psychiatric disorders strongly depends on birth year and sex, making it an appealing application of LT-FH++. We performed a GWAS of the three outcomes, case-control GWAS, LT-FH, and LT-FH++, for the four psychiatric disorders (see material and methods for analysis details). Across the four psychiatric disorders, we found ten genome-wide significant associations by using LT-FH++ compared to eight by using both LT-FH and case-control. Specifically for ADHD, LT-FH++ found seven significant associations, while case-control status and LT-FH found five. All three outcomes identified the same five variants, and LT-FH++ identified two additional variants for ADHD. One of these variants was on chromosome 11 near *LINC02758* (MIM: 618711), which was found to be associated with ADHD in a meta-analysis,[10] and the other one was on chromosome 14 in *AKAP6* (MIM: 604691), which has previously been identified as being associated with cognitive traits.[67,68] The Manhattan plots for ADHD can be seen in Figure 5 for all three outcomes, i.e., case-control, LT-FH, and LT-FH++ (see material and methods for details). Manhattan plots for all three outcomes are very similar and no one outcome clearly outperforms the others. However, LT-FH++ does have two associations that were close to genome-wide significance with both LT-FH and case-control analysis but did not pass the significance threshold. Similarly, LT-FH++ and case-control have one SNP that is not found by LT-FH, but it is also close to the genome-wide significance threshold for LT-FH. In Figure 6, we show the χ^2 statistics plot restricting to LD-clumped SNPs with a p value threshold of 5×10^{-6} for the index SNP and the clumped SNPs from the largest external meta-analyzed ADHD summary statistics (see material and methods for details). If one

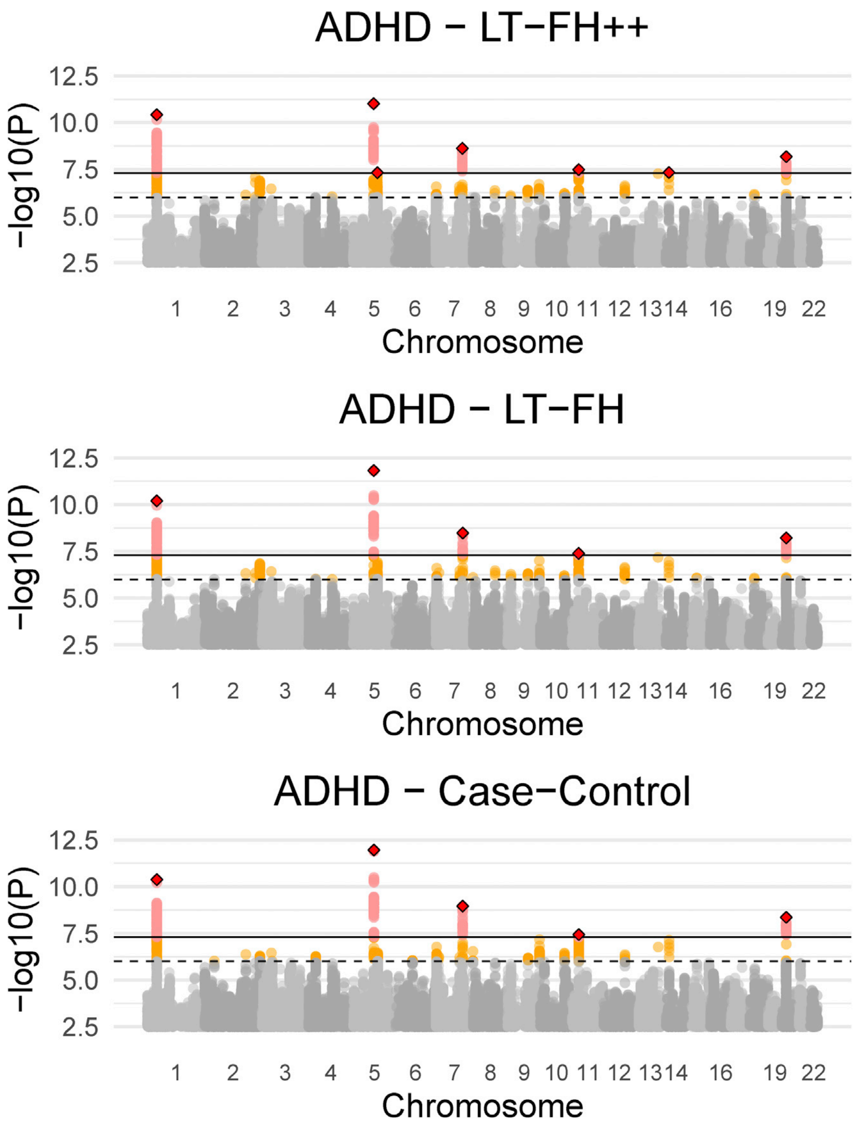

Figure 5. Manhattan plots for LT-FH++, LT-FH, and case-control GWAS of ADHD in the iPSYCH data
The dashed line indicates a suggestive p value of 5×10^{-6} and the fully drawn line at 5×10^{-8} indicates genome-wide significance threshold. The genome-wide significant SNPs are colored in red. The diamonds correspond to top SNPs in a window of size 300,000 base pairs.

method had clearly performed better than another, we would have expected to see a slope different from one, however this is not the case here. Overall, there is little power improvement by using either LT-FH or LT-FH++ over case-control GWAS for ADHD.

We performed a similar analysis for the three other iPSYCH disorders analyzed, namely ASD, depression, and schizophrenia. The Manhattan, QQ, Z scores, and χ^2 statistics plots can be found in Figures S28–S31, S33–S37, S39–S43, and S45–S49 for all iPSYCH analysis. For depression and schizophrenia, we found no genome-wide significant hits for any method used and the Z scores and χ^2 statistics indicate no difference in power between standard GWAS, LT-FH, and LT-FH++. For autism, we do see genome-wide significant hits: three for case-control GWAS and LT-FH++ and four for LT-FH. The SNP that is unique to LT-FH is also highly suggestive for case-control GWAS and LT-FH++. A table containing the COJO-independent SNPs can be found in Tables S11 and S12 for ADHD and ASD.

Discussion

Several large genetic datasets with linked electronic health registries (EHRs) have emerged in recent years, e.g., the UK Biobank data,[44] the iPSYCH data,[33] FinnGen, deCODE, and many more. As more genetic data is linked to EHRs, it is essential to develop statistical methods that make best use of all this information to decipher the genetics of common diseases. Here, we present a new and scalable method LT-FH++ for improving power in GWASs when family history and an age-of-onset distribution is available, which is typically the case in EHRs. We demonstrated the feasibility and relevance of the approach by using both simulations and real data applications. Using simulated case-control outcomes with a prevalence of 5% and 10%, we observed power gains of up to 18% compared to LT-FH and up to 61% compared to with standard case-control status. We found that LT-FH++ provided the largest relative improvements when cases were ascertained (such that in-sample case-control ratio becomes larger than prevalence) and when prevalence was high. As age-of-onset information allows us to estimate individual liabilities for cases, it makes sense that the largest relative power gains for LT-FH++ are observed when the sample prevalence is high or when the prevalence in the family history is high. Furthermore, LT-FH++ can be applied to individuals with partial or missing family information, as well as individuals for which age and age-of-onset information was missing.

We acknowledge that not everyone has access to the same level of detailed health register data (e.g., Danish registers) or other electronic health records. Therefore, we would like to point out that it is not a requirement to estimate prevalence curves in the population that you are performing the analysis in. In some instances, prevalence rates can be found in publications or from public sites such as statistikbanken or the Office for National Statistics (UK). In practice, prevalence rates may have to be

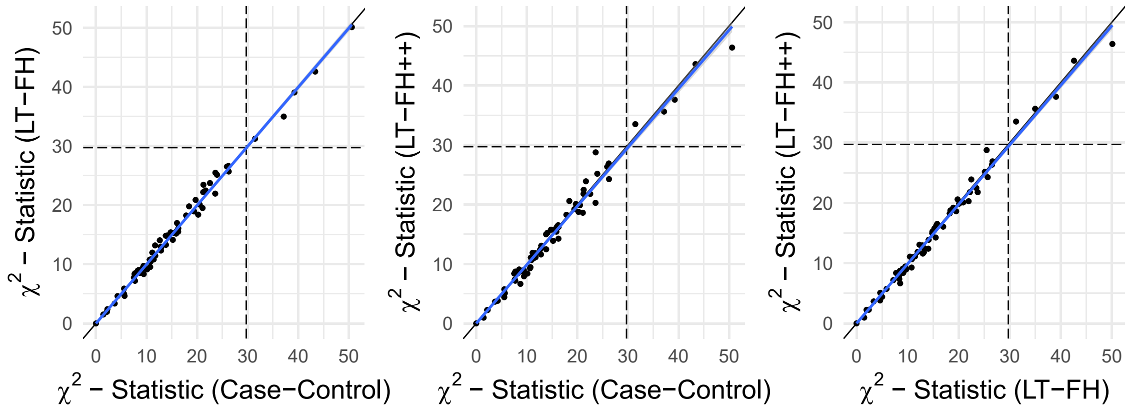

Figure 6. The X^2 statistics from the GWAS of ADHD for each of the three methods (LT-FH++, LT-FH, and case-control GWAS) plotted against each other
The dots correspond to LD-clumped SNPs that have a p value below 5×10^{-6} in the largest published meta-analysis and present in the iPSYCH cohort (see material and methods for details). The blue line indicates the linear regression line between two methods and the black line indicates the identity line. The slopes of the regression lines are not significantly different from one for any pair of methods.

approximated with external populations and subsequently used to assign the personalized thresholds in the internal population provided information such as sex, age of onset, and birth year is available in the internal and external data.

We applied LT-FH++ to study mortality in UK Biobank and four common psychiatric disorders in iPSYCH, all prevalent outcomes for which we had both family history available as well as age-of-onset distributions. This includes age, age of onset (for cases), cohort effects, and sex for both the genotyped individuals and family members. We also had access to public data for mortality incidence rates by age, sex, and birth year for England and Wales from 1840s to the present day. We compiled similar information for the four psychiatric disorders by the full Danish register data (see material and methods). For mortality in the UK Biobank data, we found ten independent associations when applying LT-FH++, compared to two with LT-FH and none with the case-control status. This result further underlines the importance of including other information in GWASs. The power increase of LT-FH over case-control status highlights the importance of family history, and the power increase of LT-FH++ over LT-FH highlights the importance of accounting for age of onset. The most significant association was found in *APOE*, which also harbored the only significant association in a recent survival model (frailty model) GWAS of mortality in the UK Biobank data.[27] Most of the identified associations were in or near well-known disease-related genes and were largely concordant with the genome-wide associations found by Pilling et al.[69] when performing a GWAS of combined mothers' and fathers' attained age.

We further applied LT-FH++ to the four common psychiatric disorders in the iPSYCH data. Combined, we found ten independent genome-wide significant associations with LT-FH++, compared to eight for LT-FH and case-control status. Compared to mortality, the observed power gain for the iPSYCH disorders was small despite having access to more information per individual. The discrepancy in performance when applied to the mortality in the UK Biobank and four common psychiatric disorders may have several reasons. First, case-control, LT-FH, and LT-FH++ performed similarly for each of the four common psychiatric disorders, and in the simulations, we saw a relative power increase when cases were ascertained through downsampling of controls; however, due to the lower overall sample size, the absolute power to detect causal SNPs also decreased significantly with sample size. We suspect a similar situation might be happening in the iPSYCH data. Second, because simulations showed the power improvement was larger when prevalence was higher and cases were ascertained, the difference may be explained by the prevalence differences. Death is a guarantee, while psychiatric disorders are not. Prevalence rates were far lower for the psychiatric disorders compared to mortality (see Tables 1 and 2), suggesting that less could be gained by accounting for family history and age of onset. Third, it is possible that the multivariate liability threshold model (underlying LT-FH and LT-FH++) may better fit mortality than psychiatric disorders. More specifically, the model makes several key assumptions. First, both LT-FH and LT-FH++ assumes that the heritability is known and that there is no environmental covariance between family members. In practice, one can often estimate the heritability in the sample or rely on published estimates. Second, it assumes that the population disease prevalence is known and (if relevant) provided for subgroups defined by age, birth year, and sex. However, simulations using LT-FH and LT-FH++ indicate that it is relatively robust to misspecification of these parameters.[32] Third, the model assumes that the genetic architecture of the disease or trait in question does not vary by age of diagnosis, birth year, or differ between sexes. Some research suggests that this assumption is reasonable for many outcomes, including the four psychiatric disorders analyzed here,[70,71] but these will generally not hold in practice. We note that case-control GWASs also assume this unless the analysis is stratified by these

subgroups. Fourth, LT-FH++ assumes that the threshold always decreases with age. The intuition behind this is that the disease prevalence is the cumulative incidence, which by definition always increases with age, and the threshold is the upper quantile of the inverse standard normal at the age-specific prevalence. An individual then only becomes a case if their liability becomes larger than the prevalence threshold, as it decreases with time. A consequence of this assumption is that early-onset cases generally have higher disease liabilities than late-onset cases, which is also the expectation in survival model analysis if the hazard rate is (positively) correlated with the genetic risk. The correlation between genetic risk and earlier age of onset has been observed for several common diseases, e.g., Alzheimer disease (MIM: 104300),[72] coronary artery disease (MIM: 608320), and prostate cancer (MIM: 176807).[73] However, if the age of onset for a given disease is not heritable, or if the genetic correlation between the age of onset and disease outcome is weak, then we do not expect LT-FH++ to improve statistical power for identifying genetic variants. Indeed, this might be one possible explanation for why we do not observe improvements in power when applying LT-FH++ to iPSYCH data, although we note that polygenic risk scores have been found to contribute to hazard rates for psychiatric disorders in the iPSYCH data.[74,75]

Conceptually, LT-FH++ combines two methods into one to improve power in genetic analyses, namely LT-FH, which is based on the liability threshold model and incorporates family history, and survival analysis, which can account for age and changes in prevalence over time and is routinely used to model time-to-event data. With family history and age-of-onset information available, we believe LT-FH++ will be an attractive method for improving power in many different genetic analyses, including GWASs and heritability analyses and for polygenic risk scores.[76–78] As more genetic datasets with linked health records and family information become available, e.g., in large national biobank projects, we expect the value of statistical methods that can efficiently distill family history and individual health information into biological insight will only increase.

Data and code availability

iPSYCH is approved by the Danish Scientific Ethics Committee, the Danish Health Data Authority, the Danish Data Protection Agency, Statistics Denmark, and the Danish Neonatal Screening Biobank Steering Committee.[33] UK Biobank received ethical approval from the NHS National Research Ethics Service North West (11/NW/0382). The present analyses were conducted under UK Biobank data application number 58024. The code used for LT-FH++ has been implemented into an R package, and it is available at https://github.com/EmilMiP/LTFHPlus. We have also reimplemented LT-FH in the package, where we utilize the Gibbs sampler to efficiently estimate the genetic liabilities, keeping the same input format as the original implementation. Summary statistics can be downloaded from https://drive.google.com/drive/folders/13Tryy7KuoXkKUSuYu4CI0nnt6WOTLniD and mortality rates were found on https://www.ons.gov.uk/.

Supplemental information

Supplemental information can be found online at https://doi.org/10.1016/j.ajhg.2022.01.009.

Acknowledgments

We would like to thank Margaux Hujoel for useful discussions and allowing us to use the LT-FH++ name. We would like to thank Mark Daly for useful advice and helpful comments. F.P. and B.J.V. were supported by the Danish National Research Foundation (Niels Bohr Professorship to Prof. John McGrath). We also acknowledge the Lundbeck Foundation Initiative for Integrative Psychiatric Research, iPSYCH (R102-A9118, R155-2014-1724, and R248-2017-2003). B.J.V. was also supported by a Lundbeck Foundation fellowship (R335-2019-2339). High-performance computer capacity for handling and statistical analysis of iPSYCH data on the GenomeDK HPC facility was provided by the Center for Genomics and Personalized Medicine and the Centre for Integrative Sequencing, iSEQ, Aarhus University, Denmark(grant to A.D.B.). This research has been conducted with the UK Biobank Resource under application number 58024.

Declaration of interests

J.C. has received honoraria for serving on the Scientific Advisory Board of Union Chimique Belge (UCB) Nordic and Eisai AB and for giving lectures for UCB Nordic and Eisai as well as travel funds from UCB Nordic and funding by the Novo Nordisk Foundation (grant number: NNF16OC0019126), the Central Denmark Region, and the Danish Epilepsy Association.

Received: July 16, 2021
Accepted: January 7, 2022
Published: February 8, 2022

Web resources

GenomeDK, https://genome.au.dk/
LTFHPlus, https://github.com/EmilMiP/LTFHPlus
Office of National Statistics, https://www.ons.gov.uk/
Statistikbanken, https://www.statistikbanken.dk/

References

1. Nielsen, J.B., Thorolfsdottir, R.B., Fritsche, L.G., Zhou, W., Skov, M.W., Graham, S.E., Herron, T.J., McCarthy, S., Schmidt, E.M., Sveinbjornsson, G., et al. (2018). Biobank-driven genomic discovery yields new insight into atrial fibrillation biology. Nat. Genet. *50*, 1234–1239.
2. Wuttke, M., Li, Y., Li, M., Sieber, K.B., Feitosa, M.F., Gorski, M., Tin, A., Wang, L., Chu, A.Y., Hoppmann, A., et al. (2019). A catalog of genetic loci associated with kidney function from analyses of a million individuals. Nat. Genet. *51*, 957–972.
3. Mahajan, A., Taliun, D., Thurner, M., Robertson, N.R., Torres, J.M., Rayner, N.W., Payne, A.J., Steinthorsdottir, V., Scott, R.A., Grarup, N., et al. (2018). Fine-mapping type 2 diabetes loci to single-variant resolution using high-density imputation and islet-specific epigenome maps. Nat. Genet. *50*, 1505–1513.
4. Siewert, K.M., and Voight, B.F. (2018). Bivariate Genome-Wide Association Scan Identifies 6 Novel Loci Associated With Lipid

Levels and Coronary Artery Disease. Circ Genom Precis Med *11*, e002239.

5. Nalls, M.A., Blauwendraat, C., Vallerga, C.L., Heilbron, K., Bandres-Ciga, S., Chang, D., Tan, M., Kia, D.A., Noyce, A.J., Xue, A., et al. (2019). Expanding Parkinson's disease genetics: novel risk loci, genomic context, causal insights and heritable risk. bioRxiv. https://doi.org/10.1101/388165.
6. Liu, M., Jiang, Y., Wedow, R., Li, Y., Brazel, D.M., Chen, F., Datta, G., Davila-Velderrain, J., McGuire, D., Tian, C., et al. (2019). Association studies of up to 1.2 million individuals yield new insights into the genetic etiology of tobacco and alcohol use. Nat. Genet. *51*, 237–244.
7. Jansen, I.E., Savage, J.E., Watanabe, K., Bryois, J., Williams, D.M., Steinberg, S., Sealock, J., Karlsson, I.K., Hägg, S., Athanasiu, L., et al. (2019). Genome-wide meta-analysis identifies new loci and functional pathways influencing Alzheimer's disease risk. Nat. Genet. *51*, 404–413.
8. Schizophrenia Working Group of the Psychiatric Genomics Consortium (2014). Biological insights from 108 schizophrenia-associated genetic loci. Nature *511*, 421–427.
9. Grove, J., Ripke, S., Als, T.D., Mattheisen, M., Walters, R.K., Won, H., Pallesen, J., Agerbo, E., Andreassen, O.A., Anney, R., et al. (2019). Identification of common genetic risk variants for autism spectrum disorder. Nat. Genet. *51*, 431–444.
10. Demontis, D., Walters, R.K., Martin, J., Mattheisen, M., Als, T.D., Agerbo, E., Baldursson, G., Belliveau, R., Bybjerg-Grauholm, J., Bækvad-Hansen, M., et al. (2019). Discovery of the first genome-wide significant risk loci for attention deficit/hyperactivity disorder. Nat. Genet. *51*, 63–75.
11. Stahl, E.A., Breen, G., Forstner, A.J., McQuillin, A., Ripke, S., Trubetskoy, V., Mattheisen, M., Wang, Y., Coleman, J.R.I., Gaspar, H.A., et al. (2019). Genome-wide association study identifies 30 loci associated with bipolar disorder. Nat. Genet. *51*, 793–803.
12. Howard, D.M., Adams, M.J., Clarke, T.-K., Hafferty, J.D., Gibson, J., Shirali, M., Coleman, J.R.I., Hagenaars, S.P., Ward, J., Wigmore, E.M., et al. (2019). Genome-wide meta-analysis of depression identifies 102 independent variants and highlights the importance of the prefrontal brain regions. Nat. Neurosci. *22*, 343–352.
13. Loh, P.-R., Bhatia, G., Gusev, A., Finucane, H.K., Bulik-Sullivan, B.K., Pollack, S.J., de Candia, T.R., Lee, S.H., Wray, N.R., Kendler, K.S., et al. (2015). Contrasting genetic architectures of schizophrenia and other complex diseases using fast variance-components analysis. Nat. Genet. *47*, 1385–1392.
14. Loh, P.-R., Kichaev, G., Gazal, S., Schoech, A.P., and Price, A.L. (2018). Mixed-model association for biobank-scale datasets. Nat. Genet. *50*, 906–908.
15. Privé, F., Aschard, H., Ziyatdinov, A., and Blum, M.G.B. (2018). Efficient analysis of large-scale genome-wide data with two R packages: bigstatsr and bigsnpr. Bioinformatics *34*, 2781–2787.
16. Jiang, L., Zheng, Z., Qi, T., Kemper, K.E., Wray, N.R., Visscher, P.M., and Yang, J. (2019). A resource-efficient tool for mixed model association analysis of large-scale data. Nat. Genet. *51*, 1749–1755.
17. Zhou, W., Nielsen, J.B., Fritsche, L.G., Dey, R., Gabrielsen, M.E., Wolford, B.N., LeFaive, J., VandeHaar, P., Gagliano, S.A., Gifford, A., et al. (2018). Efficiently controlling for case-control imbalance and sample relatedness in large-scale genetic association studies. Nat. Genet. *50*, 1335–1341.
18. Visscher, P.M., Wray, N.R., Zhang, Q., Sklar, P., McCarthy, M.I., Brown, M.A., and Yang, J. (2017). 10 Years of GWAS Discovery: Biology, Function, and Translation. Am. J. Hum. Genet. *101*, 5–22.
19. Tam, V., Patel, N., Turcotte, M., Bossé, Y., Paré, G., and Meyre, D. (2019). Benefits and limitations of genome-wide association studies. Nat. Rev. Genet. *20*, 467–484.
20. Ferreira, M.A.R., Vonk, J.M., Baurecht, H., Marenholz, I., Tian, C., Hoffman, J.D., Helmer, Q., Tillander, A., Ullemar, V., Lu, Y., et al. (2020). Age-of-onset information helps identify 76 genetic variants associated with allergic disease. PLoS Genet. *16*, e1008725.
21. Korte, A., Vilhjálmsson, B.J., Segura, V., Platt, A., Long, Q., and Nordborg, M. (2012). A mixed-model approach for genome-wide association studies of correlated traits in structured populations. Nat. Genet. *44*, 1066–1071.
22. Dahl, A., Iotchkova, V., Baud, A., Johansson, Å., Gyllensten, U., Soranzo, N., Mott, R., Kranis, A., and Marchini, J. (2016). A multiple-phenotype imputation method for genetic studies. Nat. Genet. *48*, 466–472.
23. Aschard, H., Guillemot, V., Vilhjalmsson, B., Patel, C.J., Skurnik, D., Ye, C.J., Wolpin, B., Kraft, P., and Zaitlen, N. (2017). Covariate selection for association screening in multiphenotype genetic studies. Nat. Genet. *49*, 1789–1795.
24. Turley, P., Walters, R.K., Maghzian, O., Okbay, A., Lee, J.J., Fontana, M.A., Nguyen-Viet, T.A., Wedow, R., Zacher, M., Furlotte, N.A., et al. (2018). Multi-trait analysis of genome-wide association summary statistics using MTAG. Nat. Genet. *50*, 229–237.
25. Julienne, H., Laville, V., McCaw, Z.R., He, Z., Guillemot, V., Lasry, C., Ziyatdinov, A., Vaysse, A., Lechat, P., Ménager, H., et al. (2020). Multitrait genetic-phenotype associations to connect disease variants and biological mechanisms. bioRxiv. https://doi.org/10.1101/2020.06.26.172999.
26. Hughey, J.J., Rhoades, S.D., Fu, D.Y., Bastarache, L., Denny, J.C., and Chen, Q. (2019). Cox regression increases power to detect genotype-phenotype associations in genomic studies using the electronic health record. BMC Genomics *20*, 805.
27. Dey, R., Zhou, W., Kiiskinen, T., Havulinna, A., Elliott, A., Karjalainen, J., Kurki, M., Qin, A., Lee, S., Palotie, A., et al. (2020). An efficient and accurate frailty model approach for genome-wide survival association analysis controlling for population structure and relatedness in large-scale biobanks. bioRxiv. https://doi.org/10.1101/2020.10.31.358234.
28. He, L., and Kulminski, A.M. (2020). Fast Algorithms for Conducting Large-Scale GWAS of Age-at-Onset Traits Using Cox Mixed-Effects Models. Genetics *215*, 41–58.
29. Bi, W., Fritsche, L.G., Mukherjee, B., Kim, S., and Lee, S. (2020). A Fast and Accurate Method for Genome-Wide Time-to-Event Data Analysis and Its Application to UK Biobank. Am. J. Hum. Genet. *107*, 222–233.
30. Liu, J.Z., Erlich, Y., and Pickrell, J.K. (2017). Case-control association mapping by proxy using family history of disease. Nat. Genet. *49*, 325–331.
31. Marioni, R.E., Harris, S.E., Zhang, Q., McRae, A.F., Hagenaars, S.P., Hill, W.D., Davies, G., Ritchie, C.W., Gale, C.R., Starr, J.M., et al. (2018). GWAS on family history of Alzheimer's disease. Transl. Psychiatry *8*, 99.
32. Hujoel, M.L.A., Gazal, S., Loh, P.-R., Patterson, N., and Price, A.L. (2020). Liability threshold modeling of case-control status and family history of disease increases association power. Nat. Genet. *52*, 541–547.

33. Pedersen, C.B., Bybjerg-Grauholm, J., Pedersen, M.G., Grove, J., Agerbo, E., Bækvad-Hansen, M., Poulsen, J.B., Hansen, C.S., McGrath, J.J., Als, T.D., et al. (2018). The iPSYCH2012 case-cohort sample: new directions for unravelling genetic and environmental architectures of severe mental disorders. Mol. Psychiatry *23*, 6–14.
34. Cox, D.R., and Oakes, D. (1984). Analysis of Survival Data (CRC Press).
35. Ojavee, S.E., Kousathanas, A., Trejo Banos, D., Orliac, E.J., Patxot, M., Läll, K., Mägi, R., Fischer, K., Kutalik, Z., and Robinson, M.R. (2021). Genomic architecture and prediction of censored time-to-event phenotypes with a Bayesian genome-wide analysis. Nat. Commun. *12*, 2337.
36. Li, R., Chang, C., Justesen, J.M., Tanigawa, Y., Qiang, J., Hastie, T., Rivas, M.A., and Tibshirani, R. (2020). Fast Lasso method for large-scale and ultrahigh-dimensional Cox model with applications to UK Biobank. Biostatistics, kxaa038.
37. Eddelbuettel, D., and Francois, R. (2011). Rcpp: Seamless R and C++ Integration. J. Stat. Softw. *40*, 1–18.
38. Loh, P.-R., Tucker, G., Bulik-Sullivan, B.K., Vilhjálmsson, B.J., Finucane, H.K., Salem, R.M., Chasman, D.I., Ridker, P.M., Neale, B.M., Berger, B., et al. (2015). Efficient Bayesian mixed-model analysis increases association power in large cohorts. Nat. Genet. *47*, 284–290.
39. Kragh Andersen, P., Pohar Perme, M., van Houwelingen, H.C., Cook, R.J., Joly, P., Martinussen, T., Taylor, J.M.G., Abrahamowicz, M., and Therneau, T.M. (2021). Analysis of time-to-event for observational studies: Guidance to the use of intensity models. Stat. Med. *40*, 185–211.
40. Wilhelm, S. (2015). Gibbs sampler for the truncated multivariate normal distribution. https://cran.r-project.org/web/packages/tmvtnorm/vignettes/GibbsSampler.pdf.
41. Pedersen, C.B. (2011). The Danish Civil Registration System. Scand. J. Public Health *39* (7, Suppl), 22–25.
42. Mors, O., Perto, G.P., and Mortensen, P.B. (2011). The Danish Psychiatric Central Research Register. Scand. J. Public Health *39* (7, Suppl), 54–57.
43. Hansen, S.N., Overgaard, M., Andersen, P.K., and Parner, E.T. (2017). Estimating a population cumulative incidence under calendar time trends. BMC Med. Res. Methodol. *17*, 7.
44. Bycroft, C., Freeman, C., Petkova, D., Band, G., Elliott, L.T., Sharp, K., Motyer, A., Vukcevic, D., Delaneau, O., O'Connell, J., et al. (2018). The UK Biobank resource with deep phenotyping and genomic data. Nature *562*, 203–209.
45. Bybjerg-Grauholm, J., Pedersen, C.B., Bækvad-Hansen, M., Pedersen, M.G., Adamsen, D., Hansen, C.S., Agerbo, E., Grove, J., Als, T.D., Schork, A.J., et al. (2020). The iPSYCH2015 Case-Cohort sample: updated directions for unravelling genetic and environmental architectures of severe mental disorders. medRxiv. https://doi.org/10.1101/2020.11.30.20237768.
46. Lam, M., Awasthi, S., Watson, H.J., Goldstein, J., Panagiotaropoulou, G., Trubetskoy, V., Karlsson, R., Frei, O., Fan, C.C., De Witte, W., et al. (2020). RICOPILI: Rapid Imputation for COnsortias PIpeLIne. Bioinformatics *36*, 930–933.
47. Privé, F., Luu, K., Blum, M.G.B., McGrath, J.J., and Vilhjálmsson, B.J. (2020). Efficient toolkit implementing best practices for principal component analysis of population genetic data. Bioinformatics *36*, 4449–4457.
48. Brikell, I., Kuja-Halkola, R., and Larsson, H. (2015). Heritability of attention-deficit hyperactivity disorder in adults. Am. J. Med. Genet. B. Neuropsychiatr. Genet. *168*, 406–413.
49. Sandin, S., Lichtenstein, P., Kuja-Halkola, R., Hultman, C., Larsson, H., and Reichenberg, A. (2017). The Heritability of Autism Spectrum Disorder. JAMA *318*, 1182–1184.
50. Fernandez-Pujals, A.M., Adams, M.J., Thomson, P., McKechanie, A.G., Blackwood, D.H., Smith, B.H., Dominiczak, A.F., Morris, A.D., Matthews, K., Campbell, A., et al. (2015). Epidemiology and Heritability of Major Depressive Disorder, Stratified by Age of Onset, Sex, and Illness Course in Generation Scotland: Scottish Family Health Study (GS:SFHS). PLoS ONE *10*, e0142197.
51. Hilker, R., Helenius, D., Fagerlund, B., Skytthe, A., Christensen, K., Werge, T.M., Nordentoft, M., and Glenthøj, B. (2018). Heritability of Schizophrenia and Schizophrenia Spectrum Based on the Nationwide Danish Twin Register. Biol. Psychiatry *83*, 492–498.
52. Wray, N.R., Ripke, S., Mattheisen, M., Trzaskowski, M., Byrne, E.M., Abdellaoui, A., Adams, M.J., Agerbo, E., Air, T.M., Andlauer, T.M.F., et al. (2018). Genome-wide association analyses identify 44 risk variants and refine the genetic architecture of major depression. Nat. Genet. *50*, 668–681.
53. Falconer, D.S. (1965). The inheritance of liability to certain diseases, estimated from the incidence among relatives. Ann. Hum. Genet. *29*, 51–76.
54. Fisher, R.A. (1918). The correlation between relatives on the supposition of mendelian inheritance. Trans. R. Soc. Edinb. *52*, 899, 438.
55. Lee, S.H., Wray, N.R., Goddard, M.E., and Visscher, P.M. (2011). Estimating missing heritability for disease from genome-wide association studies. Am. J. Hum. Genet. *88*, 294–305.
56. Thygesen, L.C., Daasnes, C., Thaulow, I., and Brønnum-Hansen, H. (2011). Introduction to Danish (nationwide) registers on health and social issues: structure, access, legislation, and archiving. Scand. J. Public Health *39* (7, Suppl), 12–16.
57. Hayeck, T.J., Loh, P.-R., Pollack, S., Gusev, A., Patterson, N., Zaitlen, N.A., and Price, A.L. (2017). Mixed Model Association with Family-Biased Case-Control Ascertainment. Am. J. Hum. Genet. *100*, 31–39.
58. Hayeck, T.J., Zaitlen, N.A., Loh, P.-R., Vilhjalmsson, B., Pollack, S., Gusev, A., Yang, J., Chen, G.-B., Goddard, M.E., Visscher, P.M., et al. (2015). Mixed model with correction for case-control ascertainment increases association power. Am. J. Hum. Genet. *96*, 720–730.
59. So, H.-C., and Sham, P.C. (2010). A unifying framework for evaluating the predictive power of genetic variants based on the level of heritability explained. PLoS Genet. *6*, e1001230.
60. Schächter, F., Faure-Delanef, L., Guénot, F., Rouger, H., Froguel, P., Lesueur-Ginot, L., and Cohen, D. (1994). Genetic associations with human longevity at the APOE and ACE loci. Nat. Genet. *6*, 29–32.
61. Yang, J., Ferreira, T., Morris, A.P., Medland, S.E., Madden, P.A.F., Heath, A.C., Martin, N.G., Montgomery, G.W., Weedon, M.N., Loos, R.J., et al. (2012). Conditional and joint multiple-SNP analysis of GWAS summary statistics identifies additional variants influencing complex traits. Nat. Genet. *44*, 369–375, S1–S3.
62. Chen, M.-H., Raffield, L.M., Mousas, A., Sakaue, S., Huffman, J.E., Moscati, A., Trivedi, B., Jiang, T., Akbari, P., Vuckovic, D., et al. (2020). Trans-ethnic and Ancestry-Specific Blood-Cell Genetics in 746,667 Individuals from 5 Global Populations. Cell *182*, 1198–1213.e14.

63. Tsoi, L.C., Spain, S.L., Knight, J., Ellinghaus, E., Stuart, P.E., Capon, F., Ding, J., Li, Y., Tejasvi, T., Gudjonsson, J.E., et al. (2012). Identification of 15 new psoriasis susceptibility loci highlights the role of innate immunity. Nat. Genet. *44*, 1341–1348.
64. Jones, S.E., Lane, J.M., Wood, A.R., van Hees, V.T., Tyrrell, J., Beaumont, R.N., Jeffries, A.R., Dashti, H.S., Hillsdon, M., Ruth, K.S., et al. (2019). Genome-wide association analyses of chronotype in 697,828 individuals provides insights into circadian rhythms. Nat. Commun. *10*, 343.
65. Chow, R.D., Majety, M., and Chen, S. (2021). The aging transcriptome and cellular landscape of the human lung in relation to SARS-CoV-2. Nat. Commun. *12*, 4.
66. Zhang, Q., Marioni, R.E., Robinson, M.R., Higham, J., Sproul, D., Wray, N.R., Deary, I.J., McRae, A.F., and Visscher, P.M. (2018). Genotype effects contribute to variation in longitudinal methylome patterns in older people. Genome Med. *10*, 75.
67. Savage, J.E., Jansen, P.R., Stringer, S., Watanabe, K., Bryois, J., de Leeuw, C.A., Nagel, M., Awasthi, S., Barr, P.B., Coleman, J.R.I., et al. (2018). Genome-wide association meta-analysis in 269,867 individuals identifies new genetic and functional links to intelligence. Nat. Genet. *50*, 912–919.
68. Lee, J.J., Wedow, R., Okbay, A., Kong, E., Maghzian, O., Zacher, M., Nguyen-Viet, T.A., Bowers, P., Sidorenko, J., Karlsson Linnér, R., et al. (2018). Gene discovery and polygenic prediction from a genome-wide association study of educational attainment in 1.1 million individuals. Nat. Genet. *50*, 1112–1121.
69. Pilling, L.C., Kuo, C.-L., Sicinski, K., Tamosauskaite, J., Kuchel, G.A., Harries, L.W., Herd, P., Wallace, R., Ferrucci, L., and Melzer, D. (2017). Human longevity: 25 genetic loci associated in 389,166 UK biobank participants. Aging (Albany N.Y.) *9*, 2504–2520.
70. Martin, J., Khramtsova, E.A., Goleva, S.B., Blokland, G.A.M., Traglia, M., Walters, R.K., Hübel, C., Coleman, J.R.I., Breen, G., Børglum, A.D., et al. (2020). Examining sex-differentiated genetic effects across neuropsychiatric and behavioral traits. Biol. Psychiatry *89*, 1127–1137.
71. Traglia, M., Bseiso, D., Gusev, A., Adviento, B., Park, D.S., Mefford, J.A., Zaitlen, N., and Weiss, L.A. (2017). Genetic Mechanisms Leading to Sex Differences Across Common Diseases and Anthropometric Traits. Genetics *205*, 979–992.
72. Zhang, Q., Sidorenko, J., Couvy-Duchesne, B., Marioni, R.E., Wright, M.J., Goate, A.M., Marcora, E., Huang, K.-L., Porter, T., Laws, S.M., et al. (2020). Risk prediction of late-onset Alzheimer's disease implies an oligogenic architecture. Nat. Commun. *11*, 4799.
73. Mars, N., Koskela, J.T., Ripatti, P., Kiiskinen, T.T.J., Havulinna, A.S., Lindbohm, J.V., Ahola-Olli, A., Kurki, M., Karjalainen, J., Palta, P., et al. (2020). Polygenic and clinical risk scores and their impact on age at onset and prediction of cardiometabolic diseases and common cancers. Nat. Med. *26*, 549–557.
74. Musliner, K.L., Krebs, M.D., Albiñana, C., Vilhjalmsson, B., Agerbo, E., Zandi, P.P., Hougaard, D.M., Nordentoft, M., Børglum, A.D., Werge, T., et al. (2020). Polygenic Risk and Progression to Bipolar or Psychotic Disorders Among Individuals Diagnosed With Unipolar Depression in Early Life. Am. J. Psychiatry *177*, 936–943.
75. Agerbo, E., Trabjerg, B.B., Børglum, A.D., Schork, A.J., Vilhjálmsson, B.J., Pedersen, C.B., Hakulinen, C., Albiñana, C., Hougaard, D.M., Grove, J., et al. (2021). Risk of Early-Onset Depression Associated With Polygenic Liability, Parental Psychiatric History, and Socioeconomic Status. JAMA Psychiatry *78*, 387–397.
76. Agerbo, E., Sullivan, P.F., Vilhjálmsson, B.J., Pedersen, C.B., Mors, O., Børglum, A.D., Hougaard, D.M., Hollegaard, M.V., Meier, S., Mattheisen, M., et al. (2015). Polygenic Risk Score, Parental Socioeconomic Status, Family History of Psychiatric Disorders, and the Risk for Schizophrenia: A Danish Population-Based Study and Meta-analysis. JAMA Psychiatry *72*, 635–641.
77. Lencz, T., Backenroth, D., Green, A., Weissbrod, O., Zuk, O., and Carmi, S. (2020). Utility of polygenic embryo screening for disease depends on the selection strategy. bioRxiv. https://doi.org/10.1101/2020.11.05.370478.
78. Hujoel, M.L.A., Loh, P.-R., Neale, B.M., and Price, A.L. (2021). Incorporating family history of disease improves polygenic risk scores in diverse populations. bioRxiv. https://doi.org/10.1101/2021.04.15.439975.

The American Journal of Human Genetics, Volume *109*

Supplemental information

Accounting for age of onset and family history improves power in genome-wide association studies

Emil M. Pedersen, Esben Agerbo, Oleguer Plana-Ripoll, Jakob Grove, Julie W. Dreier, Katherine L. Musliner, Marie Bækvad-Hansen, Georgios Athanasiadis, Andrew Schork, Jonas Bybjerg-Grauholm, David M. Hougaard, Thomas Werge, Merete Nordentoft, Ole Mors, Søren Dalsgaard, Jakob Christensen, Anders D. Børglum, Preben B. Mortensen, John J. McGrath, Florian Privé, and Bjarni J. Vilhjálmsson

Supplemental Information

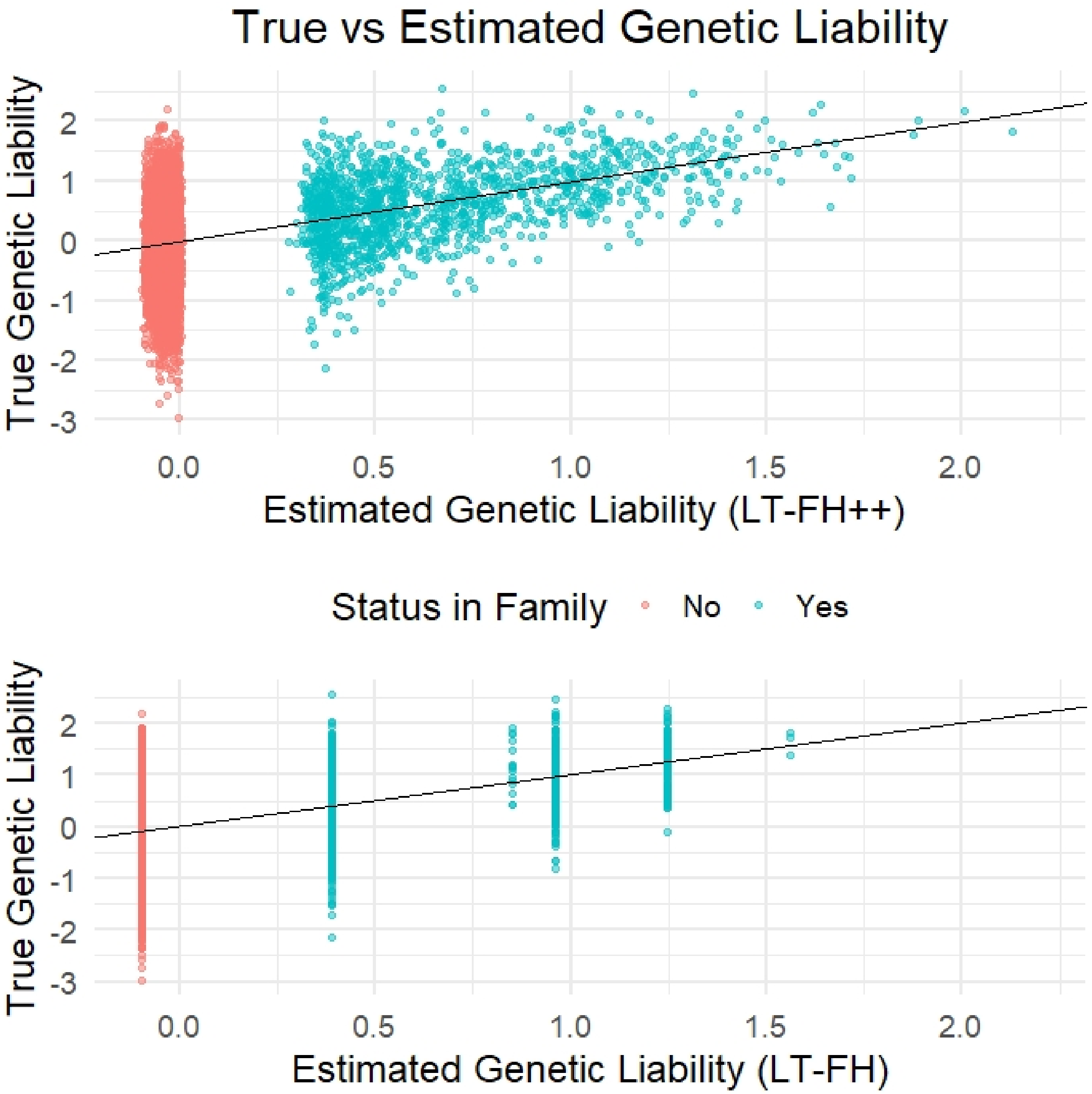

Figure S1: Simulated genetic liabilities assuming two parents and 0 siblings, a heritability of 50% and a prevalence of 10% (see Methods for details). We see that LT-FH estimates for the genetic liabilities fall into specific groups, depending on the case status of the individual and family

members. LT-FH++ takes age into account to obtain a more refined prediction of the genetic liability.

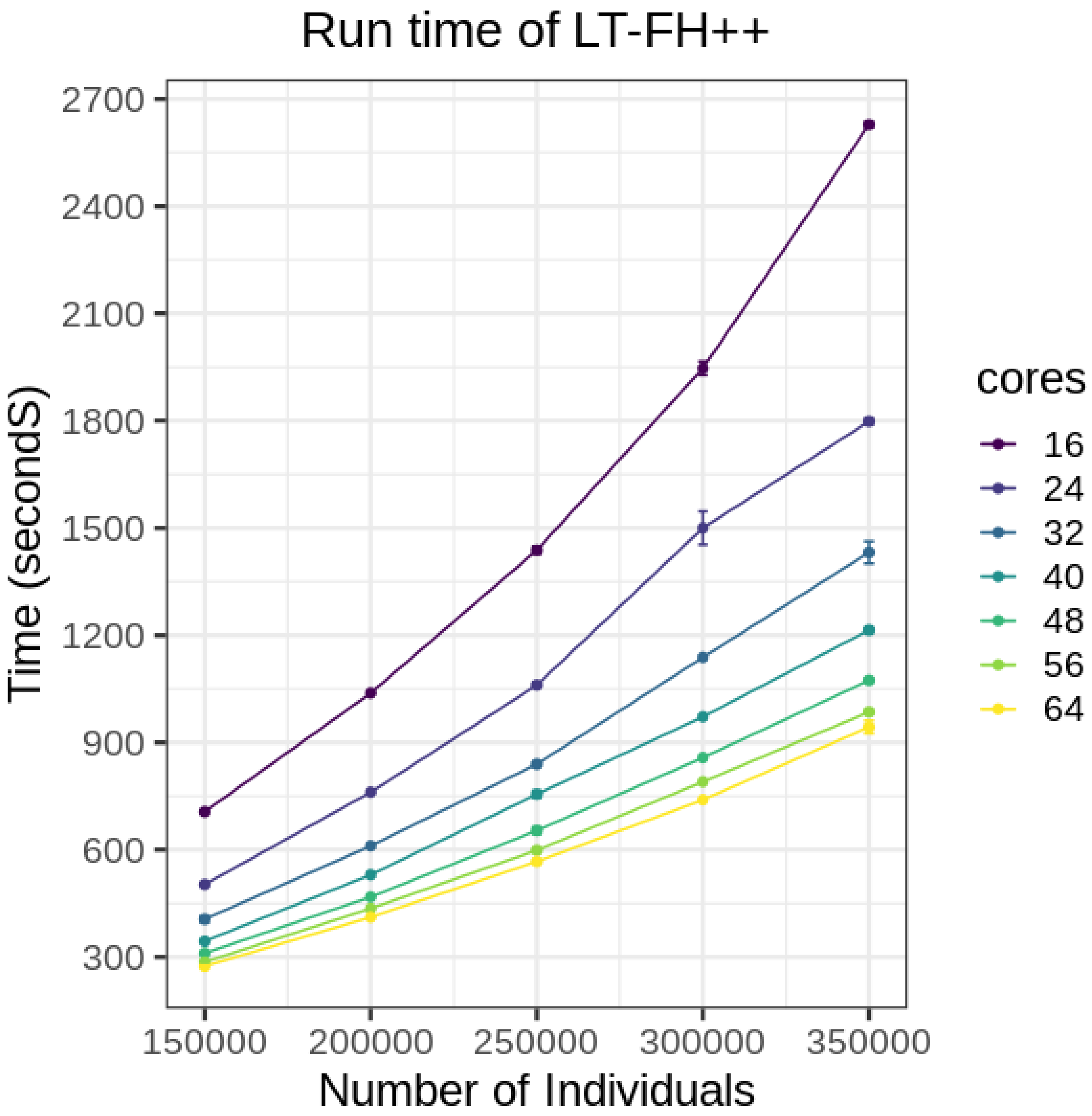

Figure S2: Plot of computation times of LT-FH++ with varying number of cores and individuals. This plot shows computation times for more than 100k individuals and 16 to 64 cores. Error bars correspond to the standard error of the times multiplied by 1.96.

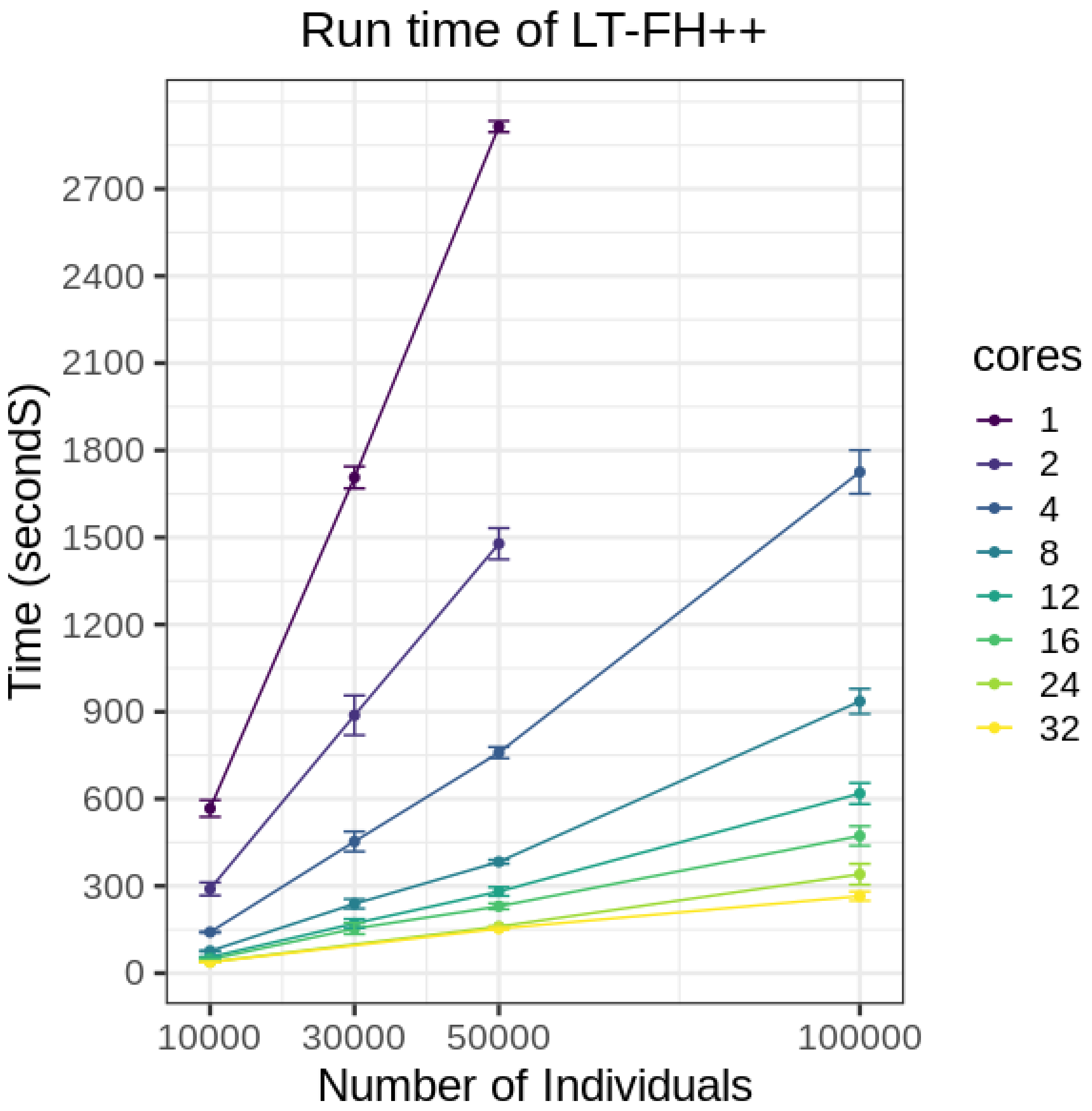

Figure S3: Plot of computation times of LT-FH++ with varying number of cores and individuals. This plot shows computation times for at most 100k individuals and 1 to 32 cores. Error bars correspond to the standard error of the times multiplied by 1.96.

Simulation Results

Simulation Results: 5% Prevalence

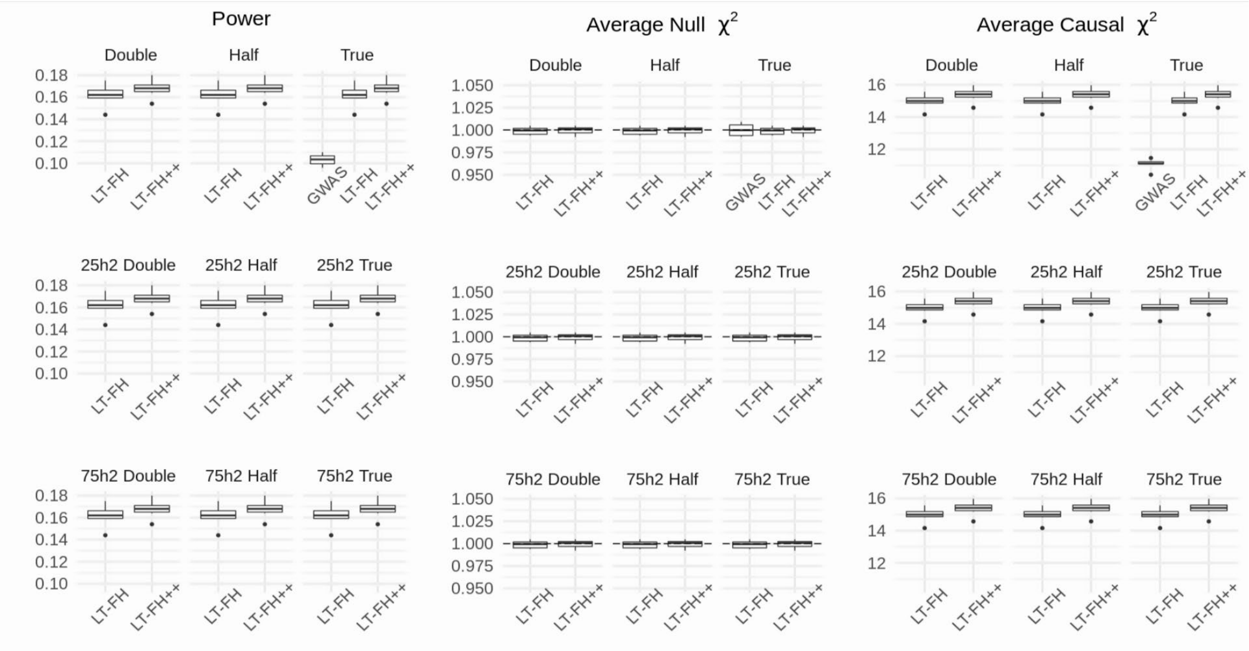

Figure S4: Simulation results with misspecified parameters and a prevalence of 5%. “Half” and “Double” refers to the misspecified prevalence, where “Half” means half of the true prevalence was used, and “Double” means double of the true prevalence was used. For reference, we added “True”, which is the true prevalence. If no heritability is specified in a subplot’s title, the default heritability of 50% was used. The true underlying heritability remains 50%.

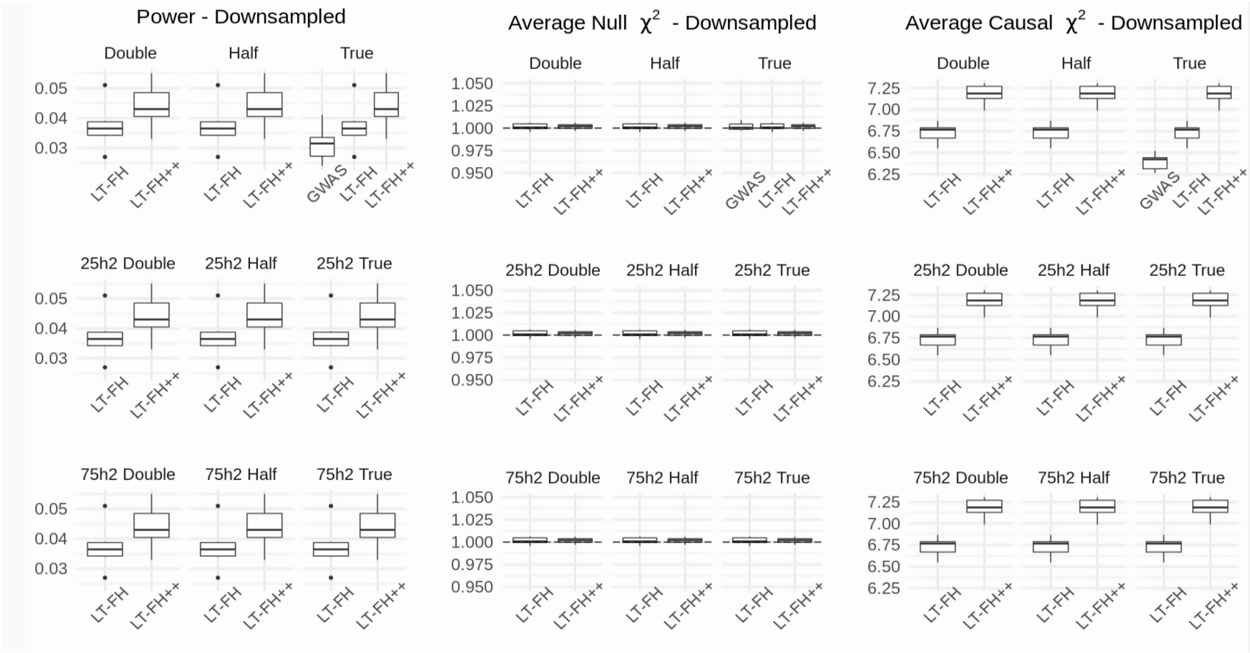

Figure S5: Simulation results with misspecified parameters, a prevalence of 5%, and downsampling of controls. “Half” and “Double” refers to the misspecified prevalence, and “Half” means half of the true prevalence was used, and “Double” means double of the true prevalence was used. For reference, we added “True”, which is the true prevalence. If no heritability is specified in a subplot’s title, the default heritability of 50% was used. The true underlying heritability remains 50%.

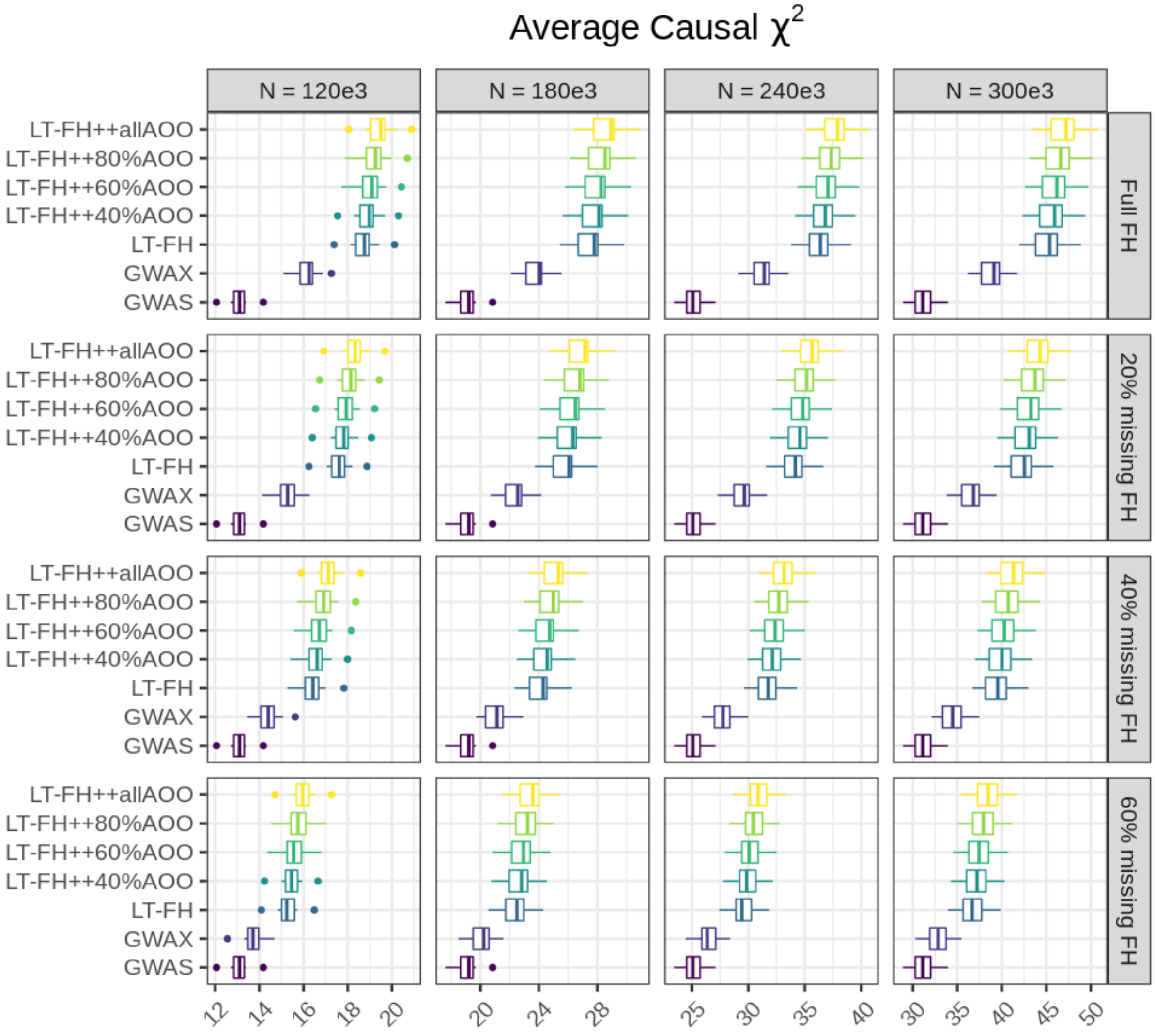

Figure S6: Simulation results of varying degrees of missingness in family history and age-of-onset. The simulation setup used is the default setting, with a prevalence of 5%, varying the number of individuals between 120k and 300k in steps of 60k, and family history and age-of-onset available for everyone to 40% in steps of 20% for each method (where applicable). Family history and age-of-onset information is removed at random, but for the same individuals across phenotypes.

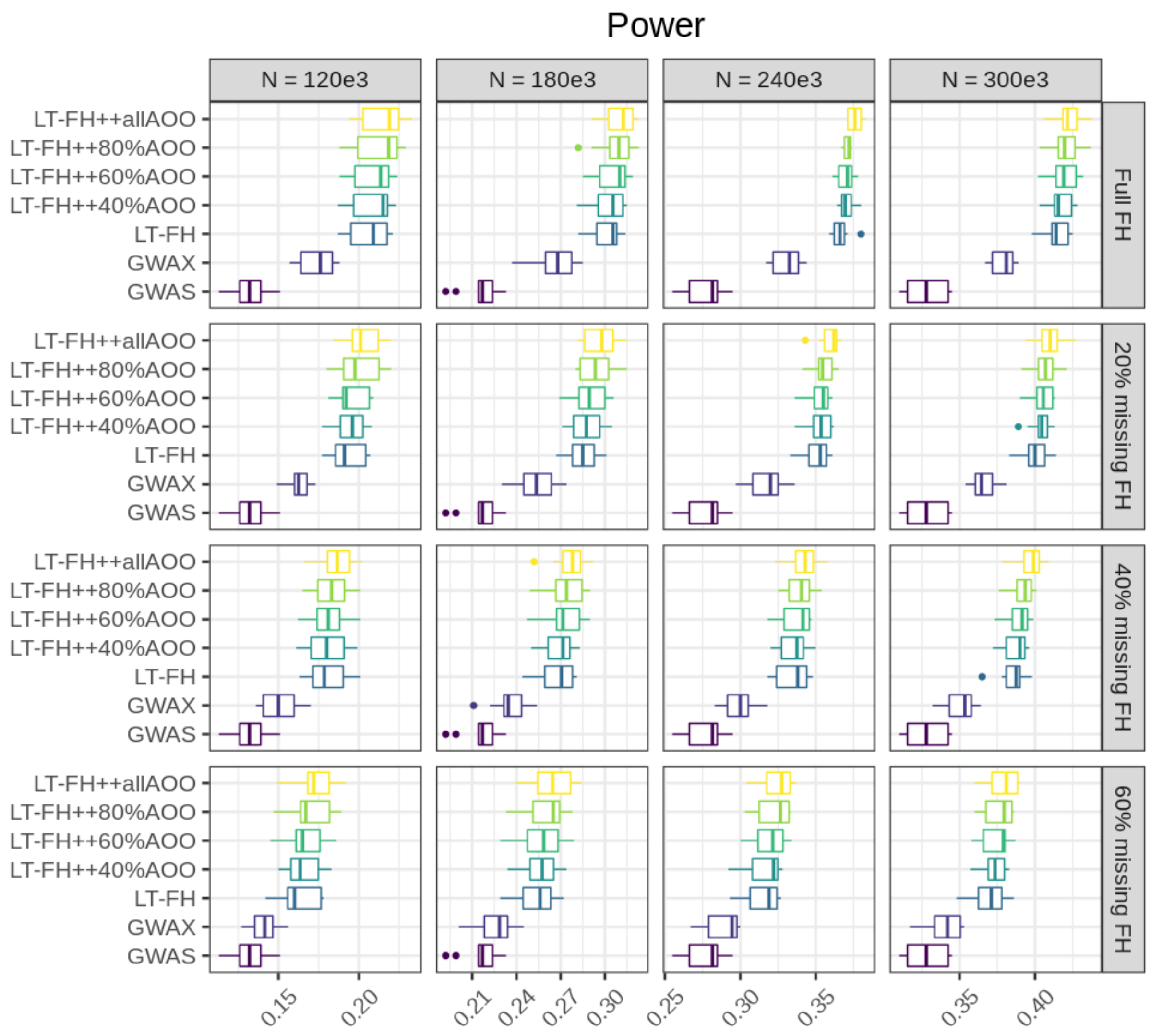

Figure S7: Simulation results of varying degrees of missingness in family history and age-of-onset. The simulation setup used is the default setting, with a prevalence of 5%, varying the number of individuals between 120k and 300k in steps of 60k, and family history and age-of-onset available for everyone to 40% in steps of 20% for each method (where applicable). Family history and age-of-onset information is removed at random, but for the same individuals across phenotypes.

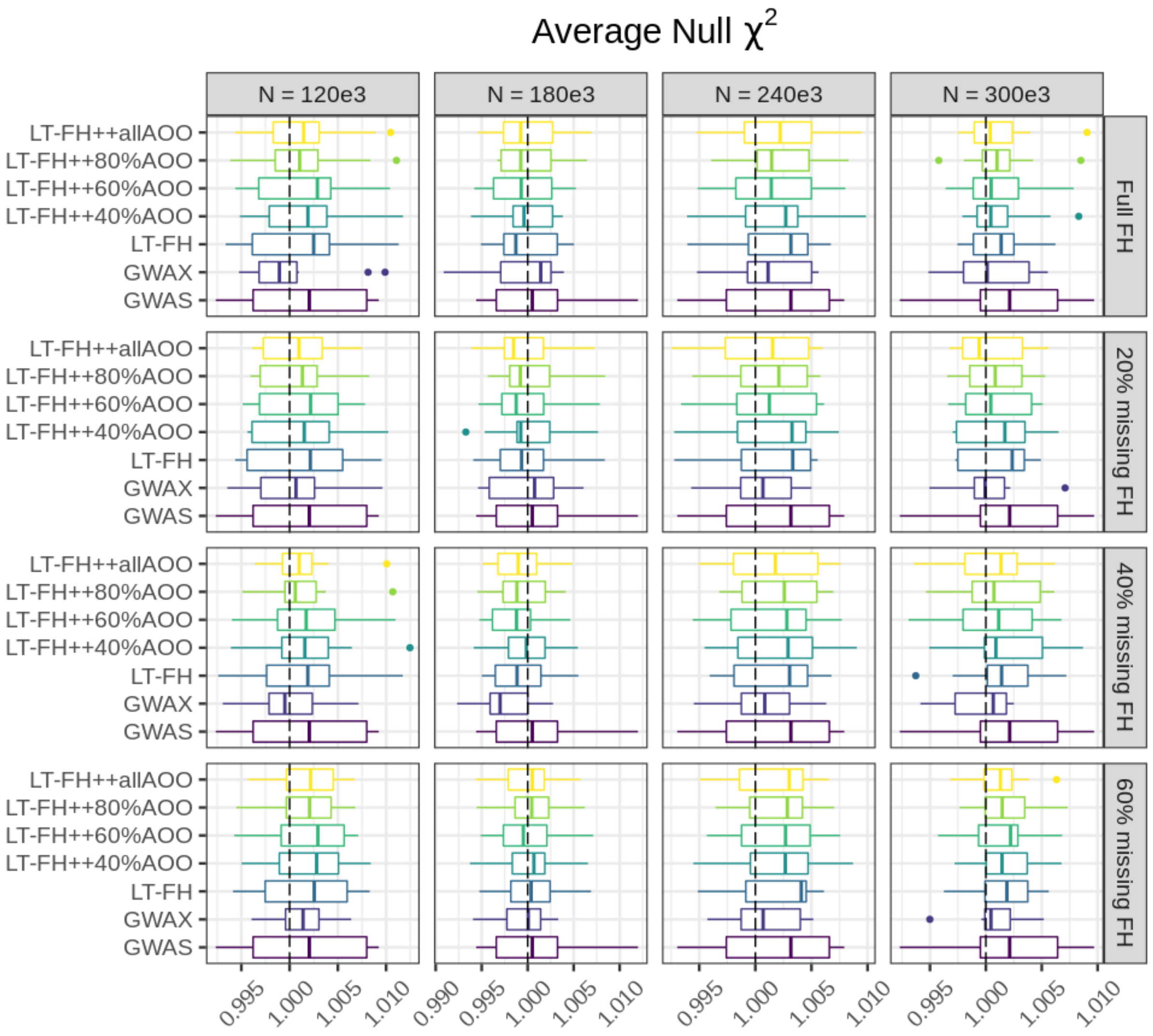

Figure S8: Simulation results of varying degrees of missingness in family history and age-of-onset. The simulation setup used is the default setting, with a prevalence of 5%, varying the number of individuals between 120k and 300k in steps of 60k, and family history and age-of-onset available for everyone to 40% in steps of 20% for each method (where applicable). Family history and age-of-onset information is removed at random, but for the same individuals across phenotypes.

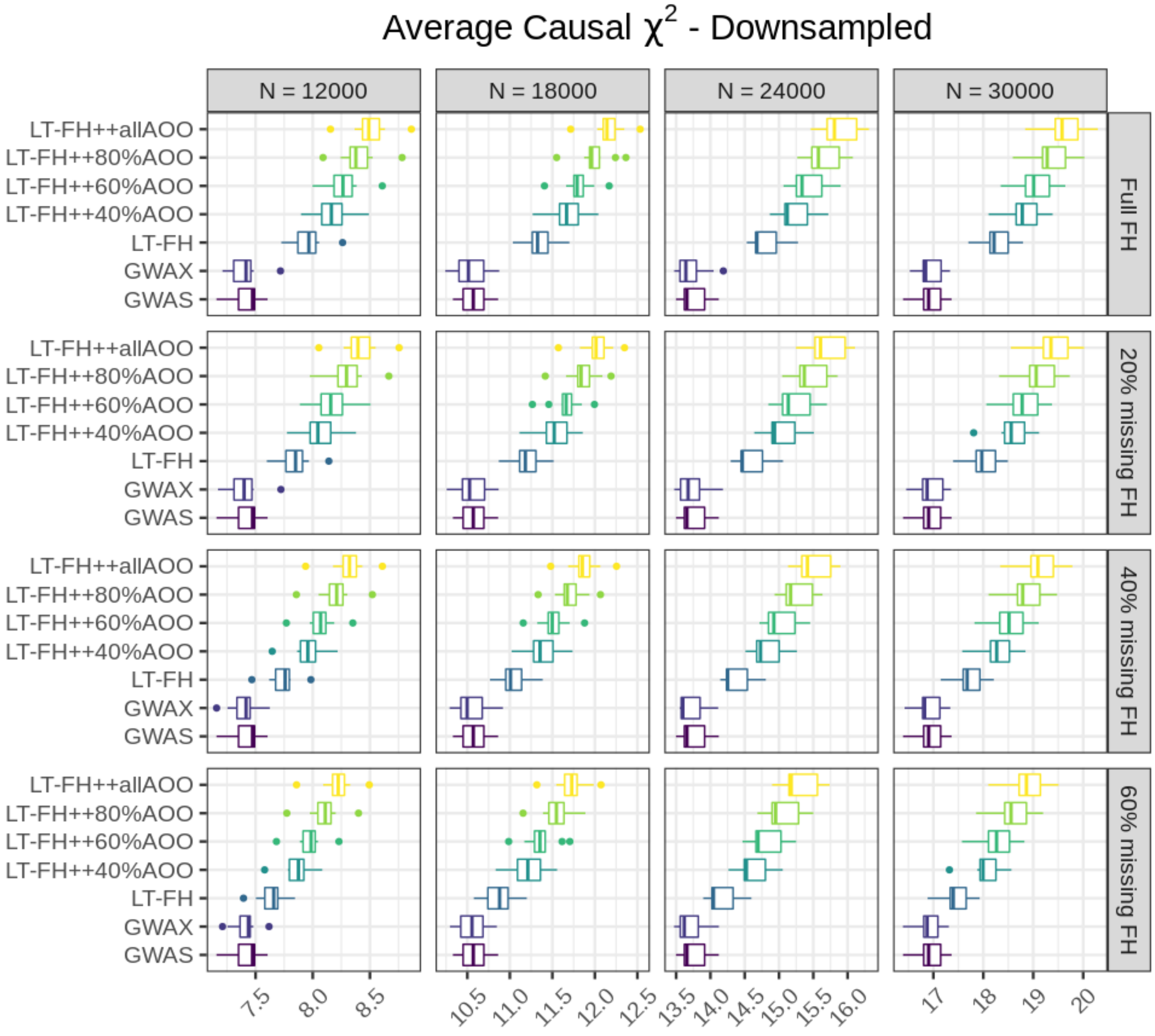

Figure S9: Simulation results of varying degrees of missingness in family history and age-of-onset when downsampling controls. The simulation setup used is the default setting, with a prevalence of 5%, varying the number of individuals between 12k and 30k in steps of 6k, and family history and age-of-onset available for everyone to 40% in steps of 20% for each method (where applicable). Family history and age-of-onset information is removed at random, but for the same individuals across phenotypes.

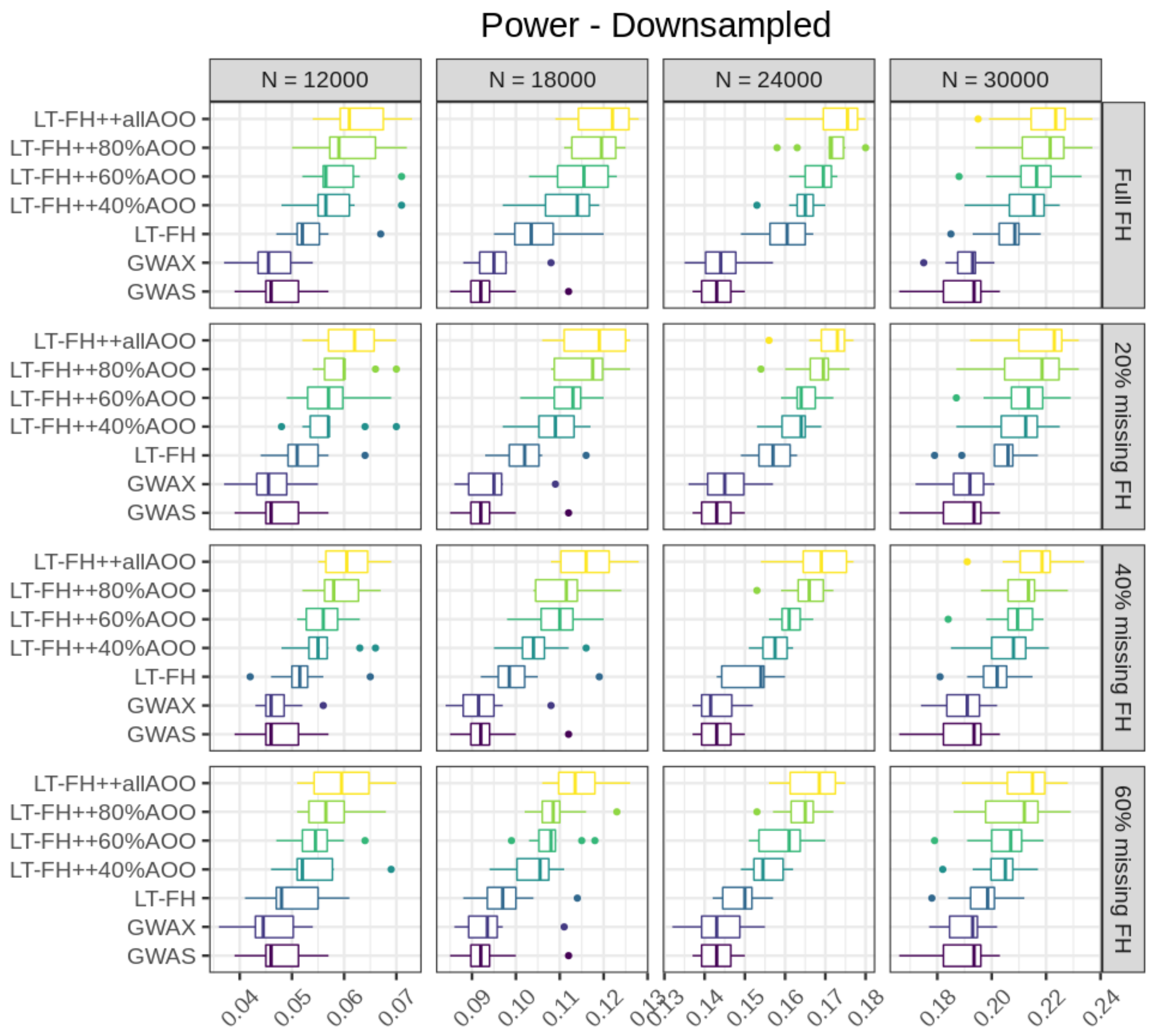

Figure S10: Simulation results of varying degrees of missingness in family history and age-of-onset when downsampling controls. The simulation setup used is the default setting, with a prevalence of 5%, varying the number of individuals between 12k and 30k in steps of 6k, and family history and age-of-onset available for everyone to 40% in steps of 20% for each method (where applicable). Family history and age-of-onset information is removed at random, but for the same individuals across phenotypes.

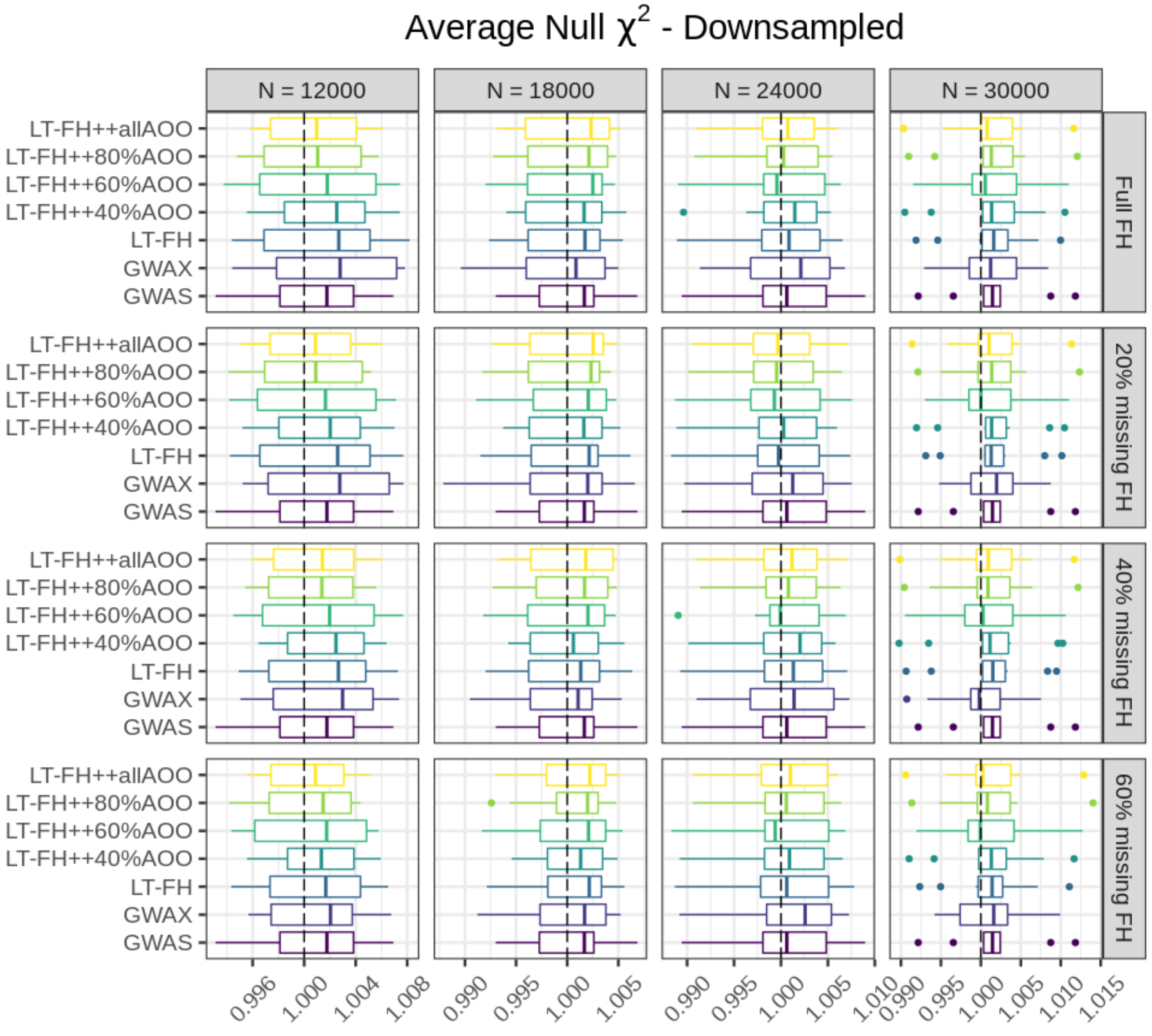

Figure S11: Simulation results of varying degrees of missingness in family history and age-of-onset when downsampling controls. The simulation setup used is the default setting, with a prevalence of 5%, varying the number of individuals between 12k and 30k in steps of 6k, and family history and age-of-onset available for everyone to 40% in steps of 20% for each method (where applicable). Family history and age-of-onset information is removed at random, but for the same individuals across phenotypes.

Simulation Results: 10% Prevalence

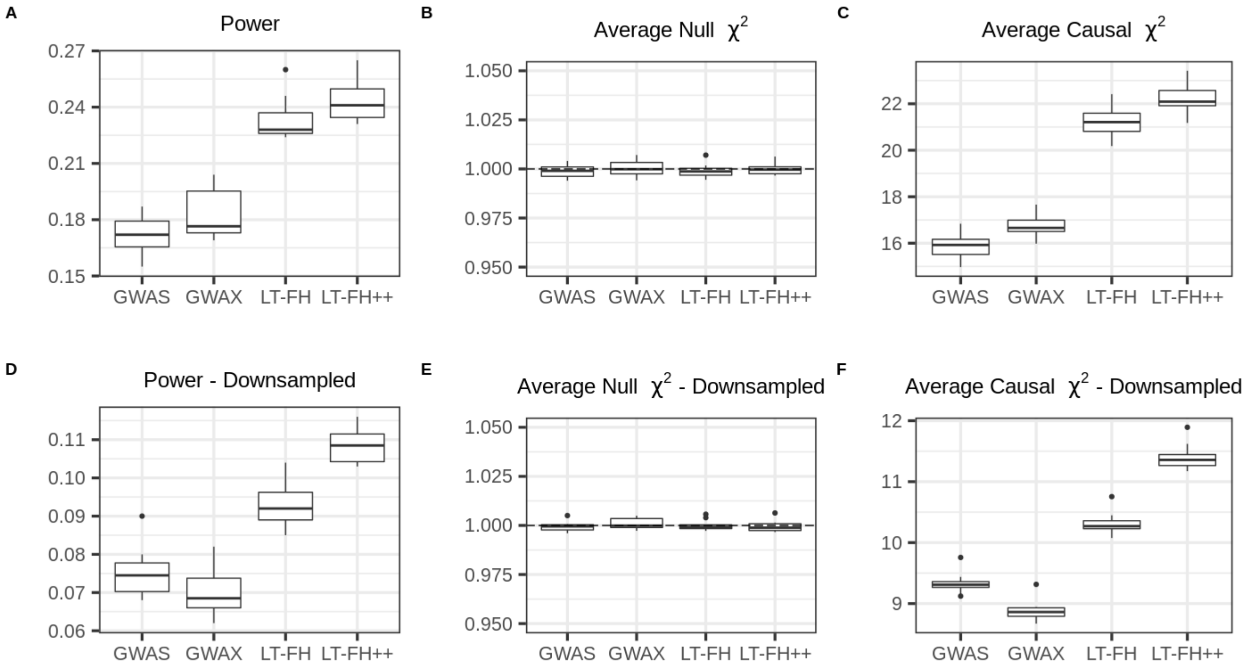

Figure S12: Simulation results under the default simulation parameters and a prevalence of 10%.

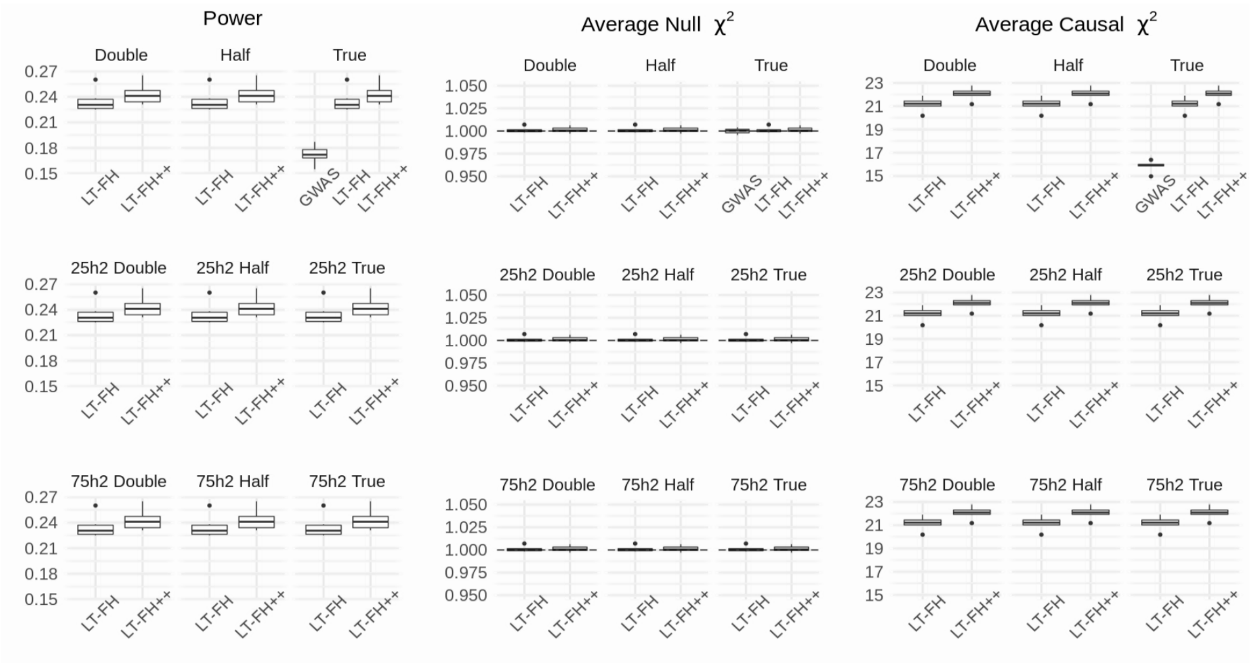

Figure S13: Simulation results with misspecified parameters and a prevalence of 10%. “Half” and “Double” refers to the misspecified prevalence, and “Half” means half of the true prevalence was used, and “Double” means double of the true prevalence was used. For reference, we added “True”, which is the true prevalence. If no heritability is specified in a subplot’s title, the default heritability of 50% was used. The true underlying heritability remains 50%.

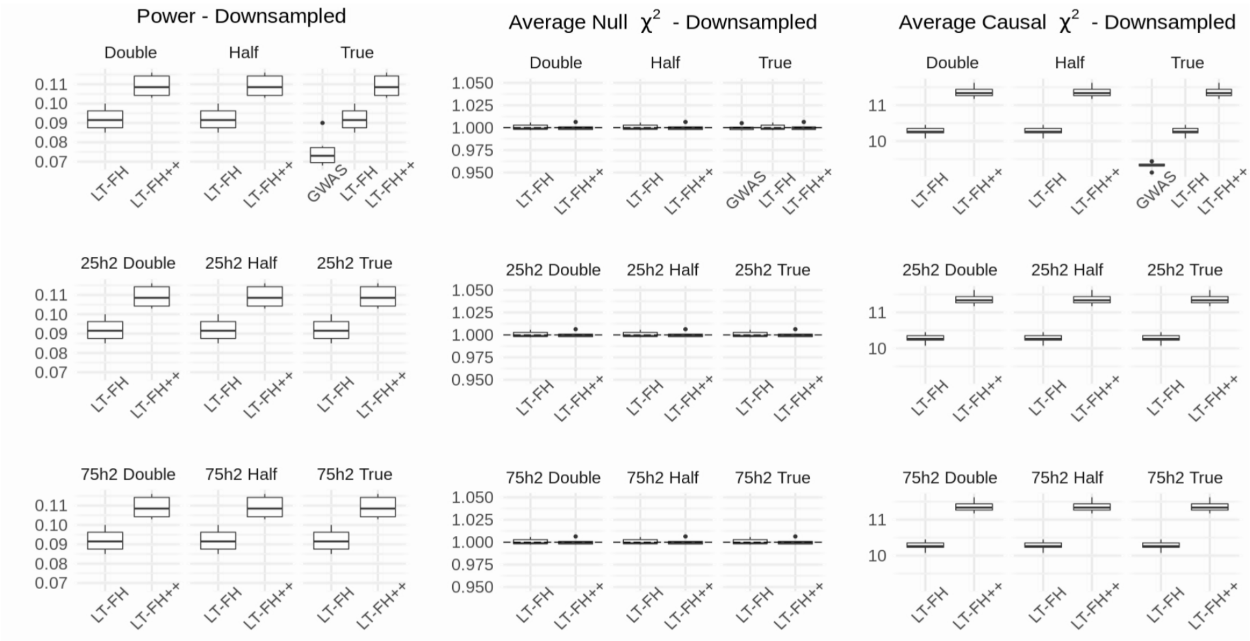

Figure S14: Simulation results with misspecified parameters, a prevalence of 10%, and downsampling of controls. “Half” and “Double” refers to the misspecified prevalence, and “Half” means half of the true prevalence was used, and “Double” means double of the true prevalence was used. For reference, we added “True”, which is the true prevalence. If no heritability is specified in a subplot’s title, the default heritability of 50% was used. The true underlying heritability remains 50%.

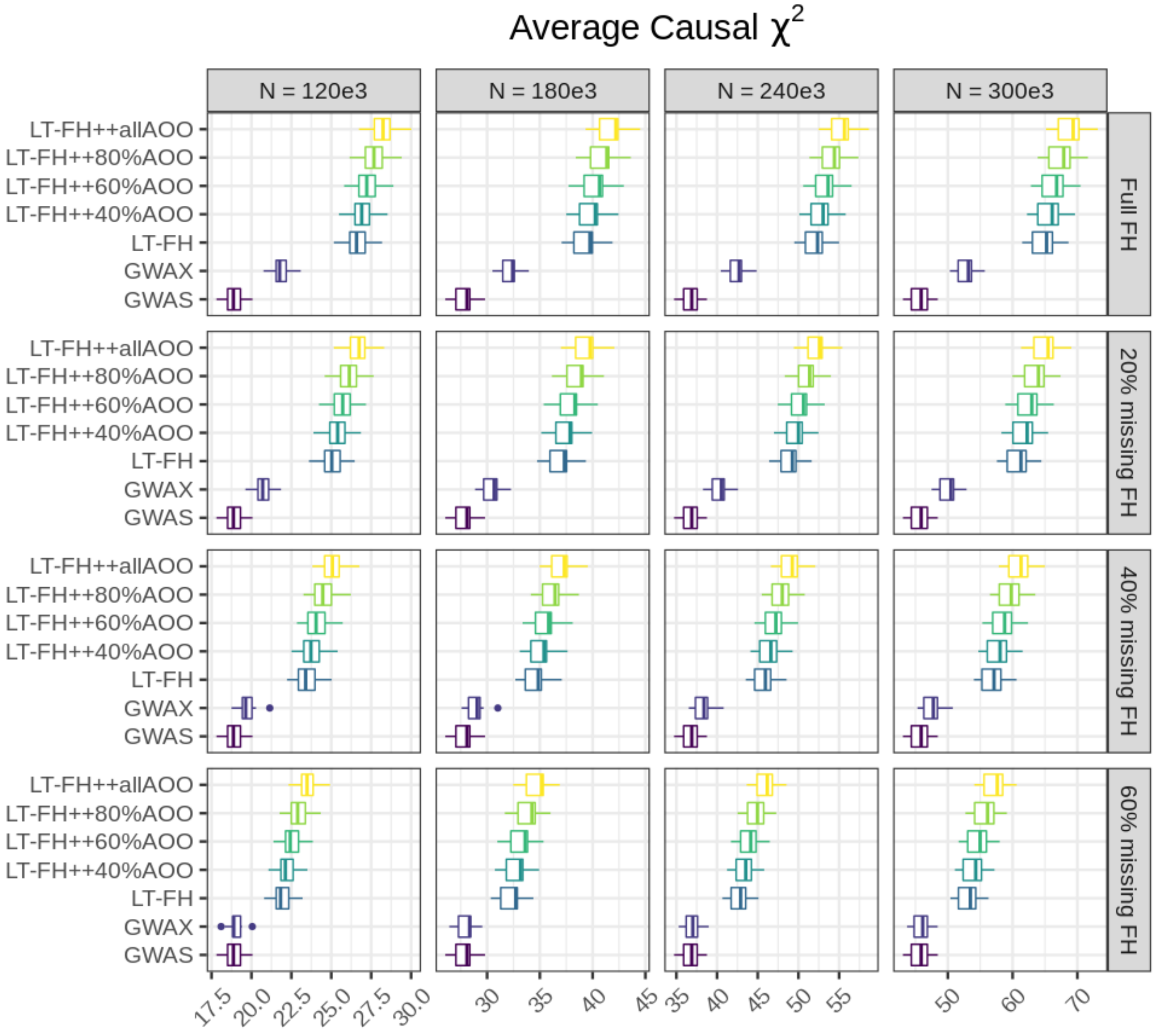

Figure S15: Simulation results of varying degrees of missingness in family history and age-of-onset. The simulation setup used is the default setting, with a prevalence of 10%, varying the number of individuals between 120k and 300k in steps of 60k, and family history and age-of-onset available for everyone to 40% in steps of 20% for each method (where applicable). Family history and age-of-onset information is removed at random, but for the same individuals across phenotypes.

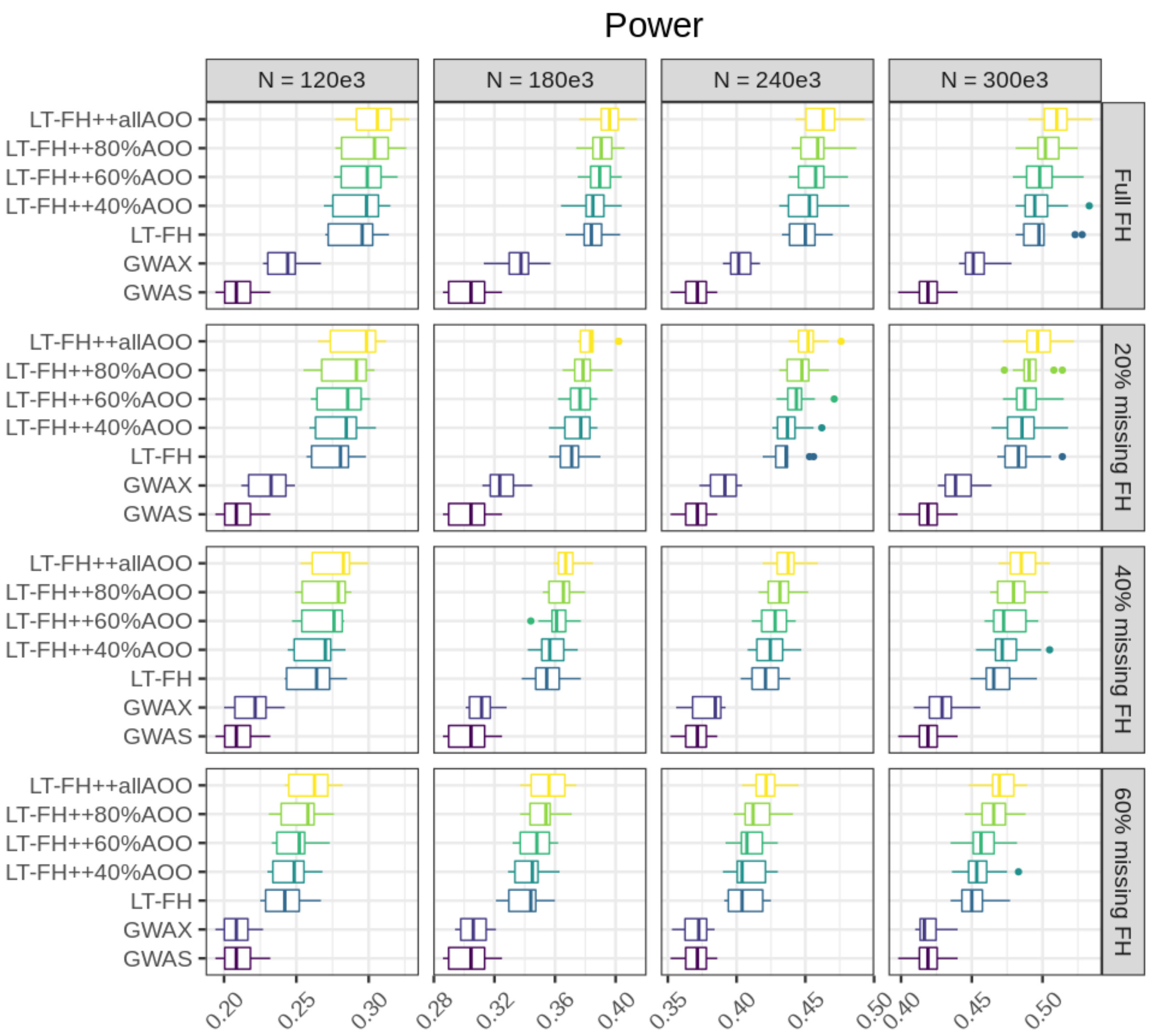

Figure S16: Simulation results of varying degrees of missingness in family history and age-of-onset. The simulation setup used is the default setting, with a prevalence of 10%, varying the number of individuals between 120k and 300k in steps of 60k, and family history and age-of-onset available for everyone to 40% in steps of 20% for each method (where applicable). Family history and age-of-onset information is removed at random, but for the same individuals across phenotypes.

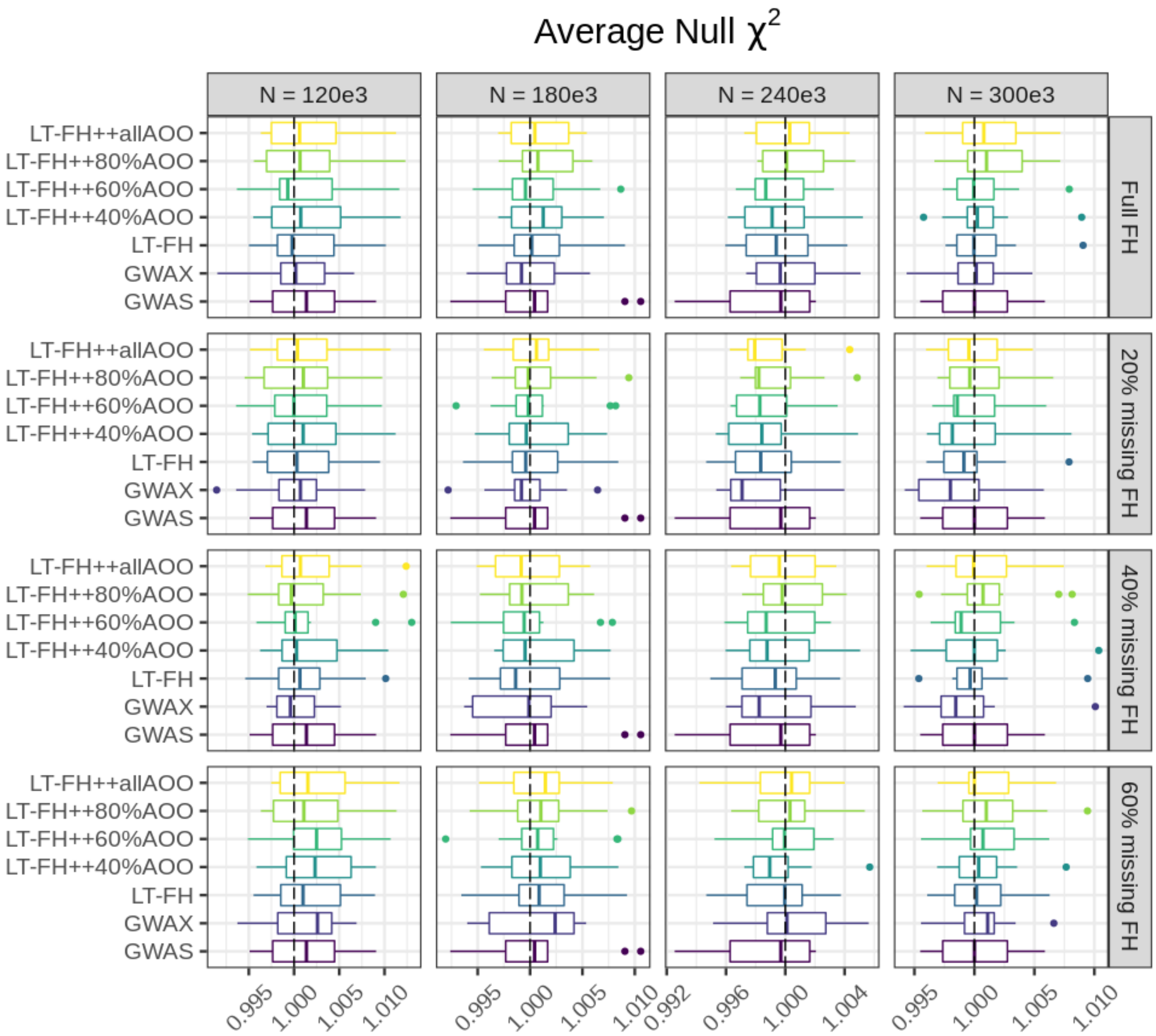

Figure S17: Simulation results of varying degrees of missingness in family history and age-of-onset. The simulation setup used is the default setting, with a prevalence of 10%, varying the number of individuals between 120k and 300k in steps of 60k, and family history and age-of-onset available for everyone to 40% in steps of 20% for each method (where applicable). Family history and age-of-onset information is removed at random, but for the same individuals across phenotypes.

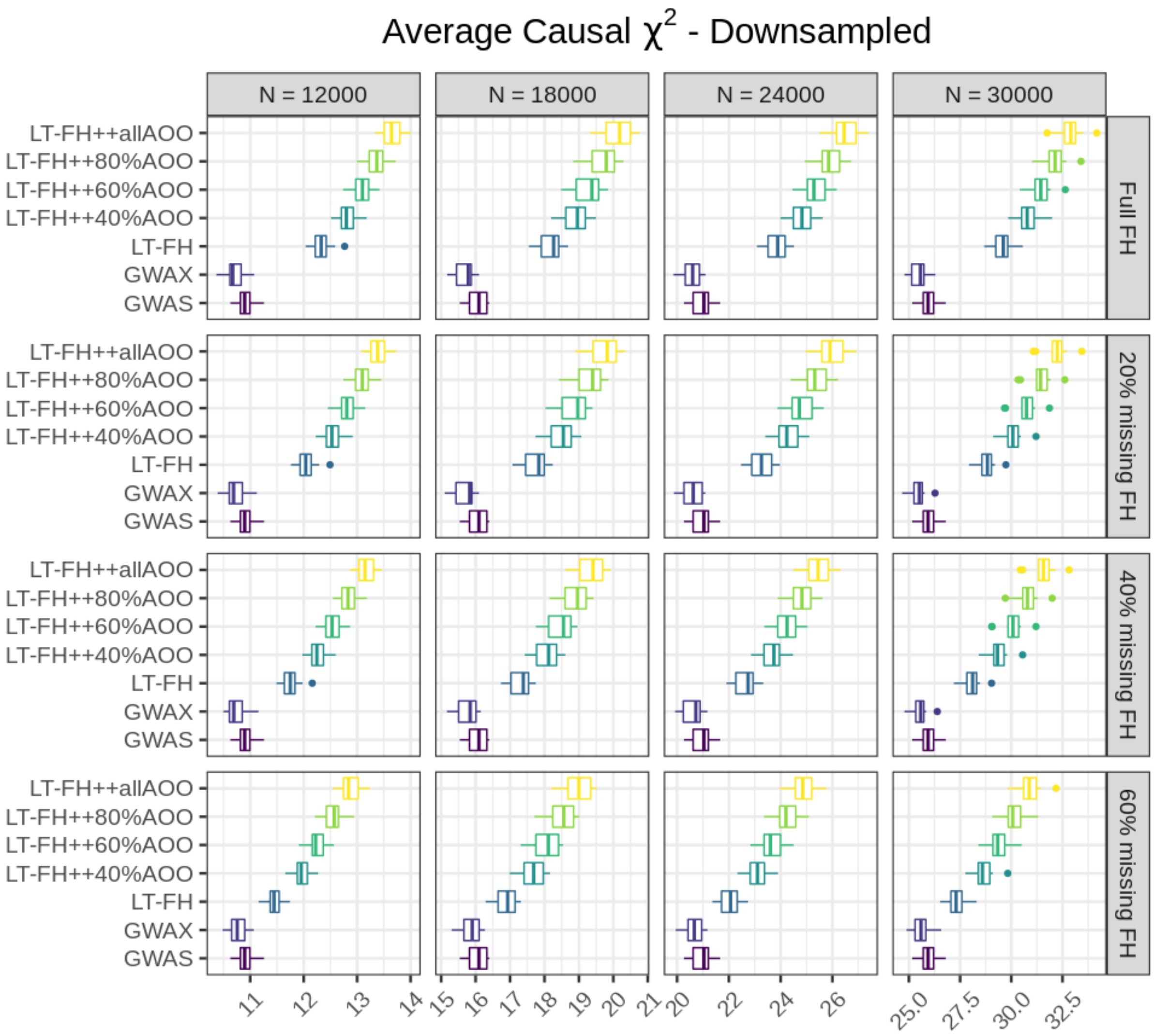

Figure S18: Simulation results of varying degrees of missingness in family history and age-of-onset when downsampling controls. The simulation setup used is the default setting, with a prevalence of 10%, varying the number of individuals between 12k and 30k in steps of 6k, and family history and age-of-onset available for everyone to 40% in steps of 20% for each method (where applicable). Family history and age-of-onset information is removed at random, but for the same individuals across phenotypes.

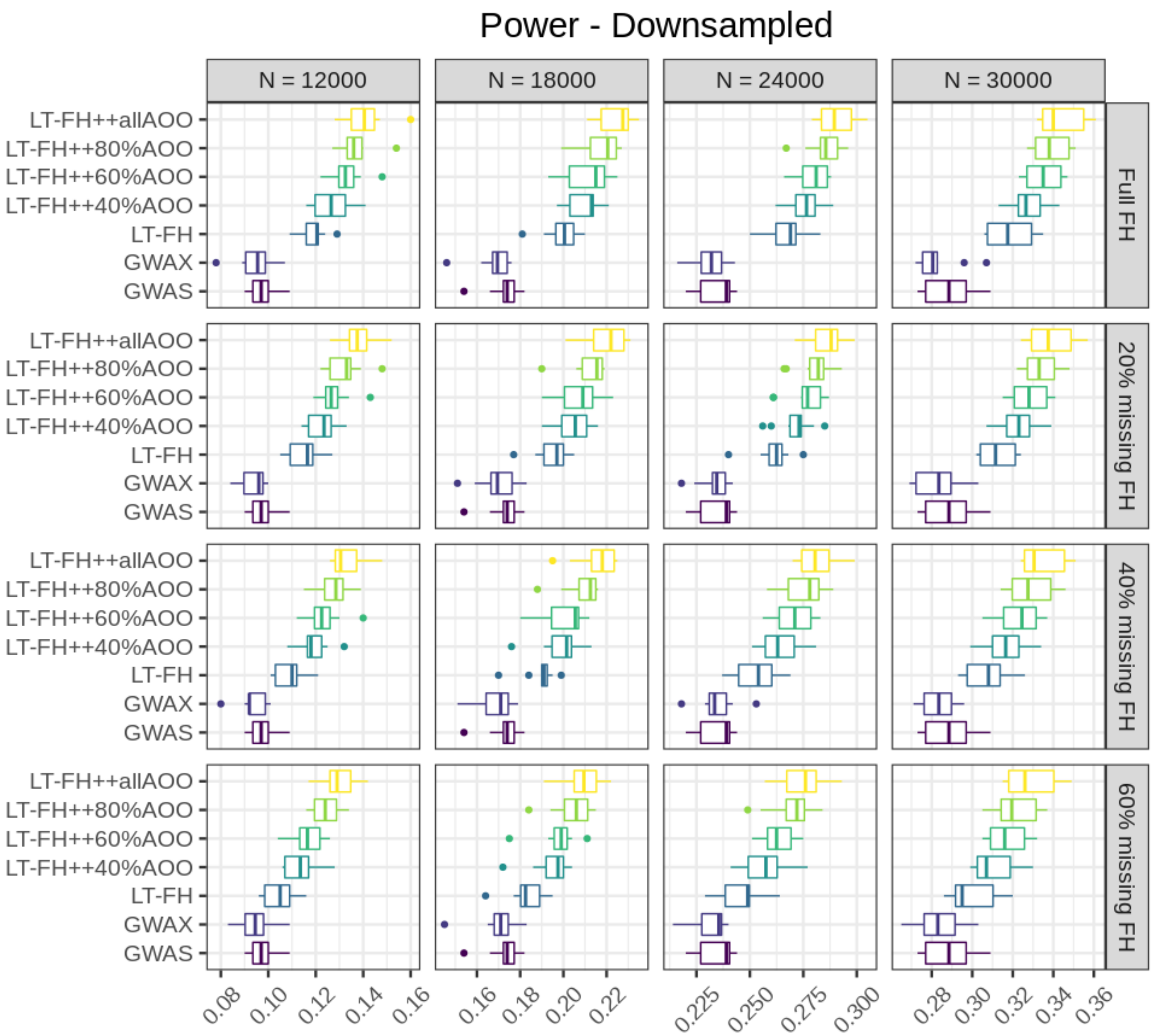

Figure S19: Simulation results of varying degrees of missingness in family history and age-of-onset when downsampling controls. The simulation setup used is the default setting, with a prevalence of 5%, varying the number of individuals between 12k and 30k in steps of 6k, and family history and age-of-onset available for everyone to 40% in steps of 20% for each method (where applicable). Family history and age-of-onset information is removed at random, but for the same individuals across phenotypes.

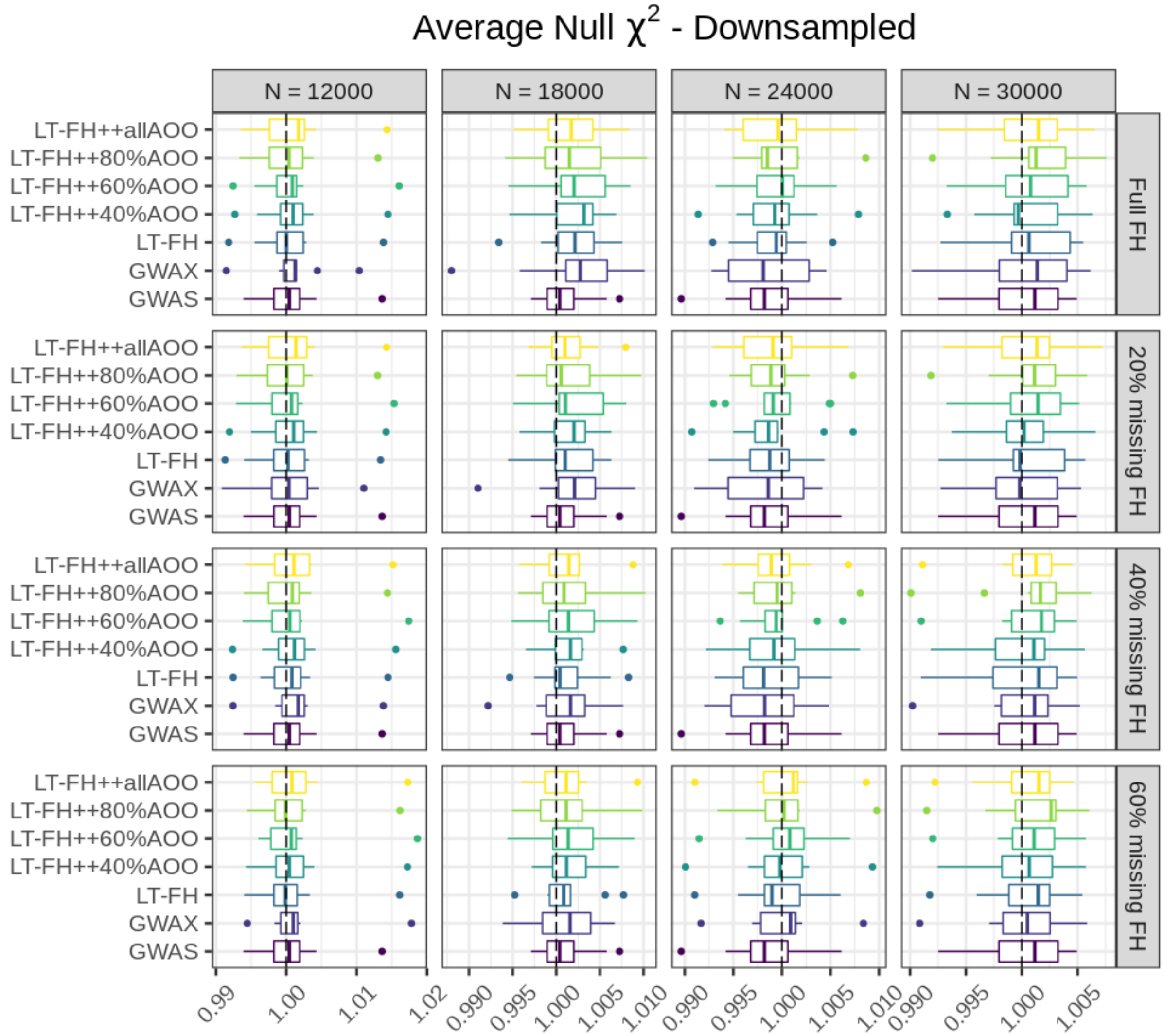

Figure S20: Simulation results of varying degrees of missingness in family history and age-of-onset when downsampling controls. The simulation setup used is the default setting, with a prevalence of 5%, varying the number of individuals between 12k and 30k in steps of 6k, and family history and age-of-onset available for everyone to 40% in steps of 20% for each method (where applicable). Family history and age-of-onset information is removed at random, but for the same individuals across phenotypes.

Mortality Results

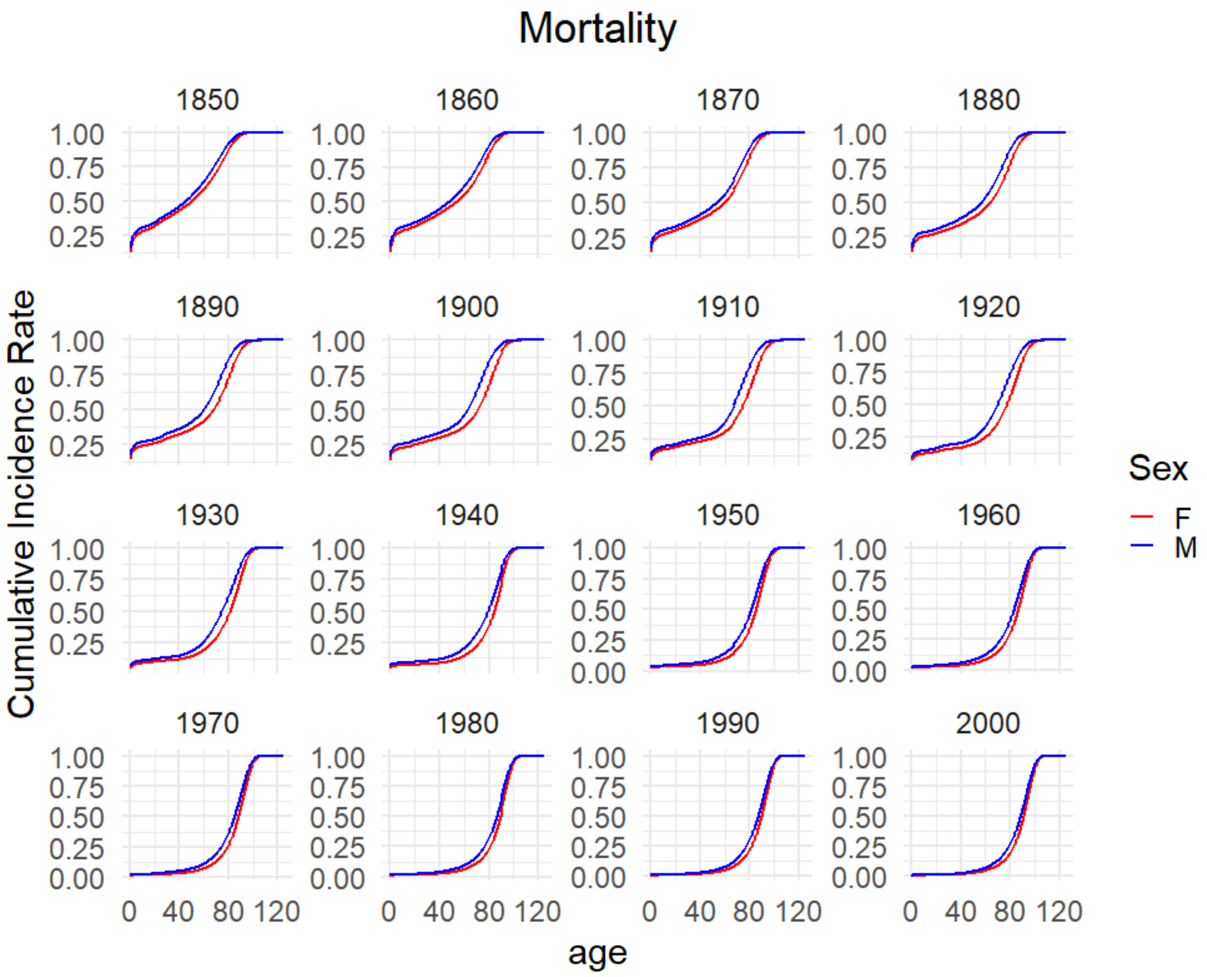

Figure S21: Plot of mortality from England and Wales, obtained from the Office for National Statistics (ONS). We plotted the cumulative mortality for each sex and from the begining of each decade from 2000 to the begining of the data. Historic mortality rates have been used upto the present, and projections for future predictions.

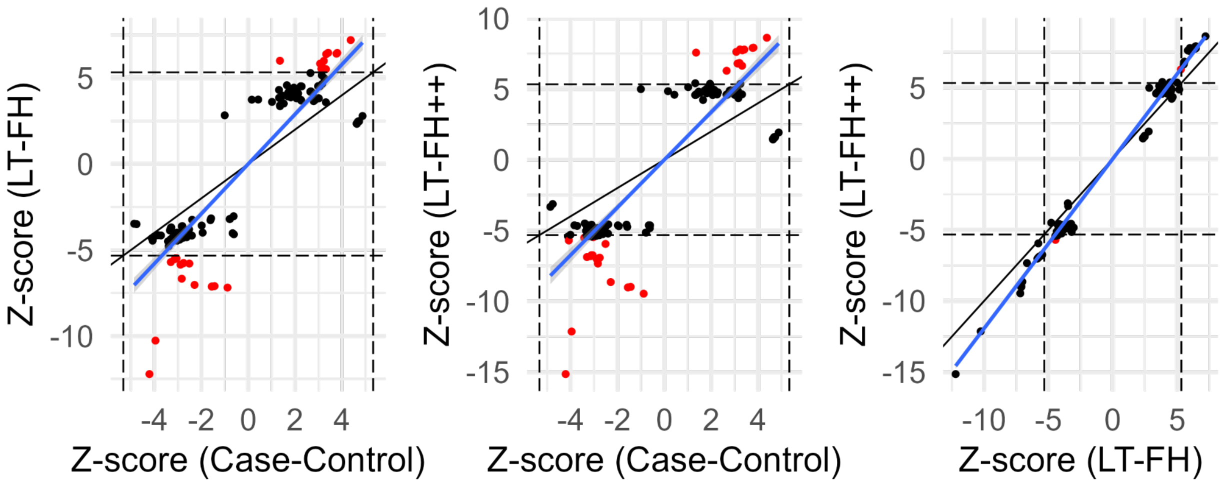

Figure S22: Z-scores for mortality in the UK biobank. We filtered on variants that had a p-value < 5×10^{-6} for at least one of the three compared outcomes. The common set of variants were LD clumped (prioritizing on minor allele frequencies) in an attempt to not bias one outcome over another. The dashed line correspond to a p-value of 5×10^{-8}, and the red dots are SNPs that are genome-wide significant for only one method. The black line is the identity line and the blue line is the best fitted line. We filtered on the p-values, keeping SNPs that are below 5×10^{-6} for at least one of the compared methods and performed . The squared slope of the fitted line indicates the power improvement of one method over another

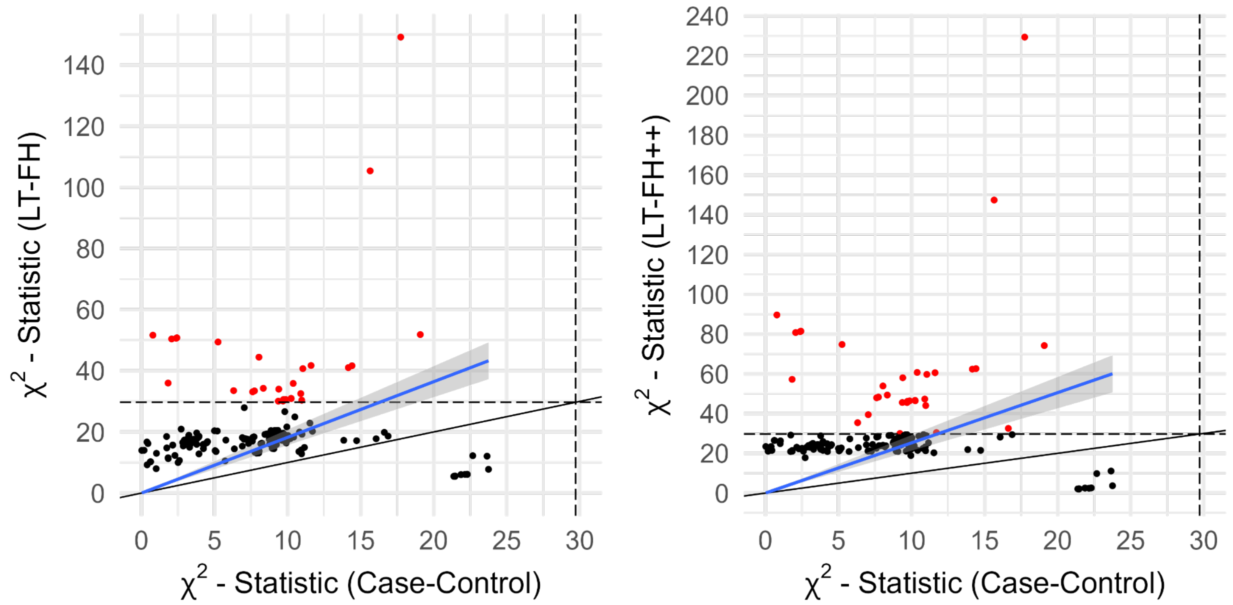

Figure S23: The χ^2 statistics for mortality between case-control status and LT-FH and LT-FH++ can be seen above. We filtered on variants that had a p-value $< 5 \times 10^{-6}$ for at least one of the three compared outcomes. The common set of variants were LD clumped (prioritizing on minor allele frequencies) in an attempt to not bias one outcome over another. The red dots are variants identified as genome-wide significant for only one of the outcomes. The black dots are suggestive associations identified by either method. The black line indicates the identity line and the blue line is the best fitted line using linear regression. The black dashed lines correspond to the threshold for genome-wide significance.

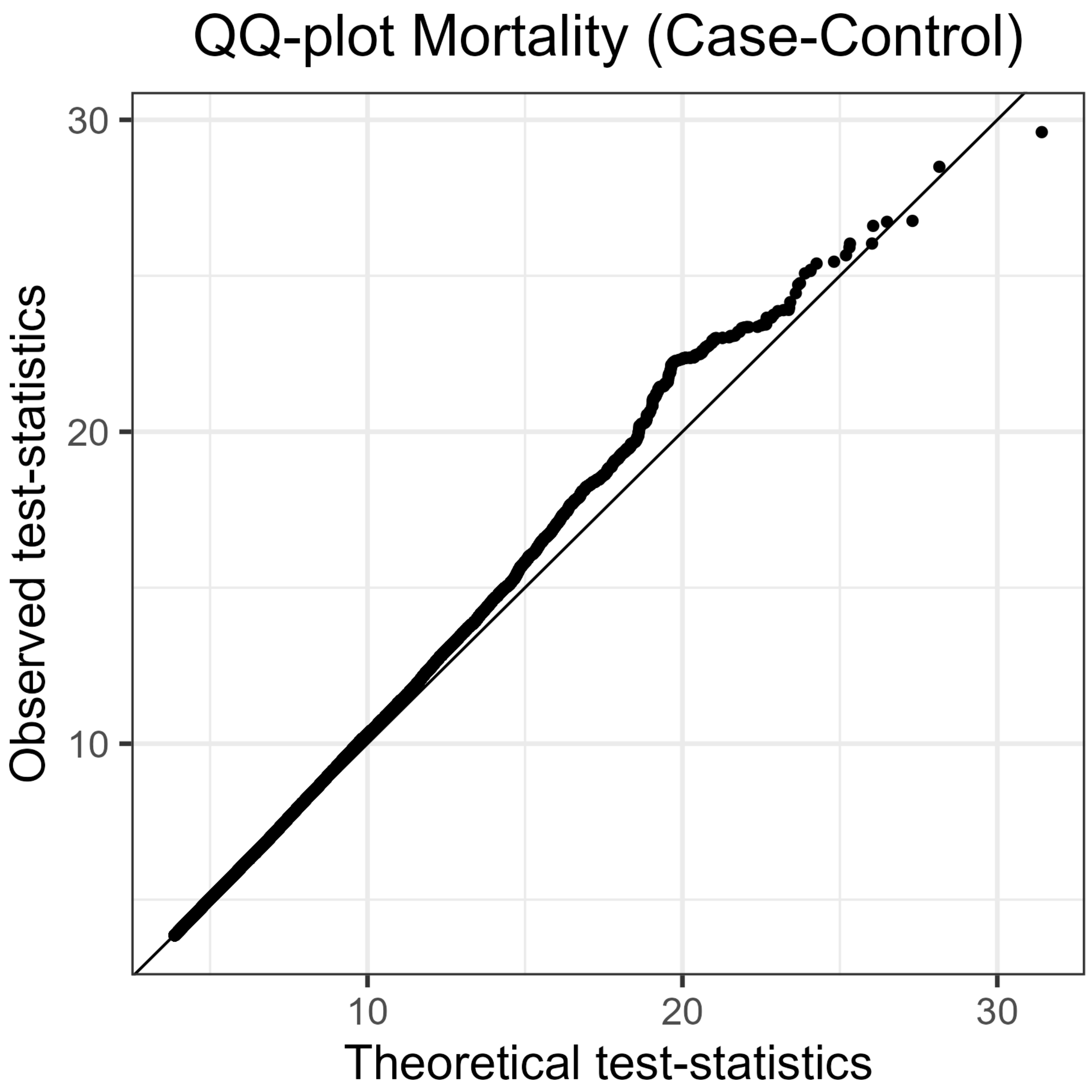

Figure S24: QQ plot of Mortality for Case-Control status. We excluded SNPs with p-values greater than 0.05.

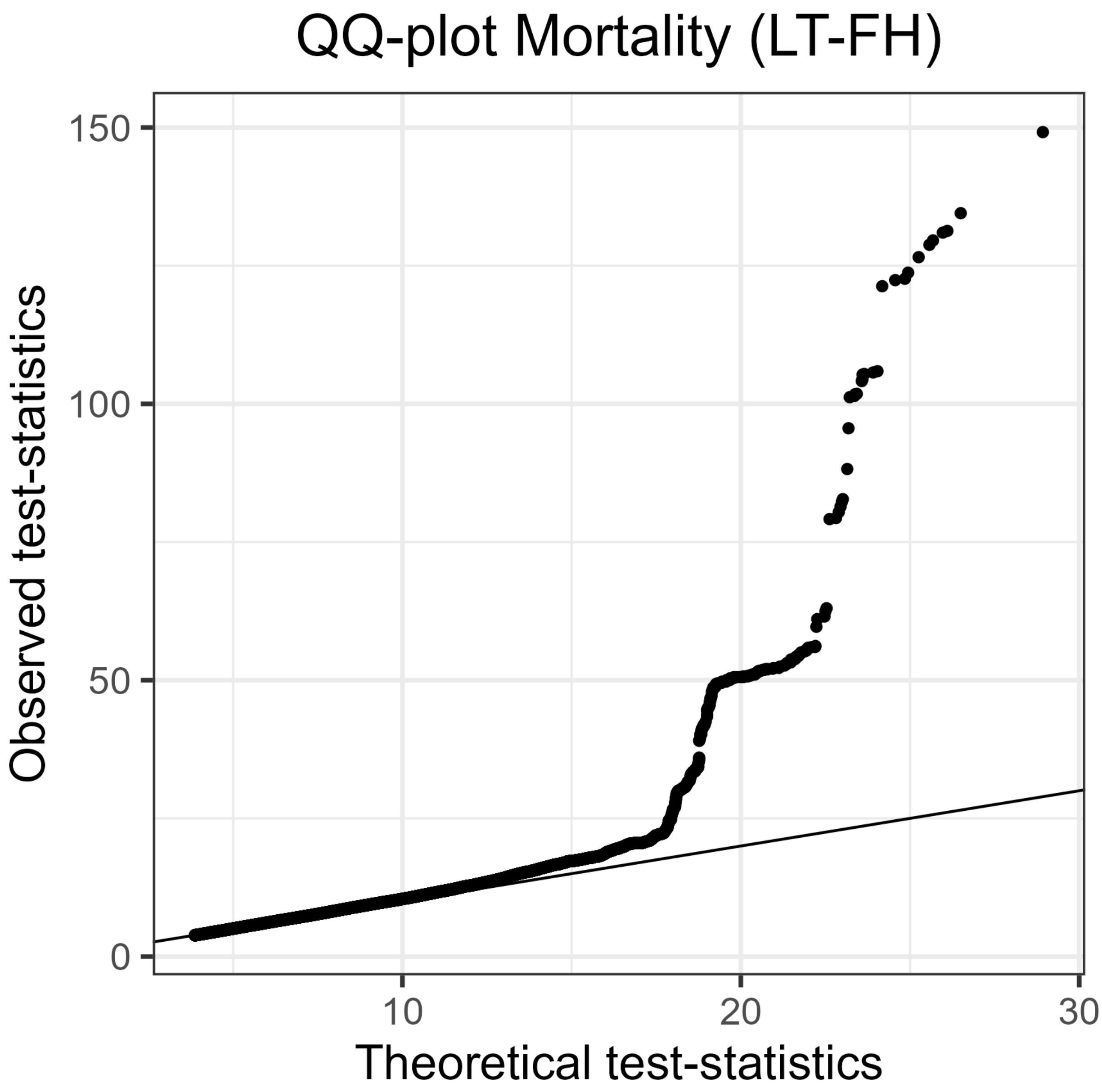

Figure S25: QQ plot of Mortality for LT-FH. We excluded SNPs with p-values greater than 0.05.

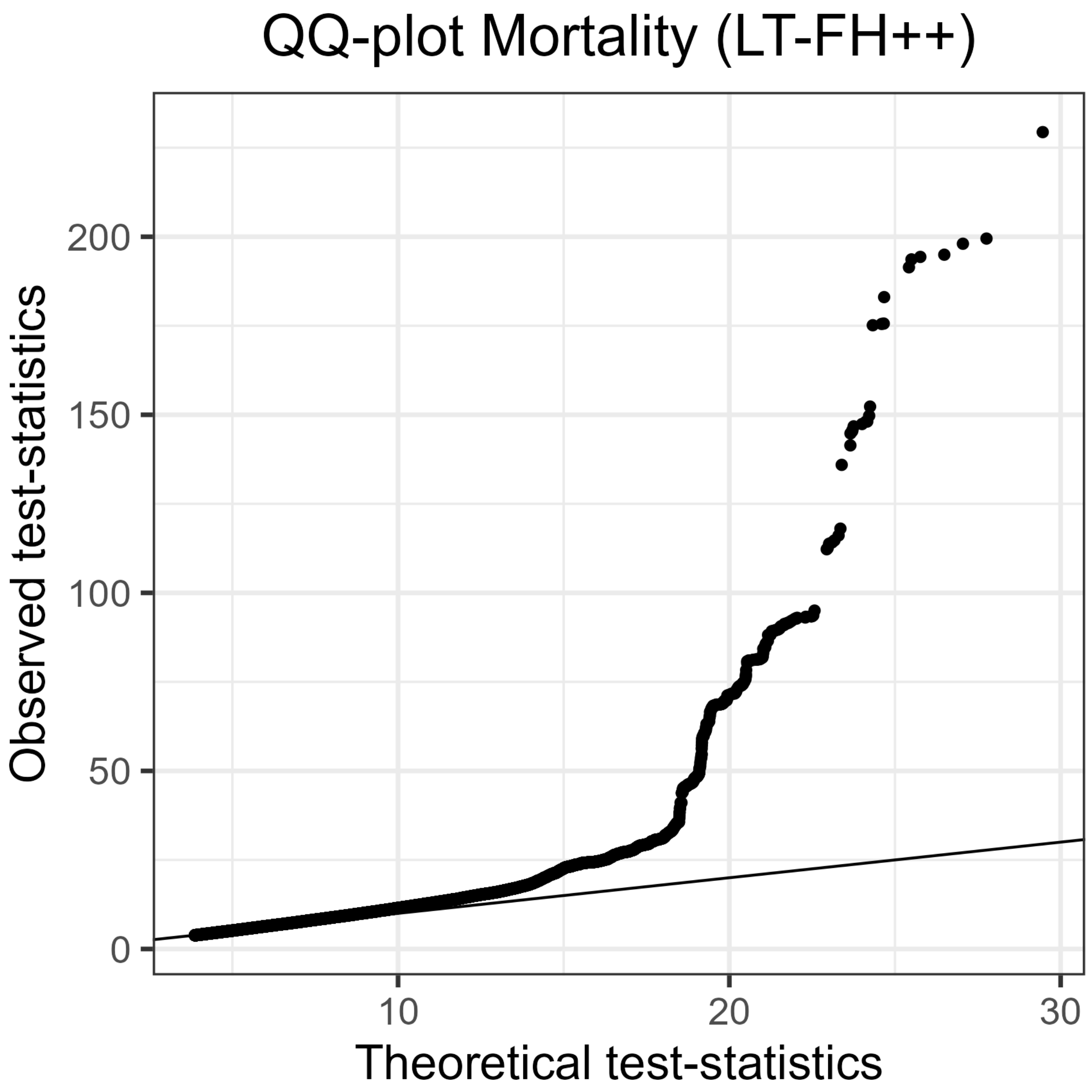

Figure S26: QQ plot of Mortality for LT-FH++. We excluded SNPs with p-values greater than 0.05.

iPSYCH Results

This part of the supplementary notes contains plots associated with the analysis of the iPSYCH, in particular about ADHD, ASD, DEP, and SCZ. The results appear in this order.

Attention Deficit Hyperactivity Disorder

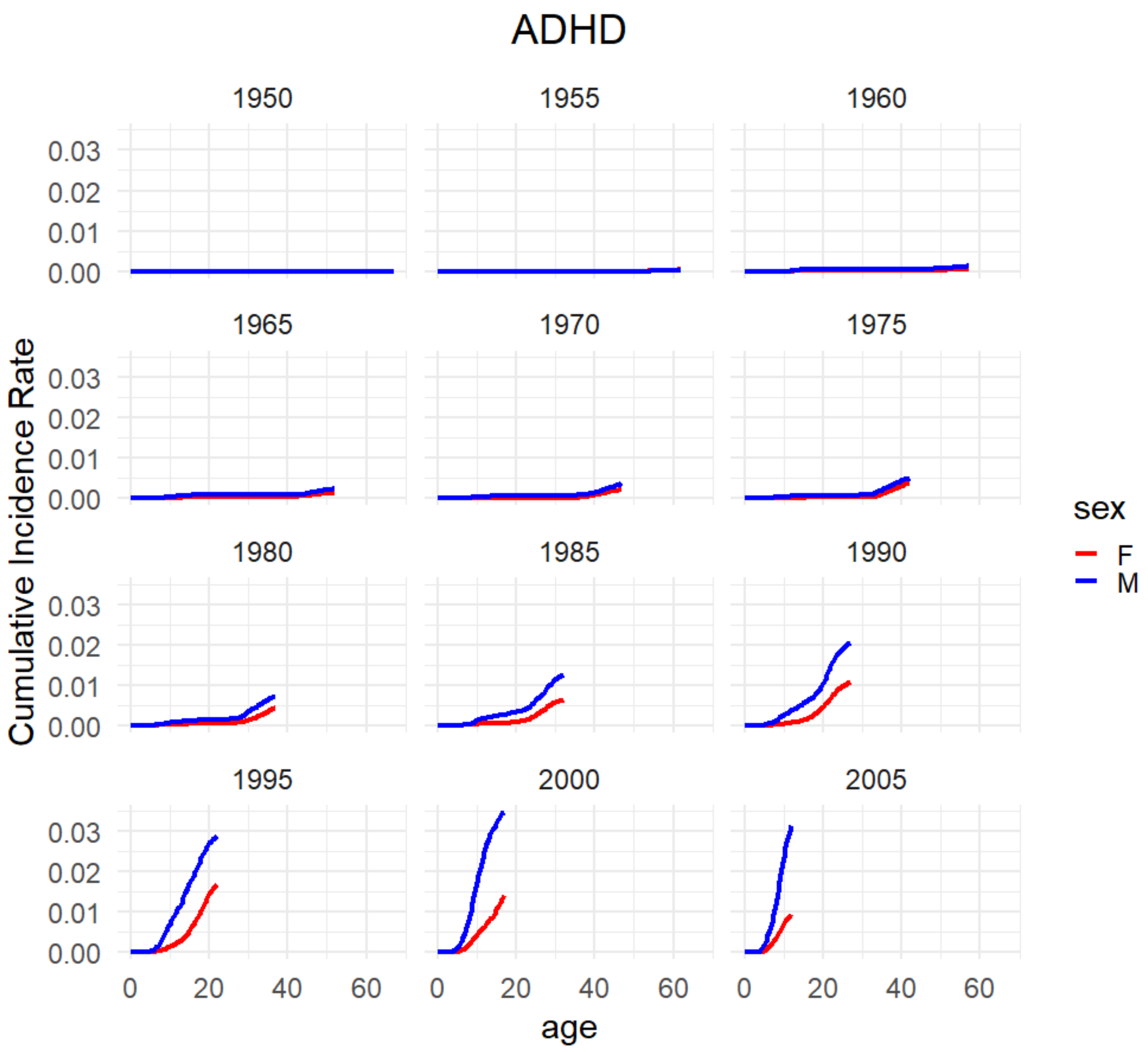

Figure S27: Plot of the cumulative incidence rate for Attention Deficit Hyperactivity Disorder grouped by birth year in the Danish registers. The red line corresponds to females and the blue corresponds to males.

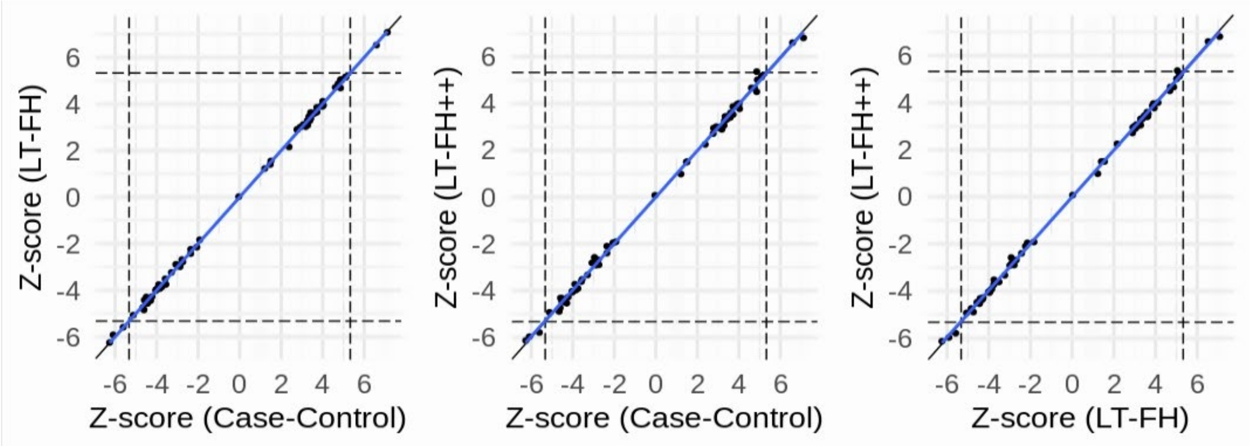

Figure S28: The Z-scores for ADHD for the three outcomes plotted against each other. The dots correspond to LD clumped SNPs that are genome-wide significant in the largest published meta-analysis and present in the iPSYCH cohort (see Methods for details). The blue line indicates the linear regression line between two outcomes and a black line indicates the identity line. The slopes of the regression lines are not significantly different from 1 for any pair of outcomes.

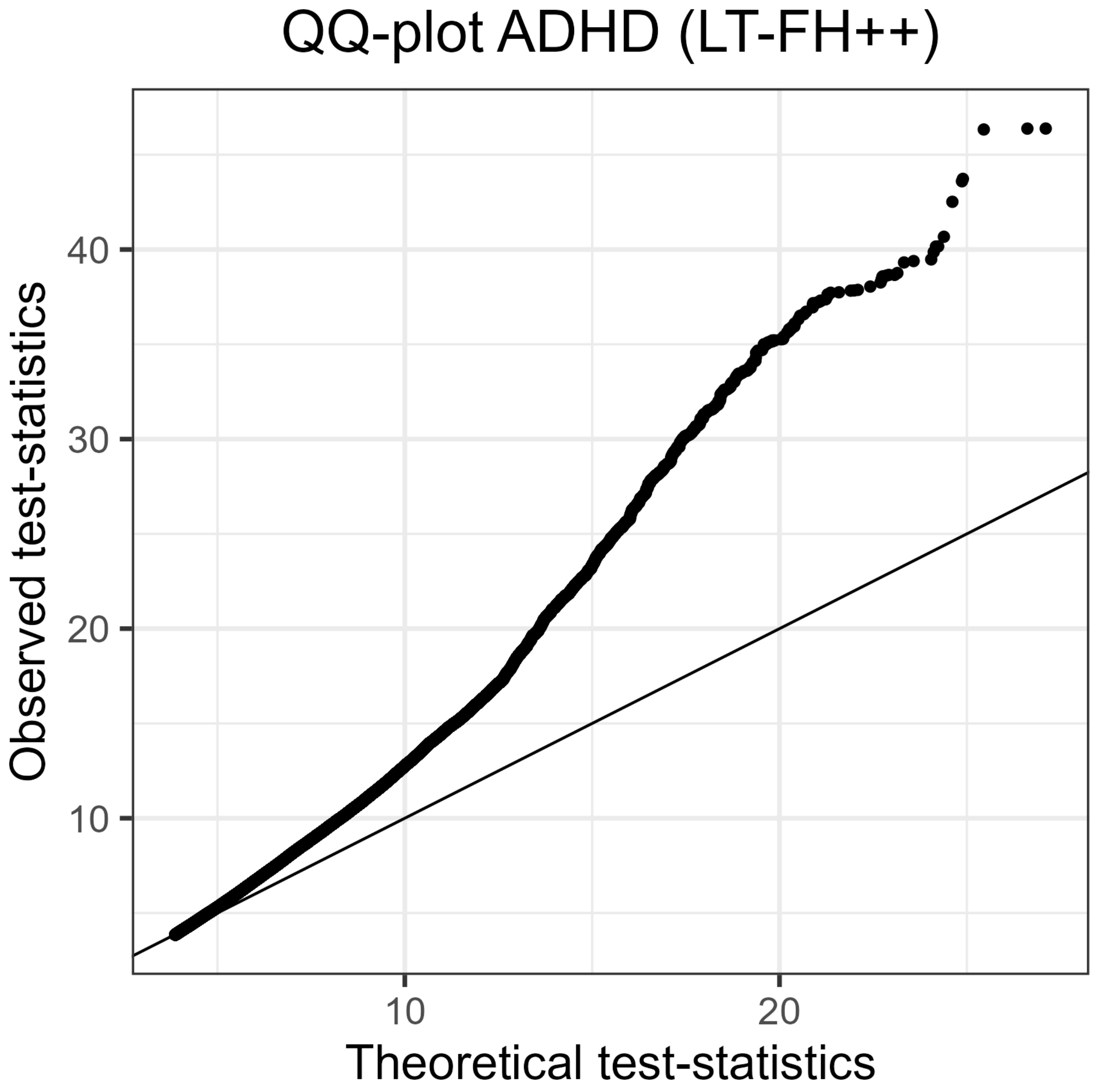

Figure S29: QQ plot of ADHD for LT-FH++. We excluded SNPs with p-values greater than 0.05.

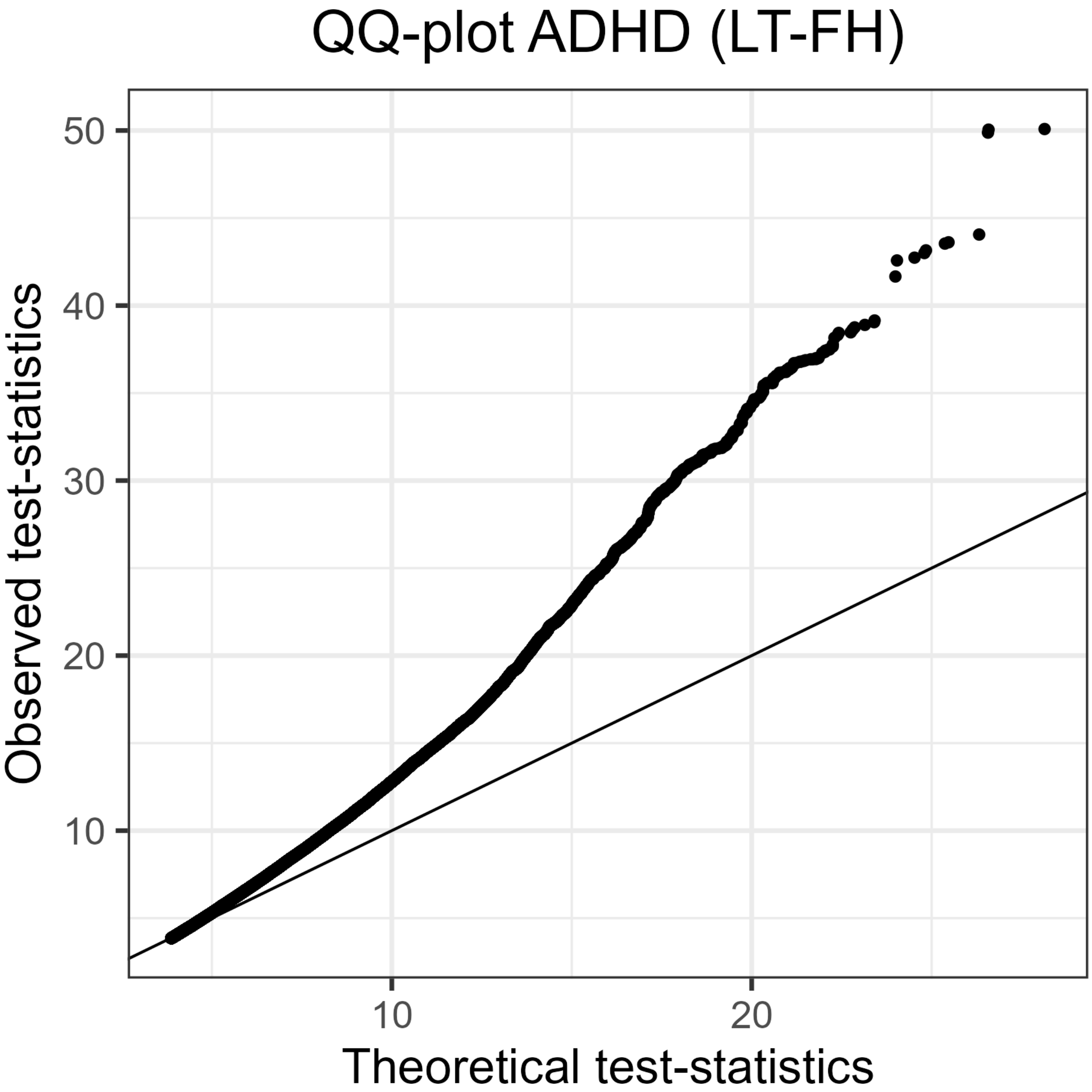

Figure S30: QQ plot of ADHD for LT-FH. We excluded SNPs with p-values greater than 0.05.

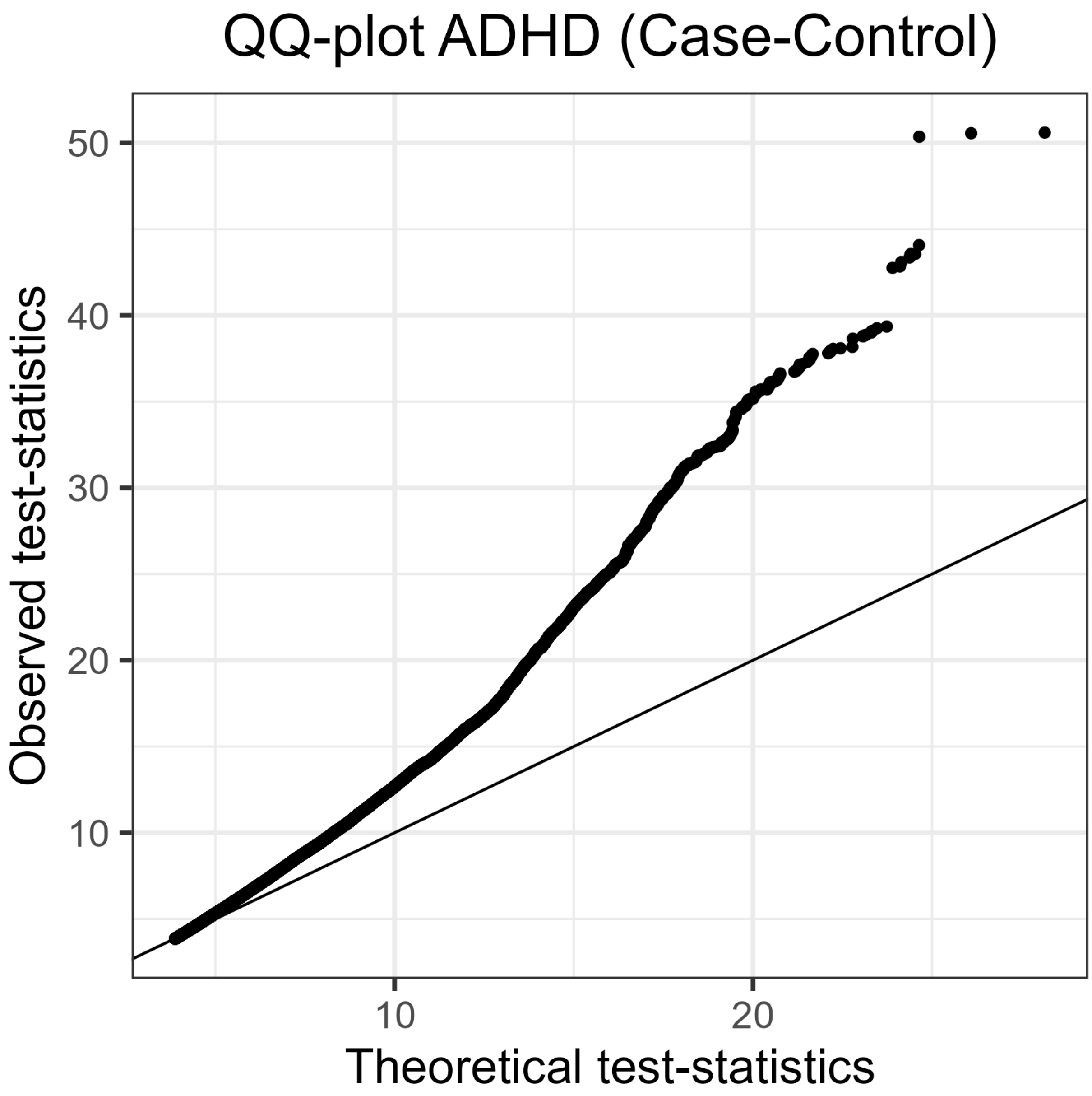

Figure S31: QQ plot of ADHD for case-control status. We excluded SNPs with p-values greater than 0.05.

Autism Spectrum Disorder

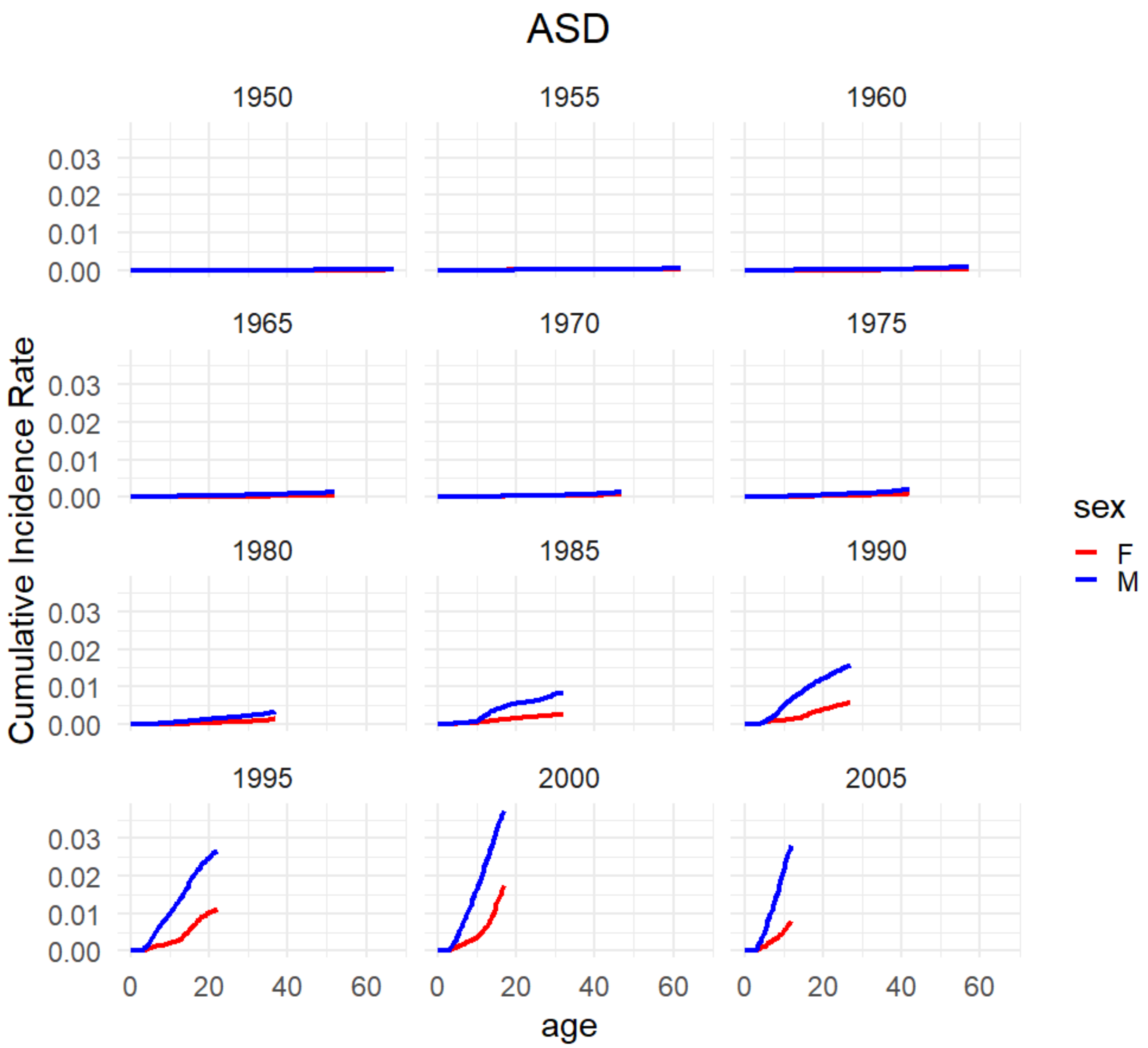

Figure S32: Plot of the cumulative incidence rate for autism spectrum disorder grouped by birth year in the Danish registers. The red line corresponds to females and the blue corresponds to males.

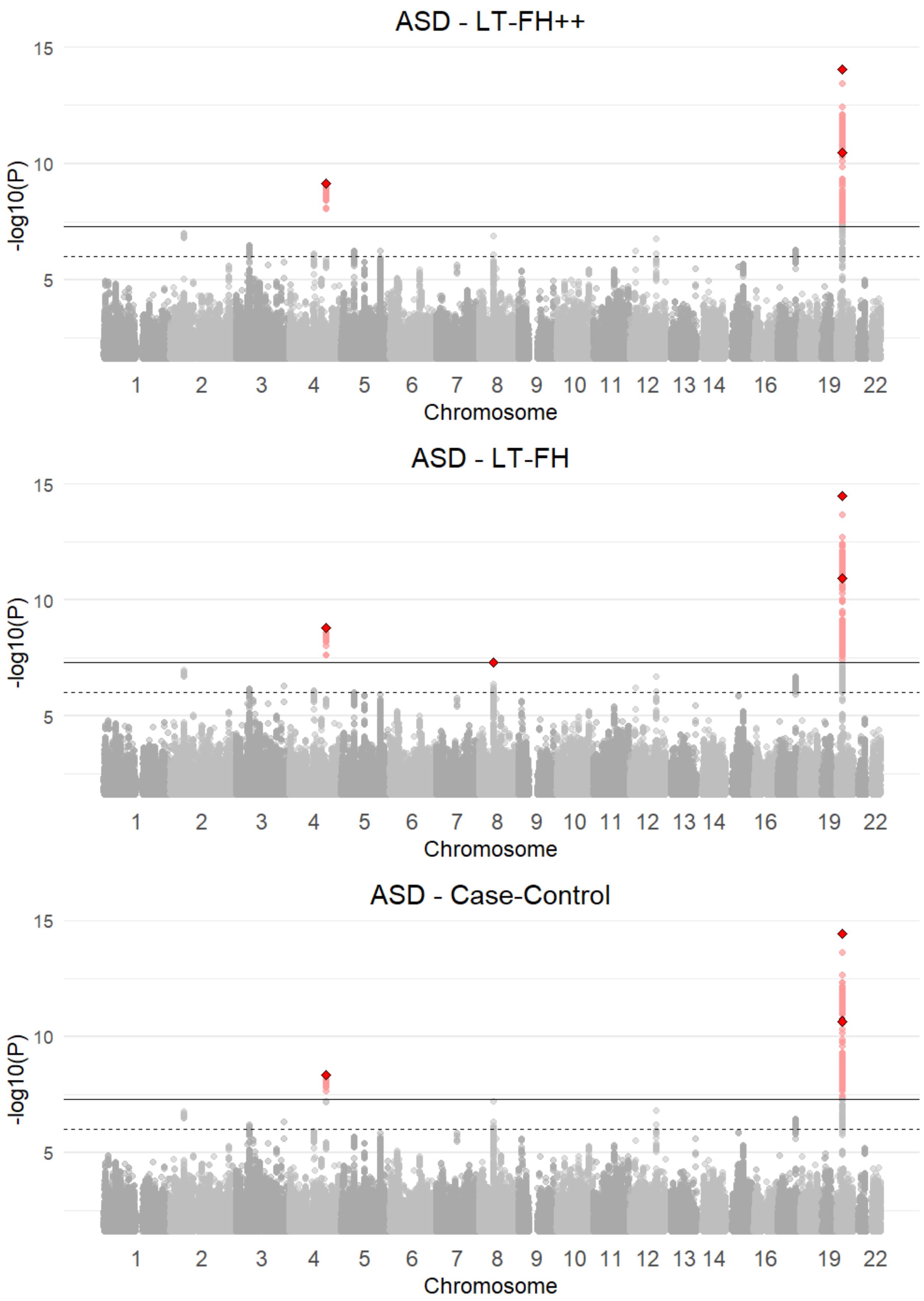
ASD - LT-FH++
15
10
5
-log10(P)
1
2
3
4
5
6
7
8
9
10
11
12
13
14
16
19
22
Chromosome
ASD - LT-FH
15
10
5
-log10(P)
1
2
3
4
5
6
7
8
9
10
11
12
13
14
16
19
22
Chromosome
ASD - Case-Control
15
10
5
-log10(P)
1
2
3
4
5
6
7
8
9
10
11
12
13
14
16
19
22
Chromosome

Figure S33: Manhattan plots for LT-FH++, LT-FH, and case-control GWAS of autism spectrum disorder (ASD) in the iPSYCH cohort. The Manhattan plots display a Bonferroni corrected significance level of 5×10^{-8}, and a suggestive threshold of 5×10^{-6}. The genome-wide significant SNPs are colored in red. The diamonds correspond to top SNPs in a window of size 300k base pairs.

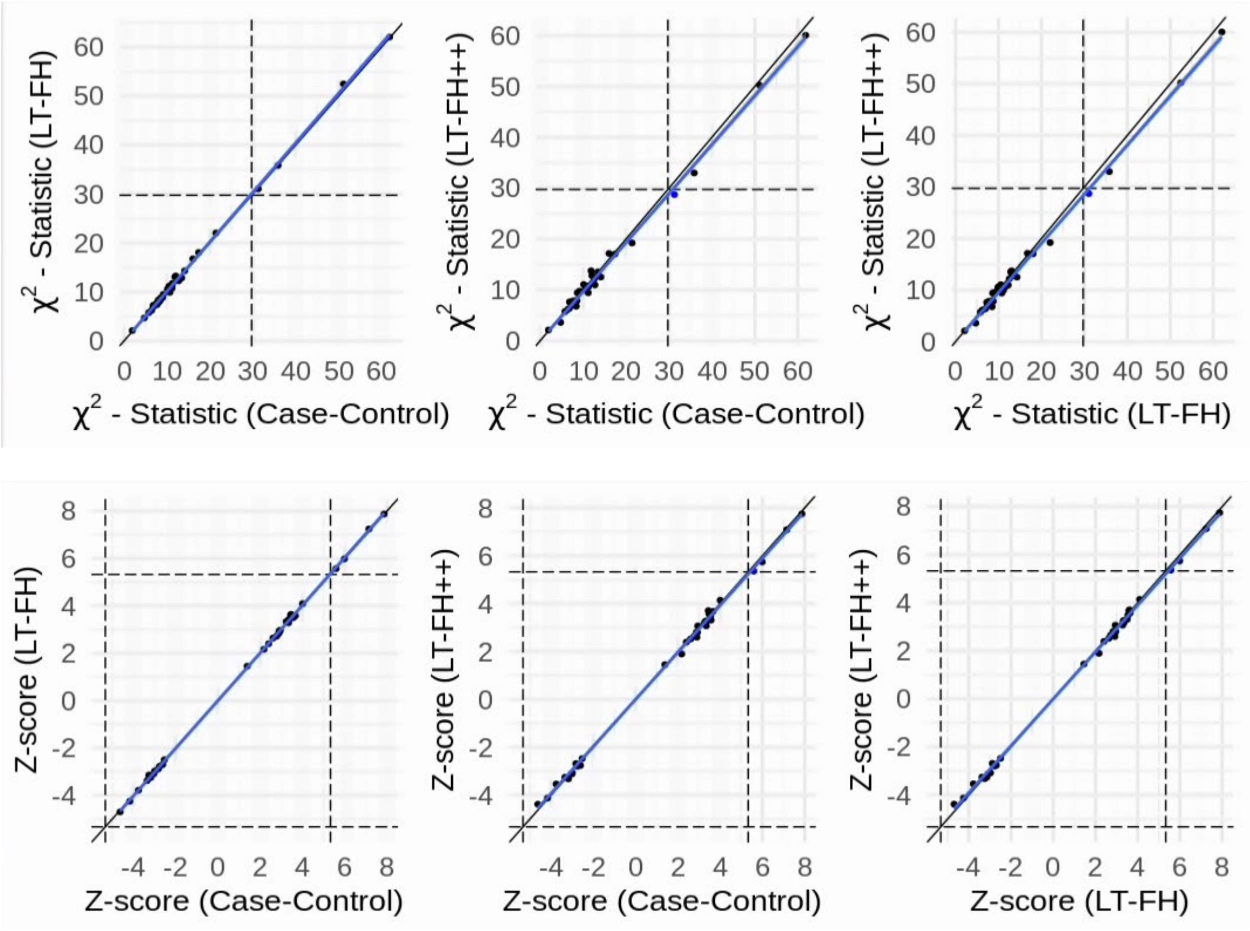

Figure S34: The Z-scores and χ^2 statistics for ASD for the three outcomes plotted against each other. The dots correspond to LD clumped SNPs that are genome-wide significant in the largest published meta-analysis and present in the iPSYCH cohort (see Methods for details). The blue line indicates the linear regression line between two outcomes and a black line indicates the identity line. The slopes of the regression lines are not significantly different from 1 for any pair of outcomes.

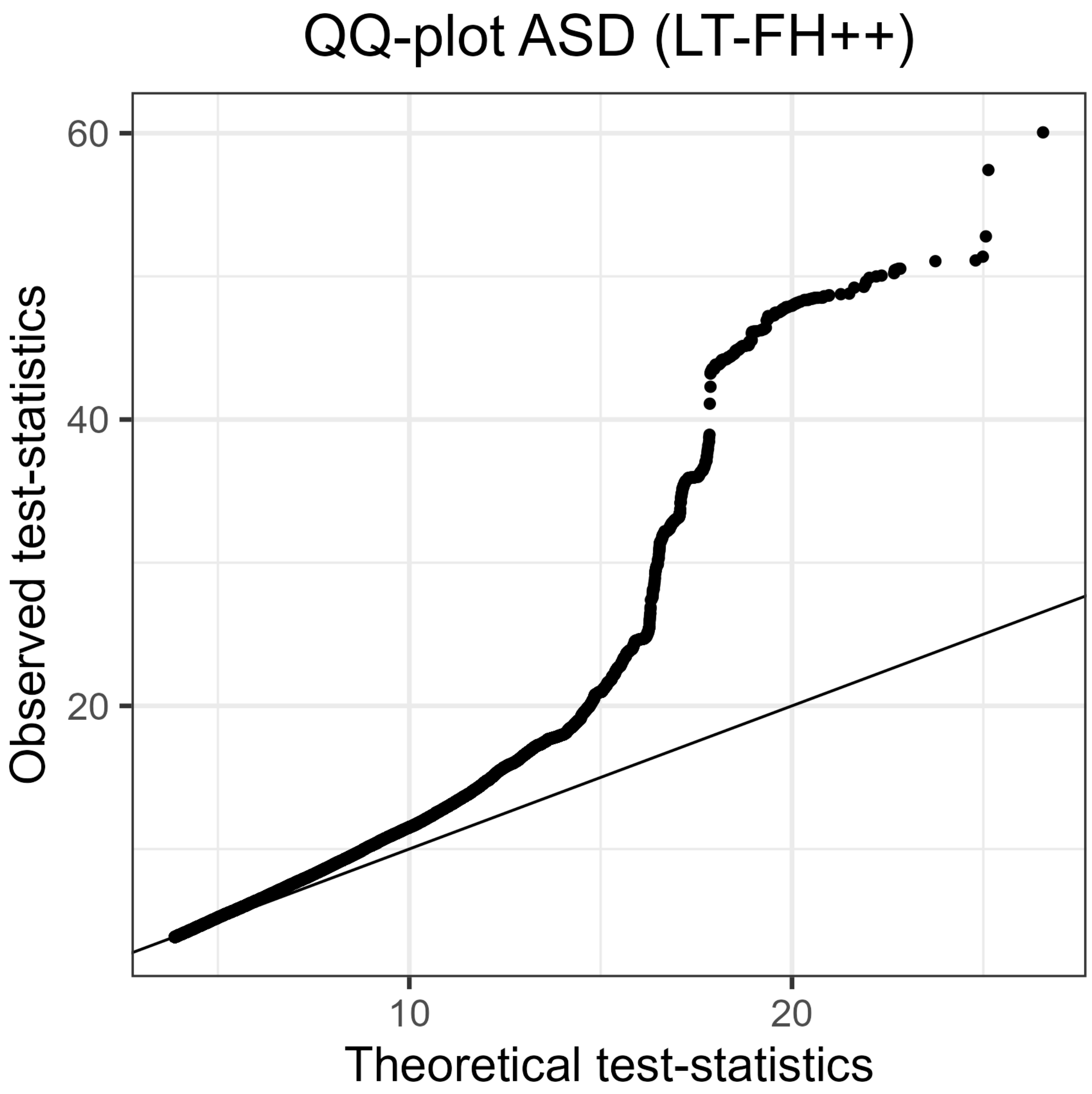

Figure S35: QQ plot of ASD for LT-FH++. We excluded SNPs with p-values greater than 0.05.

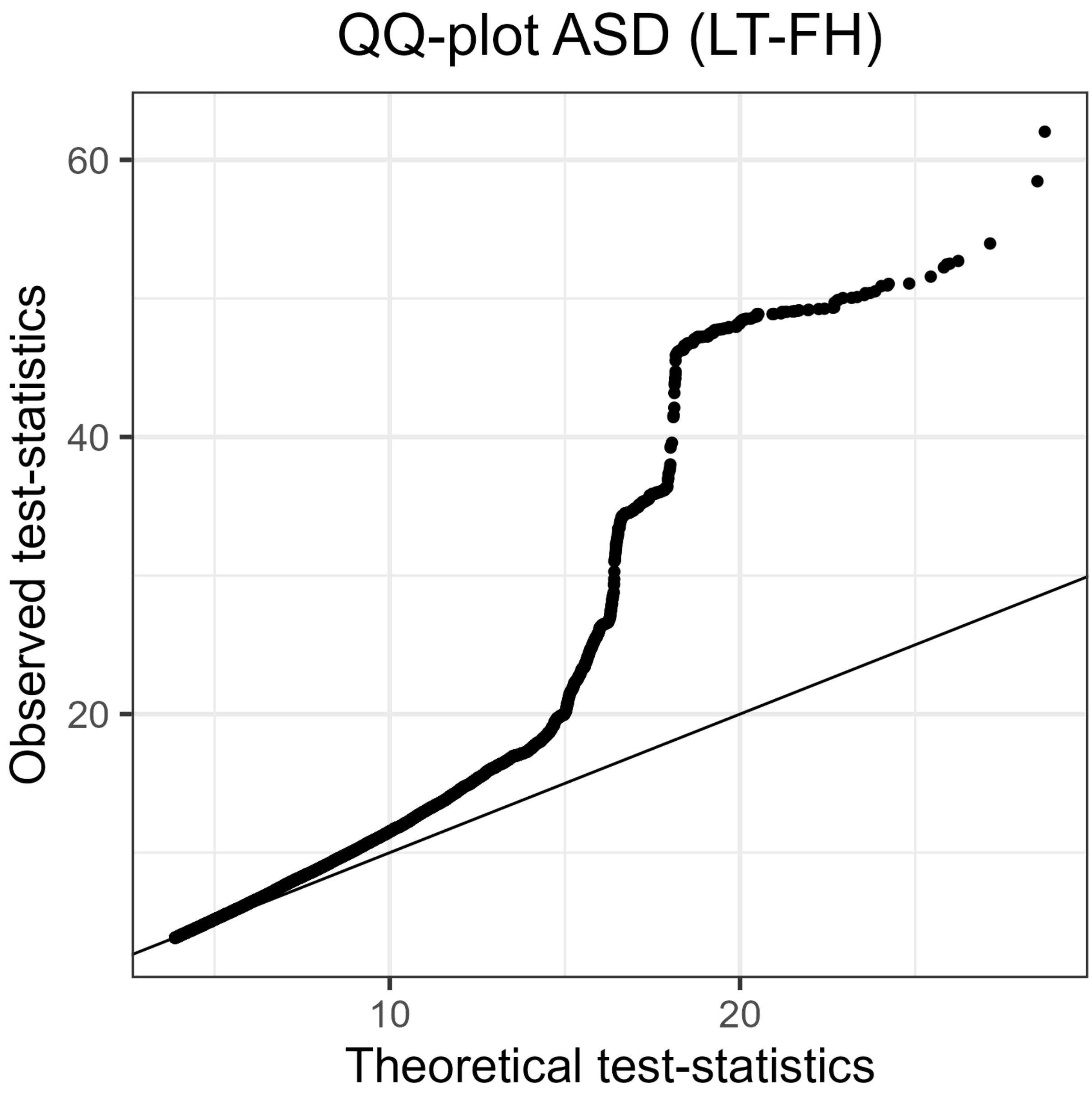

Figure S36: QQ plot of ASD for LT-FH. We excluded SNPs with p-values greater than 0.05.

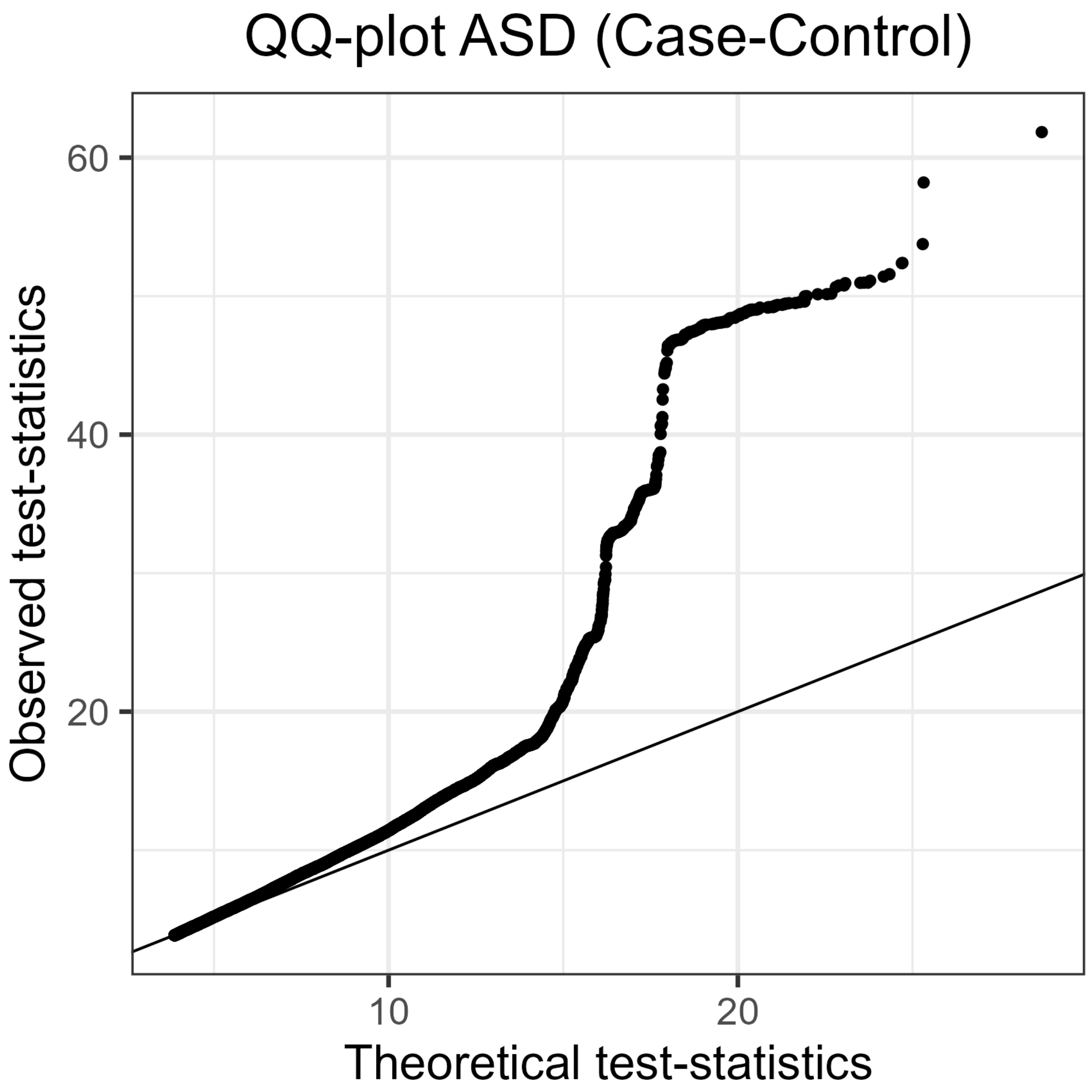

Figure S37: QQ plot of ASD for case-control status. We excluded SNPs with p-values greater than 0.05.

Depression

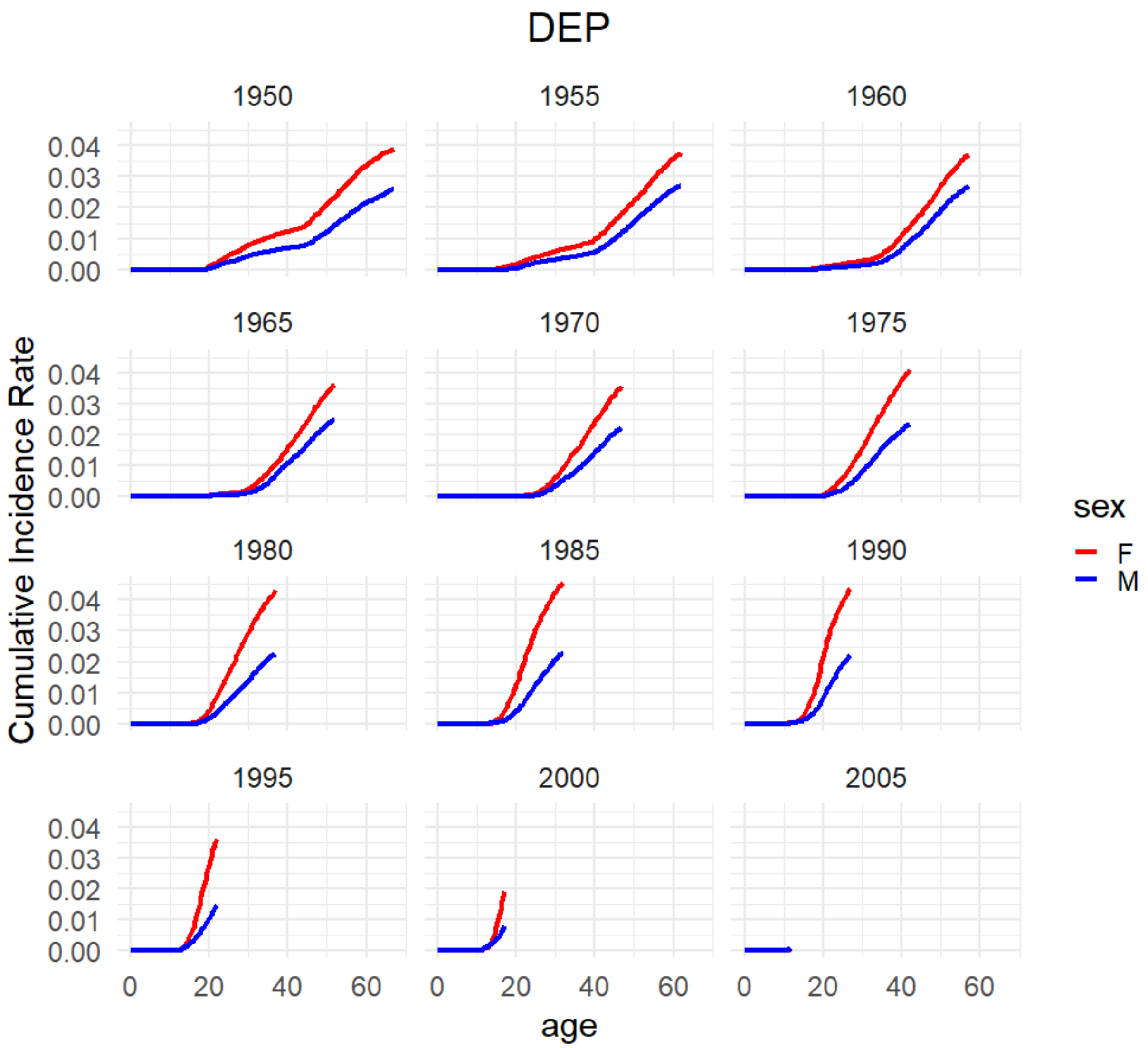

Figure S38: Plot of the cumulative incidence rate for depression grouped by birth year in the Danish registers. The red line corresponds to females and the blue corresponds to males.

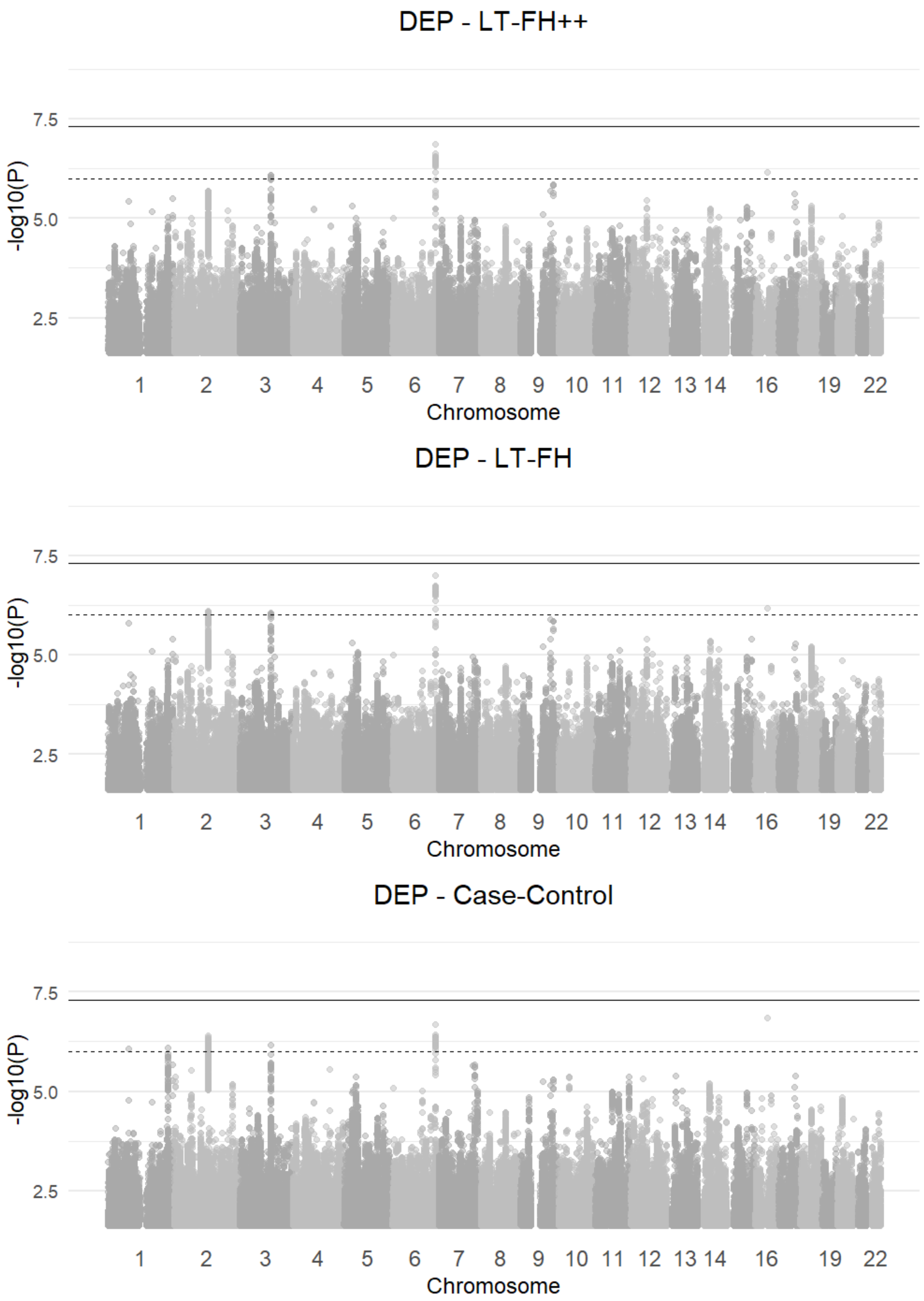
DEP - LT-FH++
7.5
5.0
2.5
-log10(P)
1 2 3 4 5 6 7 8 9 10 11 12 13 14 16 19 22
Chromosome
DEP - LT-FH
7.5
5.0
2.5
-log10(P)
1 2 3 4 5 6 7 8 9 10 11 12 13 14 16 19 22
Chromosome
DEP - Case-Control
7.5
5.0
2.5
-log10(P)
1 2 3 4 5 6 7 8 9 10 11 12 13 14 16 19 22
Chromosome

Figure S39: Manhattan plots for LT-FH++, LT-FH, and case-control GWAS of depression in the iPSYCH cohort. The Manhattan plots display a Bonferroni corrected significance level of 5×10^{-8}, and a suggestive threshold of 5×10^{-6}. The genome-wide significant SNPs are colored in red. The diamonds correspond to top SNPs in a window of size 300k base pairs.

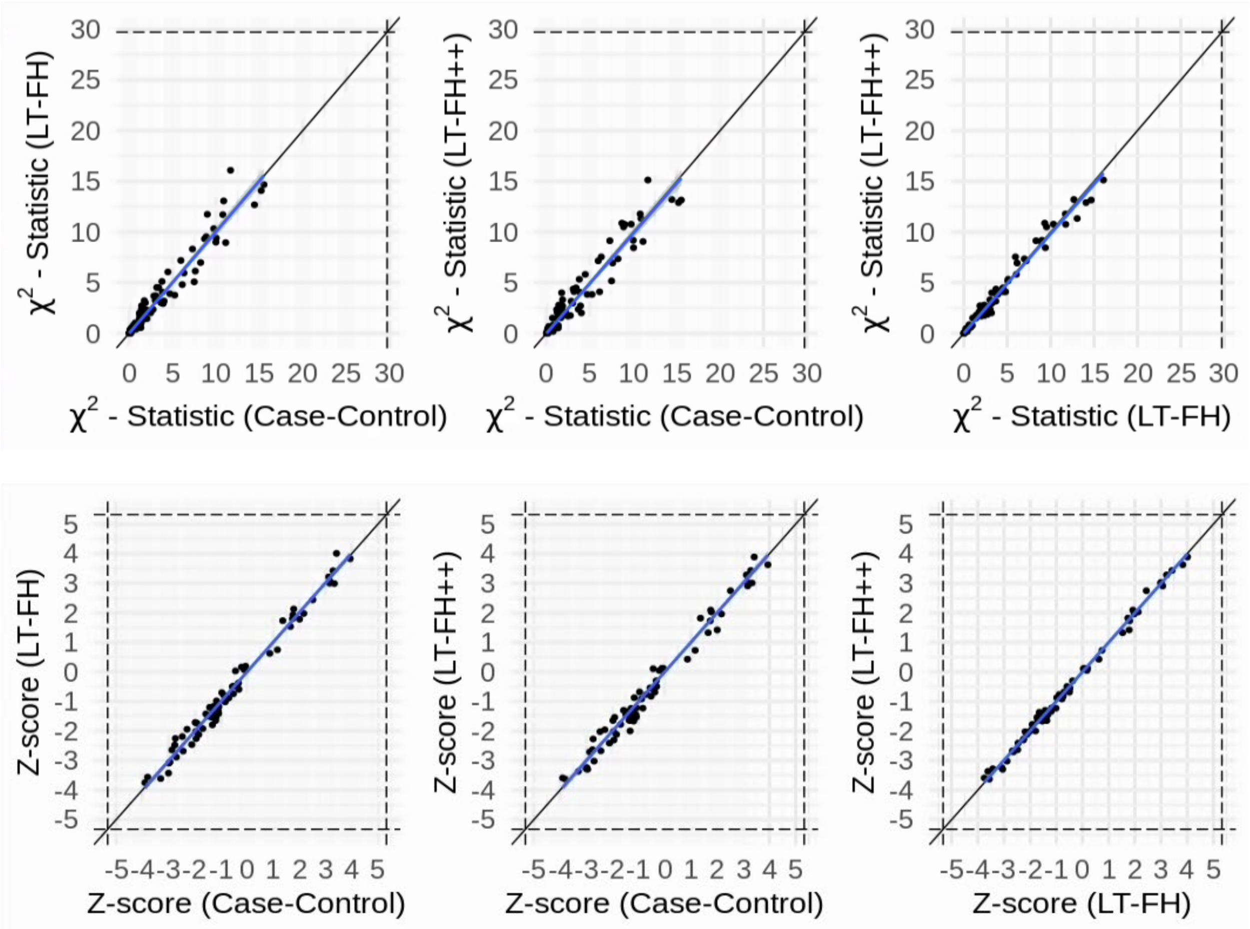

Figure S40: The Z-scores and χ^2 statistics for depression for the three outcomes plotted against each other. The dots correspond to LD clumped SNPs that are genome-wide significant in the largest published meta-analysis and present in the iPSYCH cohort (see Methods for details). The blue line indicates the linear regression line between two outcomes and a black line indicates the identity line. The slopes of the regression lines are not significantly different from 1 for any pair of outcomes.

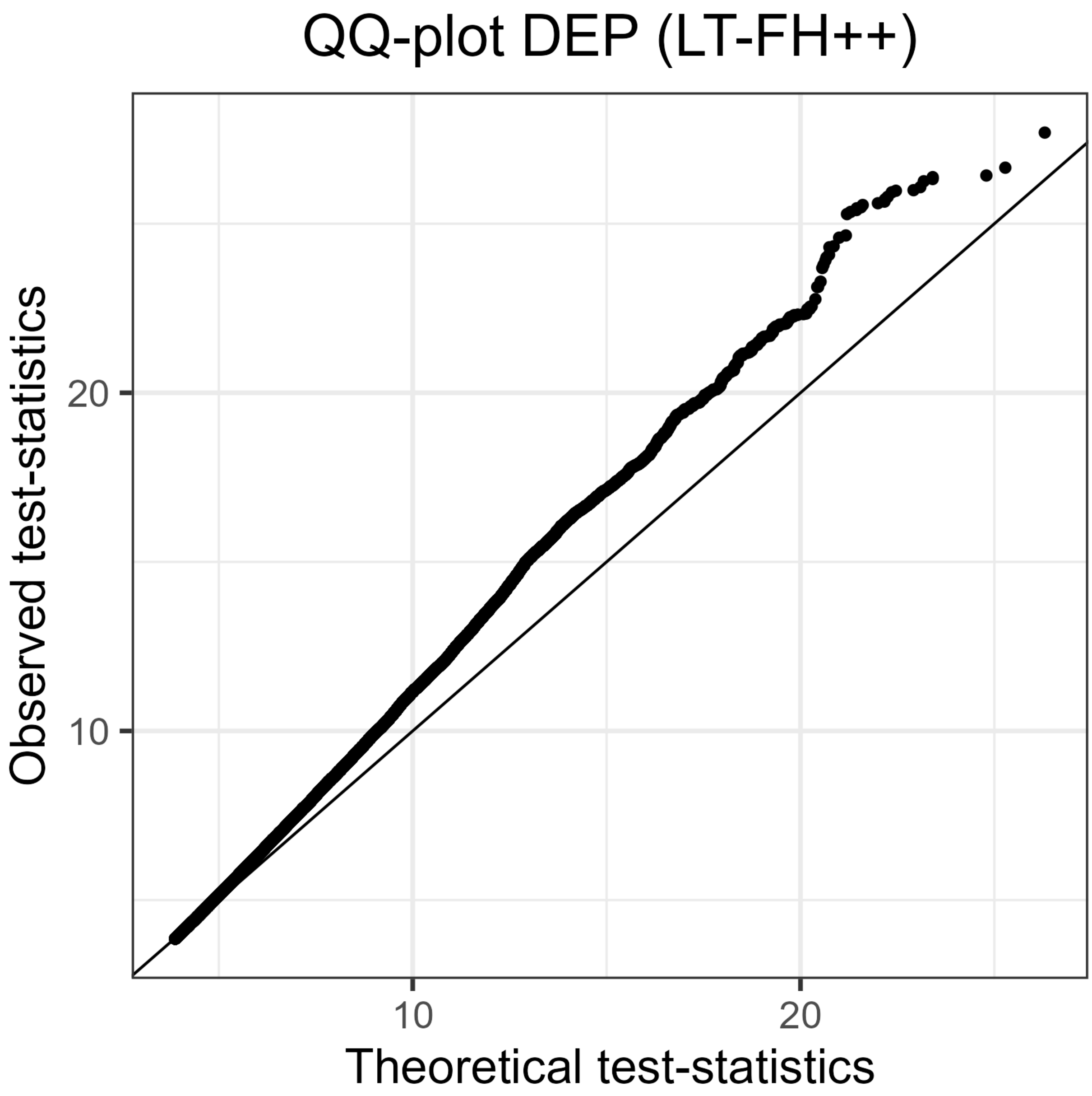

Figure S41: QQ plot of DEP for LT-FH++. We excluded SNPs with p-values greater than 0.05.

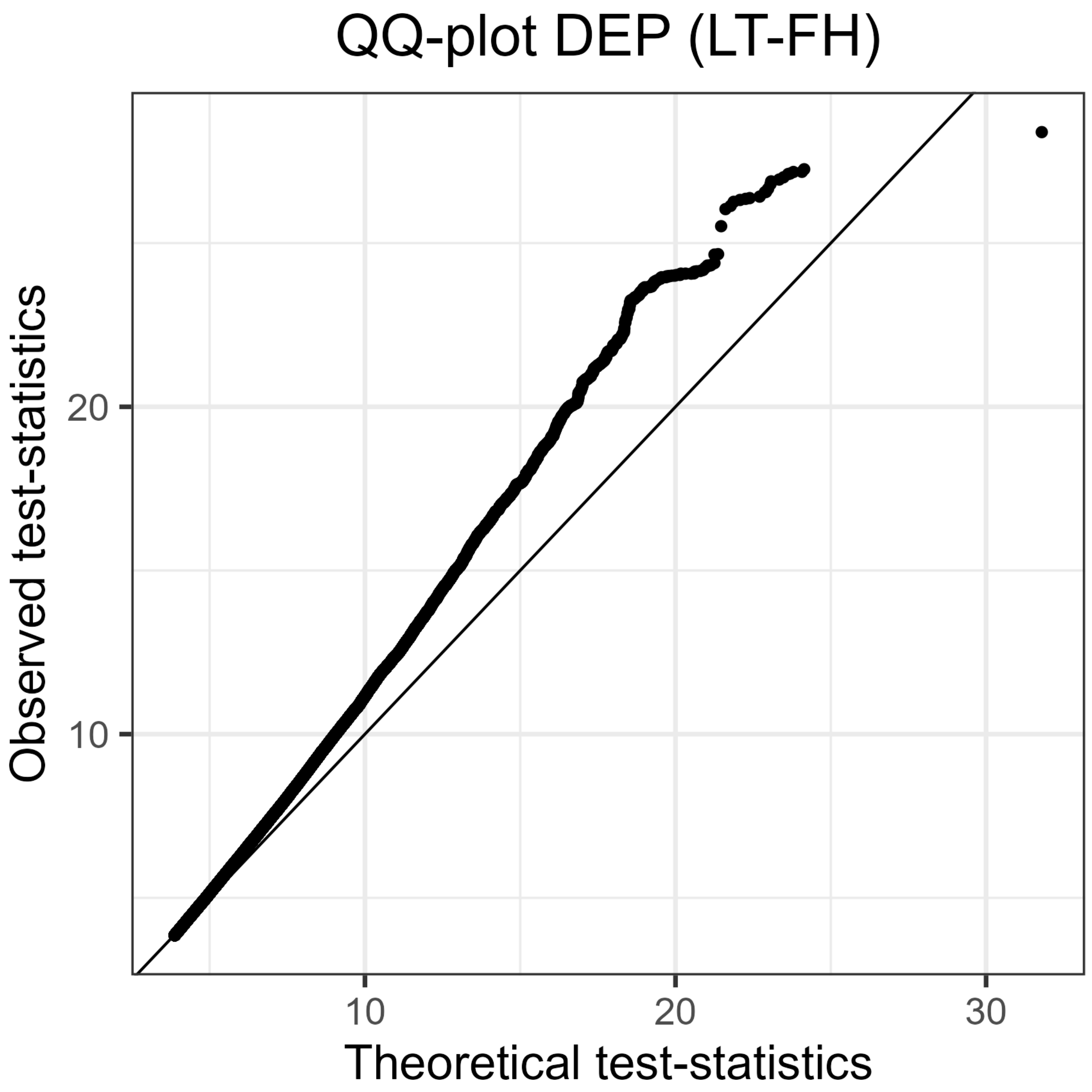

Figure S42: QQ plot of DEP for LT-FH. We excluded SNPs with p-values greater than 0.05.

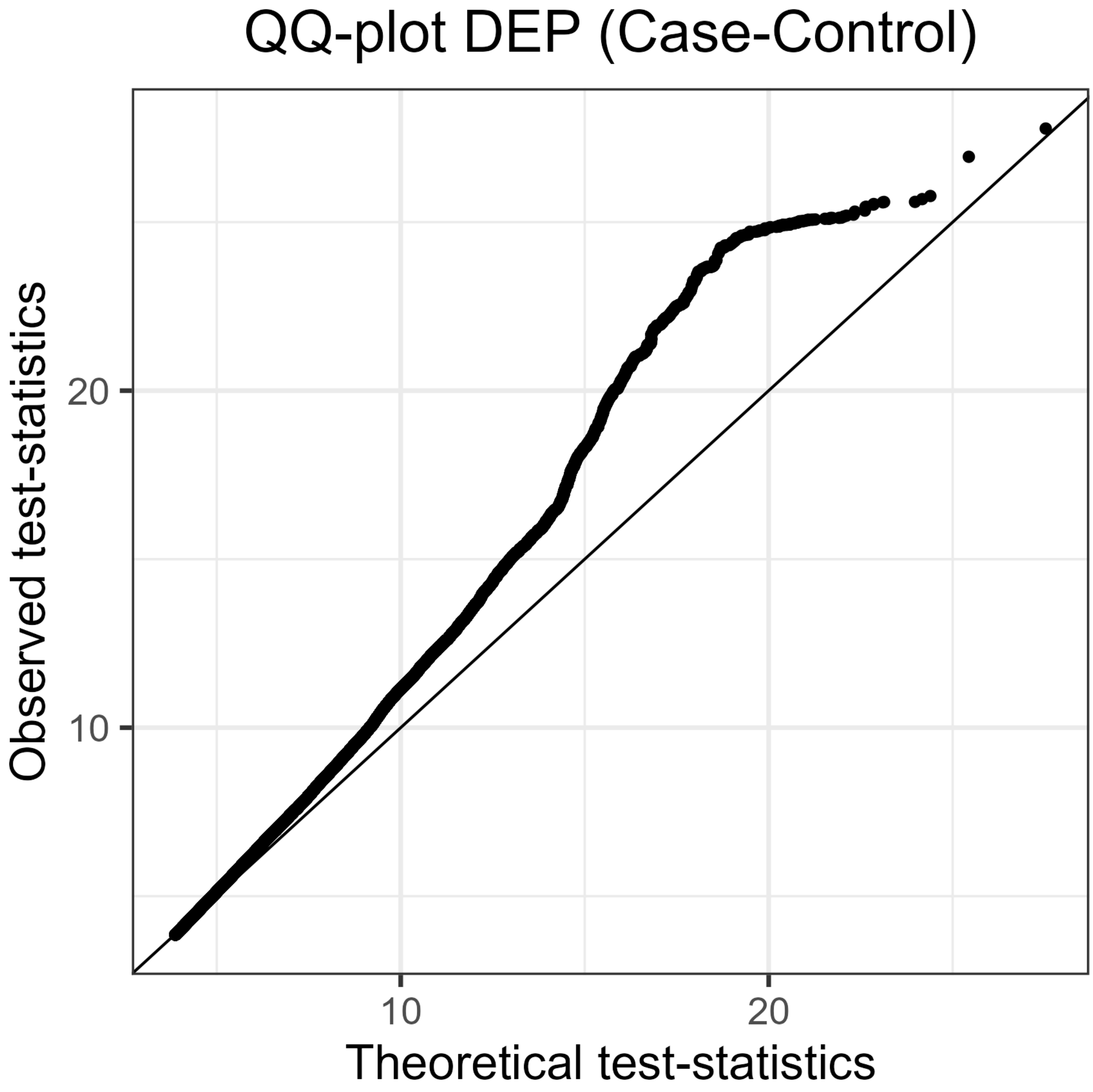

Figure S43: QQ plot of DEP for case-control status. We excluded SNPs with p-values greater than 0.05.

Schizophrenia

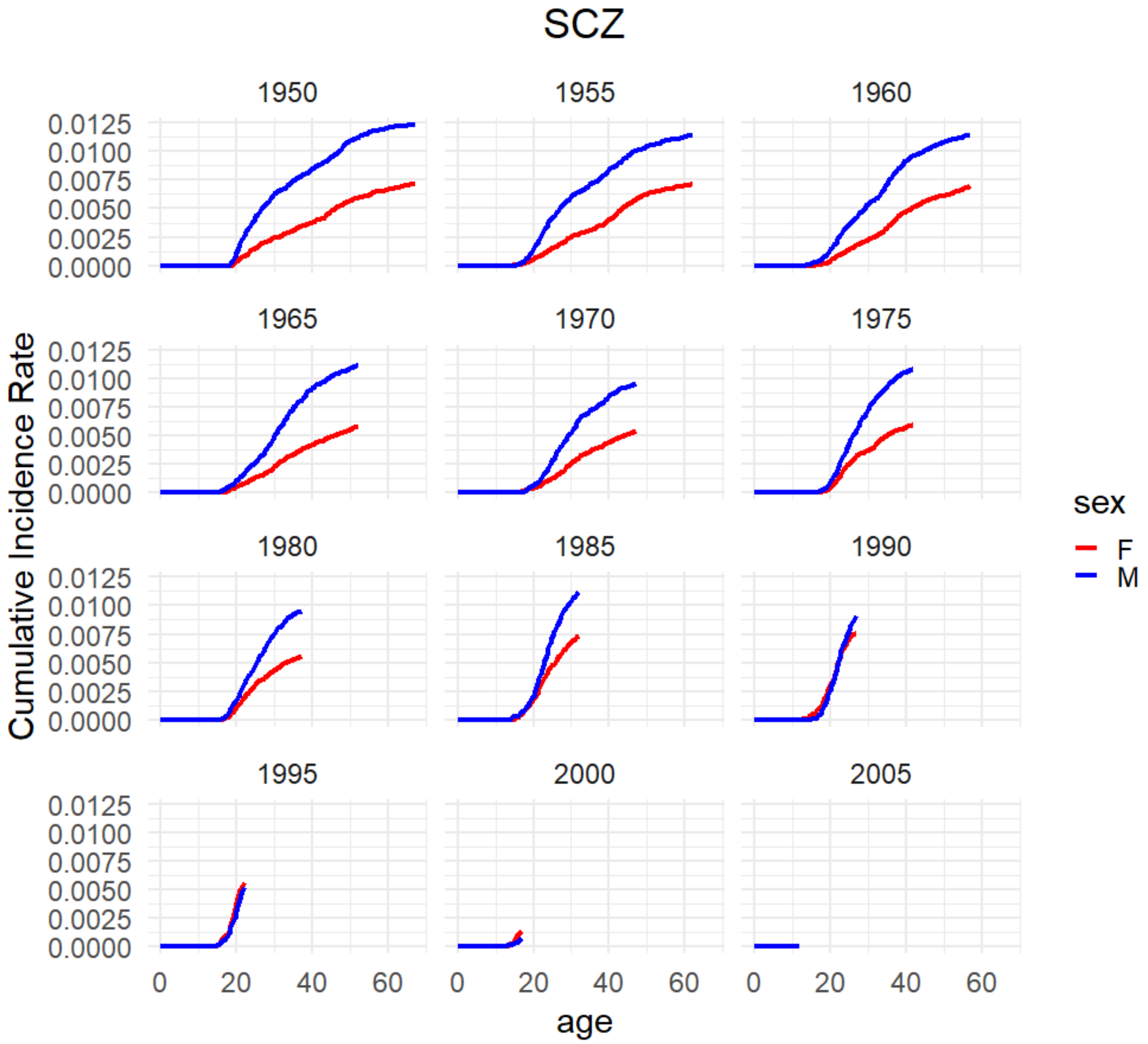

Figure S44: Plot of the cumulative incidence rate for schizophrenia grouped by birth year in the Danish registers. The red line corresponds to females and the blue corresponds to males.

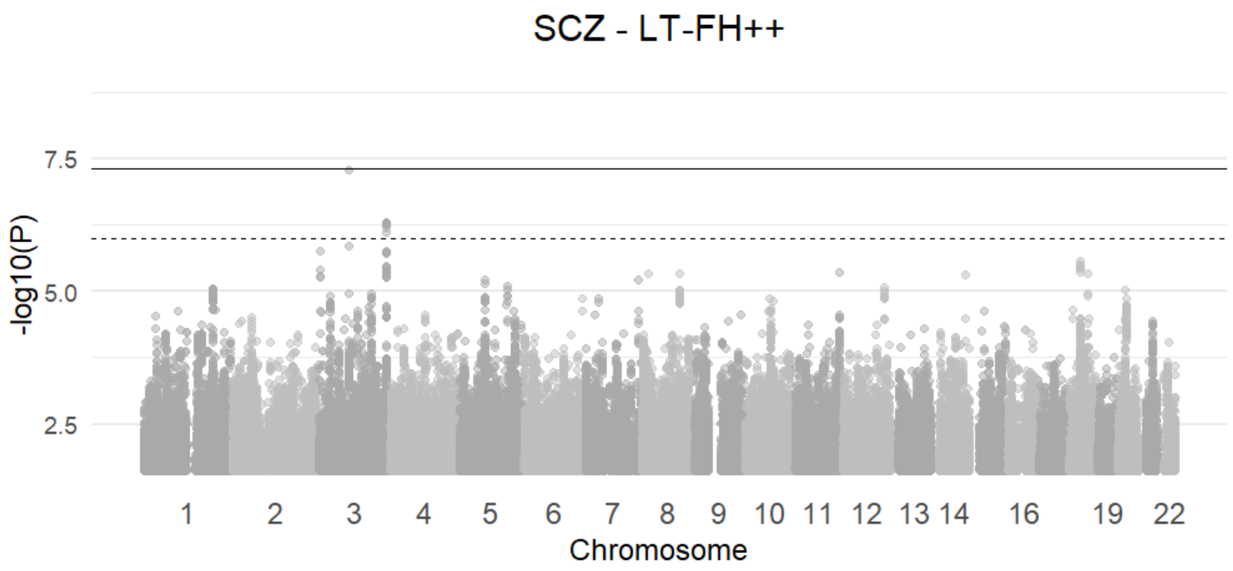
SCZ - LT-FH++
-log10(P)
7.5
5.0
2.5
1
2
3
4
5
6
7
8
9
10
11
12
13
14
16
19
22
Chromosome

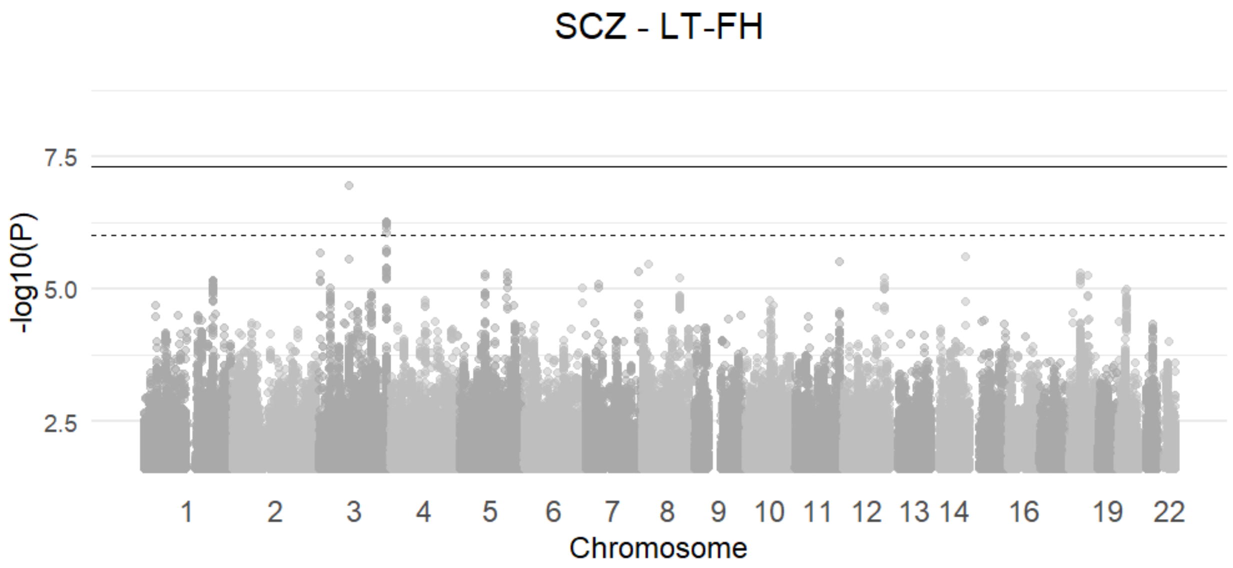
SCZ - LT-FH
-log10(P)
7.5
5.0
2.5
1
2
3
4
5
6
7
8
9
10
11
12
13
14
16
19
22
Chromosome

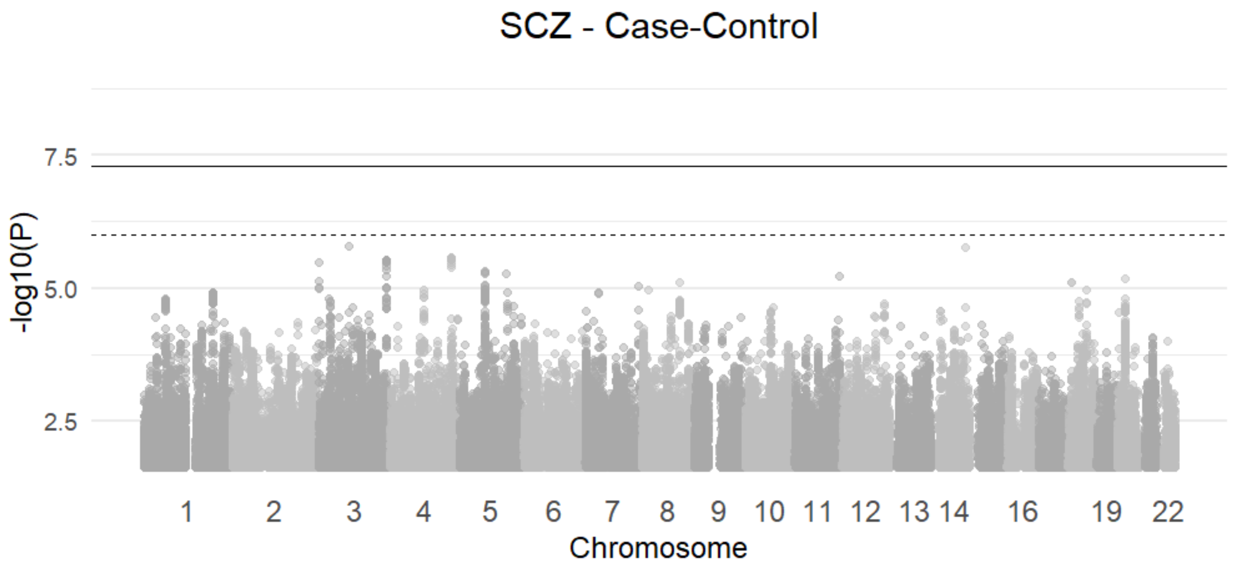
SCZ - Case-Control
-log10(P)
7.5
5.0
2.5
1
2
3
4
5
6
7
8
9
10
11
12
13
14
16
19
22
Chromosome

Figure S45: Manhattan plots for LT-FH++, LT-FH, and case-control GWAS of schizophrenia in the iPSYCH cohort. The Manhattan plots display a Bonferroni corrected significance level of 5×10^{-8}, and a suggestive threshold of 5×10^{-6}. The genome-wide significant SNPs are colored in red. The diamonds correspond to top SNPs in a window of size 300k base pairs.

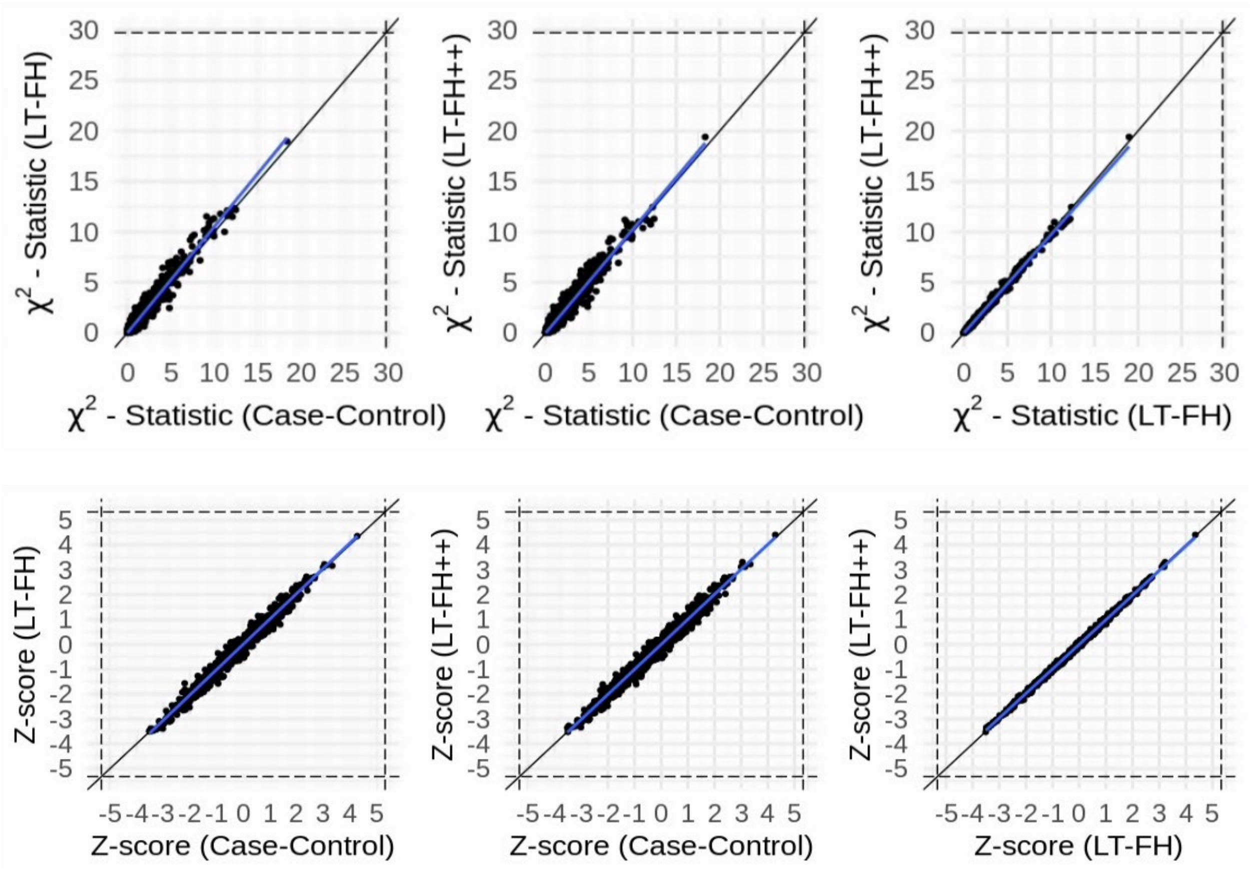

Figure S46: The Z-scores and χ^2 statistics for schizophrenia for the three outcomes plotted against each other. The dots correspond to LD clumped SNPs that are genome-wide significant in the largest published meta-analysis and present in the iPSYCH cohort (see Methods for details). The blue line indicates the linear regression line between two outcomes and a black line indicates the identity line. The slopes of the regression lines are not significantly different from 1 for any pair of outcomes.

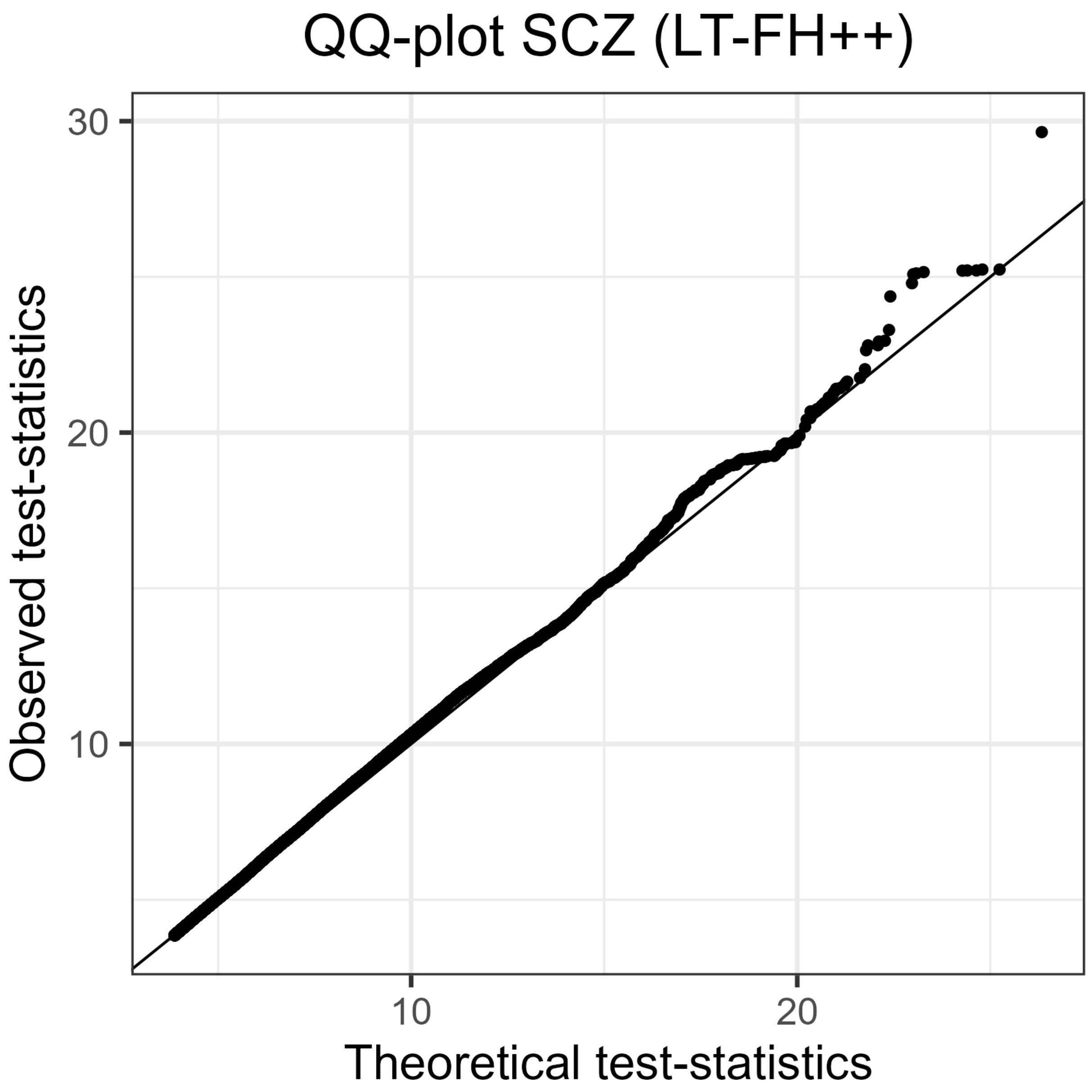

Figure S47: QQ plot of SCZ LT-FH++. We excluded SNPs with p-values greater than 0.05.

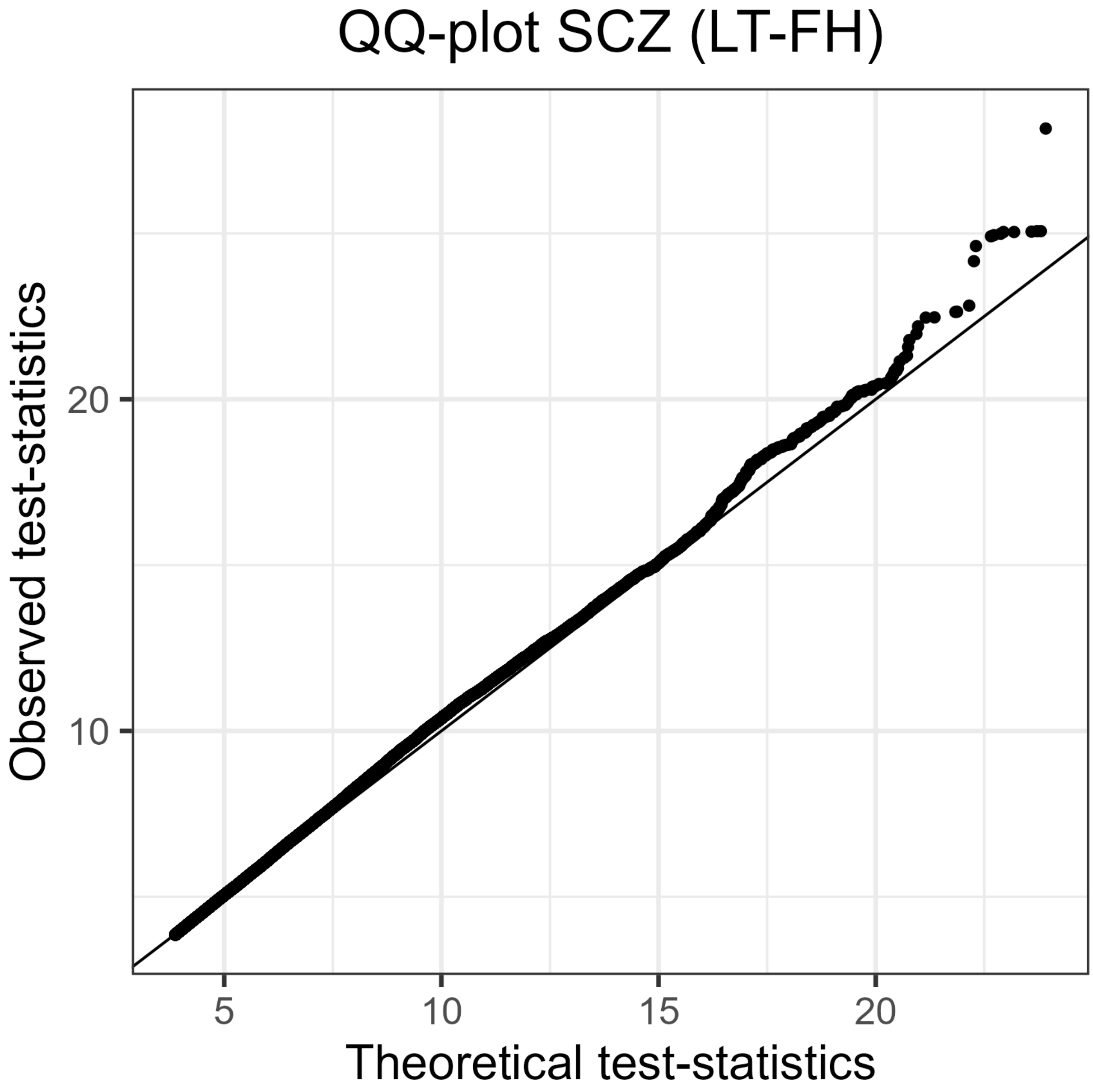

Figure S48: QQ plot of SCZ for LT-FH. We excluded SNPs with p-values greater than 0.05.

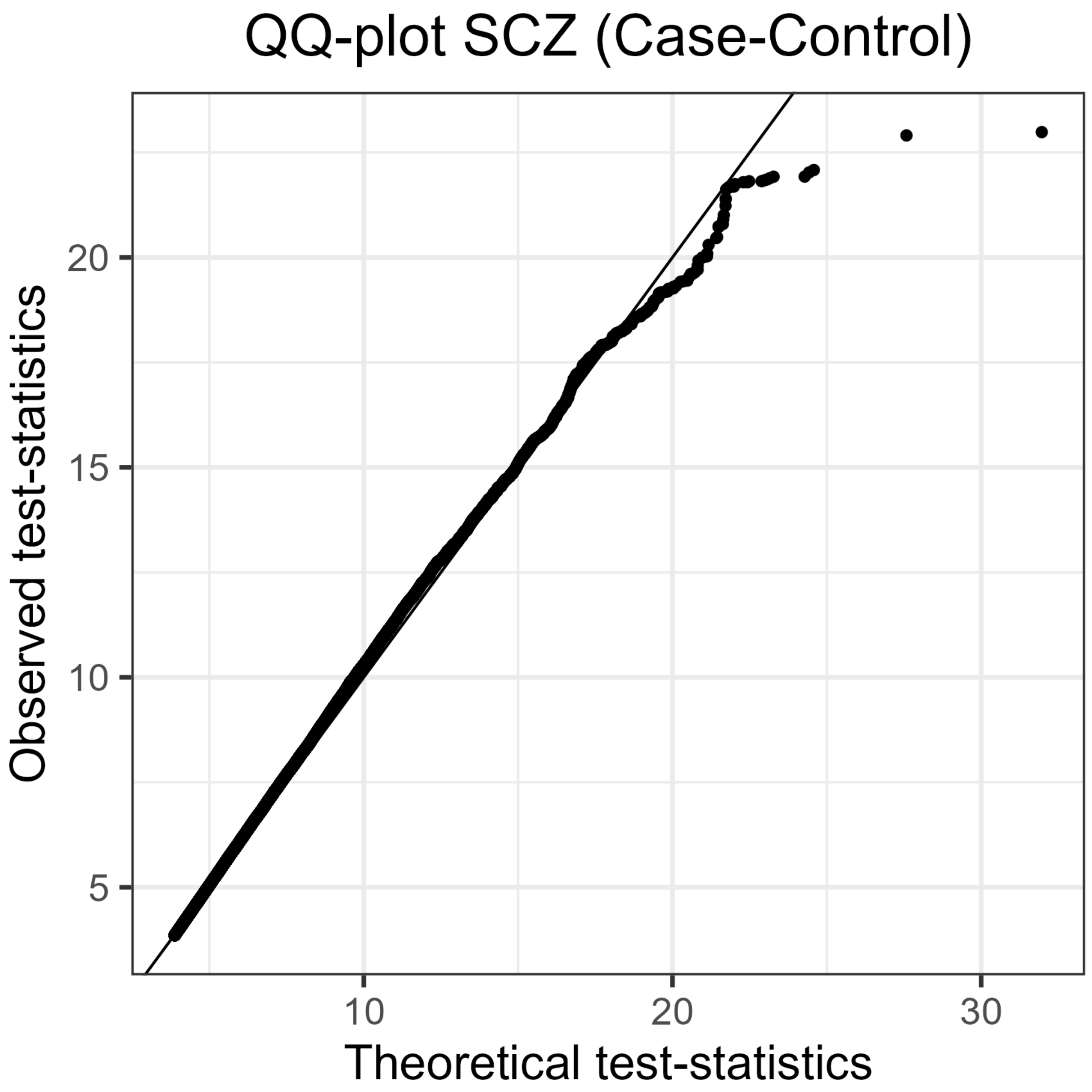

Figure S49: QQ plot of SCZ for case-control status. We excluded SNPs with p-values greater than 0.05.

Time-to-event model

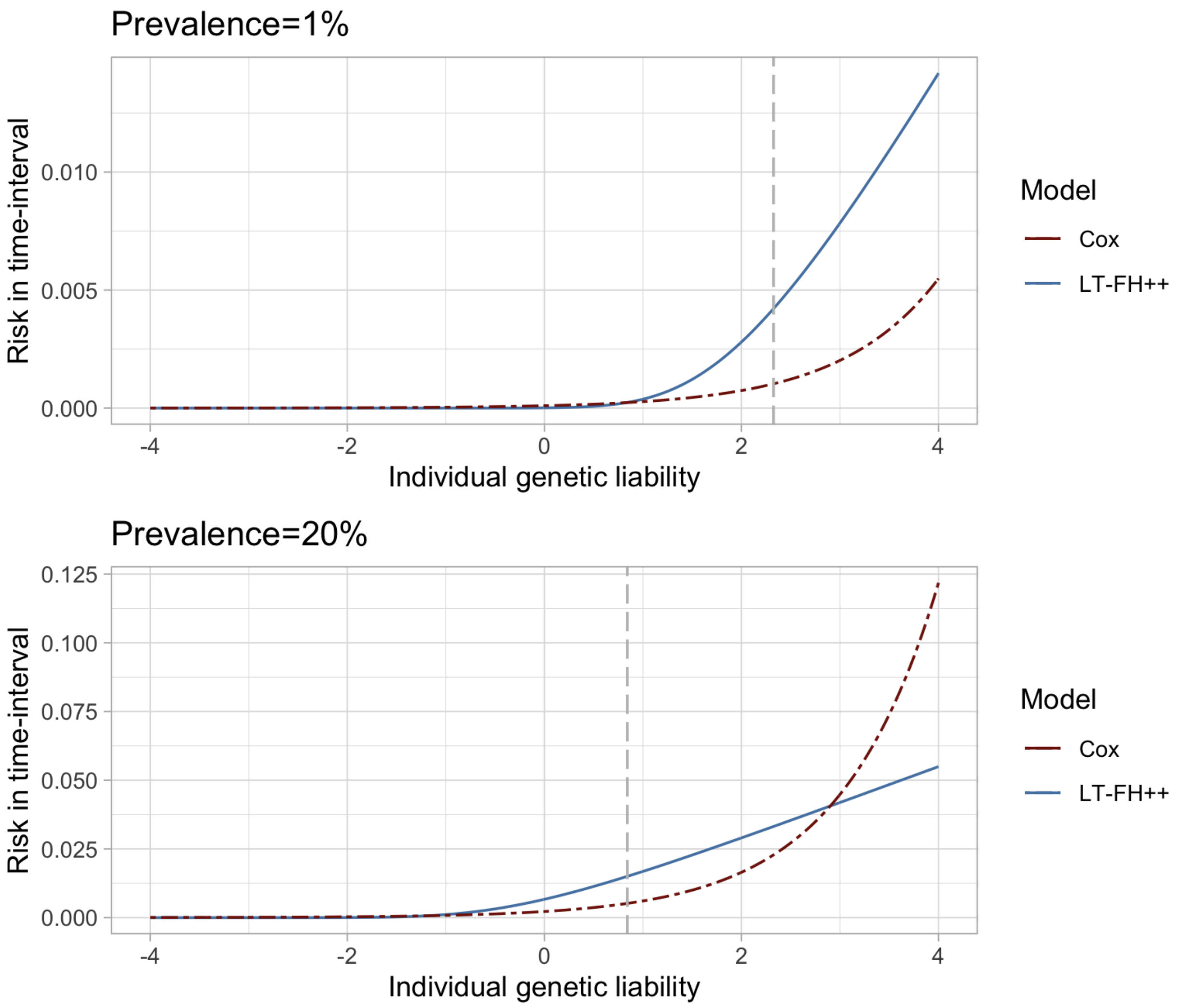

Figure S50: Risk (probability) for becoming a case within a time-interval corresponding to 1% relative increase in prevalence as a function of the genetic liability. The total prevalence changes from 1% and 20% to 1.01% and 20.2% respectively. For Cox regression we assume a constant base incidence rate, corresponding to the prevalence. The vertical dotted grey line denotes the liability threshold corresponding to the prevalence. We note that the risk for becoming a case within a small time-interval is proportional to the hazard rate.

Supplemental Tables

In this section we will include supplementary tables. We have split the tables into results from the simulations and results from the real-world analysis.

Simulation Results

Power & chi-square statistics

downsampling	Method	Power	Power sd	Mean causal chisq	Mean causal chisq sd	Mean null chisq	Mean null chisq sd
No	GWAS	0.1032	0.00711493	11.0968799	0.43115084	0.99897719	0.00578427
No	GWAX	0.1231	0.00546606	12.5786011	0.4423601	0.99990076	0.00449278
No	LT-FH	0.1594	0.0090701	15.0321414	0.55502615	0.99931755	0.00428873
No	LT-FH++	0.1659	0.00769488	15.4442868	0.55315674	0.9999296	0.00423999
Yes	GWAS	0.0315	0.00538	6.39052221	0.14904845	0.99977481	0.00449538

Yes	GWAX	0.0326	0.00474225	6.29082337	0.16595288	0.99942523	0.00382606
Yes	LT-FH	0.0376	0.00636309	6.74523086	0.16838193	0.99971582	0.00422892
Yes	LT-FH++	0.0436	0.00638053	7.18764144	0.18546156	0.99969847	0.00427345

Table S1: Table containing simulation results for the default simulation setup with a prevalence of 5%.

downsampling	Method	Power	Power sd	Mean causal chisq	Mean causal chisq sd	Mean null chisq	Mean null chisq sd
No	GWAS	0.1722	0.01050714	15.8921563	0.53905794	0.99877984	0.00315812
No	GWAX	0.1825	0.0134433	16.7631072	0.48298124	1.0003692	0.00452109
No	LT-FH	0.2335	0.01162612	21.2427511	0.65085209	0.9991315	0.00352166
No	LT-FH++	0.2444	0.01220382	22.1870996	0.64715594	1.00009105	0.00312604
Yes	GWAS	0.0752	0.00657943	9.33170612	0.17869466	0.99945213	0.00251433
Yes	GWAX	0.0702	0.00694102	8.88419642	0.17602146	1.00085604	0.00278447
Yes	LT-FH	0.0929	0.00597123	10.3183013	0.18243892	1.00009436	0.00268586
Yes	LT-FH++	0.1086	0.00471876	11.4003675	0.21725906	0.99952079	0.00294178

Table S2: Table containing simulation results for the default simulation setup with a prevalence of 10%.

Method	Mean causal chisq	Mean causal chisq sd	Power	Power sd	Mean null chisq	Mean null chisq sd
GWAS	31.2035947	3.257629924	0.3285	0.013938356	1.002499879	0.005953325
GWAX	38.88355922	3.743794854	0.3791	0.00807534	1.000536754	0.005106242
LT-FH	45.22722295	4.623442789	0.4145	0.009046178	1.001252174	0.003933444
LT-FH++	47.0349021	4.831365713	0.4227	0.008857514	1.00116158	0.004565413

Table S3: Table containing the mean chi-square test statistic for the causal and null snps, as well as the power. The table contains these values for N = 300,000, 5% prevalence, no downsampling, and full family history and age-of-onset information.The other parameter setups can be found in the supplementary data, and include 2 different prevalences, 4 different values of N, 4 different levels of completeness of family history and age-of-onset information.

Method	Mean causal chisq	Mean causal chisq sd	Power	Power sd	Mean null chisq	Mean null chisq sd
GWAS	45.69678239	4.422692924	0.4195	0.012385027	1.000089338	0.008232819
GWAX	52.84787089	4.169667408	0.4549	0.013461468	0.999932281	0.006525725
LT-FH	64.87177976	5.687542538	0.4989	0.015701734	1.000850998	0.008694631
LT-FH++	69.00093357	6.287391807	0.5095	0.013826303	1.001138582	0.007489548

Table S4: Table containing the mean chi-square test statistic for the causal and null snps, as well as the power. The table contains these values for N = 300,000, 10% prevalence, no downsampling, and full family history and age-of-onset information. The other parameter setups can be found in the supplementary data, and include 2 different prevalences, 4 different values of N, 4 different levels of completeness of family history and age-of-onset information.

False positive rates

Method	Alpha level	Proportion of False positives	Standard error
GWAS	0.000005	5.0505E-06	3.8671E-06
GWAS	0.00005	5.6566E-05	2.3664E-05
GWAS	0.0005	0.00053131	7.3058E-05
GWAS	0.005	0.0050303	0.0002248
GWAS	0.05	0.04988889	0.0006919
GWAX	0.000005	5.0505E-06	4.7546E-06
GWAX	0.00005	4.2424E-05	1.9987E-05
GWAX	0.0005	0.00048232	6.9577E-05
GWAX	0.005	0.00505202	0.00022527
GWAX	0.05	0.05031414	0.00069471

LT-FH	0.000005	6.0606E-06	5.4689E-06
LT-FH	0.00005	4.9495E-05	2.1687E-05
LT-FH	0.0005	0.00049697	7.0602E-05
LT-FH	0.005	0.00493434	0.00022266
LT-FH	0.05	0.04987929	0.00069187
LT-FH++	0.000005	5.0505E-06	4.4588E-06
LT-FH++	0.00005	5.2525E-05	2.1524E-05
LT-FH++	0.0005	0.00049545	7.0385E-05
LT-FH++	0.005	0.00495253	0.00022305
LT-FH++	0.05	0.04985859	0.00069173

Table S5: Table of the false positive rate at varying levels of significance thresholds in the default simulation setup with a prevalence of 5%.

Method	Alpha level	Proportion of False positives	Standard error
GWAS	0.000005	5.0505E-06	5.0505E-06
GWAS	0.00005	5.2525E-05	2.2498E-05
GWAS	0.0005	0.00046465	6.8385E-05
GWAS	0.005	0.00504646	0.00022517
GWAS	0.05	0.04989293	0.00069196
GWAX	0.000005	6.0606E-06	5.173E-06
GWAX	0.00005	5.7071E-05	2.3661E-05
GWAX	0.0005	0.00051111	7.1703E-05
GWAX	0.005	0.00503889	0.00022499
GWAX	0.05	0.0498697	0.0006918

LT-FH	0.000005	2.5253E-06	2.5252E-06
LT-FH	0.00005	6.1616E-05	2.4492E-05
LT-FH	0.0005	0.00049596	7.0626E-05
LT-FH	0.005	0.00507475	0.0002258
LT-FH	0.05	0.05006364	0.00069308
LT-FH++	0.000005	3.5354E-06	3.5353E-06
LT-FH++	0.00005	5.303E-05	2.2861E-05
LT-FH++	0.0005	0.00051263	7.179E-05
LT-FH++	0.005	0.00501919	0.00022455
LT-FH++	0.05	0.04988535	0.00069191

Table S6: Table of the false positive rate at varying levels of significance thresholds in the default simulation setup with a prevalence of 5% and downsampling of controls.

Method	Alpha level	Proportion of False positives	Standard error
GWAS	0.000005	4.0404E-06	3.4487E-06
GWAS	0.00005	6.2626E-05	2.4478E-05
GWAS	0.0005	0.00047071	6.8846E-05
GWAS	0.005	0.00484949	0.00022073
GWAS	0.05	0.04968586	0.0006906
GWAX	0.000005	7.0707E-06	5.8386E-06
GWAX	0.00005	4.3939E-05	1.9716E-05
GWAX	0.0005	0.00050303	7.0968E-05
GWAX	0.005	0.00499848	0.00022406
GWAX	0.05	0.05002525	0.00069283

LT-FH	0.000005	3.5354E-06	2.9436E-06
LT-FH	0.00005	4.899E-05	2.0835E-05
LT-FH	0.0005	0.00048939	7.0106E-05
LT-FH	0.005	0.00487525	0.00022135
LT-FH	0.05	0.04968333	0.00069058
LT-FH++	0.000005	8.0808E-06	6.8487E-06
LT-FH++	0.00005	4.596E-05	2.0976E-05
LT-FH++	0.0005	0.00048535	6.9909E-05
LT-FH++	0.005	0.00494747	0.00022295
LT-FH++	0.05	0.04999091	0.00069261

Table S7: Table of the false positive rate at varying levels of significance thresholds in the default simulation setup with a prevalence of 10%.

Method	Alpha level	Proportion of False positives	Standard error
GWAS	0.000005	1.1111E-05	9.9276E-06
GWAS	0.00005	4.3434E-05	2.0597E-05
GWAS	0.0005	0.0005101	7.1635E-05
GWAS	0.005	0.00507879	0.00022588
GWAS	0.05	0.04976263	0.0006911
GWAX	0.000005	7.0707E-06	6.1831E-06
GWAX	0.00005	6.2121E-05	2.456E-05
GWAX	0.0005	0.00052374	7.2577E-05
GWAX	0.005	0.00510808	0.00022654
GWAX	0.05	0.05011818	0.00069344

LT-FH	0.000005	6.5657E-06	5.9739E-06
LT-FH	0.00005	4.9495E-05	2.1948E-05
LT-FH	0.0005	0.00052222	7.2523E-05
LT-FH	0.005	0.00512071	0.00022682
LT-FH	0.05	0.04984899	0.00069167
LT-FH++	0.000005	9.596E-06	7.4763E-06
LT-FH++	0.00005	5.5556E-05	2.2965E-05
LT-FH++	0.0005	0.0005	7.091E-05
LT-FH++	0.005	0.00501616	0.00022451
LT-FH++	0.05	0.04996465	0.00069242

Table S8: Table of the false positive rate at varying levels of significance thresholds in the default simulation setup with a prevalence of 10% and downsampling of controls.

Method	Alpha level	Proportion of False positives	Standard error
GWAS	0.000005	5.0505E-06	3.7697E-06
GWAS	0.00005	4.3434E-05	2.0434E-05
GWAS	0.0005	0.00050505	7.1238E-05
GWAS	0.005	0.00494949	0.000223
GWAS	0.05	0.04972828	0.00069088
GWAX	0.000005	3.0303E-06	2.4386E-06
GWAX	0.00005	4.5455E-05	2.1107E-05
GWAX	0.0005	0.00050505	7.1313E-05
GWAX	0.005	0.00494545	0.00022291
GWAX	0.05	0.05018384	0.00069387

LT-FH	0.000005	2.0202E-06	2.0202E-06
LT-FH	0.00005	5.1515E-05	2.2289E-05
LT-FH	0.0005	0.00049293	7.0392E-05
LT-FH	0.005	0.0050101	0.00022436
LT-FH	0.05	0.05006162	0.00069307
LT-FH++	0.000005	5.0505E-06	3.0303E-06
LT-FH++	0.00005	4.8485E-05	2.1632E-05
LT-FH++	0.0005	0.00049192	7.031E-05
LT-FH++	0.005	0.00504848	0.00022521
LT-FH++	0.05	0.0501798	0.00069384

Table S9: Table containing the false positive rates with varying levels of alpha level for each of the considered methods with N = 300,000, 5% prevalence, no downsampling, and full family history and age-of-onset information. The other parameter setups can be

found in the supplementary data, and include 2 different prevalences, 4 different values of N, 4 different levels of completeness of family history and age-of-onset information.

Significant associations - Mortality

Variant ID	Chromosome :Position (hg38)	LT-FH++ P-value	Effect size (SE)	Nearest gene	Selected previously reported associations
rs429358	19:44908684	8.8e-52	-0.176493(0.0116573)	APOE	Alzheimer's[7], metabolic traits[80], mortality[60,70]
15:78828640	15:78828640	1.9e-22	0.088522 (0.00908256)	HYKK	Smoking and lung cancer[6], mortality[70]
rs10455872	6:160589086	7.5e-15	-0.120683 (0.0155212)	LPA	heart disease, mortality[70]
6:161075384	6: 161075384	5.1e-14	-0.243674(0.0323606)	MAP3K4	Endometriosis[81]
rs34386495	6:32658953	4.7e-10	0.0664307(0.0106654)	HLA-DQB1	Asthma[82], autoimmune diseases[83],

					mortality[70]
rs61905747	11:113769120	8.5e-9	-0.0620208(0.0107705)	ZW10	Glioma[84] mortality[70,85]
rs2507989	6:31356638	1.6e-8	-0.0592997(0.0104863)	HLA-B	White blood cell count[62], Psoriasis[63]
rs3838008	20:63357289-63357318 (indel)	1.9e-8	0.0608869 (0.0108248)	CHRNA4	Smoking and lung cancer[6], mortality[70]
rs17691989	13:77093116	4.4e-8	-0.1571(0.028695)	MYCBP2	Circadian rhythm (chronotype)[64]
rs79339645	3:166883110	4.7e-8	0.120294(0.0220177)	ZBBX	DNA methylation in older people[66]

Table S10: Independent LT-FH++ associations for mortality in UK biobank identified using COJO[61] and sorted by lowest p-value. The two strongest associations are shared with LT-FH, and seven out of three were previously identified in association studies of longevity[85] or parental age[70].

Significant associations - iPSYCH

Variant ID	Chromosome :Position (hg38)	LT-FH++ P-value	Effect size (SE)	Nearest gene	Selected previously reported associations
rs56022653	5:88588020	5.8e-12	0.132154(0.191985)	LINC00461	Educational attainment[68], ADHD[10,86]
rs11210887	1:43610348	1.1e-11	0.133962(0.0203968)	PTPRF	Smoking initiation[6], Educational attainment[68], ADHD[10,86]
rs9969232	7:114518899	2.1e-9	-0.120184(0.0200724)	FOXP2	Risk taking[87], ADHD[10]
rs6082363	20:21270205	5.0e-9	0.122019(0.0208684)	ZNF877P	ASD[9,88]
rs11030386	11:28609701	3.7e-8	-0.106526(0.0193581)	LINC02758	ADHD[10]

rs4261436	14:32830276	4.3e-8	-0.103069(0.0188137)	AKAP6	Cognitive traits[67,68]
rs7026534	9:134907263	4.7e-8	0.111291(0.0203778)		Education attainment, Smoking initiation [6,68]

Table S11: Independent LT-FH++ genome-wide significant associations for ADHD using COJO[61] and sorted by lowest p-value.

Variant ID	Chromosome :Position (hg38)	LT-FH++ P-value	Effect size (SE)	Nearest gene	Selected previously reported associations
rs910805	20:21248116	9.6e-15	0.194518 (0.0251149)	ZNF877P, AL117332.1	ASD[9]
rs4274907	4:135863730	7.7e-10	0.173381 (0.0281911)	LOC105377437	None reported

Table S12: Independent LT-FH++ genome-wide significant associations for ASD using COJO[61] and sorted by lowest p-value.

Table S13: Excel file containing all simulation results on power, mean causal and null chi-square test statistics, as well as their standard deviations. Furthermore, information on false positive rates in simulations are included for different significance levels (alpha levels), and the numbers from the run time simulations of LT-FH++.

Method	prev	downsampling	Symmetry test	Paired t-test	Wilcoxon Signed rank test	Paired Mcnemar
LT-FH++	10%	No	0	0.000160	0.00592	0
LT-FH++	10%	Yes	0	0.00000179	0.00586	0
LT-FH++	5%	No	0	0.0000208	0.00554	0
LT-FH++	5%	Yes	0	0.00000854	0.00563	0

Table S14: Table containing tests between LT-FH and LT-FH++ for significant differences. Symmetry test corresponds to a test for independence in a contingency table. The table contains the sum of all causal SNPs detected across all 10 simulations for each method in the first row and the sum of all undetected in the second. The paired t-test corresponds to a t-test on the average power across all 10 simulations with each group being a method. Wilcoxon signed rank test corresponds to a non-parametric test for difference in location between two data sets. Paired Mcnemar is a paired test for independence in a contingency table. All parameter setups showed that there was a significant difference between the number of SNPs found by LT-FH++ compared to LT-FH.

Method	prev	downsampling	diff_mean	diff_sd	ratio_mean	ratio_sd
GWAS	10%	No	-61.3	6.90	0.737	0.0267
GWAX	10%	No	-51	6.46	0.781	0.0294
LT-FH	10%	No	0	0	1	0
LT-FH++	10%	No	10.9	5.57	1.05	0.0241
GWAS	5%	No	-56.2	6.21	0.648	0.0306
GWAX	5%	No	-36.3	5.56	0.773	0.0249
LT-FH	5%	No	0	0	1	0

LT-FH++	5%	No	6.5	2.55	1.04	0.0181
GWAS	10%	Yes	-17.7	4.57	0.809	0.0458
GWAX	10%	Yes	-22.7	4.37	0.755	0.0480
LT-FH	10%	Yes	0	0	1	0
LT-FH++	10%	Yes	15.7	4.57	1.17	0.0546
GWAS	5%	Yes	-6.1	2.60	0.839	0.0606
GWAX	5%	Yes	-5	4.71	0.876	0.113
LT-FH	5%	Yes	0	0	1	0
LT-FH++	5%	Yes	6	2.11	1.16	0.0625

Table S15: Table containing the absolute and relative difference between LT-FH and all other considered phenotypes, case-control status (GWAS), GWAX, and LT-FH++. The differences are shown for each parameter configuration. The default simulation setup was used with a heritability of 50% and 1000 causal SNPs.
